# Molecular Evolution and Diversification of Proteins Involved in miRNA Maturation Pathway

**DOI:** 10.3390/plants9030299

**Published:** 2020-03-01

**Authors:** Taraka Ramji Moturu, Sansrity Sinha, Hymavathi Salava, Sravankumar Thula, Tomasz Nodzyński, Radka Svobodová Vařeková, Jiří Friml, Sibu Simon

**Affiliations:** 1 Mendel Centre for Plant Genomics and Proteomics, Central European Institute of Technology (CEITEC), Masaryk University, Kamenice, 62500 Brno, Czech Republic; tarakaramji@gmail.com (T.R.M.); sravan.tula@gmail.com (S.T.); nodzynski@ceitec.muni.cz (T.N.); 2 National Centre for Biomolecular Research, Faculty of Science, Masaryk University, Kamenice, 62500 Brno, Czech Republic; 3 Department of Biomolecular Sciences, Weizmann Institute of Sciences, Rehovot 7610001, Israel; sansritisinha@gmail.com; 4 Repository of Tomato Genomics Resources, Department of Plant Sciences, University of Hyderabad, Hyderabad 500046, India; 5 Centre for Structural Biology, Central European Institute of Technology (CEITEC), Masaryk University, Kamenice, 62500 Brno, Czech Republic; 6 Institute of Science and Technology (IST Austria), 3400 Klosterneuburg, Austria; jiri.friml@ist.ac.at

**Keywords:** small RNA (smRNAs), Dawdle (DDL), Tough (TGH), Serrate (SE/ARS2), Argonaute (AGO), Dicer-Like (DCR/DCL), evolution, phylogeny

## Abstract

Small RNAs (smRNA, 19–25 nucleotides long), which are transcribed by RNA polymerase II, regulate the expression of genes involved in a multitude of processes in eukaryotes. miRNA biogenesis and the proteins involved in the biogenesis pathway differ across plant and animal lineages. The major proteins constituting the biogenesis pathway, namely, the Dicers (DCL/DCR) and Argonautes (AGOs), have been extensively studied. However, the accessory proteins (*DAWDLE (DDL)*, *SERRATE (SE)*, and *TOUGH (TGH))* of the pathway that differs across the two lineages remain largely uncharacterized. We present the first detailed report on the molecular evolution and divergence of these proteins across eukaryotes. Although DDL is present in eukaryotes and prokaryotes, SE and TGH appear to be specific to eukaryotes. The addition/deletion of specific domains and/or domain-specific sequence divergence in the three proteins points to the observed functional divergence of these proteins across the two lineages, which correlates with the differences in miRNA length across the two lineages. Our data enhance the current understanding of the structure–function relationship of these proteins and reveals previous unexplored crucial residues in the three proteins that can be used as a basis for further functional characterization. The data presented here on the number of miRNAs in crown eukaryotic lineages are consistent with the notion of the expansion of the number of miRNA-coding genes in animal and plant lineages correlating with organismal complexity. Whether this difference in functionally correlates with the diversification (or presence/absence) of the three proteins studied here or the miRNA signaling in the plant and animal lineages is unclear. Based on our results of the three proteins studied here and previously available data concerning the evolution of miRNA genes in the plant and animal lineages, we believe that miRNAs probably evolved once in the ancestor to crown eukaryotes and have diversified independently in the eukaryotes.

## 1. Introduction

**Figure 1:**
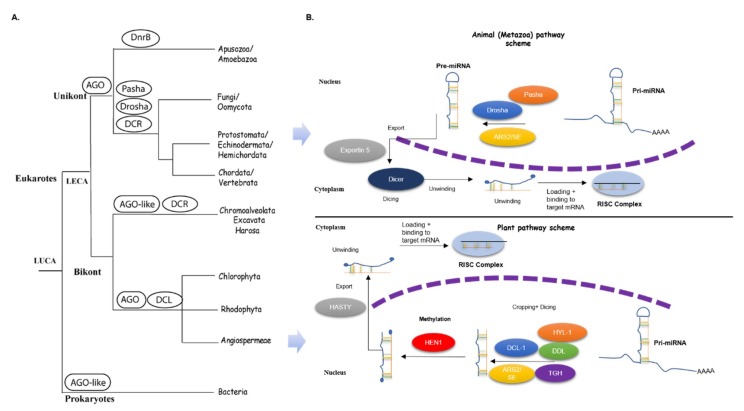
Distribution of key proteins (Dicer/Dicer-like (DCR/DCL)) and Argonaute (AGO) involved in the miRNA pathway in animal and plant lineages and the corresponding scheme of miRNA biogenesis in these lineages. (**A**) Tree of life representing the distribution of major proteins of the miRNA pathway (Dicer/Dicer-like (DCR/DCL)) and Argonaute (AGO): The presence and absence of DCL and AGO in various lineages is shown (adapted from work in [8,9,10,11]). AGO and DCLs appear in most eukaryotic lineages. The pattern of evolution, however, is not known for the accessory proteins (*DDL*, *SE*, and *TGH*) involved in the smRNA machinery. (**B**) Schematic of the miRNA pathway in animals and plants: The proteins involved in various steps of the pathway and the cellular compartments corresponding to miRNA processing in both lineages are shown. As is evident from this figure, miRNA biogenesis differs in two major aspects between the two lineages, one of them being the methylation of pri-miRNAs and the other being the cytoplasmic processing of mature miRNA, both of which are specific to the plant lineage. In animals, methylation is absent except for in piwi-interacting RNAs (piRNAs) and the maturation of miRNAs occurs in the cytoplasm itself.

Small RNAs (smRNAs) play a major role in regulating gene expression in eukaryotes. They are 19–25 nucleotides long and originate from the processing of long RNAs. smRNAs are classified (based on (i) the biogenesis pathway adopted and (ii) the genomic loci from which they are generated) into microRNAs (miRNAs), small interfering RNAs (siRNAs), trans-acting RNAs (tasi-RNAs), natural antisense miRNAs, and siRNAs [1,2,3,4,5]. The smRNAs are synthesized from the cleavage of perfect or near-perfect double-stranded RNA (dsRNA) by the RNA-induced silencing complex (RISC). The difference in the biogenesis of each class of smRNAs primarily lies in their precursors and the enzyme complex involved [3,4,5]. The components of RISC constituting smRNAs biogenesis are the RNA-dependent RNA polymerases (*RDRs*), Dicer-like proteins (ribonuclease III domain-containing proteins, *DCLs*), and Argonaute (*AGOs*) [3,6,7]. Generally, each class of smRNAs is associated with the enzyme complex involved in its biogenesis, and both smRNAs and *AGO/DCLs* complexes are known to recognize and bind to the precise target mRNAs to regulate fine-tune expression. The molecular evolution of *AGO* and *DCRs* have been studied together as well as separately in plant and animal lineages [8,9,10,11]. Both the proteins have been shown to undergo lineage-specific domain addition/deletion/rearrangements in the eukaryotic lineages. This has resulted in a varied number of functionally divergent *AGO* proteins in both unikont and bikont lineages [12]. The *DCR* protein has undergone functional divergence in most major unikont lineages as a result of domain rearrangements (for instance, the presence of a specialized *DCR*, *DnrB*, in Amoebazoa) and gene duplication (in the opisthokonts lineage; giving rise to nuclear (Drosha and Pasha) and cytoplasmic Dicer, respectively [8,9,11]). The distribution of these proteins across the unikont and bikont lineages of eukaryotes is summarized in Figure 1A.

miRNAs are short endogenous sequences ranging from 19 to 25 nucleotides in length generated from imperfect stem-loops from the primary transcripts of miRNA genes [13]. These have been shown to be involved in the regulation of genes participating in a plethora of intracellular signaling pathways, including the mRNAs of proteins controlling various developmental processes in plants and animals [14,15]. The miRNAs regulate the expression of target genes in response to developmental and environmental signals, either post-transcriptionally (transcript degradation) or translationally (inhibition of protein synthesis by *AGO* proteins) [14]. In *Arabidopsis thaliana*, the miRNA genes mostly exist as independent transcription units [16], with the exception of a few that either originate from the intronic region of other genes (for example miR838 that resides in the 14th intron of *DCL1)* or from splice junctions [7]. To date, there have been no reports of shared miRNAs being observed between plants and animals. Within the plant lineage, however, a recent study revealed three miRNAs (namely, miR160, miR166, and miR408) conserved between the liverwort *Pellia endivifolia* and the green alga *Chlamydomonas reinhardtii* miRNAs [17] inferring a convergent origin within virdiplantae (liverworts and green alga).

The transcripts of genes encoding miRNAs are transcribed by RNA polymerase II [18,19,20]. In animals, although the biogenesis of miRNA occurs in the nucleus, the maturation of miRNAs occurs in the cytoplasm (Figure 1B). Each step in the synthesis of miRNAs is tightly regulated according to external and developmental stimuli. The primary transcript generated is known as the primary miRNA (pri-miRNA). This forms a stem-loop structure, which is recognized by the Drosha, an RNase III [21,22]. In animals, Drosha interacts with DiGeorge Syndrome Critical Region 8 (*DGCR8*), which has two dsRNA-binding domains (dsRBDs) and assists Drosha in substrate recognition [23,24]. The Drosha forms a microprocessor complex with interacting factors, cleaves the pri-miRNA with a 2-nucleotide overhang at the 3′end [6,25]. This short overhang is thought to be a primary determinant of the subsequent processes in miRNA biosynthesis [26,27].

However, Drosha and DGCR8 are absent in plants. Instead, *DCL*, along with other factors, plays a major role in miRNA biogenesis in plants. There are four homologs of DCLs in Arabidopsis, *DCL1*, *DCL2*, *DCL3*, and *DCL4*, which produce ~21, 22, 24, and 21 nucleotide long small RNAs, respectively [28,29,30]. Unlike animals, in plants the entire process of miRNA biosynthesis and maturation is limited to the dicing bodies (D-bodies) of the plant nucleus [31]. Dicer-like 1 (*DCL1*) along with its interacting proteins, HYL1 (Hyponastic leaves 1, an RNA-binding protein) [23,32], *DDL* (DAWDLE, which stabilizes the miRNA transcripts) [33], *SE* (SERRATE, a C2H2 zinc finger protein) [34,35], and *TGH* (TOUGH) [36] are known to be involved in most of the miRNA processing (Figure 1A). *HYL1* and *SE* physically interact with *DCL1* in the nucleus to improve the effectiveness and cleavage accuracy of *DCL1* [37,38]. *DDL* is thought to recruit the primary miRNA to *DCL1* via its fork-headed associated domain and helps in stabilizing the miRNA [33,39]. *TGH* is also an RNA-binding protein and associates with the *DCL1* complex and assists *DCL1* to function efficiently to process pri-miRNA and pre-miRNA. *TGH* also aids in the interaction of pri-miRNA and *HYL1*. *TGH* is said to have a role in miRNA maturation as *TGH* mutants exhibited impaired *DCL* function with low levels of miRNAs and siRNAs [36]. The pri-miRNA is processed by the *DCL1* complex proteins to precursor miRNA (pre-miRNA) and then to the miRNA duplex, where the stem loop is removed from the pre-miRNA.

In animals, this miRNA duplex is then transported to the nucleus by exportin 5, a RAN-GTPase protein [22,40]. In plants, there is partial evidence that the miRNA duplex is transported through *HASTY (HST)*, a nuclear exporter [41]. Before the transportation of the miRNA duplex to the cytoplasm, the 3′ ends of the duplex are methylated by *HUA ENHANCER1 (HEN1)* [42]. The methylation event protects the duplex from uridylation and further decay [43,44,45]. The exported miRNA is transported either single-stranded or double-stranded and is now designated as mature miRNA. Once exported, the RISC binds to the dsRNA: one of the strands is loaded onto *AGO* proteins and associated proteins forming the RISC complex, and the other strand of the duplex is degraded in the cytoplasm (Figure 1B).

Multiple studies have shown that many genes involved in the smRNA pathway are conserved in plants and in animals. Previous studies on smRNA pathway genes were mostly focused on *DCLs* and *AGOs* (Figure 1A), and there is limited data on the accessory proteins involved in the smRNA pathway. This motivated us to study the molecular evolution of the accessory proteins involved in miRNA biogenesis.

In this study, we looked at the molecular evolution of the three major proteins (*DDL*, *SE*, and *TGH*) that are associated with miRNA biogenesis in plants across the tree of life. Previous studies with a comparative analysis of a limited subset of these proteins narrowed down our understanding of the structure–function relationship and functional divergence of these proteins across the tree of life [44,46,47,48,49]. Our in-depth comprehensive analysis is primarily focused on the evolution and diversification of these proteins in the plant lineage. The presence and absence of these proteins, and their respective domain architecture, changes across the lineages leading to functional diversification in eukaryotes are discussed in this study. This is further supported by the phylogenetic analyses, which provided insights into the neofunctionalization of these proteins in multiple lineages across the tree of life. These findings shed light on the presence/absence of specific features of the respective proteins in animals (Metazoa, unikonts) and plants (Plantae, bikonts). These lineages appear to be coherent with the previously reported differences in the miRNA biogenesis pathway.

## 2. Results

### 2.1. Identification and Annotation of DDL SE and TGH Orthologs across the Tree of Life

**Figure 2:**
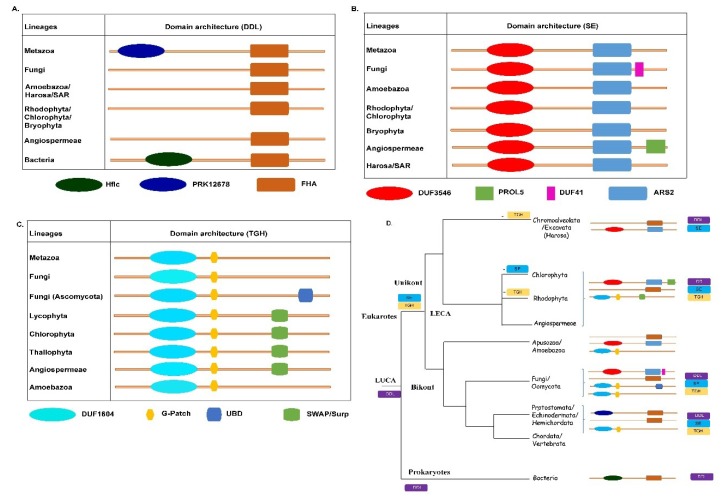
Domain architecture of *DDL* (**A**), *SE* (**B**), and *TGH* (**C**) proteins and their distribution across the tree of life (**D**). The distribution of domains for all three proteins across various lineages is shown. The index for the domain shapes is shown at the bottom of the figure. The interdomain region is shown in brown thick lines. The domains are not to scale and roughly represent the multiple sequence alignment. (D) A representative tree of life showing the lineages with the indicated presence and absence of proteins and the corresponding domain distribution.

To trace the evolutionary and structural dynamics of the miRNA biogenesis machinery, we examined three different factors (*DDL*, *SE*, and *TGH*) that facilitate miRNA biogenesis in the plant kingdom. The orthologous sequences of *DDL*, *SE*, and *TGH* proteins were obtained from eukaryotes (unikont and bikont lineages) and prokaryotes (clades are shown in Figure 2D) from the Uniprot, Phytozome, and NCBI databases using the homology-based BLAST method (Figure S1). Well-annotated protein sequences of these proteins were used as query sequences for sequence-based homology searches. All major lineages of eukaryotes that have been previously used for studying the molecular evolution of *DCL* ad *AGO* proteins were included in this study [8,9,10,11]. The *DDL* and *TGH* protein sequences of *Arabidopsis thaliana* and the *SE* protein sequence from humans were used as query sequences for blast searches. The final data set consisted of 76 *DDL*, 88 *SE*, and 131 *TGH* orthologs spanning all unikont and bikont lineages of eukaryotes and Gram-positive and Gram-negative bacteria group from prokaryotes (Tables S1–S3).

A single copy of *DDL* was identified across organisms of Opisthokonts, Amoebazoa, and in the recently sequenced genome of the apusozoan *Thecamonas trahens*. In the bikont lineage, a single copy of *DDL* has been identified in Harosa (Chromalveolates and excavates) lineages and in all Plantae lineages (including Rhodophyte, Thalophyte, Bryophyte, Cholorophytes, Embryophytes, and Angiospermae). In addition to eukaryotes, *DDL* orthologs were also identified from prokaryotic lineages of Gram-positive and Gram-negative bacteria. Thus, the data on the presence and absence of proteins across eukaryotes and prokaryotes suggests that *DDL* proteins emerged as a single copy in the Last Universal Common Ancestor (LUCA) and were subsequently distributed in prokaryotic and eukaryotic lineages (Table S1).

Serrate (*SE*), an RNA effector molecule homolog also known as arsenite-resistant protein 2 (ARS2), plays a major role in facilitating biogenesis. *SE* orthologs are distinctive with DUF3546 and ARS2 domains. A single copy of *SE* was identified across organisms of Opisthokonts, Amoebazoa, and in the recently sequenced genome of the apusozoan *Thecamonas trahens*. In the bikont lineage, a single copy of *SE* was identified in Harosa (Chromalveolates and excavates) lineages and in most Plantae lineages (including Rhodophyte, Thalophyte, Bryophyte, Embryophytes, and Angiospermae). A few lineages of plants (in particular the grasses and the bryophytes) exhibited the presence of multiple SE proteins, indicating the presence of in paralogs in such lineages. *SE* orthologs could not be identified in prokaryotes, suggesting that *SE* proteins emerged as a single copy in the Last Eukaryotic Common Ancestor (LECA) and were subsequently diversified in most eukaryotic lineages (Table S2).

*TGH* orthologs were only identified in the eukaryotic lineages. In the unikonts, a single copy of *TGH* orthologs was identified in Metazoa, whereas two copies of *TGH* were identified in Fungi. In the Plantae lineage, *TGH* orthologs were not found in rhodophytes. In the eukaryotes, orthologs of *TGH* were identified in most lineages of Plantae, with the exception of early-diverging rhodophytes, suggesting that *TGH* appeared first in the chlorophyte’s lineage of viridiplantae. Alternatively, the gene encoding the *TGH* protein was lost in the chlorophytes. The data from the presence and absence of *TGH* orthologs across the tree of life suggests that like *SE*, *TGH* appeared as a single copy gene in the LECA (Table S3).

### 2.2. Conservation of Domain Architecture in DDL SE and TGH Orthologs

Domain architecture plays a key role in understanding the functionality of proteins along with their evolutionary history. To gain further insights on the gain and loss of different domains in these three proteins, we inspected the domains in each clade and their associated functionally divergent residues.

All unikont and bikont *DDL* orthologs share the presence of a single FHA domain (Figure 2A). The arrangement of residues and the associated secondary structure elements in the FHA domain-containing *DDL* is conserved across eukaryotes and prokaryotes, with some exceptions where additional N-terminal domains are found conjugated with the FHA domain in DDL proteins. For instance, in Cyanobacteria, *Coleofasciculus chthonoplastes* (NCBI: WP_006106057), and in *Mycobacterium tuberculosis*, the FHA domain is found in conjugation with the N-terminal Hflc domain (Regulation of protease activity, stomatin/prohibitin superfamily, PFAM: COG0330). Similarly, in the unikonts, particularly in the vertebrates, FHA occurs in combination with the N-terminal PRK12678 domain (PFAM: cl36163) (Figure 2A).

Serrate has a signature domain architecture with DUF3546 and ARS2 (Figure 2B). The arrangement of residues and the associated secondary structure elements in DUF3546 and ARS2 domains containing SE proteins is conserved across eukaryotes with some exceptions. For instance, in plants, the *Arabidopsis thaliana* signature SE domain architecture is found in conjugation with the C-terminal PROL5-SMR domain (Regulation of protease activity, stomatin/prohibitin superfamily, PFAM: cl24055). Interestingly, of the three orthologs of SE identified in the bryophyte *Physcomitrella patens* (Phytozome ID: Pp3c11_5370V3.1), one lacks the N-terminal DUF3546 domain. Similarly, one of the two in paralogs in both *Malus domestica* (Phytozome ID: MDP0000770341) and *Ananas comosus* (Phytozome ID: Aco008281.1) lacks the DUF3546 domain, which appears to be a result of independent domain loss events in these lineages. In fungi, the *SE* orthologs harbor a DUF4187 domain (PFAM: pfam13821) between the DUF3546 and ARS2 domains. The presence of additional domains in *SE* orthologs in plants and fungi indicate probable domain addition events in these lineages. The function of *SE* proteins is conserved across eukaryotes. Therefore, the observation of sequence divergence in residues constituting the functional domain of *SE* orthologs across different lineages suggests lineage-specific adaptations in these proteins (Figure 2B).

All *TGH* orthologs share an uncharacterized 80 amino acid (aa) long DUF1604 domain (pfam07713) (38–120 in *A. thaliana TGH* orthologs, 31–116 in human *TGH* ortholog) and 40 aa long G-patch domain (pfam01585) (159–199 in *A. thaliana TGH* orthologs, and 152–184 in human *TGH* ortholog) (Figure 2C). In the plants, the DUF1604 domain and G-patch domain occur in combination with a C-terminal Suppressor-of-white-apricot (SWAP) domain (50 aa long, 403–453, *A. thaliana TGH* ortholog; also known as Surp domain), suggesting the occurrence of a lineage-specific domain addition event in the native TGH domain architecture (containing the DUF1604 domain and G-patch domain). Consistent with the previous study, we found an occurrence of three and five conserved Gly residues in all plant and metazoan *TGH* orthologs (Supplementary Figure S2). Also, the two Gly residues are present in the G-patch domain of all unikont *TGH* orthologs, but are absent in the bikont *TGH* orthologs (shown by red-colored stars in Supplementary Figure S2). In addition, a specific Gly residue is present in the fungal and plant *TGH* orthologs, but absent in metazoan *TGH* orthologs (shown by cyan-colored stars in Supplementary Figure S2). The presence of the SWAP domain in plant *TGH* orthologs may contribute to their functional specificity in the plant miRNA biosynthesis and maturation pathway (Figure 2C). The domain organization of *TGH* orthologs in the Opisthokont/Metazoa is conserved with the exception of primate *TGH* orthologs from human (NP_060495.2), *Pan troglodytes* (XP_512571.3), and *Macacca Mulatta* (XP_014978991.2) that harbor the DAGK-cat domain (Diacylglycerol kinase catalytic domain; cl01255) in the C-terminus (region 403–485, human *TGH*). However, it is not a conserved feature in this lineage. It appears to be a primate-specific variant of the SWAP domain of plant *TGH* orthologs and the presence of DAGK-cat domains suggests the neofunctionalization of *TGH* in certain organisms of primate lineage.

In fungi, two distinct domain combinations of *TGH* orthologs are observed. Although the *TGH* orthologs from Basidiomycota (club fungi), Zygomycota (bread molds), and Chytridomycota (chytrids) have a DUF1604 domain with two C terminal additions, namely, the G-patch and ubiquitin-binding domain (UBD), only the *TGH* orthologs from the Ascomycota lineage of fungi (yeast, sac fungi, and filamentous fungi) share the domain architecture with metazoan *TGH* orthologs. Given the fact that chytrids, bread mold, and club fungi appeared earlier than the more recent filamentous fungi, the absence of UBD in Ascomycota appears to be a result of the domain loss event in the *TGH* orthologs of this lineage (Figure 2C). In summary, the *TGH* protein appears to have undergone multiple independent lineage-specific neo-subfunctionalization events in eukaryotes.

The absence of domain shuffling events in *DDL*, *SE*, and *TGH* orthologs suggests that these proteins probably evolved under strong negative selection pressure.

### 2.3. Phylogenetic Classification of miRNA Biogenesis Factors

**Figure 3:**
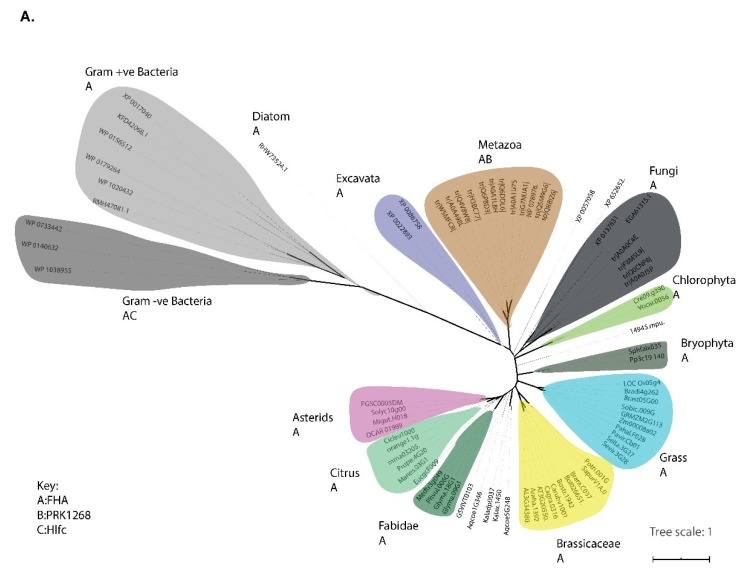

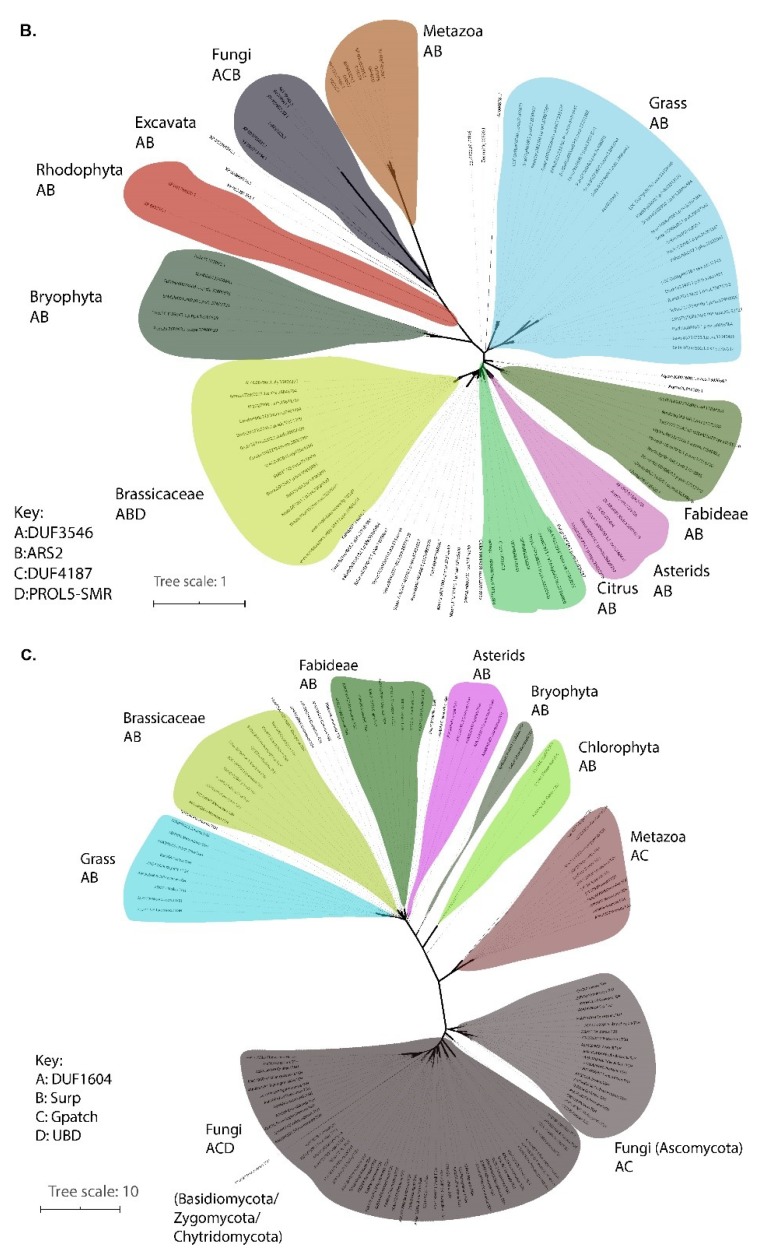
Reconciled maximum likelihood (ML) and Bayesian phylogenetic trees of *DDL* (**A**), *SE* (**B**), and *TGH* (**C**). The well-supported clades (PP > 90 and bootstrap values >900) are shown by thick black lines in the tree. The lineage-specific clusters for each protein are marked in different colors, and the corresponding domain architecture for the three proteins in each lineage is shown as alphabetical codes. The index for the domain is shown in the lower left corner of the figure. IQ-Tree and Mr.Bayes (CIPRES cluster) were used to run the ML (1000 bootstrap replicates) and Bayesian trees, respectively. The fungi cluster containing the DUF1604 and the SWAP domain of *TGH* orthologs is comprised of Ascomycota lineage fungi alone. The other lineages of fungi including the Basidiomycota, Zygomycota, and Chytridomycota contain *TGH* orthologs with a distinct domain architecture, as shown in Figure 3C.

The phylogenetic trees of *DDL*, *SE*, and *TGH* proteins were inferred using the ML and Bayesian methods to understand their evolutionary history. For *DDL* orthologs, ML and Bayesian trees were generated using a full-length protein alignment as well as an alignment containing the FHA domain region alone. Our phylogenetic analysis of *DDL* showed a distinct cluster of different lineages of eukaryotes and prokaryotes (Figure 3A). Interestingly, ML and Bayesian trees generated from the full-length protein alignment and the region corresponding to the FHA domain alone shared similar topologies, suggesting that the residues constituting the FHA domain have evolved in parallel with the residues constituting the full-length proteins. The hypothesis of the emergence of *DDL* proteins in LUCA is further supported by the conservation of the domain architecture of the FHA domain-containing *DDL* proteins across eukaryotes and prokaryotes, and from the phylogenetic analyses of *DDL* proteins. Together, the results from the presence and absence of genes, conservation of domain architecture and phylogeny suggest that *DDL* proteins emerged in LUCA and subsequently diversified across prokaryotic and eukaryotic lineages with sequence variations in the FHA domain.

Distinct lineage-specific clusters of *SEs*’ orthologs were obtained in both ML and Bayesian trees. The phylogenetic analysis points to the lack of well-defined gene duplication patterns of *SE* orthologs (particularly in bryophytes), suggesting that the gene encoding *SE* proteins duplicated independently in this lineage. Interestingly, the observation of distinct sub-clades of *SE* in paralogs in grasses is indicative of previously unknown probable neofunctionalization after gene duplication events in these lineages. The hypothesis of the emergence of *SE* proteins in the LECA is further supported by the conservation of domain architecture across the eukaryotes and by their phylogenetic analyses (Figure 3B).

Similar to *DDL* and *SE* orthologs, lineage-specific clusters were identified in both Bayesian and ML trees of *TGH* orthologs. Interestingly, unlike the trees of *DDL* and *SE* orthologs, two well-supported distinct clusters of fungal *TGH* orthologs were observed in the phylogenetic tree. The two distinct clusters of fungal *TGH* orthologs correspond to the Ascomycota *TGH* orthologs and Basidiomycota, Zygomycota, and Chytridomycota *TGH* orthologs (Figure 3C). Therefore, the data from the presence/absence of the three proteins and the conservation of the domain architectures connected to their emergence is consistent with the phylogenetic analysis of the three proteins.

### 2.4. Functionally Divergent Residues Were Identified in the FHA Domain of Metazoan and Green Plant DDL Orthologs

**Figure 4:**
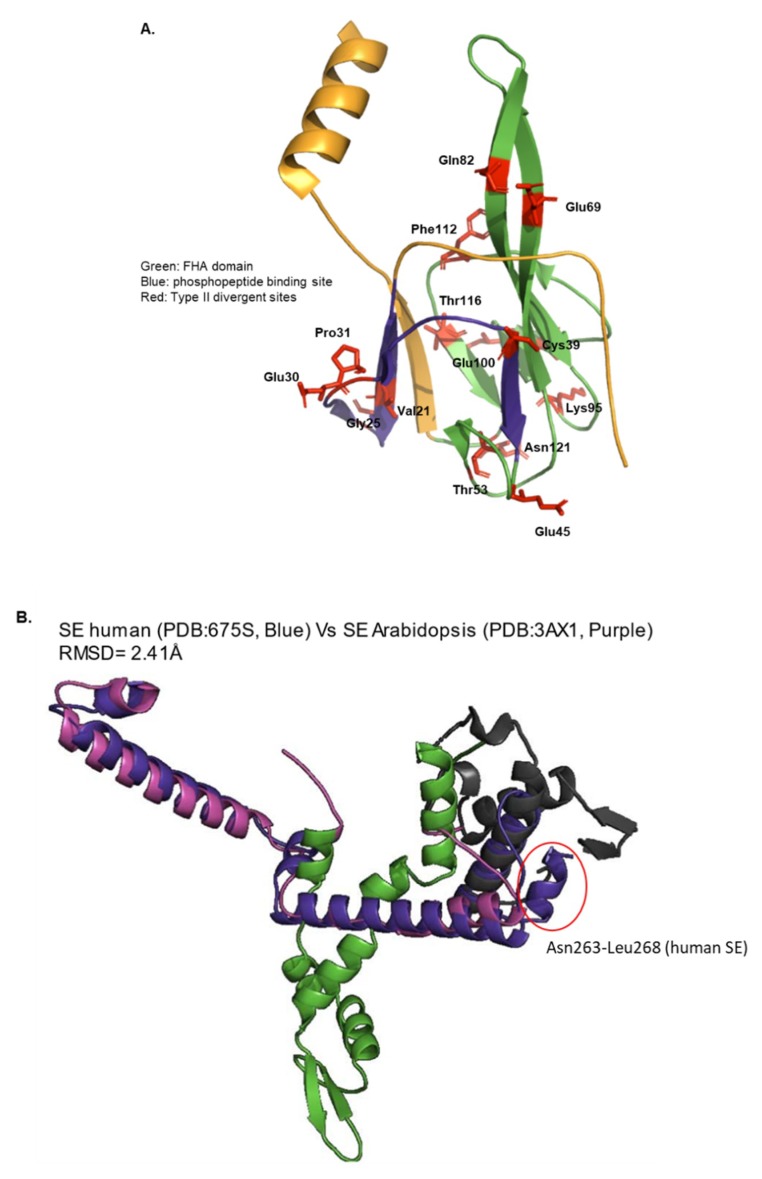
Functional divergence test for *DDL* (**A**) and *SE* (**B**) protein sequences using DIVERGE 2.0. Amino acids substitution rates among different classes (type II divergence) are shown in red (in stick form) in *DDL* (A), these residues were identified in the phosphopeptide binding region (V21 (PP = 4.269792), P31V (PP = 4.269792), and K95G (4.269792)) (details in Table 1) and (B) ARS2 domain overlap of *SE* in humans, and *A. thaliana* (B) shows the sequence divergence-driven structural differences within the DUF3546 domain in the C-terminal region from Asn263 to Leu268 (Human). This region may contribute to the divergent functional roles of *SE* in the plant and animal lineage. The PDB IDs of the structures and the colors used for the analysis are mentioned in the figures. In panel B, the structural homology between the structures of the two *SE* orthologs is depicted by RMSD values. FATCAT server was used for structural superposition. All figures were prepared using PyMol.

The occurrence of functionally divergent residues in crucial regions of protein structure is a common phenomenon often attributed to functional divergence across orthologous sequences. When an amino acid at a position in one group is replaced by another amino acid, and the groups have different physicochemical properties, this is known as type II divergence across proteins. A type II divergence in functional motifs often contributes to divergent physicochemical properties. The functional domains of the three proteins examined in this study were analyzed for the presence of type II divergent sites. The FHA domain appears to have undergone sequence divergence in residues constituting an epitope which has specificity for phosphothreonine-containing epitopes. Functionally divergent residues were identified in the FHA domain, specifically in the phosphopeptide binding region (V21P (PP = 4.269792), P31V (PP = 4.269792), and K95G (4.269792)). We identified residues exhibiting type II divergence in the FHA domain as well as in the phosphopeptide binding region of *DDL* proteins (Table 1). This is probably indicative of the possible neofunctionalization of *DDL* proteins across the unikont and bikont lineages. Alternatively, the identified residues may indeed contribute to the differences in the binding affinity of the phosphopeptide binding region to the phosphothreonine-containing epitopes (ligand). The occurrence of functionally divergent residues across *DDL* orthologs may account for the functional divergence of *DDL* proteins across unikont and bikont lineages. The residues constituting type-II functional divergences have been mapped onto the crystal structure of the FHA domain of the *DDL* ortholog from *A. thaliana* (PDB:3VPY) (Figure 4A).

Our analysis for identifying functional divergence began with a comparison of the crystal structures of *A. thaliana* (PDB:3AX1) and human (PDB:6F7S) *SE* orthologs. The two structures superposed well, indicating high structural homology (RMSD = 2.41Å). Sequence divergence-driven structural differences within the DUF3546 domain were observed in the C-terminal region corresponding to Asn263 to Leu268 of the human *SE* sequence. Our combinatorial sequence and structural analyses suggest high structural homology, despite sequence divergence corresponding to the DUF3546 of the two *SE* orthologs (Figure 4B, Supplementary Figure S3). Our analyses for the identification of functionally divergent residues in *TGH* proteins identified a single type-II divergent residue in the DUF1604 domain (Arg54 of *A. thaliana TGH* sequence; PP = 1.902) and SWAP domain (Tyr 451, *A. thaliana*, PP = 1.02). Other residues identified for type-II divergence lie in the interdomain regions. The absence of available structures restricted our analyses of these residues in terms of elucidating probable structure–function relationships.

## 3. Discussions

smRNAs constitute an essential population of eukaryotic non-coding RNAs. The availability of genomic and proteomic data combined with technical advances in bioinformatic approaches has expanded our understanding of several crucial proteins across organisms of different lineages including non-model organisms. Given the fact that smRNAs contribute to fine-tuning gene expression in a variety of cellular processes, the hypothesis of common or independent origin of proteins involved in smRNA biogenesis and signaling pathways in plants and animals has always remained an area of general interest.

The data on the similarities in the miRNA biogenesis pathway and the key proteins involved (*AGOs* and *DCLs*) suggest a common origin of these in eukaryotes. The differences in the pathways across the animal and plant lineages are suggestive of the functional diversity of the proteins participating in the miRNA pathways across these lineages. We focused on the three accessory proteins involved in this pathway, namely, *DDL*, *SE*, and *TGH*. In our study, we demonstrated the common origin of these proteins in eukaryotes. The presented data that suggest a common evolutionary origin supports the observed diversification of the proteins in plants and animals in terms of the differences in miRNA biogenesis and signaling in these lineages.

**Figure 5:**
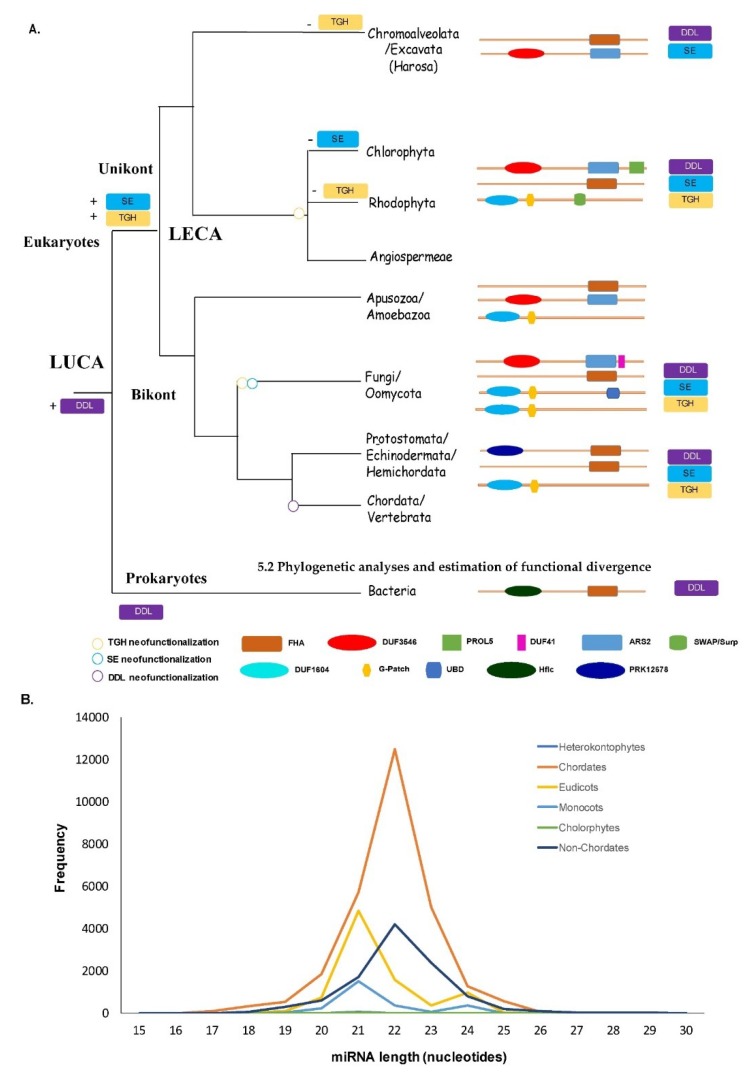
Proposed evolutionary history of miRNA processing factors (**A**) and size distribution of the mature miRNAs in different clades (**B**). (A) The distribution and divergence of DDL, SE, and TGH orthologs are shown across the tree of life. The presence and absence of the three proteins and their domain organization in each lineage are shown. The index at the bottom of the figure provides details on the symbols used for marking different domains and neofunctionalization of the three proteins. The index for the symbols used is shown in the lower left corner of panel A. (B) The miRNA-length (*X*-axis) and the miRNA-frequency (*Y*-axis) in various lineages are color-coded. The index for the colors used for various lineages is shown on the right side of the graph. The lineages chordates and non-chordates (including the protostomes, echinoderms, and hemichordates) are the subgroups of Metazoa of unikonts, whereas the lineages eudicots, monocots, and chlorophytes constitute the Viridiplantae lineage of bikonts.

In this study, we identified orthologs of *DDL*, *SE*, and *TGH* from both unikonts and bikonts, supporting the hypothesis of the common origin of elements of the miRNA pathway in eukaryotes. Our protein sequence and structure-based phylogenetic analyses reveal that these proteins were inherited from ancestral proteins in the LECA and have evolved independently in all eukaryotic lineages (Figure 3 and Figure 5A). The hypothesis of independent lineage-specific diversification of these proteins is supported by the differences in the domain architecture of these proteins. The events corresponding to domain addition/deletion were observed in specific lineages of eukaryotes. For example, the addition of the PRK12678 domain in the metazoan *DDL* orthologs, the addition of the PROL5 domain in angiosperm *SE* orthologs and the addition of the SWAP domain to the angiosperm *TGH* orthologs. The presence of the specific domains in the *TGH* and *SE* sequences in angiosperms indicates the neofunctionalization of these proteins in this lineage.

The biochemical data generated by site-specific mutagenesis for *SE* is lacking. The *Se-1* mutant (which lacks seven nucleotides in the 1st intron of *SE* mRNA) has been shown to regulate leaf patterning and miRNA regulation by regulating the expression of *PHABULOSA (PHB)* and *KNOX.* Also, *SE* and *HYL1* interact with *DCL1* and are crucial in miRNA biogenesis. In addition, it is also speculated that SE may also be involved in tasiRNA biogenesis [34,39,50]. The molecular evolution of *HYL1* suggests that, similar to *DDL*, *SE*, and *TGH* proteins, *HYL* is also present in most eukaryotic lineages including the Angiosperms of bikonts and the Metazoa of unikont lineage [51]. *SE* null alleles are embryonically lethal in *A. thaliana* and share functional roles in both plants and animals. The conserved core regions (residues 195–543 of *A. thaliana*) [39] with the domains (Figure 4B) play a major role in the interaction with *HYL1* and *DCL1* [39,50].

The previous comparative sequence analyses on a limited subset of the divergent FHA domain-containing genes suggests that these phosphopeptide binding proteins may have evolved early in eukaryotes and have lineage-specific divergent functions [48]. *DDL* encodes a forkhead-associated (FHA) domain-containing proteins that interact with the *DCL1* to regulate miRNA and endogenous siRNA biogenesis in *A. thaliana* [39]. FHA domain-containing *DDL* proteins bind to ssRNAs and prevent the degradation of pri-miRNA. *DDL* in *A. thaliana* also controls several aspects of organ development. Screens for insertional mutations in other Arabidopsis FHA domain-containing genes identified mutants with pleiotropic defects, such as plants with defective *DDL* that produce defective roots, shoots, and flowers, and have a reduced seed set [52]. *DDL* is also known as Smad nuclear interacting protein 1 (*SNIP1*) in humans. This FHA domain-containing protein functions as an inhibitor of the TGF-β and NF-κB signaling pathways by competing with the TGF-β signaling protein Smad4 and the NF-κB transcription factor p65/RelA for binding to the transcriptional coactivator p300 [53,54]. *SNIP1* interacts with the transcription factor/oncoprotein c-Myc and enhances its activity by bridging its interaction with p300 [55]. The results presented here on the comparative analysis of *DDL* sequences across the tree of life are consistent with those observed in the literature and point to specific residues that may account for the observed functional divergence of *DDL* proteins in animals and plants. Interestingly, residues contributing to functional divergence were identified in this region in our analyses (Table 1; Figure 4A) and may account for the functional divergence of *DDL* proteins across unikont and bikont lineages. A similar hypothesis may be suggested for the divergent DUF3546 domain of plant and metazoan *SE* orthologs (Figure 4B).

The evolutionarily conserved *TOUGH (TGH*) protein is a novel regulator required for *A. thaliana* development. The G-patch (with a series of conserved Gly residues) are exclusively found in proteins involved (predicted or known role) in RNA binding or RNA processing. The SWAP domain is a conserved domain with a presumed function in RNA binding that was first identified in the splicing regulator SWAP from *Drosophila melanogaster* [47,49] (Figure 2D). The G-patch and SWAP domains have only been found together in proteins with a role in RNA binding and RNA processing, inviting the hypothesis that SWAP and G-patch domain-containing proteins, and therefore *TGH* may play a role in these processes [46]. The ssRNA binding protein *TGH* promotes pri-miRNA processing and is characterized by the DUF1604 domain and the SWAP/Surf domain; its mutation leads to an increase in the accumulation of the pri-miRNA transcripts, which has been associated with developmental defects. *TGH* localizes to the nucleus. The presence of a conserved viridiplanteae-specific SWAP domain in TGH orthologs makes it a novel and important component of the DCL1-pri-miRNA complex. It has been shown to co-localize with SRp34 (splicing regulators) and has remained largely uncharacterized. Consistent with previous reports, we find that the *TGH* orthologs are highly divergent in the C-terminal regions across the unikonts and bikonts. In addition, as previously reported, we found conserved Gly residues; the G-patch is highly divergent across all eukaryotic *TGH* sequences [56] (Supplementary Figure S2). The currently uncharacterized Gly residues of the G-patch domain that are present in the unikont *TGH* orthologs may point to the neofunctionalization of *TGH* orthologs in this lineage.

We investigated the miRNA size differences based on the available data in miRBase. The frequency of the occurrence of varied lengths of miRNAs in different lineages was computed. Our results are consistent with the notion of an expansion of the number of miRNA-coding genes in animal and plant lineages that correlate with organismal complexity. miRNA evolution has been an area of growing interest. The shared and unique features of miRNAs in the plant and animal lineages have been recently reviewed [11,57]. In addition to previously known features, our analysis suggests that although miRNAs in plant lineages (eudicots and monocots) predominantly occur with a length of 21nt, in the metazoans, the average length of miRNAs is 22nt (Figure 5B). Whether this difference functionally correlates with the diversification (or presence/absence) of the three proteins studied here or the miRNA signaling in the plant and animal lineages is unclear. Based on our results of the three proteins studied here and previously available data concerning the evolution of miRNA genes in the plant and animal lineages [57], we believe that miRNAs have probably evolved once in the ancestor to the crown eukaryotes and have diversified independently in the eukaryotes. For instance, in plants, previous analysis has shown that the size distribution of the smRNAs was influenced by the presence of *NRPE1* (also known as *NRPD1*, nuclear RNA polymerase D1B), a DNA-directed RNA polymerase protein. There was a significant difference in the 24nt/21nt ratio between the species with an *NRPE1* homolog and those without. The use of high-throughput techniques like iCLIP [58] or high-throughput sequencing together with UV cross-linking and immunoprecipitation (HITS-CLIP) [59] can provide further insights towards our understanding their function and importance with single-nucleotide resolution, as well as also the binding of these ssRNA and dsRNA binding accessory proteins.

## 4. Conclusions

Given the limited functional characterization of the three proteins investigated here in plants and animals, our study represents their first comparative analysis in eukaryotes and points to functionally conserved and divergent regions in them. Of the three proteins studied here, only *DDL*s share homologs across prokaryotes and eukaryotes. Collating our findings with the available biochemical and functional data on these proteins, the presence/absence of specific domains of these proteins in plants strongly indicates their functional specialization in the miRNA biogenesis and signaling in this lineage. The data points out specific functionally divergent residues, which can be used for their functional characterization in the context of miRNA biogenesis and signaling pathways in plants. Furthermore, the presence of two kinds of domain architecture in *TGH* orthologs from fungi suggests that (i) the two types of *TGH* proteins may have additional functions in fungi, and (ii) the two kinds of *TGH* orthologs may perform distinct functions in fungi. A similar hypothesis of acquired distinct functions can be extended to the functionally divergent metazoan PRK12678 domain-containing *DDL* orthologs and currently uncharacterized G-patch domain-containing *TGH* orthologs. The loss of *TGH* in the Harosa and rhodophyte lineages and the loss of *SE* in the chlorophyte lineage, however, remain to be explained. In summary, the three proteins studied here appear to be monogenic and form monophyletic clusters on the phylogenetic tree, which is similar to the evolution of *DCLs* and *AGOs*. Similar to *DCLs* and *AGOs*, the *SE* and *TGH* orthologs were only identified in the eukaryotes [8,11]. However, unlike *DCLs* and *AGOs* that are present in all lineages of unikonts and bikonts, *SE* and *TGH* appear to be lost in basal plant lineages (chlorophytes and rhodophytes, respectively). Understanding the molecular evolution and coevolution patterns of all the proteins involved in the miRNA biogenesis pathway in eukaryotes will provide previously unidentified functional insights that will significantly enhance current understanding of the miRNA biogenesis and signaling pathway in eukaryotes. The data presented in this study aid our current understanding of the structure–function relationship of these proteins and paves a way for their functional characterization.

## 5. Materials and Methods

### 5.1. Homolog Mining Domain Architecture Analyses and Multiple Sequence Alignment

Orthologs of Dawdle (*DDL/DWL*) protein were mined using blast searches [60] (blastp, E-value cutoff of 0.01, percentage identity cut-off of 30% (for *DDL* and SE orthologs and 25% for *TGH* orthologs) from the Phytozome [61], NCBI (non-redundant protein database), and UniProt [62] protein databases using well-annotated protein sequences as queries. The well-annotated *DDL*, *SE*, and *TGH* orthologs from *Arabidopsis thaliana* (for *DDL* and *TGH* orthologs) and human (for *SE* orthologs) were used to identify *DDL*, *SE*, and *TGH* orthologs across the unikont and bikont lineages of eukaryotes and from the bacterial lineages of prokaryotes. Only full-length protein sequences were included in the study. The retrieved orthologs were refined using the program Cluster Database at High Identity with Tolerance (CD-hit) [63], with a word size of 5 and 99% identity as the clustering threshold to remove redundant sequences and pseudogenes. All *SE* orthologs contain the DUF3546 and the ARS2 domains. The identity of the orthologs was further ascertained by reciprocal blast and by analyzing the presence of conserved domains using PFAM and NCBI-CDD. For instance, all the *DDL* orthologs contain the FHA domain (Forkhead associated domain, PFAM: cl00062), which was used to further ascertain the identity of retrieved sequences using PFAM [64,65] and the NCBI Conserved Domain Database (NCBI-CDD).

Multiple sequence alignment was performed using MAFFT [66] (G-ins-1 strategy, BLOSUM62 matrix). Poorly aligned regions were either removed manually using the BioEdit tool or GUIDANCE2 tool [67] (100 bootstrap replicates with sequence cutoff 0.6 and column cut-off at 0.93). The certainty of the sequence-based alignment was confirmed using structurally informed sequence alignment. The alignment was printed using Espript 3.0 server [68] (File S2 (DDL), File S3 (SE), File S4 (TGH).

### 5.2. Phylogenetic Analyses and Estimation of Functional Divergence

The best-fit model for phylogeny determined from the IQTree server [69] was used for the phylogenetic analysis [70,71]. The computation of a best-fit amino acid substitution model based on AIC criteria [72] and the parameter values for the dataset was done using IQTree server. The phylogenetic trees corresponding to the alignments were run using ML, Bayesian, and aLRT strategies using IQTree server and PhyML server on the CIPRES cluster [73]. ML tree was run for 1000 bootstrap replicates. Bayesian tree, Markov Chain Monte Carlo (MCMC) analysis was used to approximate the posterior probabilities of the trees. The tree was run for 1 million generations using a stop value of 0.01. The initial 25% trees were discarded and data from the remaining trees were used to generate the consensus tree. All the trees were visualized and modified using the iTOL v3 online server [74].

The functionally divergent residues in the domain across the orthologs were identified using the DIVERGE 2.0 tool [75]. In this analysis, consisting of 30 sequences from the three proteins of interest, a value of θII and posterior probability (PP)>1 indicates type II functional divergence. These residues were mapped to the crystal structure of the *Arabidopsis thaliana DDL* protein (PDB:3VPY), human *SE* (PDB:6F7S), and *A. thaliana SE* (PDB:3AX1) to gain functional insights. The structural superposition of the two SE structures was done using FATCAT server (http://fatcat.sanfordburnham.org/).

### 5.3. miRNA Length Analysis

To decipher any lineage-specific differences in the mature miRNA length, the frequency of miRNAs of a particular length in all lineages was calculated. All the available mature miRNAs were extracted from miRBase v22.1 [76] and processed using an in-house shell script to calculate the frequency with respect to the length of the miRNAs (Figure 5B).

### 5.4. Deposition of the Phylogenetic Tree

The whole phylogenetic tree generated using IQTree is shared on the iTOL server [74] and is available in Newick format in the files S5 (*DDL*), S6 (*SE*), and S7 (*TGH*).

## Figures and Tables

**Table 1:** Sites displaying type II functional divergence across *DDL* orthologs in eukaryotes (Metazoa vs. Angiospermae). The numbers of residues corresponding to the *A. thaliana DDL* protein sequence.

Type II Divergence	
α_ML_	0.753156
θ_II_ ± SE	0.054996 ± 0.105161
G_R_/G_C_	0.612903/0.387097
N/C/R	101/24/38
F00, N/C/R	0.331288/0.024540/0.012270
Residues (PP)	V21P (4.269792)
	P31V (4.269792)
	K95G (4.269792)

θ denotes the coefficient of functional divergence. SE is the standard error. PP denotes the Posterior Probability for amino acid residues causing functional divergence. All residue positions correspond to the numbering in *A. thaliana* DDL. GR and GC denote the proportion of radical change and conserved change, respectively; F00, N; F00, R; and F00, C represent the proportion of no change, radical change, and conserved change of amino acids between clusters but no change within clusters, respectively.

## Supplementary Materials

The following are available online at https://www.mdpi.com/2223-7747/9/3/299/s1, Supplementary Figure S1: Summary of the pipeline adapted to study the evolution of Dawdle (*DDL*), Serrate (*SE*) and Tough (*TGH*) across the tree of life. Supplementary Figure S2: Multiple sequence alignment of G-patch domain of plant, fungal and metazoan *TGH* orthologs. The conserved Gly residues are indicated by numbers at the top of the figure. The Gly residues absent in metazoan and plant *TGH* orthologs are shown as cyan and red-colored stars, respectively. Supplementary Figure S3: Consensus of DUF3546 and ARS2 domains of eukaryotic *SE* proteins. A. Variability in DUF3546 domain across unikonts and bikonts of *SE* orthologs. B. Variability in DUF3546 domain across angiosperms, fungi, and metazoan *SE* orthologs. C. Variability in ARS2 domain across unikonts and bikonts of *SE* orthologs. Files S1–S3: Multiple sequence alignment of *DDL* (S1), *SE* (S2), *TGH* (S3) generated from Espript server, File S4–S6: Phylogenetic trees of *DDL* (S4), *SE* (S5), *TGH* (S6) in nwk format. Supplementary Tables S1–S3: Orthologs identified in the study Dawdle (*DDL*) S1, Serrate (*SE*) S2 and Tough (*TGH*) S3 across the tree of life.
