# Supplementary material for: Molecular Evolution and Diversification of Proteins Involved in miRNA Maturation Pathway
Source: Plants (Basel). 2020 Mar 1;9(3):299. doi: 10.3390/plants9030299 (PMC7154892; doi:10.3390/plants9030299)
Supplement: Supplementary file 1 [file plants-09-00299-s001.zip › Supplementary Files/Supplementary Figures_S1-S3.pptx]

## Slide 1
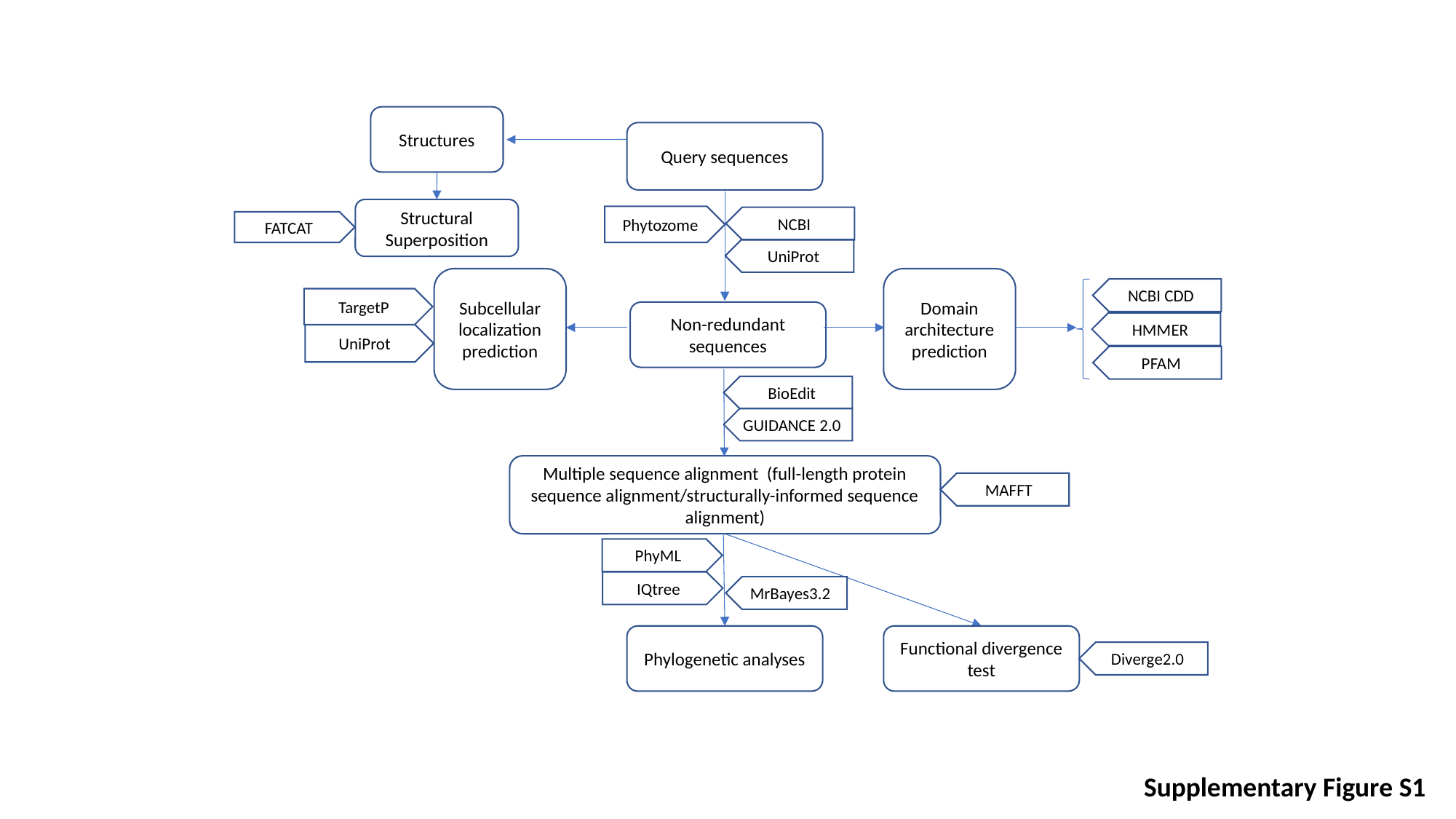

Structures
Query sequences
NCBI
UniProt
Subcellular localization prediction
Domain architecture prediction
NCBI CDD
TargetP
Non-redundant sequences
HMMER
PFAM
Multiple sequence alignment (full-length protein sequence alignment/structurally-informed sequence alignment)
MAFFT
PhyML
MrBayes3.2
Functional divergence test
Phylogenetic analyses
Diverge2.0
BioEdit
Phytozome
UniProt
Structural Superposition
FATCAT
GUIDANCE 2.0
IQtree
Supplementary Figure S1

## Slide 2
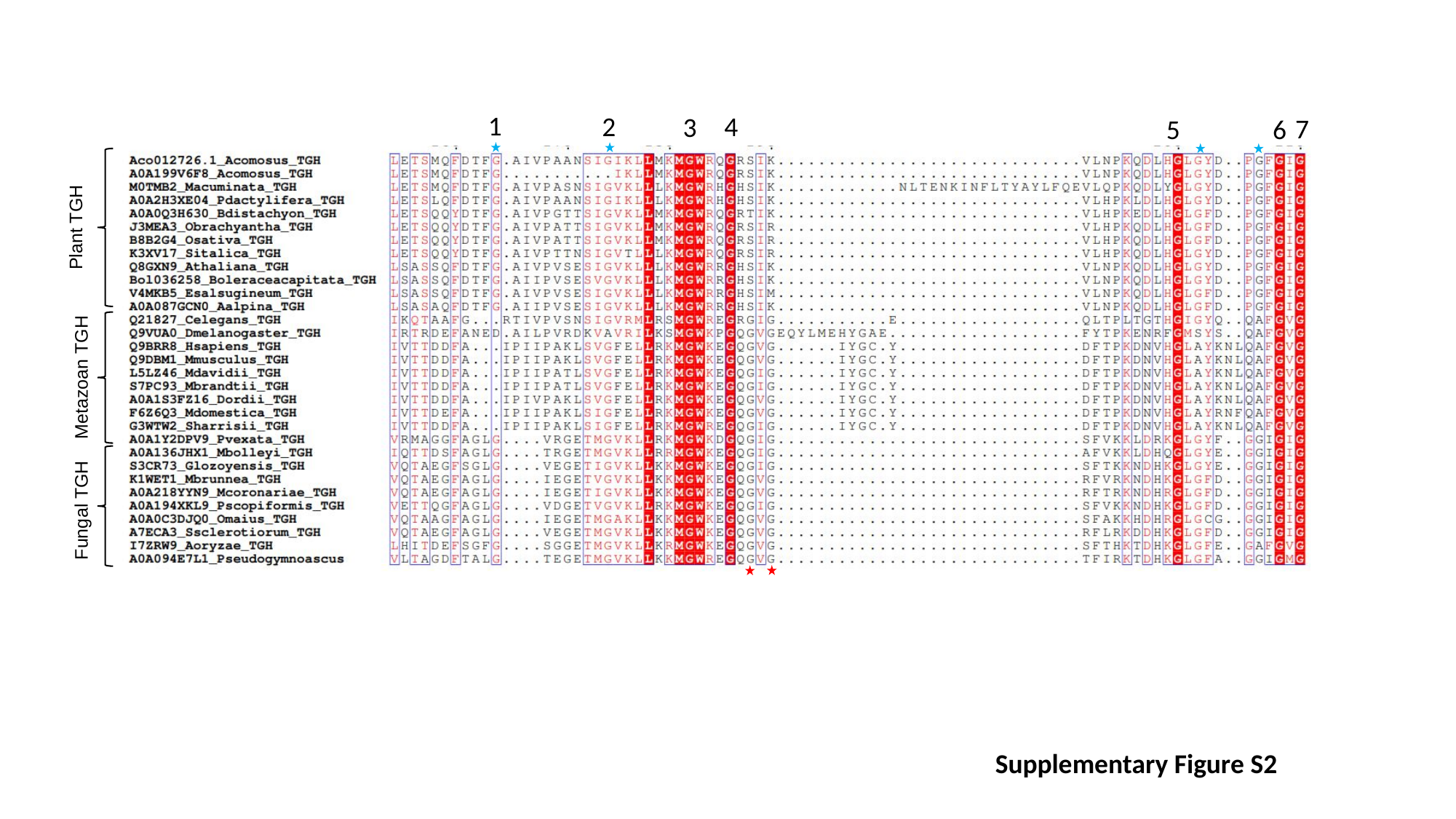

1
2
4
3
7
5
6
Plant TGH
Metazoan TGH
Fungal TGH
Supplementary Figure S2

## Slide 3
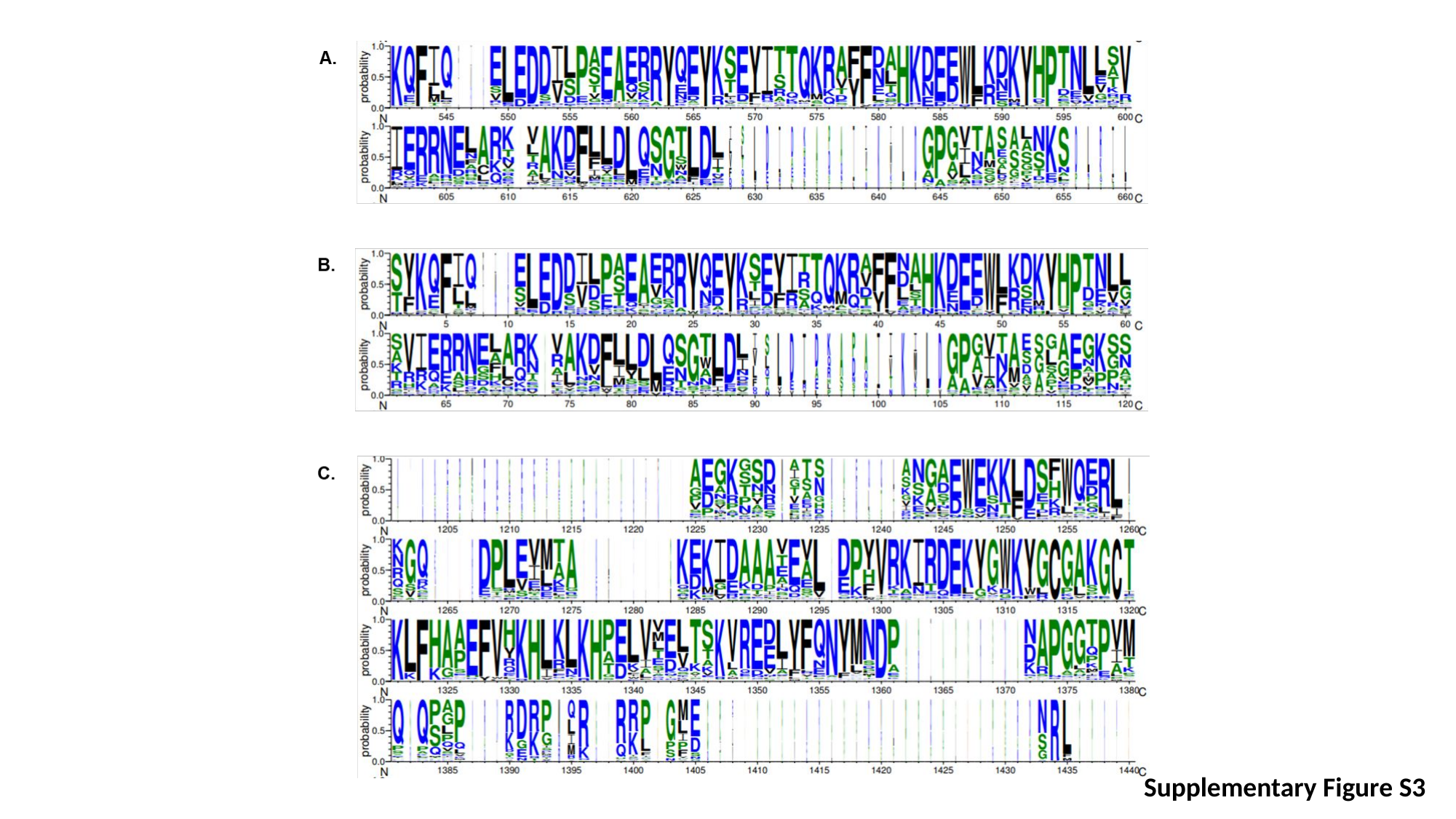

Supplementary Figure S3
