# Supplementary material for: Molecular Evolution and Diversification of Proteins Involved in miRNA Maturation Pathway
Source: Plants (Basel). 2020 Mar 1;9(3):299. doi: 10.3390/plants9030299 (PMC7154892; doi:10.3390/plants9030299)
Supplement: Supplementary file 1 [file plants-09-00299-s001.zip › Supplementary Files/Supplementary File S2.pdf]

Pp3c25_7680V3.1.p.ppa.32980429	1	MAD.....LPQ.EP.VVKEESRRDR.....S...RD....RGSED.................................
Pp3c16_11390V3.1.p.ppa.32986529	1	MAD.....LPQ.EV.VVKEESRRDR.....S...RD....RGFDD.................................
Sphfalx0012s0220.1.p.sfa.32627721	1	MADVM.D.VPP.GP.LLREDRRRDR.....SAERKD....RTLED.................................
LOC_Os02g05610.1.osa.33135686	1	MADVL.L.PEL.RS....................................PPPPPP....PPPPT............N
LOC_Os08g40560.1.osa.33102829	1	MAEVA.R...ASAS....PPP...............
Pavir.Fb01956.1.p.pvi.30278011	1	MAEDA.D.A..S....PPP...............
Pahal.F00690.1.pha.32488202	1	MAEDA.D.A..S....PPP...............
Sevir.6G221600.1.p.svi.32643744	1	MAEVA.D.A..S....PPP...............
Sobic.007G200600.1.p.sbi.37935124	1	MAEVA.D.A..S....PPP...............
GRMZM2G132780_P02.zma.31013431	1	MAEAA.D.A..S....PPP...............
Zm00008a003300_P01.zma.37201202	1	MAEAA.D.A..S....PPP...............
Bradi3g40630.1.p.bdi.32820467	1	MAEVA.D.APPRGT....................................PGASTS....PPP...............
Brast03G282200.1.p.bst.32857559	1	MAEVA.D.APARGT....................................PGASTS....PPP...............
Sobic.010G249100.1.p.sbi.37910925	1	MADVL.D.PAS.DA....................................PRARRP....PPPPP............D
GRMZM5G817439_P01.zma.30992522	1	MADVL.D.PAS.DA....................................PRARRP....PPPPP............D
Pavir.Da00165.1.p.pvi.30193584	1	MADVL.D.PSS.DA....................................PRARRP....PPPPP............D
Sevir.4G256700.1.p.svi.32645485	1	MADVL.D.PAS.DA....................................PRARRP....PPPPP............D
LOC_Os06g48530.1.osa.33147743	1	MADVI.D.PASTEA....................................PRARRP....PPPPP............D
Bradi1g33970.1.p.bdi.32801481	1	MADVI.D.PGS.EA....................................PRARRP....PPPPP............D
Brast07G193800.1.p.bst.32871232	1	MADVI.D.PGS.EA....................................PRARRP....PPPPP............D
Migut.N02418.1.p.mgu.28925732	1	MAEDL.T.ASE.EN.L...DRPQEI.....NATSDD....KPSDTASDTGPEATS.....PPP...............
Migut.D00714.1.p.mgu.28928758	1	MAEAL.NDAAV.EN.L...DSIGDQDTNANPSDDSN....KPSTTAADAVPDSTSS....PPPS..............
Medtr8g043980.1.mtr.31070730	1	MAEVL.TPIPS.DS.L...DQSS...........................PTTTSP....PPP...............
Tp57577_TGAC_v2_mRNA25888.tpr.35955570	1	MLYLTVS.....SS.F...DYS..
Medtr3g006760.1.mtr.31058945	1	MAEVE.N.LPP.ES.H...DRS............................PTITTA....PPP...............
Glyma.08G227300.1.p.gma.30540922	1	MAEVM.N.VPP.ES.L...DKS............................PSSSAP....PPP...............
Phvul.011G187000.1.p.pvu.37156720	1	MAEVM.N.VPP.ES.L...DKS............................PSSAAP....PPP...............
Phvul.005G010800.1.p.pvu.37152760	1	MAEVI.N.MPP.ES.L...DQS............................PSASAP....PPP...............
Glyma.06G314900.1.p.gma.30549408	1	MAEVINN.MPP.ES.L...DQS............................PSSSAP....PPP...............
Solyc01g009090.2.1.sly.36140441	1	MAEVM.D.APS.EG.L...ERRRER.....SKENPN....KSTEE.....PPVSSP....PPP...............
Thhalv10001919m.esa.20200705	1	MADVT.L.PPA.DS.V..................................PEKTTS..SSPPP...............
AT2G27100.1.ath.19641299	1	MADVN.L.PPS.DS.V...DNRL...........................PEKSTS..SSPPP...............
Q9ZVD0	1	MADVN.L.PPS.DS.V...DNRL...........................PEKSTS..SSPPP...............
Araha.4729s0005.1.p.aha.28848294		....
AL4G20790.t1.aly.35923270	1	MADIT.L.PPS.DSAV...DNRL...........................PEKSTS..SSPPP...............
Bostr.29505s0043.1.p.adr.30675300		....
Carubv10022733m.cru.20903468	1	MADVT.L.PPS.DS.V...DNRL...........................PEKSTS..SSPPP...............
AL4G20820.t1.aly.35924196	1	MADET.L.PPS.ES.V...DNRL...........................PEKSTS..SSPPP...............
Carubv10022734m.cru.20901991	1	MADVT.L.PPS.DS.V...DNSL...........................PEKSIS..SSPPP...............
Bostr.28794s0002.1.p.adr.30657126	1	MADVT.L.PQS.DS.V...DNRL...........................PEKST.......................
Brara.D01611.1.p.bra.30620383	1	MADVT.L.PPP.DS.V...DIRP...........................PEN.TP..SLPPP...............
Bol026461.bol.37343291	1	MADVT.L.PPP.DS.V...DIRP...........................PENTTT..SLPPP...............
Brara.G01299.1.p.bra.30635851	1	MADET.L.PPA.DP.V...DIPL...........................PEKTTS..LSPPP...............
Bol027793.bol.37343994	1	MADET.L.PPA.D.....................................PEKTTS..LS..................
mrna07673.1-v1.0-hybrid.fve.27266960	1	MAEIM.N.MPV.DS.L...DRRR.........DRKD....KSSEEPQSSSPQQAPQ..QQPPP...............
Prupe.6G153800.1.p.ppe.32086540	1	MAEIM.N.MPV.ES.L...DRRR.........DRKE....KSSEDP.QSSPQQEAP..PPPPP...............
GSVIVT01037454001.vvi.17842075	1	MAEVI.N.MPL.DS.L...DRR..........DGND....KSAEEQ....SQSQSP..SPPPP...............
orange1.1g003865m.csi.18122801	1	MAQVM.N.MAV.DS.L...DRRRDN.GNGNGDNED...S..........APQSSP.....PPP...............
Gorai.004G227500.1.gra.26773739	1	MAKVI.N.MPV.DS.L...DRRR.......GGDRKD..RQLPPSDD....PNLS......PPA...............
Thecc1EG042011t1.tca.27426134	1	MAEVI.N.MLV.DS.L...DRRR........GDRKDNNRQPPSSDD....PNSS......PPP...............
evm.model.supercontig_103.67.cpa.16404954		....
Potri.004G196400.1.p.ptr.26990875	1	MAEVI.N.MPV.DS.L...DRRGGSGGGGGGRDRKD....KPNTDD.....QSSP.....PPP...............
SapurV1A.0615s0230.1.p.spu.31434857	1	MAEVI.N.MPV.DS.L...DRRGGSGGGGGGRDRKD....KPNTDD...DPQSSP.....PPP...............
29942.m000751.rco.16814310	1	MAEVI.N.MPV.DS.L...DRRGRE......RDRKE....KSSADD....PQSSS.....PPP...............
Manes.11G111400.1.p.mes.32355670	1	MAEVI.N.VTV.DS.L...DRRS........REGKE....KSSADD....PQSSS.....PPP...............
Manes.S021700.1.p.mes.32354619	1	MAEVI.N.MPV.DS.L...DRRS........RERKD....KSSADD....PQSSS.....PPP...............
Eucgr.J00624.1.p.egr.32033287	1	MADVM.N.PPA.DS.L...DGPP.......EGKDKD....KSSDDPLLPPPQQQPAAQQPPPP...............
GSVIVT01027348001.vvi.17834761	1	MAQVM.N.MPL.DS.L...EKPDKS.....PSATAD....AVADDD.......SAP....PPP...............
Kalax.0225s0069.1.p.kla.32599097	1	MAEVA.N.ATI.DG.V...DRRKE................KSTEDD....PSASSPIHQQQQP...............
Kaladp0081s0182.1.p.kfe.35761984	1	MAEVA.N.ATI.DG.V...DRRKE................KSTEDD....PGASSPIHQQQQP...............
Kalax.0216s0065.1.p.kla.32585583	1	MAEVA.N.APA.DD.V...DRPQE................KSPEED....PNPSSPI.LGQQP...............
DCAR_002130.dca.36055141	1	........MAV.EP.L...ERRE.........DSKE....KPPPDE...RPASP......SPP...............
Aqcoe2G007600.1.p.aco.33056562	1	MAEVM.N.MPA.ET.L...DRRRER.....QSERKE....KSVDND.......LP.....PPP...............
Bradi3g03840.1.p.bdi.32813176	1	MARDVHD.RAL.SP....DDQRR.......................GCSQPQGPLP....PPPPL.GSSRPKRLVEDD
Brast04G302900.1.p.bst.32866594	1	MARDVFD.RAL.SP....DDQRR.......................GCSQPQGPLP....PPPPL.CSSFPKRLVEEE
Sevir.1G087000.1.p.svi.32667668	1	MEYVL.C.SPA.GS....................................PGPPSS..SLPPAP......P.......
Pavir.J22990.1.p.pvi.30308347	1	MEDVL.R.SPA.GS....................................PGPPSL....PPAAAAAGGAP.......
Pahal.A00287.1.pha.32525941	1	MEYAL.C.SPA.GS....................................PGPSSL....PPAA...GGTP.......
OAV97825.1	1	MSDLA.G........L................................GEPRLRS.....PSP...............
XP_003293253.1	1	MEEAV.......DE.F...GR.......................................PLR...............
XP_005713144.1	1	M...
XP_005702831.1	1	MSQGS.T.LTG.EQ................QVENN.........................PNL...............
XP_009857252.1	1	MDSSY.......HS.Y................................REGSARS.....PGDR..............
GES64941.1		....
KEY79445.1	1	MDSY........ES.F.......................G.....S..............P.................
XP_008869116.1	1	MDPAQ.......EE.L...DQSA.......QAEMHQA.......................QPP...............
Q66I22	1	MGDSD.......DE.Y...DRRR...
RXN03320.1	1	MADSD.......DE.Y...DRRR...
XP_015195966.1	1	M...
B1H1X4	1	MADSD.......DE.Y...DRRR...
NP_001362205.1	1	MADSD.......DE.Y...DRRR...
Q99MR6	1	MGDSD.......DE.Y...DRRR...
Q9BXP5	1	MGDSD.......DE.Y...DRRR...
XP_005549330.1	1	MGDSD.......DE.Y...DRRR...
H2ZUZ4	1	MGDSD.......DE.Y...DRRR...
XP_001748332.1	1	MAPRS...VAA........GLGL......................T.LVQPRGHA.....PAE...............
XP_002286542.1	1	MA....V.....DE.F...GREI.......QGSHHDAL.G.....S.GGDNALRSA....PPL...............
XP_649316.1	1	MQIEL.N........I..
consensus>70		mad...p.................

```
                                                                                                         .
Pp3c25_7680V3.1.p.ppa.32980429 27 ............................................RR.RDRDR.....RDDRD................
Pp3c16_11390V3.1.p.ppa.32986529 27 ............................................RR.RDRDR.....RDERE................
Sphfalx0012s0220.1.p.sfa.32627721 33 ............................................RRQRDRET.....REERE................
LOC_Os02g05610.1.osa.33135686 24 DDRAR..AVDLPSPT....PECD.......................................................
LOC_Os08g40560.1.osa.33102829 14 ............ASA...............................................................
Pavir.Fb01956.1.p.pvi.30278011 12 ............T.................................................................
Pahal.F00690.1.pha.32488202 12 ............T.................................................................
Sevir.6G221600.1.p.svi.32643744 12 ............T.................................................................
Sobic.007G200600.1.p.sbi.37935124 12 ............A.................................................................
GRMZM2G132780_P02.zma.31013431 12 ............P.................................................................
Zm00008a003300_P01.zma.37201202 12 ............P.................................................................
Bradi3g40630.1.p.bdi.32820467 22 ............PSS...............................................................
Brast03G282200.1.p.bst.32857559 22 ............PSS...............................................................
Sobic.010G249100.1.p.sbi.37910925 24 SPEDP..SPQLPPPP....PGGP.................PAASRK................................
GRMZM5G817439_P01.zma.30992522 24 SPEDR..SPQLPPPP....PGGP.................PAASRK................................
Pavir.Da00165.1.p.pvi.30193584 24 SPEDR..SPQLPPPP....PGGP.................PAASRK................................
Sevir.4G256700.1.p.svi.32645485 24 SPEDR..SPQLPPPP....PGGP.................PAPSRK................................
LOC_Os06g48530.1.osa.33147743 25 SPEGR..SPPLPPPP....PGGPPQ...............PAATRK................................
Bradi1g33970.1.p.bdi.32801481 24 SPEDR..SLPIPPPP....PPGG.................PPPTRK................................
Brast07G193800.1.p.bst.32871232 24 SPEDR..SLPLPPPP....PPGG.................PPPTRK................................
Migut.N02418.1.p.mgu.28925732 43 ............PLP....PR....................RGIRD.RERDS.....RERRD..DRD...........
Migut.D00714.1.p.mgu.28928758 51 ...........PPPP....PR....................RGIRD.RDRNS.....RERRD..DRD...........
Medtr8g043980.1.mtr.31070730 27 ............PPQ....PQSPPPTSAPDSDLPLPPP..QSSFRR.RDY.........RRDDRDRD...........
Tp57577_TGAC_v2_mRNA25888.tpr.35955570 ..............................................................................
Medtr3g006760.1.mtr.31058945 25 ............P................DDDLPPPP...NPNPRR.RD........FDRRD..DRD...........
Glyma.08G227300.1.p.gma.30540922 25 ............PPP....PP...................HYPPRR...........RDRRD..DRD...........
Phvul.011G187000.1.p.pvu.37156720 25 ............PPP....PPPTQPTPA.ADDLPPPPPPSHYPPRR...........RDRRD..DRD...........
Phvul.005G010800.1.p.pvu.37152760 25 ............PPP....P.....TSAAEED........PPPPRR...........RDRRE..DRE...........
Glyma.06G314900.1.p.gma.30549408 26 ............PPP....PPAS..SSAPADDLPPPPL..PPPPRR...........RDRRD..DRD...........
Solyc01g009090.2.1.sly.36140441 39 ............PPP....P.....................PSRRG.RDRDS.....RERRD..DRD...........
Thhalv10001919m.esa.20200705 24 ............P...SSLPQQEP.AQEQQE.........QQPPNR.RERDS.....RERRD..ERD...........
AT2G27100.1.ath.19641299 28 ............PPPSSSLPQQE...QEQDQ.........QQLPLR.RERDS.....RERRD..ERD...........
Q9ZVD0 28 ............PPPSSSLPQQE...QEQDQ.........QQLPLR.RERDS.....RERRD..ERD...........
Araha.4729s0005.1.p.aha.28848294 ..............................................................................
AL4G20790.t1.aly.35923270 29 ............PPPSSSLPQQE...QEQDQ.........QQPPQR.RERDS.....R......DRD...........
Bostr.29505s0043.1.p.adr.30675300 ..............................................................................
Carubv10022733m.cru.20903468 28 ............PPPSSSLPQQE...QEHEQ.........QQPPQR.RERDS.....RERRD..DKD...........
AL4G20820.t1.aly.35924196 28 ............PPPSSSLPQQE...QEQDQ.........EQPPQR.RERDS.....RERRD..ERD...........
Carubv10022734m.cru.20901991 28 ............PPPSSSLPQPE...QEQEQ.........EQPPQR.RERDS.....RERRD..DRD...........
Bostr.28794s0002.1.p.adr.30657126 ..............................................................................
Brara.D01611.1.p.bra.30620383 27 ............P.......QEH...EPQEQ.........QQPP....QRDS.....RERRD..DRD...........
Bol026461.bol.37343291 28 ............Q.......QDH...EPQEQ.........QQPP....QRDS.....RERRD..DRD...........
Brara.G01299.1.p.bra.30635851 28 ............PPPQHEQELQE...QQQEE.........KQP....RERDS.....RERRD..DID...........
Bol027793.bol.37343994 19 ............PPPQQEQEMQE...QQQEE.........KQP....RERDS.....RERRD..EID...........
mrna07673.1-v1.0-hybrid.fve.27266960 42 ............PPP............................SRR.RDRDE.....KER....DRD...........
Prupe.6G153800.1.p.ppe.32086540 41 ............PPP............................TRR.RDRDE.......GRD..DRD...........
GSVIVT01037454001.vvi.17842075 37 ............PPP....P......................SSRR...........RDRRD..DRE...........
orange1.1g003865m.csi.18122801 39 ............PPP............................ARR.RERDS.....RERRD..DRD...........
Gorai.004G227500.1.gra.26773739 38 ............PPP............................PRR.RDRDS.....RERRD................
Thecc1EG042011t1.tca.27426134 39 ............LPP............................PRR.RDRDS.....RERRD................
evm.model.supercontig_103.67.cpa.16404954 ..............................................................................
Potri.004G196400.1.ptr.26990875 43 ............PQP............................QRR.RERDS.....RERRE....D...........
SapurV1A.0615s0230.1.p.spu.31434857 45 ............LQP............................QRR.RERDS.....RERRE....D...........
29942.m000751.rco.16814310 38 ............PHP....HQ....................NNSRR.RDRDS.....RDR......D...........
Manes.11G111400.1.p.mes.32355670 36 ............PPPPSN.PQ....................NTSRR.RDRDS.....RDR......D...........
Manes.S021700.1.p.mes.32354619 36 ............PPP....PQ....................NISRR.RDRDS.....RDR......E...........
Eucgr.J00624.1.p.egr.32033287 46 ............PPP..........................AAPRR.RDRDS.....RER......E...........
GSVIVT01027348001.vvi.17834761 37 ............PPP....PPSQP.....QQ.........TLPVRR.RDRDS.....RERMD..DRD...........
Kalax.0225s0069.1.p.kla.32599097 37 ............PLQ....P.....................NQVSK.RPEEG.....RERRD..DRD...........
Kaladp0081s0182.1.p.kfe.35761984 37 ............QLQ....P.....................NQVSK.RPEDG.....RERRD..DRD...........
Kalax.0216s0065.1.p.kla.32585583 36 ............PLQ....T.....................NQASK.RHEDG.....RDRRG..DRD...........
DCAR_002130.dca.36055141 29 ............PPP............................MRR.RDRDS.....RERRD..NAE...........
Aqcoe2G007600.1.p.aco.33056562 36 ............PAS............................SRR.RDRDS.....RERRE..ERD...........
Bradi3g03840.1.p.bdi.32813176 45 DDDED..QLLLPPPP....PLGS.................SRPKRL...............D..DDD...........
Brast04G302900.1.p.bst.32866594 45 DDD.D..RLLLPPPP....PLGS.................SLPKRL...........VEEDD..DDD...........
Sevir.1G087000.1.p.svi.32667668 25 PDLAALLPLPLPPPA....PLGS.................SRPERL.VDRLEALA..RGRRRY...............
Pavir.J22990.1.p.pvi.30308347 29 EDVAL...LPLPPPP....PLGS.................SRPERL.ADRLEALA.PRTRRCY...............
Pahal.A00287.1.pha.32525941 26 EDVAL...LHLPPPP....PLGS.................S...RL.ADRLDALA.PRTRRCY...............
OAV97825.1 18 .....................G...................................RGGR.................
XP_003293253.1 14 ...............................................PNRDS.....SSDSDS.DRE...........
XP_005713144.1 ..............................................................................
XP_005702831.1 20 .....................GDS......................................................
XP_009857252.1 20 .....................GWG................RGDDRIKDERDTFY...RGRSPA.DRTRR.....RSRS
GES64941.1 ..............................................................................
KEY79445.1 11 .....................GRN...............REGDPYAARTQTDSY...RRRSPG.AQDRRRGTRGRSRS
XP_008869116.1 ..............................................................................
Q66I22 13 .................................................RDKFR...RERSD................
RXN03320.1 13 .................................................RDKFR...RERSD................
XP_015195966.1 2 ...........................................................SSE................
B1H1X4 13 .................................................RDKFR...RERSD................
NP_001362205.1 13 .................................................RDKFR...RERSD................
Q99MR6 13 .................................................RDKFR...RERSD................
Q9BXP5 13 .................................................RDKFR...RERSD................
XP_005549330.1 13 .................................................RDKFR...RERSD................
H2ZUZ4 13 .................................................RDKFR...RERSD................
XP_001748332.1 25 ............PKE.....I..........................HNHEYIFVIGQHHSGT...............
XP_002286542.1 33 .........................................................RGRSRS...............
XP_649316.1 8 ................................................................AKN...........
consensus>70 ..............................................................................
```

```
                                                     .
Pp3c25_7680V3.1.p.ppa.32980429             39  ....E....R.................................G....RR..........................
Pp3c16_11390V3.1.p.ppa.32986529            39  ....D....R.................................G....RR..........................
Sphfalx0012s0220.1.p.sfa.32627721          46  ....D....R....PP...RRE..........YNNE.......R....KT........................SQ
LOC_Os02g05610.1.osa.33135686              41  ....D....R....SP...RREL....................G....RS.PE..............DHGVPLPPP
LOC_Os08g40560.1.osa.33102829              17  .........................ST.....GGDR........................................
Pavir.Fb01956.1.p.pvi.30278011             13  ................................GSDR........................................
Pahal.F00690.1.pha.32488202                13  ............................GGG.GGDR........................................
Sevir.6G221600.1.p.svi.32643744            13  ........................GG.GGGG.GGDR........................................
Sobic.007G200600.1.p.sbi.37935124          13  ............................TTG.GGDR........................................
GRMZM2G132780_P02.zma.31013431             13  ............................TVG.RGDR........................................
Zm00008a003300_P01.zma.37201202            13  ............................TAG.RGDR........................................
Bradi3g40630.1.p.bdi.32820467              25  ........................GG.GGGG.SGDR........................................
Brast03G282200.1.p.bst.32857559            25  ........................SG.GGGG.SGDR........................................
Sobic.010G249100.1.p.sbi.37910925          47  .........R.................................S....RS.PP...................PPPP
GRMZM5G817439_P01.zma.30992522             47  .........R.................................S....RS.PP...................PPPP
Pavir.Da00165.1.p.pvi.30193584             47  .........R.................................S....RS.PPLP............PSSLPPPPP
Sevir.4G256700.1.p.svi.32645485            47  .........R.................................S....RS.PPPP............PSSLPPPPP
LOC_Os06g48530.1.osa.33147743              50  .........R.................................S....RS.PPPP............PPPPSLPPP
Bradi1g33970.1.p.bdi.32801481              47  .........R.................................S....RS.PPRP................SLPPP
Brast07G193800.1.p.bst.32871232            47  .........R.................................S....RS.PPRP................SLPPP
Migut.N02418.1.p.mgu.28925732              66  ...SD....R....PP...RR.........D.YYDR.......N....RS.PP......................P
Migut.D00714.1.p.mgu.28928758              75  ...FD....R....PP...RR.........E.YYNR.......N....RS.PP......................P
Medtr8g043980.1.mtr.31070730               66  ...FD....R....PP...PRR........E.YYDR.......N....NI.SP......................P
Tp57577_TGAC_v2_mRNA25888.tpr.35955570     14  ................................................RS..........................
Medtr3g006760.1.mtr.31058945               50  ...FD....R....RP...HRGGRGGRGGGG.YY..........................................
Glyma.08G227300.1.p.gma.30540922           44  ...FD....R....PP...NRR.......GD.YYDR.......N....AS.SPSS.........PRDRD....PLP
Phvul.011G187000.1.p.pvu.37156720          62  ...FD....R....PP...NRR.......GD.YYDR.......N....AS.........................P
Phvul.005G010800.1.p.pvu.37152760          50  ...LD....R....HP...NRS.......RD.YY..........................................
Glyma.06G314900.1.p.gma.30549408           60  ...FD....R....HP...NRS.......RD.YY..........................................
Solyc01g009090.2.1.sly.36140441            61  ...FD....R....RG...GR........GD.YYDR.......N....RS.PPHH....................P
Thhalv10001919m.esa.20200705               58  ...LE....R....PP...NRR............ER.......D....RS.PL......................P
AT2G27100.1.ath.19641299                   63  ...IE....RP...PP...NRR............ER.......D....RS.PL......................P
Q9ZVD0                                     63  ...IE....RP...PP...NRR............ER.......D....RS.PL......................P
Araha.4729s0005.1.p.aha.28848294               ............................................................................
AL4G20790.t1.aly.35923270                  60  ...IE....RP...PP...NRR............DR.......D....RS.PL......................P
Bostr.29505s0043.1.p.adr.30675300              ............................................................................
Carubv10022733m.cru.20903468               63  ...LE....R....PP...NRR............DR.......D....RS.PL......................P
AL4G20820.t1.aly.35924196                  63  ...IE....R....PP...NRR............DR.......D....RS.PL......................P
Carubv10022734m.cru.20901991               63  ...LE.............RR............DR.......D....RS.PL......................P
Bostr.28794s0002.1.p.adr.30657126              ............................................................................
Brara.D01611.1.p.bra.30620383              52  ...LE....R....PP...NRR.....................D....RS.PL.....................PP
Bol026461.bol.37343291                     53  ...LE....R....PP...NRC.....................D....RS.PL.....................PP
Brara.G01299.1.p.bra.30635851              60  ...LE....R....PA...NR..............R.......D....RS.PP......................P
Bol027793.bol.37343994                     51  ...LE....R....PP...NR..............R.......D....RS.PP......................P
mrna07673.1-v1.0-hybrid.fve.27266960       59  ...LD....R....PP...RR........PD.YYDRG......N....RS.PP......................P
Prupe.6G153800.1.p.ppe.32086540            58  ...LD....R....PP...SRR.......PD.YYDR.......N....RS.PP......................P
GSVIVT01037454001.vvi.17842075             53  ...LD....R....PP...NRR.......AD.YYDR.......N....RS.SP......................P
orange1.1g003865m.csi.18122801             58  ...LD....RP...PP...NRR......GPD.YYDR.......N....RS.PP......................P
Gorai.004G227500.1.gra.26773739            54  .........R....................D.YYDR.......N....RS.PP......................P
Thecc1EG042011t1.tca.27426134              55  .........R....................E.YYDR.......N....RS.PP......................P
evm.model.supercontig_103.67.cpa.16404954      ............................................................................
Potri.004G196400.1.ptr.26990875            60  ...FD....R....PL...NRR......GGD.YHDR.......N....RS.PP......................P
SapurV1A.0615s0230.1.p.spu.31434857        62  ...FD....R....PL...NRR......GGE.FHDR.......N....RS.PP......................P
29942.m000751.rco.16814310                 57  ...FD....R....PPPPPQNRR.......D.FYER.......NHNHHRS.PPL.....................P
Manes.11G111400.1.p.mes.32355670           58  ...LD....R....PP...NRR......GGD.YYDR.......N....RS.PL......................P
Manes.S021700.1.p.mes.32354619             55  ...LD....R....PP...NRR......GVD.YYDR.......N....RS.PP......................P
Eucgr.J00624.1.p.egr.32033287              63  ...LD....R....PP...NRR......GGD.YYDR.......N....QSSPP......................P
GSVIVT01027348001.vvi.17834761             66  ...GD....R....PR...NRRGS....GAD.HYDR.......N....LS.PP......................P
Kalax.0225s0069.1.p.kla.32599097           59  ...SD....R....PG...NRRA.....GGG.EYER.......N....RS.PP......................P
Kaladp0081s0182.1.p.kfe.35761984           59  ...SD....R....PG...NRRA.....GGG.EYER.......N....RS.PP......................P
Kalax.0216s0065.1.p.kla.32585583           58  ...LE....R....PG...NRRA.....GGG.DFER.......N....RS.PP......................P
DCAR_002130.dca.36055141                   48  ..TVD....R....AV...RR.........E.YNDR.......S....GN..........................
Aqcoe2G007600.1.p.aco.33056562             55  ...IDI..DRL..PPPIRGNRGGGGG.GGGD.FHDR.......N....RS.PP......................P
Bradi3g03840.1.p.bdi.32813176              72  .........RLLLPPP..................................PPLGSSRPKRLVEDENDDDRLLLPPP
Brast04G302900.1.p.bst.32866594            75  .........RLLLPPP..................................PPLGSSLPKRLVEDENDDDMLLFPPP
Sevir.1G087000.1.p.svi.32667668            64  .........R........RKRRSASP.GAGG...GRATPTP..T......PP..........RETEDAPALPLPPP
Pavir.J22990.1.p.pvi.30308347              66  .........R........RRRTSASP.GAGG..RARA......T......PP..........REPQDAPPLPLPPP
Pahal.A00287.1.pha.32525941                60  .........R........TRRRSASP.GAGG..RARA......T......PP..........REPQDAPPLPLPPP
OAV97825.1                                 23  ...FDDAGRRS...PPP.PADRWN....GGN...DR.......N....GTGPPGSNL...................
XP_003293253.1                             28  ...YD....R......................YRKR......RD....RY.SP.......................
XP_005713144.1                                 ............................................................................
XP_005702831.1                             23  ...MD.............................GGN...........SK..VASAEP....................LPPP
XP_009857252.1                             52  PAALD....RY...EP....RART...RDD.YPTR.......D....PR.DPRD....................P
GES64941.1                                  1  ..MID....RY...EP...SDRRQP...RDD.YYAT.......A....RE.HAG.....................
KEY79445.1                                 49  PAMID....RY...EP...SVRRSS...RDD.YYAS.......T....RE.NPF.....................
XP_008869116.1                             23  ...FDEQ..Q....PP...ANIYS....SGG...................PPSSYL...................P
Q66I22                                     23  ...YDRSRER........EDRRR......DD.WNDRRPSAREWD................................
RXN03320.1                                 23  ...YDRSRER........EDRRR......DD.WNDR........................................
XP_015195966.1                              5  ...AAVALEA........EAVET......SS.............................................
B1H1X4                                     23  ...YDRSRER........EERRR......DD.WSDR....EWD.................................
NP_001362205.1                             23  ...YDRSRER........EERRR......DD.WSDR....EWD.................................
Q99MR6                                     23  ...YDRSRER........DERRR.....GDD.WNDR....EWD.................................
Q9BXP5                                     23  ...YDRSRER........DERRR.....GDD.WNDR....EWD.................................
XP_005549330.1                             23  ...YDRSRER........DERRR.....GDD.WNDR....EWD.................................
H2ZUZ4                                     23  ...YDRSRER........EDRRR......DD.WNDR....EWD.................................
XP_001748332.1                             45  .TILDLILCQ......................HNNI.......SC..I............................
XP_002286542.1                             39  ...FDAA.......LP...EDHVGG...GGG.YNNN.......N................................
XP_649316.1                                11  ...VE.....................IGDVYTSD........................................
consensus>70                                   .........r..................................................................
```

```
Pp3c25_7680V3.1.p.ppa.32980429            44  ..........DGDRRDR..........................DRDY.......KR..RK.SGT.........PP...
Pp3c16_11390V3.1.p.ppa.32986529           44  ..........DGDRRDR..........................DRDY.......KR..RK.SGT.........PP...
Sphfalx0012s0220.1.p.sfa.32627721         62  ERLGGGGGGGGGERRER..........................ERDH.......KR..RK.SPT.........PP...
LOC_Os02g05610.1.osa.33135686             63  PPL.......GSSRPERLAS.DRPEEGASAAAQPCGGRSESPTARSM......WPR..RL.SPA.......SLPP...
LOC_Os08g40560.1.osa.33102829             23  ......................................................KRG.RS.SPS........LPP...
Pavir.Fb01956.1.p.pvi.30278011            17  ......................................................KRR.HA.SPV........LPP...
Pahal.F00690.1.pha.32488202               20  ......................................................KRR.HA.SPV........LPP...
Sevir.6G221600.1.p.svi.32643744           23  ......................................................KRR.HA.SPV........LPP...
Sobic.007G200600.1.p.sbi.37935124         20  ......................................................KRG.RASSPV........LPP...
GRMZM2G132780_P02.zma.31013431            20  ......................................................KRG.RA.SPV........LPP...
Zm00008a003300_P01.zma.37201202           20  ......................................................KRG.RA.SPV........LPP...
Bradi3g40630.1.p.bdi.32820467             35  ......................................................KRG.RA.SPV........LPP...
Brast03G282200.1.p.bst.32857559           35  ......................................................KRG.RA.SPV........LPP...
Sobic.010G249100.1.p.sbi.37910925         57  PPL.......GSSRPQR..........................YRDH..RGGGGGRG.GS.SPS.........PP...
GRMZM5G817439_P01.zma.30992522            57  PPL.......GSSRPQR..........................YRDH..R.SGGGRG.GS.SPS.........PP...
Pavir.Da00165.1.p.pvi.30193584            64  PPL.......GSSRPQR..........................YRDH..R..GGGRG.GS.SPS.........PP...
Sevir.4G256700.1.p.svi.32645485           64  PPL.......GSSRPQR..........................YRDH..R..GGGRG.GS.SPS.........PP...
LOC_Os06g48530.1.osa.33147743             67  PPL.......GSSRPER..........................YRDNHHRGGGGGRG.GG.SSS.........PP...
Bradi1g33970.1.p.bdi.32801481             60  PPL.......GSSRPER..........................YRDRDRDNNRGGRG.GG.STS.........PP...
Brast07G193800.1.p.bst.32871232           60  PPL.......GSSRPER..........................YRDRDRDNHRGGRG.GG.STS.........PP...
Migut.N02418.1.p.mgu.28925732             84  P...............R..........................ERDF......HKRG.RL.SPS........PPP...
Migut.D00714.1.p.mgu.28928758             93  PL............PPR..........................ERDY......QKRG.RP.SPS........PPP...
Medtr8g043980.1.mtr.31070730              85  ..............RDR..........................DRDF.......KR..RR.SPS.........PP...
Tp57577_TGAC_v2_mRNA25888.tpr.35955570        ..............................................................................
Medtr3g006760.1.mtr.31058945              69  ..............NNR..........................DRDI.......KR..RR.SPS..........P...
Glyma.08G227300.1.p.gma.30540922          73  PR.........DRDRDR..........................DSDL.......KR..RR.SPS..........P...
Phvul.011G187000.1.p.pvu.37156720         80  PR.........DRDRDR..........................DRDF.......KR..RR.SPS..........P...
Phvul.005G010800.1.p.pvu.37152760         62  ...............DR..........................DRDF.......KR..RR.SPS..........P...
Glyma.06G314900.1.p.gma.30549408          72  ...............DR..........................DRDF.......KR..RR.SPS..........P...
Solyc01g009090.2.1.sly.36140441           82  P..............GR..........................ERDY.......KRG.RP.SHS........PPP...
Thhalv10001919m.esa.20200705              74  PP.........................................RRDY.......KR..RP.SGS........PPP...
AT2G27100.1.ath.19641299                  80  PP.........................................RRDY.......KR..RP.SLS........PPP...
Q9ZVD0                                    80  PP.........................................RRDY.......KR..RP.SLS........PPP...
Araha.4729s0005.1.p.aha.28848294              ..............................................................................
AL4G20790.t1.aly.35923270                 77  PP.........................................RRDY.......KR..RP.SLS........PPP...
Bostr.29505s0043.1.p.adr.30675300             ..............................................................................
Carubv10022733m.cru.20903468              79  PP.........................................RRDY.......KR..RP.SMS........PPP...
AL4G20820.t1.aly.35924196                 79  PP.........................................RRDY.......KR..RP.SLS........PPP...
Carubv10022734m.cru.20901991              75  PP.........................................RRDD.......KR..RP.SLS........PPP...
Bostr.28794s0002.1.p.adr.30657126             ..............................................................................
Brara.D01611.1.p.bra.30620383             67  PP.........................................RRDY.......KR..RP.SGS........PPP...
Bol026461.bol.37343291                    68  PP.........................................RRDY.......KR..RP.SGS........PPP...
Brara.G01299.1.p.bra.30635851             74  PP.........................................RRDY.......KR..RP.SGS........PPP...
Bol027793.bol.37343994                    65  PP.........................................RRDY.......KR..RP.SGS........PPP...
mrna07673.1-v1.0-hybrid.fve.27266960      79  PR...........ERDR..........................DRDY.......KR..RG.SVS........PPP...
Prupe.6G153800.1.p.ppe.32086540           78  PPI.......RERERDR..........................ERGY.......KR..RS.SLS........PPP...
GSVIVT01037454001.vvi.17842075            73  ..............RER..........................DRDY.......KR..RS.SPS........PPP...
orange1.1g003865m.csi.18122801            80  PMP...........RER..........................DRDY.......KR..RS.TVS........PPP...
Gorai.004G227500.1.gra.26773739           66  PP.............TR..........................ERDY.......KR..RS.SIS........PPP...
Thecc1EG042011t1.tca.27426134             67  PP.............PR..........................ERDY.......KR..RS.SVS........PPP...
evm.model.supercontig_103.67.cpa.16404954     ..............................................................................
Potri.004G196400.1.ptr.26990875           81  PQ............RER..........................EKEY.......KR..RS.STS........PPP...
SapurV1A.0615s0230.1.p.spu.31434857       83  PQ............RER..........................EKEY.......KR..RS.STS........PPP...
29942.m000751.rco.16814310                85  P.............RER..........................EREY.......KR..RN.SLS........PPP...
Manes.11G111400.1.p.mes.32355670          79  ..............RER..........................EREY.......KR..RS.SLS........PPP...
Manes.S021700.1.p.mes.32354619            76  ..............RER..........................ERDY.......KR..RN.SLS........PPP...
Eucgr.J00624.1.p.egr.32033287             85  PPL...........RER..........................DRGY.......KR..RG.SPS........PPP...
GSVIVT01027348001.vvi.17834761            89  ..............RDR..........................ERDF.......KR..RR.SPS..........P...
Kalax.0225s0069.1.p.kla.32599097          81  PLV.......GG..MNR..........................ERES......SKR..RA.SPG........SPP...
Kaladp0081s0182.1.p.kfe.35761984          81  PPV.......GG..MNR..........................ERES......SKR..RA.SPG........SPP...
Kalax.0216s0065.1.p.kla.32585583          80  APA.......GG..MNR..........................ERES......YKR..RA.SPG........SPP...
DCAR_002130.dca.36055141                  64  ..............LGR..........................EREY......NKR..RA.SPG........SPP...
Aqcoe2G007600.1.p.aco.33056562            88  PP............RER..........................ERDY.......KR..RL.SPS.........PP...
Bradi3g03840.1.p.bdi.32813176            105  PPL.......RSPRPKRLVEDDRLEADS...............PRNN.....GTPRG.HGRSPENVYAPLLPAPPAGS
Brast04G302900.1.p.bst.32866594          108  PPL.......GSSRPKRLVE.YRLEGDS...............PRNN.....GTTRG.HGRPPENVHVPLLPAPPAGS
Sevir.1G087000.1.p.svi.32667668          101  PPL.......GSSRPELAVV.ARLEGDG...............ARP..AAAGSVGR.....SAS..........PGAG
Pavir.J22990.1.p.pvi.30308347            100  PPL.......GSSRPELAV..ARLEGGD...............ARPA.AAAGSMGR.....SAS..........PGAG
Pahal.A00287.1.pha.32525941               94  PPL.......GSSRPELAV..VQLEGDG...............APPA.AAAGYMRK.....SAS..........PGAG
OAV97825.1                                55  ................RS.........G...............SAEF.......GRKRRR.DDS.........PP...
XP_003293253.1                            41  ...........................................DR........YQRG.........................
XP_005713144.1                                ..............................................................................
XP_005702831.1                            40  P..................................................RRG....SSG......F...P...
XP_009857252.1                            80  RDL..........RDPR..........................DRDD.......QSR....RMR........SPP...
GES64941.1                                27  ................R..........................ERED.......RRR....PPS..........PT..
KEY79445.1                                77  ................R..........................ERED.......RRA....APS..........PT..
XP_008869116.1                            45  P..................................................DRS.RS...............P...
Q66I22                                    49  .........RGRERRSRG.........E...............YRDYD.......RG.........................
RXN03320.1                                    ..............................................................................
XP_015195966.1                                ..............................................................................
B1H1X4                                    44  .........RGRERRSRG.........E...............YRDYE......SRS.........................
NP_001362205.1                            44  .........RGRERRSRG.........E...............YRDYE......SRS.........................
Q99MR6                                    45  .........RGRERRSRG.........E...............YRDYD.......RN.........................
Q9BXP5                                    45  .........RGRERRSRG.........E...............YRDYD.......RN.........................
XP_005549330.1                            45  .........RGRERRSRG.........E...............YRDYD.......RN.........................
H2ZUZ4                                    44  .........RGRERRSRG.........E...............YRDYD.......RG.........................
XP_001748332.1                            61  ......................................................QRL....SPR......LDPDP...
XP_002286542.1                            59  ................RM.....................................RRG.RN.SIS..........PS..
XP_649316.1                                   ..............................................................................
consensus>70                                  .......................................................r.....s............p...
```

Sequence	Start	Alignment
		....
Pp3c25_7680V3.1.p.ppa.32980429	64	...ADGSYR....DR.R......HS.P............RRSS..........P........P..Y.....KR.LRDD
Pp3c16_11390V3.1.p.ppa.32986529	64	...AGGSYR....DR.R......HS.P............RRSS..........P........P..Y.....KR.RRDD
Sphfalx0012s0220.1.p.sfa.32627721	92	...A...YR....DR.R......HS.P.......PL...RR.S..........P........G..YN....KRSRRDD
LOC_Os02g05610.1.osa.33135686	114	........RG...GR.R.....SES.P......TPRSIWRRLSPSPPP...PLPP......LP..P.....KRPRLD.
LOC_Os08g40560.1.osa.33102829	34	..........................P.......PP......GPP........PQ......VP..HG...SKRHRRDE
Pavir.Fb01956.1.p.pvi.30278011	28	..........................P.......LP......GPP........PP......GP..H.....KRHRRED
Pahal.F00690.1.pha.32488202	31	..........................P.......PP......GPP........PP......GP..H.....KRHRREE
Sevir.6G221600.1.p.svi.32643744	34	..........................P.......PP......GPP........PP......GP..H.....KRHRREE
Sobic.007G200600.1.p.sbi.37935124	32	..........................P.......PP......GPP........PP......GP..H.....KR.RRED
GRMZM2G132780_P02.zma.31013431	31	..........................P.......PP......GPP........PP......GP..H.....KK.RREE
Zm00008a003300_P01.zma.37201202	31	..........................P.......PP......GPP........PP......GP..H.....KK.RREE
Bradi3g40630.1.p.bdi.32820467	46	..........................P.......PP......GPP........PP......AA..Q....NKRHRAE.
Brast03G282200.1.p.bst.32857559	46	..........................P.......PP......GPP........PP......AS..Q....NKRHRAE.
Sobic.010G249100.1.p.sbi.37910925	86	......PYRG...SR.R......HS.P.......PR...R..SPS........P........P..F.....KRSRRDD
GRMZM5G817439_P01.zma.30992522	85	......PYRG...SR.R......HS.P.......SR...R..SPS........P........P..F.....KRSRRDD
Pavir.Da00165.1.p.pvi.30193584	91	......PYRG...SR.R......HS.P.......PR...R..SPS........P........P..F.....KRSRRDD
Sevir.4G256700.1.p.svi.32645485	91	......PYRG...SR.R......HS.P.......PR...R..SPS........P........P..F.....KRSRRDD
LOC_Os06g48530.1.osa.33147743	98	......PYRS...GR.R......HS.P.......SR...R..SPS........P........P..F.....KRSRRDD
Bradi1g33970.1.p.bdi.32801481	91	......P......RR.R......HS.P.......SR...R..SPS........P........P..F.....KRSRRDD
Brast07G193800.1.p.bst.32871232	91	......P......RR.R......HS.P.......SR...R..SPS........P........P..F.....KRSRRDD
Migut.N02418.1.p.mgu.28925732	102	.....PSYR....DR.RG..GGPYS.P.......PP...RR.SPP.......IP........P..Y.....KR.RRDD
Migut.D00714.1.p.mgu.28928758	114	.....PPYR....DR.RG..GGQHS.P.......PG...RR.SPP.......FP........P..Y.....KR.RRDD
Medtr8g043980.1.mtr.31070730	101	......PYR....DR.R......HS.P.......PM...RR.SP.........PH.....YNN..Y.....KRSRR..
Tp57577_TGAC_v2_mRNA25888.tpr.35955570	16	......GY.....DH..Y............
Medtr3g006760.1.mtr.31058945	84	......NYR....DR.R......YS.P.......PPQQSRR.SP.........P........P..Y.....KRSR...
Glyma.08G227300.1.p.gma.30540922	93	......PHR....ER.R......HS.P.......P....RR.LP.........P........P..Y.....KRSR...
Phvul.011G187000.1.p.pvu.37156720	100	......PHR....DQ.R......YS.P.......AP...RR.SP.........P........P..Y.....KRSR...
Phvul.005G010800.1.p.pvu.37152760	76	......GYR....DR.R......YS.P.......QP.PSRR.SP.........P........S..Y.....KRSR...
Glyma.06G314900.1.p.gma.30549408	86	......GYR....DR.R......YS.P.......PP.PSRR.SP.........P........P..Y.....KRSR...
Solyc01g009090.2.1.sly.36140441	100	...PLPPYR....DR.RG....GYS.P.......PH...RR.SPP.......FP........P..Y.....KRSRRDD
Thhalv10001919m.esa.20200705	90	......PYR....DR.R......HS.P.......PM...RR.S..........P........P..H.....KRYRRDD
AT2G27100.1.ath.19641299	96	......PYR....DR.R......HS.P.......PQ...RR.S..........P........P..Q.....KRYRRDD
Q9ZVD0	96	......PYR....DR.R......HS.P.......PQ...RR.S..........P........P..Q.....KRYRRDD
Araha.4729s0005.1.p.aha.28848294	1	....D
AL4G20790.t1.aly.35923270	93	......PYR....DR.R......HS.P.......PQ...RR.S..........P........P..H.....KRYRRDD
Bostr.29505s0043.1.p.adr.30675300		....
Carubv10022733m.cru.20903468	95	......PYR....DR.R......HS.P.......PM...RR.S..........P........P..H.....KRYRRDD
AL4G20820.t1.aly.35924196	95	......PYR....DR.R......HS.P.......PQ...RR.S..........P........P..Q.....KRYRRDD
Carubv10022734m.cru.20901991	91	......PYR....DR.R......YSPP.......PQ...RR.S..........P........P..H.....KRYRRDD
Bostr.28794s0002.1.p.adr.30657126		....
Brara.D01611.1.p.bra.30620383	83	......PYR....DR.R......HS.P.......PM...RR.SP.........P........P..S.....KRYRRDD
Bol026461.bol.37343291	84	......PYR....DR.R......HS.P.......PM...RR.SP.........P........P..P.....KRYRRDD
Brara.G01299.1.p.bra.30635851	90	......PYRG...DR.R......HS.P.......PM...RR.SP.........P........P..P.....KRYRRDD
Bol027793.bol.37343994	81	......PYRG...DR.R......HS.P.......PM...RR.SP.........P........P..P.....KRYRRDD
mrna07673.1-v1.0-hybrid.fve.27266960	99	......PYR....DRGR......HS.P.......PR...RS.PP.........P........P..L.....KRSRRED
Prupe.6G153800.1.p.ppe.32086540	102	......PYR....DR.R......HS.P.......TR...RS.PP.........P........P..F.....KRSRRED
GSVIVT01037454001.vvi.17842075	90	......PYR....DR.R......HS.P.......PR...RS.P..........PL......QP..Y.....KRSRRDD
orange1.1g003865m.csi.18122801	100	...PPLPYR....DR.R......HS.P.......PP...RR.S..........P........P..Y.....KRSRRDD
Gorai.004G227500.1.gra.26773739	84	...PPLNYR....DR.R......HS.P.......PP...RR.S..........P........P..Y.....KRSRRED
Thecc1EG042011t1.tca.27426134	85	...PPLNYR....DR.R......HS.P.......PP...RR.S..........P........P..Y.....KRSRRED
evm.model.supercontig_103.67.cpa.16404954		....
Potri.004G196400.1.ptr.26990875	100	....PLPYR....DR.R......HS.P.......PP...RR.S..........P........P..Y.....KRSRRED
SapurV1A.0615s0230.1.p.spu.31434857	102	....PLPYR....DR.R......HS.P.......PP...RR.S..........P........P..Y.....KRSRRED
29942.m000751.rco.16814310	103	.....IPYR....DR.R......HS.P.......PA...RR.S..........P........P..Y.....KRSRRDD
Manes.11G111400.1.p.mes.32355670	96	.....VPYR....DR.R......HS.P.......PP...RR.S..........P........P..Y.....KRSRRED
Manes.S021700.1.p.mes.32354619	93	.....VPYR....DR.R......HS.P.......PP...RR.S..........P........P..Y.....KRSRRED
Eucgr.J00624.1.p.egr.32033287	105	......PYR....DR.R......RS.P.......P..................P........P..Y.....KRSRRED
GSVIVT01027348001.vvi.17834761	104	......PYR....DR.R......HS.P.......PR...RS.S..........P........PLHF.....KRSRRDD
Kalax.0225s0069.1.p.kla.32599097	104	......PYR.......R......HS.P.......G....RR.SP.........P........P..Y.....KRSRRDE
Kaladp0081s0182.1.p.kfe.35761984	104	......PYR.......R......HS.P.......G....RR.SP.........P........S..Y.....KRSRRDE
Kalax.0216s0065.1.p.kla.32585583	103	......PYR.......R......HS.P.......G....RR.SP.........P........P..Y.....KRSRRDE
DCAR_002130.dca.36055141	82	....PSVYR....DR.R.....NYS.P.......PP...RR.SP.........PG......QG..Y.....KRMRRDE
Aqcoe2G007600.1.p.aco.33056562	106	......PYR....DR.R......HS.P..........................P........P..F.....KRSRRDD
Bradi3g03840.1.p.bdi.32813176	155	SRPGPVVYRL..L...E.....SES.P.......PD...GR.SPN........P........................
Brast04G302900.1.p.bst.32866594	157	SRPEGVVYR...L...E.....SQS.P.......PD...GR.SPC........PGGGVLGEAPPSP.....KRSRSND
Sevir.1G087000.1.p.svi.32667668	139	A.GAGGGQR....RRWQ......RS.P.......TR...RS.SPS........P.......AP..P.....KRSRRGS
Pavir.J22990.1.p.pvi.30308347	138	A.GGGQRLQ....RR.W......RS.P.......KR...RS.SPS........P.......AP..P.....KRSRRGS
Pahal.A00287.1.pha.32525941	132	A.GGGGQRR....RR.R......RS.P.......TR...RS.SPS....................P.....KRSRRGS
OAV97825.1	73	.............SRDRYIPNYEHR.P.......AR...............HLP.......NP...............
XP_003293253.1	47	.............GR.......DHS.P.......ER...RRRSPS........P........SQGYR..GRGRNRGDD
XP_005713144.1		....
XP_005702831.1	49	.......RRGSSRFRSR.....GQRGPF.....TSS..HFRMAQQLVTTGKHGQ...........YIQSLGRRQHRD.
XP_009857252.1	100	...ANI.......DR........YV.P...........GQDVAPIQPLTN...P.......IP...............
GES64941.1	40	..VANI.......DR........YV.P...........GQDGGKRPIPTN...P.......LP...............
KEY79445.1	90	..VANI.......DR........YV.P...........GQESGKRPLPTN...P.......LP...............
XP_008869116.1	52	.......YRGGGRNRSR.....SRS.P.......PR...RR.SRS........P........P..F.....RGRRRS.
Q66I22	66	.............RRER......FS.P.......PR...HDMS..........P........Q.......QKRMRRD.
RXN03320.1		....
XP_015195966.1	19	.............LSLC......LS.PFVSCAVVPN...ALLS..........A........A.......SVSFR...
B1H1X4	62	.............RRER......FS.P.......QR...HDLS..........P........P.......QKRMRRD.
NP_001362205.1	62	.............RRER......FS.P.......QR...HDLS..........P........P.......QKRMRRD.
Q99MR6	62	.............RRER......FS.P.......PR...HELS..........P........P.......QKRMRRD.
Q9BXP5	62	.............RRER......FS.P.......PR...HELS..........P........P.......QKRMRRD.
XP_005549330.1	62	.............RRER......FS.P.......PR...HELS..........P........P.......QKRMRRD.
H2ZUZ4	61	.............RRER......FS.P.......PR...HELS..........P........P.......QKRMRRD.
XP_001748332.1	72	.............EMHRS....L...PYCS........SRMLST.........PVLE..SNAP...............
XP_002286542.1	71	..........................P.......EGR.GRK........................F.....SRDYHDD
XP_649316.1	21	..........................PYVV..FTSE..GKKLKTQIIDCT.LDPI.........W....NKK.....
consensus>70		..............r.........s.p..........................p........p........kr.rrd.

```
Pp3c25_7680V3.1.p.ppa.32980429                 89  ....EFD.GR..RGS..PG.......................D.DR..........RYGGFD.GV.SAYGRG.......
Pp3c16_11390V3.1.p.ppa.32986529                89  ....EFD.GR..RGS..PG.......................D.DR..........RYGGFD.GV.GPYGRG.......
Sphfalx0012s0220.1.p.sfa.32627721             117  ....EFEGGG..RRG..AAAGA...............V.AG.E.DNGRLTGFLGEGRP.GFD.GM.PPYDRT....GGM
LOC_Os02g05610.1.osa.33135686                 151  ........GR..RSP..PR.GG..................................RF.GFE.HE.RGRERS......M
LOC_Os08g40560.1.osa.33102829                  54  GGGGGFD.RR..RLG..PG.VG..............YD.NP.D.DR..........RY.GND.HG.G...........A
Pavir.Fb01956.1.p.pvi.30278011                 46  GGGGGFD.RR..RLG..PV.GVG.............GH.EQ.D.DR..........RY.GNG.HG.G...........V
Pahal.F00690.1.pha.32488202                    49  GGGGGFD.RR..RLG..PV.GGG.............GH.DQ.D.DR..........RY.GNG.HG.G...........I
Sevir.6G221600.1.p.svi.32643744                52  .GGGGFD.RR..RLG..PV.GGG.............GH.EQ.D.DR..........RY.GNG.HG.G...........V
Sobic.007G200600.1.p.sbi.37935124              49  .GGGGFD.RR..RLG..PV.GGG.............GH.DQ.D.DR..........RY.GNG.LG.G...........V
GRMZM2G132780_P02.zma.31013431                 48  .GGGGFD.RR..RLG..PV.GGG.............GH.EQ.D.DR..........RY.GNG.LG.G...........V
Zm00008a003300_P01.zma.37201202                48  .GGGGFD.RR..RLG..PV.GGG.............GH.EQ.D.DR..........RY.GNG.LG.G...........V
Bradi3g40630.1.p.bdi.32820467                  64  ..GGGFD.RR..RLG..SG.GG..................Q.D.DR..........RF.GND...G.............
Brast03G282200.1.p.bst.32857559                64  ..GGGFD.RR..RLG..SG.GG..................Q.D.DR..........RF.GND...G.............
Sobic.010G249100.1.p.sbi.37910925             112  ....GYD.RRGGRRS.PPR..................Y.GY.D.DR..........RR.GYD......HERG.......
GRMZM5G817439_P01.zma.30992522                111  ....GYD.RRGGRGS.PPR..................Y.GY.D.DR..........RR.GYD......HERG.......
Pavir.Da00165.1.p.pvi.30193584                117  ....GYD.RRGGRGS.PPR..................Y.GY.D.DR..........RR.GYD......HERG.......
Sevir.4G256700.1.p.svi.32645485               117  ....GYD.RRGGRGS.PPR..................Y.GY.D.DR..........RR.GYD......YERG.......
LOC_Os06g48530.1.osa.33147743                 124  ....GYD.RRGGRGSPPPR..................Y.GY.G.DR..........RY.GYD......HERG.......
Bradi1g33970.1.p.bdi.32801481                 114  ....GYD.RR.GRGS.PPR.................................................YDRG.......
Brast07G193800.1.p.bst.32871232               114  ....GYD.RR.GRGS.PPR.................................................YDRG.......
Migut.N02418.1.p.mgu.28925732                 133  ....GYD.GR..RGS..PR.GG...............H.GR.G.DR..........RF.GYD.YP.GGYERE......N
Migut.D00714.1.p.mgu.28928758                 145  ....GFD.GR..RGS..PR.GG...............YGGR.G.DR..........RF.GYD.HP.GGYERD......M
Medtr8g043980.1.mtr.31070730                  127  ............GGS..PR.GG...............Y.GP.D.D...........RS.EYD.NY.GGYERGGRGGYDR
Tp57577_TGAC_v2_mRNA25888.tpr.35955570         21  ...GGYD.....RGG...R.GG................Y.....DR........GGRG.GYD.RG.GRGGND......R
Medtr3g006760.1.mtr.31058945                  109  ............RGS..PH....................RP.N.D...........RF.GHD.HF.GGYERG.......
Glyma.08G227300.1.p.gma.30540922              114  ............RGS..PR.GG...............Y.GP.D.DS..........RH.GYD.YY.GGYERG......M
Phvul.011G187000.1.p.pvu.37156720             122  ............RGS..PR.GG...............Y.GP.D.D...........RH.GYN.YY.GGYERG......M
Phvul.005G010800.1.p.pvu.37152760             100  ............RGS..PR..G...............Y.GP.E.D...........RF.GYE.YF.GGYERG......V
Glyma.06G314900.1.p.gma.30549408              110  ............RGS..PR.GG..............GY.GP.D.D...........RF.GYD.YS.GGYERG......A
Solyc01g009090.2.1.sly.36140441               132  .....YD.GR..RGS..PR.GG...............F.GH.G.DR..........RY.GYD.HQ.GGYDRE......M
Thhalv10001919m.esa.20200705                  114  ...NGFD.GR..RGC..PR.GG...............Y.GP.P.DR..........RF.GYD.YG.GGYDRE......M
AT2G27100.1.ath.19641299                      120  ...NGYD.GR..RGS..PR.GG...............Y.GP.P.DR..........RF.GYD.HG.GGYDRE......M
Q9ZVD0                                        120  ...NGYD.GR..RGS..PR.GG...............Y.GP.P.DR..........RF.GYD.HG.GGYDRE......M
Araha.4729s0005.1.p.aha.28848294                2  ...NGYD.AR..RGS..PR.GG...............Y.GP.P.DR..........RF.GYD.HG.GGYDRE......M
AL4G20790.t1.aly.35923270                     117  ...NGYD.AR..RGS..PR.GG...............Y.GP.P.DR..........RF.GYD.HG.GGYDRE......M
Bostr.29505s0043.1.p.adr.30675300                  ...............................................................................
Carubv10022733m.cru.20903468                  119  ...NGYD.AR..RGS..PR.GG...............Y.GP.P.DR..........RF.GYD.YG.GGYDRE......M
AL4G20820.t1.aly.35924196                     119  ...NGYD.TR........R.GG...............Y.GP.P.DR..........RF.GYD.HG.GGYDRE......M
Carubv10022734m.cru.20901991                  116  ...NGFD.SR..RGG..NR.GG...............Y.GP.P.DR..........RF.GYD.YG.GGYDRE......M
Bostr.28794s0002.1.p.adr.30657126                  ...............................................................................
Brara.D01611.1.p.bra.30620383                 108  ...NGYD.GR..RGG..G..GG...............Y.GP.P.DR..........RF.GYE......NDRE......M
Bol026461.bol.37343291                        109  ...NGYD.GR..RG......GG...............Y.GP.P.DR..........RF.GYE......NDRE......M
Brara.G01299.1.p.bra.30635851                 116  ...NGYD.GR..RG......GG...............Y.GP.P.DR..........RF.GYD......YDRE......M
Bol027793.bol.37343994                        107  ...NGYD.AR..RG......GG...............Y.GP.PADR..........RF.GYD......YDRE......M
mrna07673.1-v1.0-hybrid.fve.27266960          125  ...GGYD.GR..RGS..PR.GG...............F.GPGG.DR..........RS.GYD.YA.SGYDRE......M
Prupe.6G153800.1.p.ppe.32086540               127  ...GGYE.GR..RGS..PR.GG...............F.GP.G.DR..........RF.GYD.Y..TGYDRE......M
GSVIVT01037454001.vvi.17842075                116  ...AGYD.GR..RGS..PR.GG...............F.GP.G.DR..........RF.GYD.YA.SGYERE......M
orange1.1g003865m.csi.18122801                127  ...GGYE.PR..RGS..PR.GG...............F.GP...DR..........RF.GYD.YT.GGYERE......M
Gorai.004G227500.1.gra.26773739               111  ...GGYE.GR..RGS..PR.GG...............F.GP.G.DR..........RF.GYD.YG.GGYDRE......M
Thecc1EG042011t1.tca.27426134                 112  ...GGYE.GR..RGS..PR.GG...............F.GP.G.DR..........RF.GYD.YG.GGYDRE......M
evm.model.supercontig_103.67.cpa.16404954          ...............................................................................
Potri.004G196400.1.ptr.26990875               126  ...GGFD.AR..RGS..PR.GG...............F.GG.G.DR..........RF.GYD.YG.GGYERE......M
SapurV1A.0615s0230.1.p.spu.31434857           128  ...GGYD.AR..RGS..PR.GG...............F.GG.G.DR..........RF.GYD.YG.GGYERE......M
29942.m000751.rco.16814310                    128  ...GGYD.GR..RGS..PR.GG...............F.GP.G.DR..........RF.GYD.YG.GGYERE......M
Manes.11G111400.1.p.mes.32355670              121  ...GGYE.GR..RGS..PR.GG...............F.GP.G.DR..........RF.GYD.YA.GGYERE......I
Manes.S021700.1.p.mes.32354619                118  ...GGYE.GR..RGS..PR.GG...............F.GP.G.DR..........RF.GYD.YA.GGYERE......M
Eucgr.J00624.1.p.egr.32033287                 125  ...GGYE.GR..RGS..PR.GG...............F.GP.G.DR..........RF.GYD.Y...GYDRE......M
GSVIVT01027348001.vvi.17834761                130  ...GGYD..R..RGS..PR.GG...............F.GP.D.DR..........RF.GYD.YI.GGYER.......M
Kalax.0225s0069.1.p.kla.32599097              126  ..AGGFD.GR..R.......GG...............F.GP.G.DR..........RF.GYD.HG.GGYDRD......M
Kaladp0081s0182.1.p.kfe.35761984              126  ..AGGFD.GR..R.......GG...............F.GP.G.DR..........RF.GYD.HG.RGYDRD......M
Kalax.0216s0065.1.p.kla.32585583              125  ..GGGFD.GR..R.......GG...............F.AP.G.DR..........RF.GYD.HG.GGYDRD......M
DCAR_002130.dca.36055141                      112  ........................................................GF.GYNQHL.NGNDRE......M
Aqcoe2G007600.1.p.aco.33056562                125  ....GYD.GR..RRS..PR.GG...............F.GP.D.DR..........RF.GYD.YA.GGYDRG.....GA
Bradi3g03840.1.p.bdi.32813176                      ...............................................................................
Brast04G302900.1.p.bst.32866594               199  .....WEKRR..RSP..PR.RSP................SPVR.DK..........RT.RRD.......DRD.....GA
Sevir.1G087000.1.p.svi.32667668               172  ....SWSKCR..RSE..GR.GS.......................................................RT
Pavir.J22990.1.p.pvi.30308347                 170  ....SWSKDR..PSE..GR.GS.........................................................
Pahal.A00287.1.pha.32525941                   161  ....SWSKGR..RSE..GR.GS.......................................................RT
OAV97825.1                                     93  ...EIFDDPY..R.....R.GGSA...............YR.D.DM.................FP.YSPDRGLPA...R
XP_003293253.1                                 76  ....NWD.NY..R...NNR.NG.................YP.D.DI........NGHQ.GHH.GH.HGHHSS...NSNN
XP_005713144.1                                     ...............................................................................
XP_005702831.1                                 96  .....WDESF..E.......GGS.................................RV....................A
XP_009857252.1                                123  ............................................DPIKL.....PYQV.GFS.YY.GEWWRANE.....
GES64941.1                                     64  ............................................NPLTL.....DFQV.GFN.WF.AEWWRAEQ.....
KEY79445.1                                    114  ............................................NPLNL.....DFQV.GFN.WF.AEWWRAEQ.....
XP_008869116.1                                 82  .....FE.....R.............................D.DR.............................FRGN
Q66I22                                         88  .....WD.DH..........GG......................DP.................YH.GGYDLG...Y.GG
RXN03320.1                                     41  ......D.DH..........GG......................DP.................YH.GGYDLG...Y.GG
XP_015195966.1                                 46  ......D.DH..........GG......................DP.................YHGGGYDLP...Y.GG
B1H1X4                                         84  .....WD.DH..........GS......................DP.................YH.SGYDLP...YSSS
NP_001362205.1                                 84  .....WD.DH..........GS......................DP.................YH.SGYDMP...YSSA
Q99MR6                                         84  .....WD.EH..........SS......................DP.................YH.SGYDMP...YAGG
Q9BXP5                                         84  .....WD.EH..........SS......................DP.................YH.SGYEMP...YAGG
XP_005549330.1                                 84  .....WD.EH..........SS......................DP.................YH.SGYEMP...YAGG
H2ZUZ4                                         83  .....WD.DH..........GG......................DP.................YH.AGYDMP...YGGG
XP_001748332.1                                 96  ............Q.............................N.E..............GQH.LQ.HVYPSARK....L
XP_002286542.1                                 86  ...DGYSNGH.HRGS.LGG.GGRHANFSHRRSRGESF.....DHDD..........RH.....HH.HRRGRS...HSRG
XP_649316.1                                    49  .....FDVKY..N.......IGEVVVF........EVF....DHD..................................
consensus>70                                       ......d.....r...............................d..............g...................
```

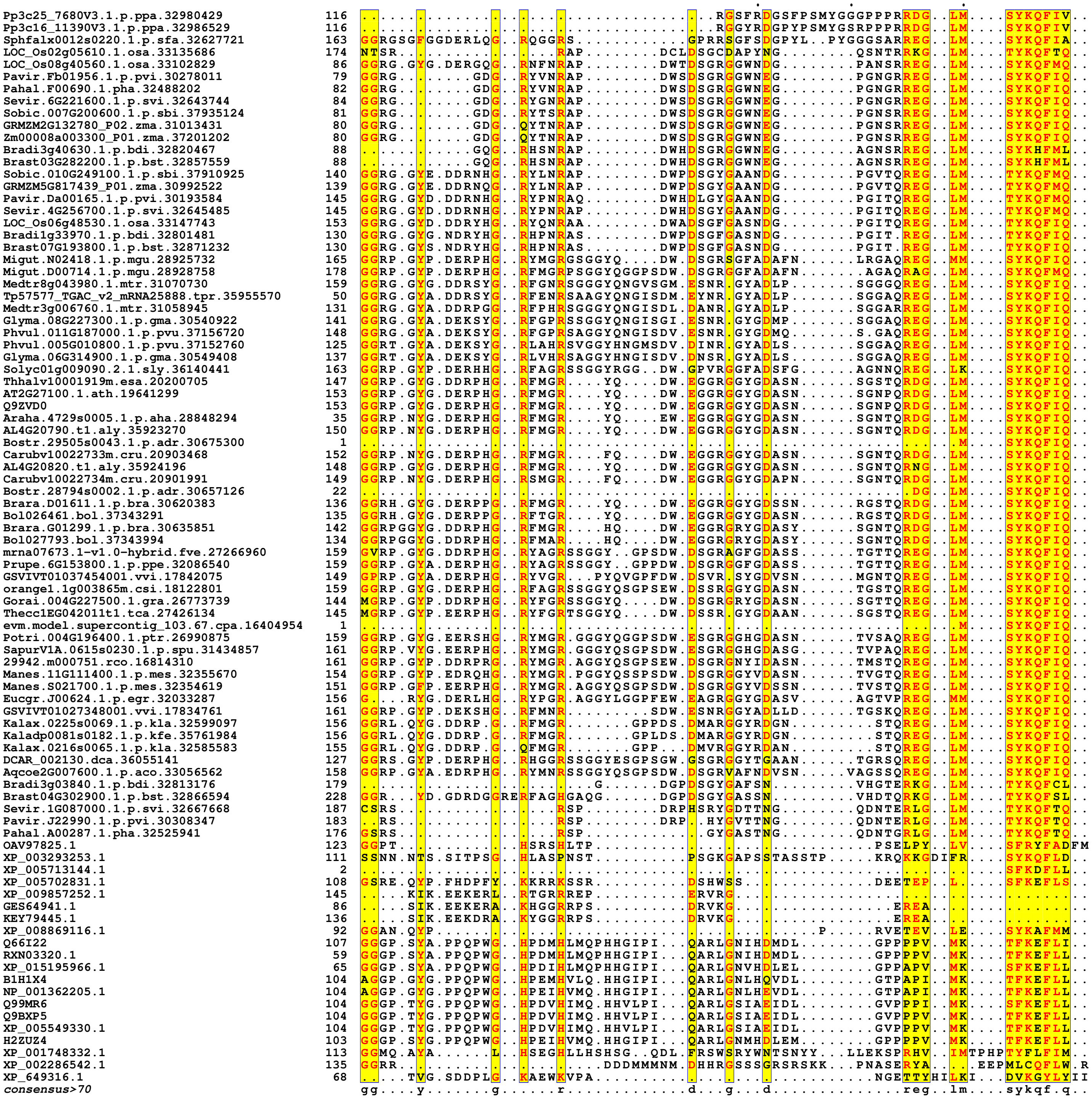

Pp3c25_7680V3.1.p.ppa.32980429 116 ......................................RGSFRDGSFPSMYGGGPPPRDG..LM....SYKQFIV..
Pp3c16_11390V3.1.p.ppa.32986529 116 ......................................RGGYRDGPYPSMYGSRPPPRDG..LM....SYKQFIV..
Sphfalx0012s0220.1.p.sfa.32627721 163 GGRGSGFGGDERLQG..RQGGRS............GPRRSGFSDGPYL.PYGGGSARREG..LM....SYKQFIA..
LOC_Os02g05610.1.osa.33135686 174 NTSR.................RAP........DCLDSGCDAPYNG.......QSNTRRKG..LM....TYKQFTQ..
LOC_Os08g40560.1.osa.33102829 86 GGRG.GYG.DERGQG..RNFNRAP........DWTDSGRGGWNDG.......PANSRREG..LM....SYKQFMQ..
Pavir.Fb01956.1.p.pvi.30278011 79 GGRG........GDG..RYVNRAP........DWSDSGRGGWNEG.......PGNGRREG..LM....SYKQFIQ..
Pahal.F00690.1.pha.32488202 82 GGRG........GDG..RYVNRAP........DWSDSGRGGWNEG.......PGNGRREG..LM....SYKQFIQ..
Sevir.6G221600.1.p.svi.32643744 84 GGRG........GDG..RYGNRAP........DWSDSGRGGWNEG.......PGNGRREG..LM....SYKQFIQ..
Sobic.007G200600.1.p.sbi.37935124 81 GGRG........GDG..RYTSRAP........DWSDSGRGGWNEG.......PGNSRREG..LM....SYKQFIQ..
GRMZM2G132780_P02.zma.31013431 80 GGRG........GDG..QYTNRAP........DWSDSGRGGWNEG.......PGNSRREG..LM....SYKQFIQ..
Zm00008a003300_P01.zma.37201202 80 GGRG........GDG..QYTNRAP........DWSDSGRGGWNEG.......PGNSRREG..LM....SYKQFIQ..
Bradi3g40630.1.p.bdi.32820467 88 ............GQG..RHSNRAP........DWHDSGRGGWNEG.......AGNSRREG..LM....SYKHFML..
Brast03G282200.1.p.bst.32857559 88 ............GQG..RHSNRAP........DWHDSGRGGWNEG.......AGNSRREG..LM....SYKHFML..
Sobic.010G249100.1.p.sbi.37910925 140 GGRG.GYE.DDRNHG..RYLNRAP........DWPDSGYGAANDG.......PGVTQREG..LM....TYKQFMQ..
GRMZM5G817439_P01.zma.30992522 139 GGRG.GYE.DDRNQG..RYLNRAP........DWPDSGYGAANDG.......PGVTQREG..LM....TYKQFMQ..
Pavir.Da00165.1.p.pvi.30193584 145 GGRG.GYD.DDRNHG..RYPNRAQ........DWHDLGYGAANDG.......PGITQREG..LM....TYKQFMQ..
Sevir.4G256700.1.p.svi.32645485 145 GGRG.GYD.DDRNHG..RYPNRAP........DWHDSGYGAANDG.......PGITQREG..LM....TYKQFMQ..
LOC_Os06g48530.1.osa.33147743 153 GGRG.GYD.DDRYHG..RYQNRAA........DWADSGFGASNDG.......PGITQREG..LM....TYKQFIQ..
Bradi1g33970.1.p.bdi.32801481 130 GGRG.GYG.NDRYHG..RHPNRAS........DWPDSGFGASNDG.......PGIT.REG..LM....TYKQFIQ..
Brast07G193800.1.p.bst.32871232 130 GGRG.GYS.NDRYHG..RHPNRAS........DWPDSGFGASNDG.......PGIT.REG..LM....TYKQFIQ..
Migut.N02418.1.p.mgu.28925732 165 GGRP.GYP.DERPHG..RYMGRGSGGYQ....DW.DSGRSGFADAFN......LRGAQREG..MM....SYKQFIQ..
Migut.D00714.1.p.mgu.28928758 178 GGRP.GYP.DERPHG..RFMGRPSGGYQGGPSDW.DSGRGGFADAFN.......AGAQRAG..LM....SYKQFMQ..
Medtr8g043980.1.mtr.31070730 159 GGRG.GYG.DDRSYG..RFGNRSGGGYQNGVSGM.ESNR.GYADLP......SGGGQREG..LM....SYKQFIQ..
Tp57577_TGAC_v2_mRNA25888.tpr.35955570 50 GGRG.GYA.DDRSYG..RFENRSAAGYQNGISDM.EYNR.GYADLP......SGGAQREG..LM....SYKQFIQ..
Medtr3g006760.1.mtr.31058945 131 GGRG.GYA.DDRPGG..RFPHRSGGGYQNGISDL.DANR.GYADLP......SGGARREG..LM....SYKQFIQ..
Glyma.08G227300.1.p.gma.30540922 141 GGRG.GYA.DEKSYG..RFGPRSSGGYQNGISGI.ESNR.GYGDMP......SGGAQREG..LM....SYKQFIQ..
Phvul.011G187000.1.p.pvu.37156720 148 GGRG.GYA.DEKSYG..RFGPRSAGGYQNGISDV.ESNR.GYGDMQ......S.GAQREG..LM....SYKQFIQ..
Phvul.005G010800.1.p.pvu.37152760 125 GGRT.GYA.DEKSYG..RLAHRSVGGYHNGMSDV.DINR.GYADLS......SGGAQREG..LM....SYKQFIQ..
Glyma.06G314900.1.p.gma.30549408 137 GGRT.GYA.DEKSYG..RLVHRSAGGYHNGISDV.DNSR.GYADLS......SGGAQREG..LM....SYKQFIQ..
Solyc01g009090.2.1.sly.36140441 163 GGRP.GYP.DERHHG..RFAGRSSGGYRGG..DW.GPVRGGFADSFG.....AGNNQREG..LK....SYKQFIQ..
Thhalv10001919m.esa.20200705 147 GGRP.GYG.DDRPHG..RFMGR....YQ....DW.EGGRGGYGDASN.....SGSTQRDG..LM....SYKQFIQ..
AT2G27100.1.ath.19641299 153 GGRP.GYG.DERPHG..RFMGR....YQ....DW.EGGRGGYGDASN.....SGNPQRDG..LM....SYKQFIQ..
Q9ZVD0 153 GGRP.GYG.DERPHG..RFMGR....YQ....DW.EGGRGGYGDASN.....SGNPQRDG..LM....SYKQFIQ..
Araha.4729s0005.1.p.aha.28848294 35 GGRP.NYG.DERPHG..RFMGR....YQ....EW.EGGRGGYGDASN.....SGNTQRDG..LM....SYKQFIQ..
AL4G20790.t1.aly.35923270 150 GGRP.NYG.DERPHG..RFMGR....YQ....DW.EGGRGGYGDASN.....SGNTQRDG..LM....SYKQFIQ..
Bostr.29505s0043.1.p.adr.30675300 1 ....M....SYKQFIQ..
Carubv10022733m.cru.20903468 152 GGRP.NYG.DERPHG..RFMGR....FQ....DW.EGGRGGYGDASN.....SGNTQRDG..LM....SYKQFIQ..
AL4G20820.t1.aly.35924196 148 GGRP.GYG.DERPHG..RFMGR....YQ....DW.EGGRGGYGDASN.....SGNTQRNG..LM....SYKQFIQ..
Carubv10022734m.cru.20901991 149 GGRP.NYG.DERPHG..RSMGR....FQ....DW.EGGRGGYGDAPN.....SGNTQRDG..LM....SYKQFIQ..
Bostr.28794s0002.1.p.adr.30657126 22 ....DG..LM....SYKQFIQ..
Brara.D01611.1.p.bra.30620383 136 GGRH.GYG.DERPPG..RFMGR....YQ....DW.EGGRGGYGDSSN.....RGSTQRDG..LM....SYKQFIQ..
Bol026461.bol.37343291 135 GGRH.GYG.DERPPG..RFTGR....YQ....DW.EGGRGGYGDASN.....RGSTQRDG..LM....SYKQFIQ..
Brara.G01299.1.p.bra.30635851 142 GGRPGGYG.DDRPHG..RFMGR....HQ....DW.EGGRGRYGDASY.....SGNTQRDG..LM....SYKQFIQ..
Bol027793.bol.37343994 134 GGRPGGYG.DDRPHG..RFMAR....HQ....DW.EGGRGRYGDASY.....SGNTQRDG..LM....SYKQFIQ..
mrna07673.1-v1.0-hybrid.fve.27266960 159 GVRP.GYG.DDRPHG..RYAGRSSGGY..GPSDW.DSGRAGFGDASS.....TGTTQREG..LM....SYKQFIQ..
Prupe.6G153800.1.p.ppe.32086540 159 GGRP.GYA.DERPHG..RYAGRSSGGY..GPPDW.DSGRGGFGDASS.....TGTTQREG..LM....SYKQFIQ..
GSVIVT01037454001.vvi.17842075 149 GPRP.GYA.DERPHG..RYVGR...PYQVGPFDW.DSVR.SYGDVSN.....MGNTQREG..LM....SYKQFIQ..
orange1.1g003865m.csi.18122801 159 GGRP.GYG.DERPHG..RFAGRSSGGYQSGPSEW.DSSRGGYGDASN.....TGSTQREG..LM....SYKQFIQ..
Gorai.004G227500.1.gra.26773739 144 MGRP.GYP.DDRPHG..RYFGRSSGGYQ....DW.DSGRGRYGDASN.....SGTTQREG..LM....SYKQFIQ..
Thecc1EG042011t1.tca.27426134 145 MGRP.GYP.EERPHG..RYFGRTSGGYQ....DW.DSSR.GYGDAAN.....SGSTQREG..LM....SYKQFIQ..
evm.model.supercontig_103.67.cpa.16404954 1 ....M....SYKQFIQ..
Potri.004G196400.1.ptr.26990875 159 GGRP.GYG.EERSHG..RYMGR.GGGYQGGPSDW.ESGRGGHGDASN.....TVSAQREG..LM....SYKQFIQ..
SapurV1A.0615s0230.1.p.spu.31434857 161 GGRP.VYG.EERPHG..RYMGR.GGGYQSGPSDW.ESGRGGHGDASG.....TVPAQREG..LM....SYKQFIQ..
29942.m000751.rco.16814310 161 GGRP.GYP.DDRPRG..RYMGR.AGGYQSGPSEW.DSGRGNYIDASN.....TMSTQREG..LM....SYKQFIQ..
Manes.11G111400.1.p.mes.32355670 154 GGRP.GYP.EDRQHG..RYMGR.PGGYQSSPSDW.DSGRGGYVDASN.....TVGTQREG..LM....SYKQFIQ..
Manes.S021700.1.p.mes.32354619 151 GGRP.GFP.EERPHG..RYMGR.AGGYQSGPSDW.DSGRGGYVDSSN.....TVSTQREG..LM....SYKQFIQ..
Eucgr.J00624.1.p.egr.32033287 156 G....RYG.DERLHG..RYPGR.AGGYLGGPFEW.EAGRGGYGDASV.....AGTVPREG..MM....SYKQFIQ..
GSVIVT01027348001.vvi.17834761 161 GGRP.GYP.DEKSHG..RFMNR.........SDW.ESNRGGYADLLD.....TGSKQREG..LM....SYKQFIQ..
Kalax.0225s0069.1.p.kla.32599097 156 GGRL.QYG.DDRP.G..RFMGR.......GPPDS.DMARGGYRDGN........STQREG..LM....SYKQFIQ..
Kaladp0081s0182.1.p.kfe.35761984 156 GGRL.QYG.DDRP.G..RFMGR.......GPLDS.DMARGGYRDGN........STQREG..LM....SYKQFIQ..
Kalax.0216s0065.1.p.kla.32585583 155 GGRL.QYG.DDRP.G..QFMGR.......GPP...DMVRGGYRDAN........STQREG..LM....SYKQFIQ..
DCAR_002130.dca.36055141 127 GGRS.GYP.DERPHG..RHGGRSSGGYESGPSGW.GSGRGGYTGAAN.....TGRSQREG..LM....SYKQFIQ..
Aqcoe2G007600.1.p.aco.33056562 158 GGRP.GYA.EDRPHG..RYMNRSSGGYQSGPSDW.DSGRVAFNDVSN....VAGSSQREG..LM....SYKQFIQ..
Bradi3g03840.1.p.bdi.32813176 179 ..........................G.....DGPDSGYGAFSN........VHGTERKG..LM....TYKQFCL..
Brast04G302900.1.p.bst.32866594 228 GGR...YD.GDRDGGRERFAGHGAQG......DGPDSGYGASSN........VHDTQRKG..LM....TYKQFSL..
Sevir.1G087000.1.p.svi.32667668 187 CSRS.................RSP........DRPHSRYGDTTNG.......QDNTERLG..LM....TYKQFTQ..
Pavir.J22990.1.p.pvi.30308347 183 ..RS.................RSP........DRP..HYGVTTNG.......QDNTERLG..LM....TYKQFTQ..
Pahal.A00287.1.pha.32525941 176 GSRS.................RSP.............GYGASTNG.......QDNTGRLG..LM....TYKQFTQ..
OAV97825.1 123 GGPT.............HSRSHLTP.............................PSELPY..LV....SFRYFADFM
XP_003293253.1 111 SSNN.NTS.SITPSG..HLASPNST..........PSGKGAPSSTASSTP....KRQKKGDIFR....SYKQFLD..
XP_005713144.1 2 ....SFKDFLL..
XP_005702831.1 108 GSRE.QYP.FHDPFY..KKRRKSSR..........DSHWSS.............DEETEP..L.....SFKEFLS..
XP_009857252.1 145 .....KIK.EEKERL..RTGRRREP...........ERVRG.....................................
GES64941.1 86 .....SIK.EEKERA..KHGGRRPS...........DRVKG................EREA.................
KEY79445.1 136 .....SIK.EEKDRA..KYGGRRPS...........DRVKG................EREA.................
XP_008869116.1 92 GGAN.QYP...P....RVETEV..LE....SYKAFMM..
Q66I22 107 GGGP.SYA.PPQPWG..HPDMHLMQPHHGIPI...QARLGNIHDMDL.......GPPPPV..MK....TFKEFLI..
RXN03320.1 59 GGGP.SYA.PPQPWG..HPDMHLMQPHHGIPI...QARLGNIHDMDL.......GPPPPV..MK....TFKEFLL..
XP_015195966.1 65 GGGP.SYA.PPQPWG..HPDIHLMQPHHGIPI...QARLGNVHDLEL.......GPPAPV..MK....SFKEFLL..
B1H1X4 104 AGGP.GYG.PPQPWG..HPEMHVLQ.HHGIPI...QARLGNLHQVDL.......GTPAPI..MK....TFKEFLL..
NP_001362205.1 104 AGGP.GYG.PPQPWG..HPEIHVMQ.HHGIPI...QARLGNLHEVDL.......GTPAPI..MK....TFKEFLL..
Q99MR6 104 GGGP.TYG.PPQPWG..HPDVHIMQ.HHVLPI...QARLGSIAEIDL.......GVPPPI..MK....SFKEFLL..
Q9BXP5 104 GGGP.TYG.PPQPWG..HPDVHIMQ.HHVLPI...QARLGSIAEIDL.......GVPPPV..MK....TFKEFLL..
XP_005549330.1 104 GGGP.TYG.PPQPWG..HPDVHIMQ.HHVLPI...QARLGSIAEIDL.......GVPPPV..MK....TFKEFLL..
H2ZUZ4 103 GGGP.SYG.PPQPWG..HPEIHVMQ.HHGIPI...QARLGNMHDLEM.......GPPPPV..MK....TFKEFLL..
XP_001748332.1 113 GGMQ.AYA......L..HSEGHLLHSHSG..QDL.FRSWSRYWNTSNYY...LLEKSPRHV..IMTPHPTYFLFIM..
XP_002286542.1 135 GGRR......................DDDMMMNM.DHHRGSSSGRSRSKK..PNASERYA.....EEPMLCQFLW.R
XP_649316.1 68 .....TVG.SDDPLG..KAEWKVPA.............................NGETTYHILKI...DVKGYLYII
consensus>70 gg....y.......g......r.............d...g...d.............reg..lm....sykqf.q..

```
Pp3c25_7680V3.1.p.ppa.32980429                 148  .ELEDDILPGEAERRYEEYKSEFITTHKRAFFEQHKDDEWLREKYDPARLEIVLVRRNENAKI.LSKELLAELQTGSL
Pp3c16_11390V3.1.p.ppa.32986529                148  .ELEDDILPGEAERRYEEYKSEFITTQKRAFFEQHKDDDWLREKYDPARLEIVLVRRNENAKI.LSKELLAELQAGSL
Sphfalx0012s0220.1.p.sfa.32627721              217  .ELEDDILPGEAERRYDEYKTEFITTQKRSFFEQHKDDDWLREKYDPARLEAVLVRRNENAKI.LAKELLAELQGSSL
LOC_Os02g05610.1.osa.33135686                  211  .KLEDDVSPGEAESRYQEYKTSYITSQKQDYFDHHKNEDRLKDMYHPTNLLSVIERRNELCKA.AAKNLILDLRSGTL
LOC_Os08g40560.1.osa.33102829                  136  .ELEDDVSPDEAQHRYEEYKSEYITTQKKAYFDLHKNDDWLKNKYHPTNLEIVMERRNELART.AANQFLQDLQNGSL
Pavir.Fb01956.1.p.pvi.30278011                 123  .ELEDDVSPVEAQSRYEEYKSEYITTQKKAYFDIHKDEDWLRNKYHPTNLEVVIERRNELART.TANEFFQDLQSGSL
Pahal.F00690.1.pha.32488202                    126  .ELEDDVSPVEAQSRYEEYKSEYITTQKKAYFDLHKDEDWLRNKYHPTNLEVVIERRNELART.TANEFFHDLPSGSL
Sevir.6G221600.1.p.svi.32643744                128  .ELEDDVSPVEAQSRYEEYKSEYITTQKKAYFDLHKDEDWLRNKYNPTNLEGVIERRNELART.TANEFFQDLQSGSL
Sobic.007G200600.1.p.sbi.37935124              125  .ELEDDVSPVEAQSRYEEYKSEYITTQKKAYFDLHKDEDWLRNKYHPTNLENVIERRNELART.TANDFFLQLQSGSL
GRMZM2G132780_P02.zma.31013431                 124  .ELEDDVSPVEAQSRYEGYKSEYITTQKKAYFDLHKNEDWLRNKYHPTNLENVIERRNELART.TANDFFLKLQSGDL
Zm00008a003300_P01.zma.37201202                124  .ELEDDVSPVEAQSRYEGYKSEYITTQKKAYFDLHKDEDWLRNKYHPTNLENVIERRNELART.TANDFFLKLQSGDL
Bradi3g40630.1.p.bdi.32820467                  128  .ELEDDVSPVEAQSRYEEYKSEYITTQKKAYFDLHKNEDWLRNKYHPTNLESVMERRNELARG.TANEFFQDLQSGRL
Brast03G282200.1.p.bst.32857559                128  .ELEDDVSPVEAQSRYEEYKSEYITTQKKAYFDLHKNEDWLRNKYHPTNLESVMERRNELARG.TANEFFQDLQSGRL
Sobic.010G249100.1.p.sbi.37910925              190  .VLEDDISPSEAERRYQEYRTEYITTQKRAYFDLHKNEDWLKDKYHPTNLLSVIERRNERCKV.IAKDFFLDLQNGTL
GRMZM5G817439_P01.zma.30992522                 189  .VLEDDISPSEAERRYQEYRTEYITTQKRAYFDLHKNEDWLKDKYHPTNLLSVIERRNERCKV.IAKDFFLDLQNGTL
Pavir.Da00165.1.p.pvi.30193584                 195  .VLEDDISPSEAERRYQEYKTEYITTQKRAYFDLHKNEEWLKDKYHPTNLLSVIERRNERCKV.MAKDFFLDLQNGTL
Sevir.4G256700.1.p.svi.32645485                195  .VLEDDISPSEAERRYQEYRTEYITTQKRAYFELHKNEDWLKDKYHPTNLVSVIERRNERCKV.MAKDFFLDLQNGTL
LOC_Os06g48530.1.osa.33147743                  203  .VLEDDISPAEAEKRYQEYRTEYITTQKRAYFDLNKNDDRLKDKYHPTNLSSVIDRRNDSCKA.TAKDFFHDLQNGTL
Bradi1g33970.1.p.bdi.32801481                  179  .VLEDDISPAEAESRYQEYKTEYITTQKRAYFDLHKNEDWLKDKYHPTNLLSVIERRNERCKV.VAKDFFLDLQNGTL
Brast07G193800.1.p.bst.32871232                179  .VLEDDISPAEAESRYQEYKTEYITTQKRAYFDLHKNEDWLKDKYHPTNLVSVIERRNERCKV.VAKDFFLDLQNGTL
Migut.N02418.1.p.mgu.28925732                  220  .ELEDDILPAEAERRYQEYKTEYISTQKTNFFNAHKDEEWLKDKYHPTNLLAVIERRNELAQK.LAKDFQADLQSGNL
Migut.D00714.1.p.mgu.28928758                  236  .ELEDDILPAEAERRYQEYKTEYISTQKRTYFDAHKDEQWLKDKYHPTNLLAVIDRRKELAKK.LAKDFQHDLLSGNL
Medtr8g043980.1.mtr.31070730                   216  .ELEDDILPTEAERRYQEYKSEYISTQKRAYFSAHKDEEWLKDKYHPTNLLTVIERRNESARQ.LAKDFLLDLQSGTL
Tp57577_TGAC_v2_mRNA25888.tpr.35955570         107  .ELEDDILPTEAERRYQEYRTEYISTQKRAYFNAHKDEEWLKDKYHPTNLLTVIERRNESARQ.LAKDFLLDLQSGTL
Medtr3g006760.1.mtr.31058945                   188  .ELEDDILPSEAERRYQEYKSEYITTQKRAYFNTHKDAEWLKDKYHPTNLLTVIERRNENARR.LAKEFLLDLQSGTL
Glyma.08G227300.1.p.gma.30540922               198  .ELEDDILPAEAERRYQEYKSEYISTQKRAYFNAHKDEEWLKDKYHPTNLVTVIERRNENARQ.LAKDFLLDLQSGTL
Phvul.011G187000.1.p.pvu.37156720              204  .ELEDDILPAEAERRYQEYKSEYISTQKRAYFNAHKDEEWLKDKYHPTNLLTVIERRNENARQ.LAKDFLLDLQSGTL
Phvul.005G010800.1.p.pvu.37152760              182  .ELEDDVLPAEAERRYQEYKSEYISTQKRAYFNAHKDEEWLKDKYHPTNLLTVIERRNENARR.LAKDFLLDLQSGTL
Glyma.06G314900.1.p.gma.30549408               194  .ELEDDVLPAEAERRYQEYKSEYISTQKRAYFNAHKDEEWLKDKYHPTNLLTVIERRNENARR.LAKDFLLDLQSGTL
Solyc01g009090.2.1.sly.36140441                220  .ELEDDILPAEAERRYQEYKAGYIEAQKRAYFNAHKDEEWLKDKYHPSNLISVIERRNELARK.SAKDFLLDLQSGTL
Thhalv10001919m.esa.20200705                   198  .ELEDDILPSEAERRYQEYKSEYITTQKRVFFNTHKEEEWLKDKYHPTNLLSVIERRNELARK.VAKDFLLDLQSGTL
AT2G27100.1.ath.19641299                       204  .ELEDDILPSEAERRYQEYKSEYITTQKRAFFNTHKEEDWLKNKYHPTNLLSVIERRNDLAQK.VAKDFLLDLQSGTL
Q9ZVD0                                         204  .ELEDDILPSEAERRYQEYKSEYITTQKRAFFNTHKEEDWLKNKYHPTNLLSVIERRNDLAQK.VAKDFLLDLQSGTL
Araha.4729s0005.1.p.aha.28848294                86  .ELEDDILPSEAERRYQEYKSEYITTQKRAFFNTHKEEDWLKNKYHPTNLLSVIERRNELAQK.VAKDFLLDLQSGTL
AL4G20790.t1.aly.35923270                      201  .ELEDDILPSEAERRYQEYKSEYITTQKRAFFNTHKEEDWLKNKYHPTNLLSVIERRNDLAQK.VAKDFLLDLQSGTL
Bostr.29505s0043.1.p.adr.30675300                9  .ELEDDILPSEADRRYQEYKSEYITTQKRAFFNTHKEEEWLKNKYHPTNLLSVIERRNDLARK.VAKDFLLDLQSGEL
Carubv10022733m.cru.20903468                   203  .ELEDDILPSEAERRYQEYKSEYITTQKRAFFNTHKEEEWLKNKYHPTNLLSVIERRNDLARK.VAKDFLLDLQGGAL
AL4G20820.t1.aly.35924196                      199  .ELEDDILPSEAERRYQEYKSEYITTQKRVFFNSHKEEEWLKNKYHPTNLLSVIERRNELARK.VAKDFLLDLQSGAI
Carubv10022734m.cru.20901991                   200  .ELEDDILPSEAERRYQEYKSEYITTQKRVFFNAHKEEDWLKNKYHPTNLLSVIEGRNELARK.VAKDFLLDLQSGAI
Bostr.28794s0002.1.p.adr.30657126               33  .ELEDDILPSEAERRYQEYKSEYITTQKHVFFNSHKEEDWLKNKYHPTNLLSVIERRNELART.VAKDFLLDLQSGAI
Brara.D01611.1.p.bra.30620383                  187  .ELEDDILPSEAERRYQEYKSEYITTQKRVYFNTHKEEEWLKDKYHPTNLLTVIEKRNEHARK.LAKDFSLDLQSANL
Bol026461.bol.37343291                         186  .ELEDDILPSEAERRYQEYKSEYITTQKRVYFNTHKEEEWLKDKYHPTNLLTVIEKRNEHARK.LAKDFLLDLQSGNL
Brara.G01299.1.p.bra.30635851                  194  .ELEDDILPSEAERRYQEYKSEYITTQKRVYFNTHKEEEWLKDKYHPTNLLTVIEKRNELAQK.VAKDFILSLQSGNL
Bol027793.bol.37343994                         186  .ELEDDILPSEAERRYQEYKSEYITTQKRVYFNTHKEEEWLKDKYHPTNLLTVIERRNELAQK.VAKDFILSLQSGNL
mrna07673.1-v1.0-hybrid.fve.27266960           216  .ELEDDILPAEAERRYQEYKAEYISTQKRAYFNAHKDEEW...............RNEISQK.TAKEFLLELQSGSL
Prupe.6G153800.1.p.ppe.32086540                216  .ELEDDILPAEAERRYQEYRTEYITTQKRAYFNAHKDEDWLKDKYHPTNLLTVIERRNEHARK.VAKDFLLELQSGTL
GSVIVT01037454001.vvi.17842075                 204  .ELEDDILPAEAERRYQEYKSEYISTQKRSFFDSHKDEEWLKDKYHPTNLLSVIERRNEVARK.TAKDFLLDLQSGTL
orange1.1g003865m.csi.18122801                 218  .ELEDDILPAEAERRYQEYKSEYISTQKRVFFDAHKDEEWLKDKYHPTNLVAVIERRNDLARK.VAKDFLLDLQSGTL
Gorai.004G227500.1.gra.26773739                199  .ELEDDILPAEAERRYQEYKSEYIATQKRAFFDAHKDEEWLRDKYHPTNLVTVIERRNELARK.VAKDFFLDLQSGTL
Thecc1EG042011t1.tca.27426134                  199  .ELEDDILPAEAERRYQEYKSEYISTQKRAFFDAHKDEEWLRDKYHPTNLVTVIERRNELARK.VAKDFLLDLQSGTL
evm.model.supercontig_103.67.cpa.16404954        9  .ELEDDILPSEAERRYQEYKSEYISTQKRTFFDAHKDEEWLKDKYHPTNLLAVIERRNELARK.VAKDFLLDLQSGTL
Potri.004G196400.1.ptr.26990875                217  .ELEDDILPSEAERRYQEYKSEYISTQKRVFFEAHKDEEWLKDKYHPTNLVAVIERRNELARK.VAKDFLLDLQSGTL
SapurV1A.0615s0230.1.p.spu.31434857            219  .ELEDDILPSEAERRYQEYKSEYISTQKRVFFEVHKDEEWLKDKYHPTNLVAVIERRNELARK.IAKDFLLDLQSGTL
29942.m000751.rco.16814310                     219  .ELEDDVLPAEAERRYQEYKSEYISTQKRVFFEAHKDEEWLKDKYHPTNLVAVIERRNELARK.AAKDFLLDLQSGTL
Manes.11G111400.1.p.mes.32355670               212  .ELEDDILPAEAERRYQEYKSEYISTQKRVFFESHKDEEWLRDKYHPTNLVAVIERRNELARK.VAKDFLLDLQSGTL
Manes.S021700.1.p.mes.32354619                 209  .ELEDDILPAEAERRYQEYKSEYISTQKRVFFEAHKDEEWLRDKYHPTNLVSVVERRNELARK.VAKDFLLDLQSGTL
Eucgr.J00624.1.p.egr.32033287                  211  .ELEDDILPAEAERRYQEYKSEYISTQKRAYFDAHKDEEWLKDKYHPTNLVAVIERRNELARR.VAKDFLLDLQSGTL
GSVIVT01027348001.vvi.17834761                 211  .ELEDDILPAEAERRYQEYRSEYISTQKRAFFDAHKDEEWLKDKYHPTNLLAVIERRNENARR.MAKDFLLDLQSGTL
Kalax.0225s0069.1.p.kla.32599097               204  .ELEDDILPSEAERRYQEYKSEYIATQKRTFFAAHKDEEWLKDKYHPGNLLAVIERRNELARK.IAKDFLLDLQSGAL
Kaladp0081s0182.1.p.kfe.35761984               204  .ELEDDILPSEAERRYQEYKSEYIATQKRTFFAAHKDEEWLKDKYHPGNLLAVIERRNELARK.IAKDFLLDLQSGAL
Kalax.0216s0065.1.p.kla.32585583               201  .ELEDDILPSEAERRYQEYKSEYIATQKRTFFAAHKDEEWLKDKYHPGNLLAVIERRNELAHK.VAKDFLLDLQSGTL
DCAR_002130.dca.36055141                       186  .ELEDDILPAEAERRYQEYKSEYITTQKRAYFNAHKHEEWLKDKYHPTNLLAVIDRRNEHARK.IAKDFLLELQSGTV
Aqcoe2G007600.1.p.aco.33056562                 218  .ELEDDILPAEAERRYQEYRSEYISTQKRAYFDAHKDEDWLRDKYHPTNLLTVIERRNEHARK.IAKDFLLDLQSGTL
Bradi3g03840.1.p.bdi.32813176                  209  .FLEDDVSPGEAGSRYQEYKTAYITTQKRAYFDLHKDEIRLKEKYHPTNLLSVIERRNGFCKA.AAKDLILDLRTGVL
Brast04G302900.1.p.bst.32866594                279  .LLEDDVSPGEAGSRYQEYMTAYITTQKRAYFDLHKDEIWLKEMYHPTNLLSVIERRNGFCNV.AAKNLILDLRNGIL
Sevir.1G087000.1.p.svi.32667668                224  .VLEDDVSPSQAGGRYQEYRTEYITTQKRAYFDLNKNEDWLKDMYHPTKLLSVIERRNDFCKT.AAKNLIHDLQNGTL
Pavir.J22990.1.p.pvi.30308347                  216  .VLEDDVSPAQAGARYKEYRTEYIATQKRAYFDLNKNEDWLKDMYHPTKLLSVIERRNEFCKT.VAKNLILDLRNGTL
Pahal.A00287.1.pha.32525941                    208  .VLEDDVSPAQAGSRYQEYRTEYIATQKRAYFDFNKNEDWLKDMYHPTKLLSVIERRNEFCKT.VAKNLILDLRNGTL
OAV97825.1                                     152  RSTSPHDDKEKLAEQWKRYRLDHTRKQLVAFFEENRNKAWFREKYQPGPEFEERQRLRKKGREGKVEGFISRLEKGDL
XP_003293253.1                                 164  .YQDDQITAADADKKYEEYKVEYSKRQSRVFFKEHQNEEWFKEKYDPVFLAKKRKEKVEKSKS.LVSTFSLNLEGDKL
XP_005713144.1                                   9  .KQPDNISSHAAQEAYDEYVKSFAVRKPNEFFEQHKDEEWFRERYDPEYVSKRIVRIREEVQE.RAKVYRDLWERGGS
XP_005702831.1                                 149  .REDNSITPEQAQEAYKKYKKEFYKRKPKYFFEKHMNEEWFCEKYHPSKVLERVNRIRQSCKE.QVERFKEMLGKESI
XP_009857252.1                                 167  .PEEREKEKAKIQAAYDAYKEELQAKMAQTFVKQHKDEQWFRERYVPEIRDGFRKQLNEFRRG.AYTQWEQDLETGTF
GES64941.1                                     112  .REDREREREAQIQAAYDAYKVDLQIKMARTFVQQHRNEEWFKERYVPEVRDPLRRKLMDFRAG.AYQQWERDIENGLF
KEY79445.1                                     162  .REDREREREAQIQAAYDAYKVDLQIKMSRTFVQHHRNEEWFKERYVPEVRDPLRRRLMDFRVG.AYQQWERDLEGGLF
XP_008869116.1                                 115  .RQEDNVAPEVCLQRYEDYKKRVLVKSSRSFFDQHKMEEWLQERYSPAIRHRHQQKKLAKKRV.DVKAFAERVKTTTL
Q66I22                                         162  .SLDDSVDETESVKRYNEYKIDFRRQQMQDFFLAHKDEEWFRSKYHPDEAGRRKAEAHSALQN.RLGVYMYLMDNNWF
RXN03320.1                                     114  .SLDDSVDETESVKRYNEYKIDFRRQQMQDFFLAHKDEEWFRSKYHPDEAGRRKAEAHSALQN.RLNVYMYLMDNNSF
XP_015195966.1                                 120  .SLDDSVDETEAVKRYNDYKLDFRRQQMQDFFLAHKDEEWFRSKYHPDEVGRRKAEAHSALQN.RLGVFMYLLENGWL
B1H1X4                                         158  .SLEDTVDETEAVKRYNDYKIDFRRQQMQEFFLAHKDEEWFRSKYHPDEVGKHKQESQASLRN.RLSAFMFLMEKSWL
NP_001362205.1                                 158  .SLEDSVDETEAVKRYNDYKIDFRRQQMQEFFLAHKDEEWFRSKYHPDEVGKHKQESQAALRN.RLSAFMYLMENSWL
Q99MR6                                         158  .SLDDSVDETEAVKRYNDYKLDFRRQQMQDFFLAHKDEEWFRSKYHPDEVGKRRQEARGALQN.RLKVFLSLMESGWF
Q9BXP5                                         158  .SLDDSVDETEAVKRYNDYKLDFRRQQMQDFFLAHKDEEWFRSKYHPDEVGKRRQEARGALQN.RLRVFLSLMETGWF
XP_005549330.1                                 158  .SLDDSVDETEAVKRYNDYKLDFRRQQMQDFFLAHKDEEWFRSKYHPDEVGKRRQEARGGLQN.RLRVFLSLMETGWF
H2ZUZ4                                         157  .SLDDSVDETEAVKRYNEYKLEFRRQQMQDFFLAHKDEEWFRSKYHPDEVGKRKQESHSALRN.RLNVFTYLMDNTWF
XP_001748332.1                                 172  ......CSHYNWRRRWRALRTTCGREILLHWLQVH...EYAADHLHLHNIMGILNRSKSMAQA.YTNIFFDFLDSGTD
XP_002286542.1                                 181  KELEDEESDQEATQAYTIYNHKYCLNYIRTFFNSHLDDPWFRHHLSPLVKYRQRTRSNVEANE.IKKEILYSLEESNL
XP_649316.1                                    105  ........AKCISGGCQITRQMIDPNAKMLLELKFNKLYLLDSFY...........KISKKVSS.KGYQFTVSVKSNST
consensus>70                                        ..leddi.p.eae.ryqeyk.eyi.tqk..ffd.hkdeewl.dkyhp.nl..vierrne.....akdf..dlq.g.l
```

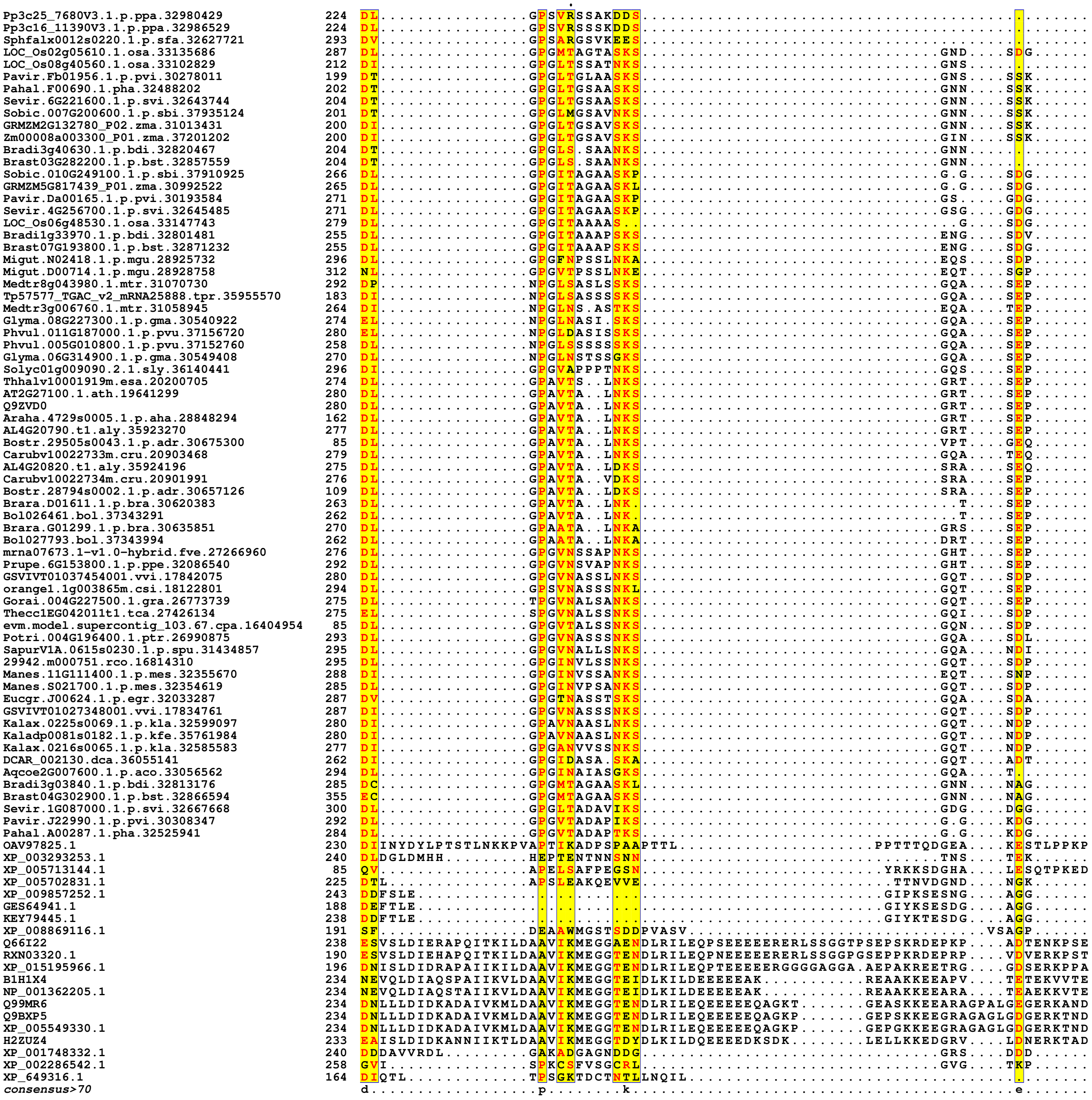

```
Pp3c25_7680V3.1.p.ppa.32980429              238 .............................GDD........AD.........GSSKRKRPPQEHVKAQE.I...N....
Pp3c16_11390V3.1.p.ppa.32986529             238 .............................GDD........AD.........GNNKRRKSSQEHVKAQE.T...I....
Sphfalx0012s0220.1.p.sfa.32627721           307 .............................EDD........GD.........MGAKGKRQSRSTKKSQE.I...N....
LOC_Os02g05610.1.osa.33135686               307 ...........................IPADD........ED.........YHNKRRRHHRGPLEETELV...S....
LOC_Os08g40560.1.osa.33102829               229 .............................VDD........TD.........ANGKKGKLGKS...QDDLY...Y....
Pavir.Fb01956.1.p.pvi.30278011              219 ...........................NL..V........DS.........SNGKKGKHGNG...PEDLY...Y....
Pahal.F00690.1.pha.32488202                 222 ...........................NL..V........DS.........SDGKKGKHGNG...PEDLY...Y....
Sevir.6G221600.1.p.svi.32643744             224 ...........................NL...........VD.........ADGKKGKLGKG...TDDLY...Y....
Sobic.007G200600.1.p.sbi.37935124           221 ...........................NL.DA........VE.........GDGKKGKLGKG...PDDSY...Y....
GRMZM2G132780_P02.zma.31013431              220 ...........................NL.DA........VE.........GDGKKGKHGKG...SDDSY...Y....
Zm00008a003300_P01.zma.37201202             220 ...........................NL.DA........VE.........GDGKKGKHGKG...SDDSY...Y....
Bradi3g40630.1.p.bdi.32820467               220 ...............................D........LN.........ANGKKGKH......PDDLY...Y....
Brast03G282200.1.p.bst.32857559             220 ...............................D........LN.........ANGKKGKH......PDDLY...Y....
Sobic.010G249100.1.p.sbi.37910925           285 ...........................NSEDD........MD..........NDKRRKHGKDSSKATDSL...S....
GRMZM5G817439_P01.zma.30992522              284 ...........................NSEDD........MD..........NDKRRKHGKDSSKATDSL...S....
Pavir.Da00165.1.p.pvi.30193584              290 ...........................NSDDD........MD..........SDKRRKHGKDSSKGADSL...S....
Sevir.4G256700.1.p.svi.32645485             291 ...........................NSDDD........MD..........GDKRRKQGKDSSKGTDSL...S....
LOC_Os06g48530.1.osa.33147743               295 ...........................NSDDD........GD..........SDKRRKHGRGSSKETDPL...S....
Bradi1g33970.1.p.bdi.32801481               275 ...........................NSEDD........GD..........GDKRRKHIRGSSKEKDPL...S....
Brast07G193800.1.p.bst.32871232             275 ...........................NSEDD........GD..........GDKRRKHIRDSSKEKDPL...S....
Migut.N02418.1.p.mgu.28925732               316 ...........................KSDDE........EN.........AGGKRKQPGRSGAKDSD.H...S....
Migut.D00714.1.p.mgu.28928758               332 ...........................HLEDE........GD.........AGGKRKRNVRGGTEESD.L...S....
Medtr8g043980.1.mtr.31070730                312 ...........................NSEEE........AE...........DQRRRDGWGSNKKSD.F...S....
Tp57577_TGAC_v2_mRNA25888.tpr.35955570      203 ...........................NSDEE........AD...........GKRRRHGRGSNKQSD.F...S....
Medtr3g006760.1.mtr.31058945                283 ...........................NSEEE........TD.........AGGKRRRHGRGSNKDND.F...S....
Glyma.08G227300.1.p.gma.30540922            293 ...........................NSEEE........AD.........TGGKRRRQGRGSNKEND.F...S....
Phvul.011G187000.1.p.pvu.37156720           300 ...........................NSEEE........GD.........TGGKRRRHGRGSNKDND.F...S....
Phvul.005G010800.1.p.pvu.37152760           278 ...........................NSEEE........TD.........GGIKRRRHGRGPNKDND.F...S....
Glyma.06G314900.1.p.gma.30549408            290 ...........................NSEEE........TD...........GKRRRHVRGPNKDND.F...S....
Solyc01g009090.2.1.sly.36140441             316 ...........................NSDEE........AD.........ENGKRRRHGRGHNKDPDLL...L....
Thhalv10001919m.esa.20200705                292 ...........................NSEDE........AA.........NGGKRKRPGKGGAKEIE.H...S....
AT2G27100.1.ath.19641299                    298 ...........................NSEDE........AA.........GVGKRKRHGMGGAKENELL...S....
Q9ZVD0                                      298 ...........................NSEDE........AA.........GVGKRKRHGMGGAKENELL...S....
Araha.4729s0005.1.p.aha.28848294            180 ...........................NSEDE........AA.........GGGKRKRHGMGGAKETELL...S....
AL4G20790.t1.aly.35923270                   295 ...........................NSEDE........AA.........GGGKRKRHGMGGSKETELL...S....
Bostr.29505s0043.1.p.adr.30675300           103 ...........................NSEDE........AA..........GGKRKRHDRVGAKETDLL...S....
Carubv10022733m.cru.20903468                297 ...........................NSDDE........AA..........GGKRKRHDRAN..EAD.F...S....
AL4G20820.t1.aly.35924196                   293 ...........................ISDDD........AA.........GGGKRKRHGSGEAKETELL...S....
Carubv10022734m.cru.20901991                294 ...........................NSEDE........AA.........GGGKRKRHGRWEGKETELL...S....
Bostr.28794s0002.1.p.adr.30657126           127 ...........................NSEDE........AA.........GGGKRKRHGRWEG..TELL...S....
Brara.D01611.1.p.bra.30620383               278 ...........................KSEDE........AG.........GGVGKR......GEESD.F...S....
Bol026461.bol.37343291                      277 ...........................KSEDE........AG.........GGGGKR......GKESE.F...S....
Brara.G01299.1.p.bra.30635851               288 ...........................NSEDE........AD.........GDGKRR.PVKRESAGTD.V...L....
Bol027793.bol.37343994                      280 ...........................NSEDE........AV.........GGGKRR.PVKRESAGTD.V...L....
mrna07673.1-v1.0-hybrid.fve.27266960        296 ...........................NSDDE........AD.........VDGKRRRHGRELNKENDLI...S....
Prupe.6G153800.1.p.ppe.32086540             312 ...........................NSDDE........AD.........VGGKRRRHVREPNKENDLL...S....
GSVIVT01037454001.vvi.17842075              300 ...........................NSDEE........TE.........MGGKRRRHGRGPTKETDLL...S....
orange1.1g003865m.csi.18122801              314 ...........................NSDDD........VD.........NGGKRRRHGRGPAKETDLL...S....
Gorai.004G227500.1.gra.26773739             295 ...........................NSEDE........TD.........IGGKRRRHGRGPTKETDIL...S....
Thecc1EG042011t1.tca.27426134               295 ...........................NSEDE........AD.........IGGKRRRHGRGPAKETD.L...S....
evm.model.supercontig_103.67.cpa.16404954   105 ...........................NSDDE........AD.........TGGKRRRHGRGPAKETDIH...S....
Potri.004G196400.1.ptr.26990875             313 ...........................NSDDE........AE.........AGGKRRRHGRPPAKDADLL...S....
SapurV1A.0615s0230.1.p.spu.31434857         315 ...........................NSDDE........AE.........AGGKRRRHGRPPAKDSDLL...S....
29942.m000751.rco.16814310                  315 ...........................NSDDE........VD.........TSGKRRRHGRAPAKENDIL...S....
Manes.11G111400.1.p.mes.32355670            308 ...........................NSDDE........VD.........AGGKRRRQGRTPAKETDLL...A....
Manes.S021700.1.p.mes.32354619              305 ...........................NSDDE........VD.........SGGKRRRHGRTQAKETDIL...S....
Eucgr.J00624.1.p.egr.32033287               307 ...........................NSDDE........MD.........TSGKRRRQGRGQTKEAD.L...S....
GSVIVT01027348001.vvi.17834761              307 ...........................NSDDE........TE.........TGDKRRRHGRGPIKESD.L...S....
Kalax.0225s0069.1.p.kla.32599097            300 ...........................NSDIE........TD.........ADGNRRRPNRGSTKETDLL...S....
Kaladp0081s0182.1.p.kfe.35761984            300 ...........................NSDIE........TD.........ADGNRRRPNRGSTKETDLL...S....
Kalax.0216s0065.1.p.kla.32585583            297 ...........................NSDAE........TD.........ADGKRRRPNRGSAKESDLL...L....
DCAR_002130.dca.36055141                    281 ...........................NSENE........VD.........VGGKMKRHERGLTKETD.F.........
Aqcoe2G007600.1.p.aco.33056562              312 ....................................................................LNQD.V...S....
Bradi3g03840.1.p.bdi.32813176               305 ...........................SSDND........ED.........YGSKRRKNRRGPQKEPEPL...S....
Brast04G302900.1.p.bst.32866594             375 ...........................SSEND........ED........YYGNKRRKNGRGPPKEPKPL...SK...
Sevir.1G087000.1.p.svi.32667668             320 ...........................SSEDD........TD.........YGEKKRKHGRGPQKEIEPL...S....
Pavir.J22990.1.p.pvi.30308347               311 ...........................SPEDD........AD.........YGERERKHGRGPRKEIEPL...S....
Pahal.A00287.1.pha.32525941                 303 ...........................SPEDD........AD.........YGERERKHGRGPQKEIELL...S....
OAV97825.1                                  283 DSEVTAVSEAAKVKTESEADVKNEVNSTNPDA...D....ADEYEEP.................DDEFCMAEAITGP
XP_003293253.1                              267 ...........................TEEDQ........NEETKMEE..DDKNEKSIASNNNNATTPTILTKSSINA
XP_005713144.1                              117 NQPVKEEGNAVSKFVEDEHDIKKEVEGTEPDQ....KQKTQD...VKE..AASEQSTVKKDENKVVKETV...SDEEE
XP_005702831.1                              251 ............VQHKDEEEMKEEEEEDNDDD........DD........TFKEQQIIHSNNMNMESEGI...STTD.
XP_009857252.1                              261 ..............V............VEKEE........GEATAANE..VLGVGDLVPANSADIRDENL.........
GES64941.1                                  206 ..............V............IEKEE........GETTAVGE..TLGVLDLLPARGGELRDEAL.........
KEY79445.1                                  256 ..............V............IEKEE........GETTAVGE..TLGVLDLLPVRGGDLRDEAL.........
XP_008869116.1                              215 ...........................VLAND........MD.........................DSARL.........
Q66I22                                      312 EKDKMEEGEESA...EKDGE.KASAGGSEGEE.KTEKEAPAEP..IPEPKKLSKKRKRKHSGDS.EDEASASESESDS
RXN03320.1                                  264 EKDKMVEGDEST...EKDA..AASAGGSEGEE.KTEKETPAEP..IPEPKKLSKKRKRKHSGDS.EDEASASDSESDS
XP_015195966.1                              269 DKEDKE..KKGE...SGGGSGEEKLKKGGEEE.QGEKSSAKE...ESEPKKLSKKRKRKRSGDS.EDEPSASDSDSDS
B1H1X4                                      297 EKEAPAKPENDK...EDESE.EKPVKPQEEEEKKVEKKVEKEEP.ERETRKPG.TRKRKRSGE..SDDGSDSESDTET
NP_001362205.1                              297 EKEPPAKPEKDK...EAETE.EKPVKPEEEEE...EKKVEKEEP.ERETRKPG.TRKRKRSGD..SDDGSDSESDTET
Q99MR6                                      305 KDEKKEDGKQAE...NDSSN.DDKTKKSEGDG...DKEEKKEEA.EKEAKK.SKKRNRKQSGDDSFDEGSVSESESES
Q9BXP5                                      305 KDEKKEDGKQAE...NDSSN.DDKTKKSEGDG...DKEEKKEDS.EKEAKKSSKKRNRKHSGDDSFDEGSVSESESES
XP_005549330.1                              305 KDEKKEDGKQSE...NDSSN.DDKTKKSEGDG...DKEEKKEDS.EKEAKKSSKKRNRKHSGDDSFDEGSVSESESES
H2ZUZ4                                      299 KEEKKEDHKKAD...SGEGS.EEKLKKSSDEE..GEKGGPKEET.EKETKKASKKRNRKRSGDDSEDEGSASESESES
XP_001748332.1                              267 ...........................DDDDE........GDDFDEDD..DKGPDHGLQRSPKRLRRRRLFHGSWIND
XP_002286542.1                              279 ...............SNLLLMRHY...RQYDD.Y......NE.......GGKEEEQQQQQSMMALDRNRI.........
XP_649316.1                                 186 .......................YVETTVGDQ......IQFE......VFAKPSRDKSYSLGTTTETESI.........
consensus>70                                    .............................ede.........d............k............d.....s....
```

```
Pp3c25_7680V3.1.p.ppa.32980429               262  ....................................AAPKAHPGS.................................
Pp3c16_11390V3.1.p.ppa.32986529              262  ....................................VAPTAHPGC.................................
Sphfalx0012s0220.1.p.sfa.32627721            331  ....................................AAPKAPAGS.................................
LOC_Os02g05610.1.osa.33135686                334  ....................................VAPKAHPVS.................................
LOC_Os08g40560.1.osa.33102829                251  ....................................AAPKAHPVS.................................
Pavir.Fb01956.1.p.pvi.30278011               241  ....................................SAPKAHPVS.................................
Pahal.F00690.1.pha.32488202                  244  ....................................AAPKAHPVS.................................
Sevir.6G221600.1.p.svi.32643744              245  ....................................AAPKAHPVS.................................
Sobic.007G200600.1.p.sbi.37935124            244  ....................................AAPKAHPVS.................................
GRMZM2G132780_P02.zma.31013431               243  ....................................AASKAHPVS.................................
Zm00008a003300_P01.zma.37201202              243  ....................................AASKAHPVS.................................
Bradi3g40630.1.p.bdi.32820467                237  ....................................AAPKAHPVS.................................
Brast03G282200.1.p.bst.32857559              237  ....................................AAPKAHPVS.................................
Sobic.010G249100.1.p.sbi.37910925            311  ....................................GAPKAHPVS.................................
GRMZM5G817439_P01.zma.30992522               310  ....................................GAPKAHPVS.................................
Pavir.Da00165.1.p.pvi.30193584               316  ....................................GAPKAHPVG.................................
Sevir.4G256700.1.p.svi.32645485              317  ....................................GAPKAHPVS.................................
LOC_Os06g48530.1.osa.33147743                321  ....................................GAPVAHPVS.................................
Bradi1g33970.1.p.bdi.32801481                301  ....................................AAPKAHPVS.................................
Brast07G193800.1.p.bst.32871232              301  ....................................AAPKAHPVS.................................
Migut.N02418.1.p.mgu.28925732                342  ....................................SAPKAHPVC.................................
Migut.D00714.1.p.mgu.28928758                358  ....................................SAPKAHPVS.................................
Medtr8g043980.1.mtr.31070730                 336  ....................................AAPKAHPVS.................................
Tp57577_TGAC_v2_mRNA25888.tpr.35955570       227  ....................................AAPMTHPIS.................................
Medtr3g006760.1.mtr.31058945                 309  ....................................AAPKAHPVS.................................
Glyma.08G227300.1.p.gma.30540922             319  ....................................AAPKAHPIS.................................
Phvul.011G187000.1.p.pvu.37156720            326  ....................................AAPKAHPIS.................................
Phvul.005G010800.1.p.pvu.37152760            304  ....................................AAPKAHPIS.................................
Glyma.06G314900.1.p.gma.30549408             314  ....................................AAPKAHPIS.................................
Solyc01g009090.2.1.sly.36140441              343  ....................................AAPKAHLVS.................................
Thhalv10001919m.esa.20200705                 318  ....................................AAPKAPSFT.................................
AT2G27100.1.ath.19641299                     325  ....................................AAPKAPSFT.................................
Q9ZVD0                                       325  ....................................AAPKAPSFT.................................
Araha.4729s0005.1.p.aha.28848294             207  ....................................AAPKAPSFT.................................
AL4G20790.t1.aly.35923270                    322  ....................................AAPKAPSFT.................................
Bostr.29505s0043.1.p.adr.30675300            129  ....................................AAPKAPSFT.................................
Carubv10022733m.cru.20903468                 320  ....................................AAPKAPSFT.................................
AL4G20820.t1.aly.35924196                    320  ....................................AAPKAPSFT.................................
Carubv10022734m.cru.20901991                 321  ....................................AAPQAPSFT.................................
Bostr.28794s0002.1.p.adr.30657126            152  ....................................AAPKAPSFT.................................
Brara.D01611.1.p.bra.30620383                298  ....................................AAPKAPSFT.................................
Bol026461.bol.37343291                       297  ....................................AAPKAPSFT.................................
Brara.G01299.1.p.bra.30635851                313  ....................................AAPKAPSFT.................................
Bol027793.bol.37343994                       305  ....................................AAPKAPSFT.................................
mrna07673.1-v1.0-hybrid.fve.27266960         323  ....................................TAPKAHPVS.................................
Prupe.6G153800.1.p.ppe.32086540              339  ....................................AAPKAHPVS.................................
GSVIVT01037454001.vvi.17842075               327  ....................................VAPKAHPVS.................................
orange1.1g003865m.csi.18122801               341  ....................................AAPKAHPIS.................................
Gorai.004G227500.1.gra.26773739              322  ....................................LAPKAHLVS.................................
Thecc1EG042011t1.tca.27426134                321  ....................................AAPKAHPVS.................................
evm.model.supercontig_103.67.cpa.16404954    132  ....................................AAPKAHPVG.................................
Potri.004G196400.1.ptr.26990875              340  ....................................AAPKAHPVS.................................
SapurV1A.0615s0230.1.p.spu.31434857          342  ....................................AAPKAHPVS.................................
29942.m000751.rco.16814310                   342  ....................................AAPKAHPVS.................................
Manes.11G111400.1.p.mes.32355670             335  ....................................AAPKAHPVS.................................
Manes.S021700.1.p.mes.32354619               332  ....................................AAPKAHPVS.................................
Eucgr.J00624.1.p.egr.32033287                333  ....................................AAPKAHPVS.................................
GSVIVT01027348001.vvi.17834761               333  ....................................TAAVAHPNS.................................
Kalax.0225s0069.1.p.kla.32599097             327  ....................................AAPKAHPVS.................................
Kaladp0081s0182.1.p.kfe.35761984             327  ....................................AAPKAHPVS.................................
Kalax.0216s0065.1.p.kla.32585583             324  ....................................AAPKAHPVS.................................
DCAR_002130.dca.36055141                     306  ......................................PKAHPVS.................................
Aqcoe2G007600.1.p.aco.33056562                    ..............................................................................
Bradi3g03840.1.p.bdi.32813176                332  ....................................TAPKAHPIS.................................
Brast04G302900.1.p.bst.32866594              404  ....................................TAPKAHPIS.................................
Sevir.1G087000.1.p.svi.32667668              347  ....................................AAPKAHPVS.................................
Pavir.J22990.1.p.pvi.30308347                338  ....................................AAPRAHPVS.................................
Pahal.A00287.1.pha.32525941                  330  ....................................AAPKAHPVS.................................
OAV97825.1                                   336  NTG.................................LAPSSALGSLIG.....ESMRGKVG.SDDIVVLP....PADN
XP_003293253.1                               308  ASNI...............................................................NDKDKS
XP_005713144.1                               183  RGGL..................VTEKK...EDPILDSAPTGEEGGGTG......LLPGQSS.QEHVLFLPLRREHQKD
XP_005702831.1                               297  ....................................SEP.........................................SC
XP_009857252.1                               295  ....................................FQP...........................................
GES64941.1                                   240  ....................................SQP...........................................
KEY79445.1                                   290  ....................................SQP...........................................
XP_008869116.1                                    ..............................................................................
Q66I22                                       382  DSDSNSHCSEKPSERERE.PEEVEEKEEEEEEEGEAADGKEQ..TDEQTEREKEKEKKV...KDDQPPRP.RPLHRTC
RXN03320.1                                   333  DSDSNSHCSEKPSEREQE.PDEGEDKEEEEEEEGEAADVKER..TEEQKERDKEKEKKP...KDDQPPRP.RPLHRTC
XP_015195966.1                               337  ESG.....SEKPARDEER.ED...EEEDEEKEEVVVKERKEEKLKDREKEREREEKKVKEKEVEIPPP.RPLHKTC
B1H1X4                                       367  ASP.......KPKAETPN.QNGRAEETPKKEEDTEKQKEKQ.....................KED.TVKP.RPLHKTC
NP_001362205.1                               364  ASP.......KPKAETPS.QNGRAEDTPKKEEETEKQKEKQ.....................KED.PVKP.RPLHKTC
Q99MR6                                       374  EGG.......QAEEEKEEAEEALKEKEKPKEEE..KEKPKD.....................AAGLECKP.RPLHKTC
Q9BXP5                                       375  ESG.......QAEEEKEEAEEALKEKEKPKEEE..WEKPKD.....................AAGLECKP.RPLHKTC
XP_005549330.1                               375  ESG.......QAEEEKEEAEEALKEKEKPKEEE..WEKPKD.....................AAGLECKP.RPLHKTC
H2ZUZ4                                       370  ESG.......QVAEEKEE..............................................PGNLSLP.RITSSSL
XP_001748332.1                               308  WNRI.............................VDMDSP...................................IC
XP_002286542.1                               317  .................E..................RHAKSHLHSFI...........................KSDA
XP_649316.1                                  221  ........................EY..........LAP..........................ILP.SGVIDVN
consensus>70                                      .....................................apka.....................................
```

```
Pp3c25_7680V3.1.p.ppa.32980429                   ..............................................................................
Pp3c16_11390V3.1.p.ppa.32986529                  ..............................................................................
Sphfalx0012s0220.1.p.sfa.32627721                ..............................................................................
LOC_Os02g05610.1.osa.33135686                    ..............................................................................
LOC_Os08g40560.1.osa.33102829                    ..............................................................................
Pavir.Fb01956.1.p.pvi.30278011                   ..............................................................................
Pahal.F00690.1.pha.32488202                      ..............................................................................
Sevir.6G221600.1.p.svi.32643744                  ..............................................................................
Sobic.007G200600.1.p.sbi.37935124                ..............................................................................
GRMZM2G132780_P02.zma.31013431                   ..............................................................................
Zm00008a003300_P01.zma.37201202                  ..............................................................................
Bradi3g40630.1.p.bdi.32820467                    ..............................................................................
Brast03G282200.1.p.bst.32857559                  ..............................................................................
Sobic.010G249100.1.p.sbi.37910925                ..............................................................................
GRMZM5G817439_P01.zma.30992522                   ..............................................................................
Pavir.Da00165.1.p.pvi.30193584                   ..............................................................................
Sevir.4G256700.1.p.svi.32645485                  ..............................................................................
LOC_Os06g48530.1.osa.33147743                    ..............................................................................
Bradi1g33970.1.p.bdi.32801481                    ..............................................................................
Brast07G193800.1.p.bst.32871232                  ..............................................................................
Migut.N02418.1.p.mgu.28925732                    ..............................................................................
Migut.D00714.1.p.mgu.28928758                    ..............................................................................
Medtr8g043980.1.mtr.31070730                     ..............................................................................
Tp57577_TGAC_v2_mRNA25888.tpr.35955570           ..............................................................................
Medtr3g006760.1.mtr.31058945                     ..............................................................................
Glyma.08G227300.1.p.gma.30540922                 ..............................................................................
Phvul.011G187000.1.p.pvu.37156720                ..............................................................................
Phvul.005G010800.1.p.pvu.37152760                ..............................................................................
Glyma.06G314900.1.p.gma.30549408                 ..............................................................................
Solyc01g009090.2.1.sly.36140441                  ..............................................................................
Thhalv10001919m.esa.20200705                     ..............................................................................
AT2G27100.1.ath.19641299                         ..............................................................................
Q9ZVD0                                           ..............................................................................
Araha.4729s0005.1.p.aha.28848294                 ..............................................................................
AL4G20790.t1.aly.35923270                        ..............................................................................
Bostr.29505s0043.1.p.adr.30675300                ..............................................................................
Carubv10022733m.cru.20903468                     ..............................................................................
AL4G20820.t1.aly.35924196                        ..............................................................................
Carubv10022734m.cru.20901991                     ..............................................................................
Bostr.28794s0002.1.p.adr.30657126                ..............................................................................
Brara.D01611.1.p.bra.30620383                    ..............................................................................
Bol026461.bol.37343291                           ..............................................................................
Brara.G01299.1.p.bra.30635851                    ..............................................................................
Bol027793.bol.37343994                           ..............................................................................
mrna07673.1-v1.0-hybrid.fve.27266960             ..............................................................................
Prupe.6G153800.1.p.ppe.32086540                  ..............................................................................
GSVIVT01037454001.vvi.17842075                   ..............................................................................
orange1.1g003865m.csi.18122801                   ..............................................................................
Gorai.004G227500.1.gra.26773739                  ..............................................................................
Thecc1EG042011t1.tca.27426134                    ..............................................................................
evm.model.supercontig_103.67.cpa.16404954        ..............................................................................
Potri.004G196400.1.ptr.26990875                  ..............................................................................
SapurV1A.0615s0230.1.p.spu.31434857              ..............................................................................
29942.m000751.rco.16814310                       ..............................................................................
Manes.11G111400.1.p.mes.32355670                 ..............................................................................
Manes.S021700.1.p.mes.32354619                   ..............................................................................
Eucgr.J00624.1.p.egr.32033287                    ..............................................................................
GSVIVT01027348001.vvi.17834761                   ..............................................................................
Kalax.0225s0069.1.p.kla.32599097                 ..............................................................................
Kaladp0081s0182.1.p.kfe.35761984                 ..............................................................................
Kalax.0216s0065.1.p.kla.32585583                 ..............................................................................
DCAR_002130.dca.36055141                         ..............................................................................
Aqcoe2G007600.1.p.aco.33056562                   ..............................................................................
Bradi3g03840.1.p.bdi.32813176                    ..............................................................................
Brast04G302900.1.p.bst.32866594                  ..............................................................................
Sevir.1G087000.1.p.svi.32667668                  ..............................................................................
Pavir.J22990.1.p.pvi.30308347                    ..............................................................................
Pahal.A00287.1.pha.32525941                      ..............................................................................
OAV97825.1                                   371 QLFIKSISPDISRTELENHCKQV.......EGFDYLALSDPHTGKKLHRVGWVAFLPG....................
XP_003293253.1                               318 TLFIKAVSPACTKDELLEVLNKV..APAGEQSVVTKLTLSEPVRYKNFYRLGWVTYKNC...................
XP_005713144.1                               233 TIFMRSIPTNLCRDDLTAVLKHG..EDGTLSLNLRRLKLGDINPLRSLERFGWAVYDSE...................
XP_005702831.1                               302 CVFLRGIPCWMSRQLLENTLKYVKKEDKWEPIQLLRLKLSEVKPEKNLERFGWAYYESV...................
XP_009857252.1                               298 TLLIKTIAPSVSRQNLEAFCKEH...LGEEEGGFKWLSLSDPNPSKRYHRIGWIMLHPAPEAPIAQ.....DEMKDDD
GES64941.1                                   243 ALLIKTLAPNVSREKIEEFCKEH...LGEQDGGFKWLSLSDPNPSKKYHRMGWIVLNPAPDVTVVERGDGRDEEGEEM
KEY79445.1                                   293 ALLIKTLAPNVSRAKIEEFCKEH...LGEQDGGFKWLSLSDPNPSKKFHRMGWIMLHPAPESAVIERGDGREEEGEEM
XP_008869116.1                               227 .LYIRRIPCSCPFSVISEAIHNQ.......GPFDELLLSDPLKKRDFERSAYILYPTA....................
Q66I22                                       453 SLFMRSIAPTISKAEIVALCRRY.......PGFMRVCLSEPQPERRFFRRCWVTFDRG....................
RXN03320.1                                   404 SLFMRSIAPTISKAEIVANRNQS.......VG...........DLKFFRRCWVTFDRG....................
XP_015195966.1                               405 SLFMRSIAPTISKAEIVALCRRY.......PGFMRVALSEPQPERR................................
B1H1X4                                       414 SIFMRNIPPNISKAEITALCKRY.......PGFMRVALSEPQPERRFLRKAYVTFDRS....................
NP_001362205.1                               411 SIFMRNIPPNISKAEITALCKRY.......PGFMRVALSEPQPERRFLRKAYVTFDRS....................
Q99MR6                                       421 SLFMRNIAPNISRAEIISLCKRY.......PGFMRVALSEPQPERRFFRRGWVTFDRS....................
Q9BXP5                                       422 SLFMRNIAPNISRAEIISLCKRY.......PGFMRVALSEPQPERRFFRRGWVTFDRS....................
XP_005549330.1                               422 SLFMRNIAPNISRAEIISLCKRY.......PGFMRVALSEPQPERRFFRRGWVTFDRS....................
H2ZUZ4                                       395 TLMLMCVC.......VFQLCKRY.......PGFMRVALSDPQPERRFFRRGWVTFDRS....................
XP_001748332.1                               320 QAVVADLEPRLNR..FGYSLKDL..TWIGEPEPLADLMLKDPYKRRKISLSGALFNHII...................
XP_002286542.1                               333 CVKIMDVPSHCTNEQIMNALMEY..CTVKPPSGVWSGEVYVPTAGRPYHRTVFVVFSSR...................
XP_649316.1                                  236 ITCLRSIYWNLNTSLIPKNVSYQ.......GKYLLTILSAS....DLSLMGQKKFYPYCS..................
consensus>70                                     ..............................................................................
```

```
Pp3c25_7680V3.1.p.ppa.32980429                     271  ..........................................................SDPKRVLKDIGIGRALVK.K
Pp3c16_11390V3.1.p.ppa.32986529                    271  ..........................................................SDPQRVLKDIELGRSLIK.K
Sphfalx0012s0220.1.p.sfa.32627721                  340  ..........................................................SDPKRVLKDIELCQSLIR.K
LOC_Os02g05610.1.osa.33135686                      343  ..........................................................SHYRRIQTDIHQTLALVK.K
LOC_Os08g40560.1.osa.33102829                      260  ..........................................................SEPRRIRIDVELAQALIC.K
Pavir.Fb01956.1.p.pvi.30278011                     250  ..........................................................SEARRIRIDIEQAQALIC.K
Pahal.F00690.1.pha.32488202                        253  ..........................................................SEARRIRIDIEQAQALIC.K
Sevir.6G221600.1.p.svi.32643744                    254  ..........................................................SEARRIRSDIEQAQALIC.K
Sobic.007G200600.1.p.sbi.37935124                  253  ..........................................................SEVRRIRIDIEQAQALIC.K
GRMZM2G132780_P02.zma.31013431                     252  ..........................................................SEVRRIRIDIEQAQVLIR.K
Zm00008a003300_P01.zma.37201202                    252  ..........................................................SEVRRIRIDIEQAQALIR.K
Bradi3g40630.1.p.bdi.32820467                      246  ..........................................................SEARRVSIDIQQAQALIS.K
Brast03G282200.1.p.bst.32857559                    246  ..........................................................SEARRVTIDIQQAQALIS.K
Sobic.010G249100.1.p.sbi.37910925                  320  ..........................................................SESRRVQADIEQTLALVR.K
GRMZM5G817439_P01.zma.30992522                     319  ..........................................................SESRRVQADIEQTLALVR.K
Pavir.Da00165.1.p.pvi.30193584                     325  ..........................................................SESHRIQADIEHTLALVR.K
Sevir.4G256700.1.p.svi.32645485                    326  ..........................................................SESRRIQADIEQTLALVR.K
LOC_Os06g48530.1.osa.33147743                      330  ..........................................................SESRRVQVDIEQALALVR.K
Bradi1g33970.1.p.bdi.32801481                      310  ..........................................................SEPRRIQTDIEQTLGLVR.R
Brast07G193800.1.p.bst.32871232                    310  ..........................................................SEPRRIQTDIEQTLGLVR.R
Migut.N02418.1.p.mgu.28925732                      351  ..........................................................SEPKRILIDIEQALALVQ.K
Migut.D00714.1.p.mgu.28928758                      367  ..........................................................SEPRRIQVDVEQAQALVR.K
Medtr8g043980.1.mtr.31070730                       345  ..........................................................SEPRRIQADVQQAQALVR.K
Tp57577_TGAC_v2_mRNA25888.tpr.35955570             236  ..........................................................SEPKRIQADVQQAQSLVH.K
Medtr3g006760.1.mtr.31058945                       318  ..........................................................SEPRRIQEDIHQVQAVVR.K
Glyma.08G227300.1.p.gma.30540922                   328  ..........................................................TEPRRIQADVQQAQALVR.K
Phvul.011G187000.1.p.pvu.37156720                  335  ..........................................................SEPRRIQADVQQAQVLVR.K
Phvul.005G010800.1.p.pvu.37152760                  313  ..........................................................SEPRRIQTDIVKAQAVVR.K
Glyma.06G314900.1.p.gma.30549408                   323  ..........................................................SEPRRIQADIQQAQAVVR.K
Solyc01g009090.2.1.sly.36140441                    352  ..........................................................SDPRRVQIDVEQAQALVR.K
Thhalv10001919m.esa.20200705                       327  ..........................................................SDPKRILTDIDQAQALVR.K
AT2G27100.1.ath.19641299                           334  ..........................................................SDPKRILTDVEQTQALVR.K
Q9ZVD0                                             334  ..........................................................SDPKRILTDVEQTQALVR.K
Araha.4729s0005.1.p.aha.28848294                   216  ..........................................................SDPKRILTDVEQTQALVR.K
AL4G20790.t1.aly.35923270                          331  ..........................................................SDPKRILTDVEQTQALVR.K
Bostr.29505s0043.1.p.adr.30675300                  138  ..........................................................SDPKRILTDIEQAQALVR.K
Carubv10022733m.cru.20903468                       329  ..........................................................SDPKRILTDIEQAQALVR.K
AL4G20820.t1.aly.35924196                          329  ..........................................................SDPKRILTDIEQAHALVR.K
Carubv10022734m.cru.20901991                       330  ..........................................................SDPKRILTDIEQAQALVR.K
Bostr.28794s0002.1.p.adr.30657126                  161  ..........................................................SDPKRILIDVEQAQALVR.K
Brara.D01611.1.p.bra.30620383                      307  ..........................................................SDAKRILTDIEQAQALVR.K
Bol026461.bol.37343291                             306  ..........................................................SDPKRILTDIEQAQALVL.K
Brara.G01299.1.p.bra.30635851                      322  ..........................................................SDPKRILTDIEQTQALVR.K
Bol027793.bol.37343994                             314  ..........................................................SDPKRILTDIEQTQALVR.K
mrna07673.1-v1.0-hybrid.fve.27266960               332  ..........................................................SDPRRIQVDVEQAQALVR.K
Prupe.6G153800.1.p.ppe.32086540                    348  ..........................................................SEPRRIQVDIEQAQALVR.K
GSVIVT01037454001.vvi.17842075                     336  ..........................................................SEPRRIQVDIEQAQALVR.K
orange1.1g003865m.csi.18122801                     350  ..........................................................SEPRRIQVDIEQAQALVR.K
Gorai.004G227500.1.gra.26773739                    331  ..........................................................SDPRRIQIDIGQAQGLVR.K
Thecc1EG042011t1.tca.27426134                      330  ..........................................................SEPRRIQIDIEQAQGLVR.K
evm.model.supercontig_103.67.cpa.16404954          141  ..........................................................SEPRRIQVDIEQAQALVR.K
Potri.004G196400.1.ptr.26990875                    349  ..........................................................SEPKRIQVDIEQAQALVC.K
SapurV1A.0615s0230.1.p.spu.31434857                351  ..........................................................SEPKRIQIDIEQAQALVC.K
29942.m000751.rco.16814310                         351  ..........................................................SEPRRIQIDVEQAQALVR.K
Manes.11G111400.1.p.mes.32355670                   344  ..........................................................SESRRIQVDIEQSQALVR.K
Manes.S021700.1.p.mes.32354619                     341  ..........................................................SEPRRIQIDVEQAQALVR.K
Eucgr.J00624.1.p.egr.32033287                      342  ..........................................................SEPRRVQVDIEQAQALVR.K
GSVIVT01027348001.vvi.17834761                     342  ..........................................................SEPRRIQADIEQALALVR.K
Kalax.0225s0069.1.p.kla.32599097                   336  ..........................................................SDPRRVQVDIEQTQALIR.K
Kaladp0081s0182.1.p.kfe.35761984                   336  ..........................................................SDPRRVQVDIEQAQALIR.K
Kalax.0216s0065.1.p.kla.32585583                   333  ..........................................................SDPRRIQVDIEQTQALIR.K
DCAR_002130.dca.36055141                           313  ..........................................................SEPRRVIIDIEQAQALVR.K
Aqcoe2G007600.1.p.aco.33056562                     318  ................................................................QDDIEQAHALLC.K
Bradi3g03840.1.p.bdi.32813176                      341  ..........................................................SKYRRIQTDIVQTLALVK.K
Brast04G302900.1.p.bst.32866594                    413  ..........................................................SKYRRIQTDIVQTLALVK.K
Sevir.1G087000.1.p.svi.32667668                    356  ..........................................................SQYRRIHTDIDRTLALVK.K
Pavir.J22990.1.p.pvi.30308347                      347  ..........................................................SQYRRIHVDIERTLALLK.K
Pahal.A00287.1.pha.32525941                        339  ..........................................................SQYRRIHADIERTLALLK.K
OAV97825.1                                         422  ............TDMKAAESALGESKINN......FTLHLMITERPALQKLRTCPGIMNTHERIIKDLGQIRKLAA.S
XP_003293253.1                                     375  ............DLAAKALKELNGYKMKD......FDLYLNINKEDAERRFKITPHIASTENRIYIDLEQSTTLMR.L
XP_005713144.1                                     290  ............ETAAKAITAVKGVKVVSRKDKNRIDCMLNLERKKKFSQGRVLPAAFGTPERMKYDVAQSAKMMR.C
XP_005702831.1                                     361  ............DEASRVMEYWNGQKLWKPNQEQEYVLDMRFNHRKKYDKRRILPELFQSARRLEHDAKQIVFVMR.H
XP_009857252.1                                     368  DEGS.AKPHV.PSTAEKALEAINGKTVKDEQRGD.FTCHVGVHNPPMNPRKKALWDLFSAPERIEKDLELVRRLVN.K
GES64941.1                                         318  DHGD.TNGATTVTAAEKALEAVNDQTIHDPVHGD.FVCHVGVHAPPSQPRKKALWDLFSAPQRIERDLELARRLVL.R
KEY79445.1                                         368  DHDQGANGTVTVSAAEKALEAVNDKTIHDPVHGD.FVCHVGVHVPPSQTRKKALWDLFSAPERIERDLELARRLVC.K
XP_008869116.1                                     277  ............AAATAAMPKLQNLLVEAPEMPHPLRLQVMIYR........KTPSYMSLPDRITYDFHQALHVAT.L
Q66I22                                             504  ............VNIKEICWNLQNIRLRD......CELAPGVNR.DLARRVRNVNGITQHKQVLRNDIKLAAKLIH.A
RXN03320.1                                         444  ............VNIKEICWNLQNIRLRD......CELAPGVNR.DLARRVRNVNGITQHKQVLRNDIKLAAKLIH.A
XP_015195966.1                                     444  .....................................LTPCL....LSRRLQ..........................
B1H1X4                                             465  ............VNIKEICWSVQNIRLRE......CELSPGVNR.DLTYRVRNINGITLHRPIVRNDIKLAARLIH.A
NP_001362205.1                                     462  ............VNIKEICWSVQNIRLRE......CELSPGVNR.DLTYRVRNINGITMHRPIVRNDIKLAARLIH.A
Q99MR6                                             472  ............VNIKEICWNLQNIRLRE......CELSPGVNR.DLTRRVRNINGITQHKQIVRNDIKLAAKLIH.T
Q9BXP5                                             473  ............VNIKEICWNLQNIRLRE......CELSPGVNR.DLTRRVRNINGITQHKQIVRNDIKLAAKLIH.T
XP_005549330.1                                     473  ............VNIKEICWNLQNIRLRE......CELSPGVNR.DLTRRVRNINGITQHKQIVRNDIKLAAKLIH.T
H2ZUZ4                                             439  ............VNIKEICWNLQNIRVSG......TVSSVVVAR.RLLSALVTMVYVNVFKQAIVTHCKLNIEFINDS
XP_001748332.1                                     375  ............ARLSLSVSLFSVSLSNSLSLSNSLSLSLSLSLLDPSPSLKCLSLKLSLSNSLSQTLSLKLSFLSLS
XP_002286542.1                                     390  ............EAKESMLDNLNRSNEDS......VDCTDVYGRKAKQVETKVLSAAVSSRERISRDKEAATTIAT.I
XP_649316.1                                        285  ...................................FTLNGSVYS........NQSYLITYQEGAFCDLEILNKNPA.L
consensus>70                                            ..........................................................se..ri..dieq...lv..k
```

```
                                                  .                         .              .                                .
Pp3c25_7680V3.1.p.ppa.32980429               290  LDAEK....................GIEG..NLLT..VDQSAGSERTM.............GGGSV.....GPIVIVR
Pp3c16_11390V3.1.p.ppa.32986529              290  LDAEK....................GIEG..NLLS..VDQNTENERTV.............GGGSV.....GPIVIVR
Sphfalx0012s0220.1.p.sfa.32627721            359  LDSEK....................GIEG..NILA.PADQQNVEGDRS............ISTGSF.....GPIVIVR
LOC_Os02g05610.1.osa.33135686                362  LDEEK....................GIVG..NILT.TGDHTKSNGDKS.............YAGST.....GPLVIVR
LOC_Os08g40560.1.osa.33102829                279  LDSEK....................GIEN..NVLS.GSDHDRAEKDKS.............H.GSM.....GPIVIIR
Pavir.Fb01956.1.p.pvi.30278011               269  LDSEK....................GVEN..NVLS.SSDHDKPDRDKS.............H.GSM.....GPIVIIR
Pahal.F00690.1.pha.32488202                  272  LDSEK....................GIEN..NVLS.SSDHDKPDRDKS.............H.GSV.....GPIVIIR
Sevir.6G221600.1.p.svi.32643744              273  LDSEK....................GIEN..NVLS.SSDHDKTDRDKS.............H.GSM.....GPIVIIR
Sobic.007G200600.1.p.sbi.37935124            272  LDSEK....................GIEN..SVLS.SSDHEKSDRDKS.............H.GSM.....GPIVIIR
GRMZM2G132780_P02.zma.31013431               271  LDSEK....................GIEN..NVLS.SSDHEKSDRDKS.............H.GSV.....GPIVIIQ
Zm00008a003300_P01.zma.37201202              271  LDSEK....................GIEN..NVLS.SSDHEKSDRDKS.............H.GSV.....GPIVIIQ
Bradi3g40630.1.p.bdi.32820467                265  LDSEK....................GIEN..NVLS.GTDDDKAARDTS.............H.GSM.....GPIVIIR
Brast03G282200.1.p.bst.32857559              265  LDSEK....................GIEN..NVLS.GTDDDKSARDTS.............R.GSM.....GPIVIIR
Sobic.010G249100.1.p.sbi.37910925            339  LDAEK....................AIEG..NVLS.SCDHDKSDVDKS.............HIGSM.....GPIIIIR
GRMZM5G817439_P01.zma.30992522               338  LDAEK....................GIDG..NVLS.SGDHDKSDVDKS.............HIGSM.....GPIVIIR
Pavir.Da00165.1.p.pvi.30193584               344  LDAEK....................GIEG..NVLS.SGDHDKPDVDKS.............HIGSM.....GPIIIIR
Sevir.4G256700.1.p.svi.32645485              345  LDAEK....................GIEG..NVLL.SGDHDKSDGDKS.............HIGSM.....GPIIIIR
LOC_Os06g48530.1.osa.33147743                349  LDTEK....................GIVG..NILS.SGDHDKSDVDKS.............HIGSM.....GPIIIIR
Bradi1g33970.1.p.bdi.32801481                329  LDTEK....................GILG..NILS.SGDHEKSDVDKS.............HIGSM.....GPIIIIR
Brast07G193800.1.p.bst.32871232              329  LDTEK....................GILG..NILS.SGDHEKSDVDKS.............HIGSM.....GPIIIIR
Migut.N02418.1.p.mgu.28925732                370  LDTEK....................GIED..NVLF.RAENDRMSRDKS.............HGSSS.....GPVVIIR
Migut.D00714.1.p.mgu.28928758                386  LDLEK....................GIED..NVLS.RADNDRMSRDKS.............HGSSS.....GPIVIIR
Medtr8g043980.1.mtr.31070730                 364  LDTEK....................RIGD..NILC.S..........................................IIIR
Tp57577_TGAC_v2_mRNA25888.tpr.35955570       255  LDTEK....................GIGD..NILC.SSDHSK.ADDKS.............HSGSA.....GPTIIIR
Medtr3g006760.1.mtr.31058945                 337  LDKEK....................GIED..NILC.TNDHSK.NSDKA.............RSGSV.....GPVVIIR
Glyma.08G227300.1.p.gma.30540922             347  LDTEK....................GIED..NILC.ISDHNR.NDDKA.............HSASV.....APIVIIR
Phvul.011G187000.1.p.pvu.37156720            354  LDAEK....................GIED..NILCSSSDHNK.NDDKA.............HSGSV.....GPIVIIR
Phvul.005G010800.1.p.pvu.37152760            332  LDTEK....................GIED..NILC.TSDHNR.SDDKS.............HSGSV.....GPIIIIR
Glyma.06G314900.1.p.gma.30549408             342  LDREK....................GIED..NILC.TSDHNK.NDDKA.............HSGSV.....GPIVIIR
Solyc01g009090.2.1.sly.36140441              371  LDSEK....................GIED..NILS.RSDNDRGSRDKS.............H.GST.....GPVIIIR
Thhalv10001919m.esa.20200705                 346  LDTEK....................GIVE..NVLS.GSESEKSGRDKL.............PTGST.....GPVIIIR
AT2G27100.1.ath.19641299                     353  LDSEK....................KIEE..NVLQ.GSETEKSGREKL.............HSGST.....GPVVIIR
Q9ZVD0                                       353  LDSEK....................KIEE..NVLQ.GSETEKSGREKL.............HSGST.....GPVVIIR
Araha.4729s0005.1.p.aha.28848294             235  LDSEK....................KIEE..NVLQ.GSETEKSGREKL.............HSGST.....GPVVIIR
AL4G20790.t1.aly.35923270                    350  LDFEK....................KIEE..NVLQ.GSETEKSGREKL.............HSGST.....GPVVIIR
Bostr.29505s0043.1.p.adr.30675300            157  LDSEK....................RIEE..NVLS.GSETEKSGREKL.............HSGST.....GPVIIIR
Carubv10022733m.cru.20903468                 348  LDSEK....................RIEE..NLLS.GSETEKSGRDKL.............HSGST.....GPVIIIR
AL4G20820.t1.aly.35924196                    348  LDSEK....................GIEE..NVLS.GSENDKLGREKL.............HSGST.....GPVIIIR
Carubv10022734m.cru.20901991                 349  LDSEK....................RIEE..NLLS.GSETEKSGREKL.............HSGST.....GPVIIIR
Bostr.28794s0002.1.p.adr.30657126            180  LDSEK....................GIEE..NVLS.GSENEKMGREKL.............HSGST.....GPVIIIR
Brara.D01611.1.p.bra.30620383                326  LDSEK....................GIVE..NVLS.GSETETSGKDKS.............HSGST.....GPVIIIR
Bol026461.bol.37343291                       325  LDSEK....................GIVE..NVLS.GSETETSGKDKS.............HSGST.....GPVIIIR
Brara.G01299.1.p.bra.30635851                341  LDAEK....................GIKE..NVLS.GSETEKSGRDNV.............HSGST.....GPVIIIR
Bol027793.bol.37343994                       333  LDAEK....................GIKE..NVLS.GSETEKSG.SNV.............HSGST.....GPVIIIR
mrna07673.1-v1.0-hybrid.fve.27266960         351  LDSEK....................GIVE..NILC.APENDKMNREKS.............HGGST.....GPVVIIR
Prupe.6G153800.1.p.ppe.32086540              367  LDSEK....................GIVE..NILA.GPDNDK.NREKL.............HGGST.....GPVIIVR
GSVIVT01037454001.vvi.17842075               355  LDSEK....................GIEE..NILC.RADNDKLNREKS.............HGGSI.....GPVIIIR
orange1.1g003865m.csi.18122801               369  LDSEK....................GITE..NILC.GSDNDKMNREKS.............H.GST.....GPVIIIR
Gorai.004G227500.1.gra.26773739              350  LDSEK....................GIEE..NILS.GSDNDKISRDKS.............HGSFA.....GPVIIVR
Thecc1EG042011t1.tca.27426134                349  LDSEK....................GIEE..NILS.GSDNDKINRDKS.............HGGLT.....GPVIIVR
evm.model.supercontig_103.67.cpa.16404954    160  LDSEK....................GVEE..NILC.GSENDKVGRDKS.............HSGST.....GPVIIIR
Potri.004G196400.1.ptr.26990875              368  LDSEK....................GVED..NILS.GSDNEKMSREKS.............HGSST.....GPVIIIR
SapurV1A.0615s0230.1.p.spu.31434857          370  LDSEK....................GVED..NILS.GPDNEKMSREKS.............LGSST.....GPVIIIR
29942.m000751.rco.16814310                   370  LDSEK....................GIEE..NILG.GSDNEKMNREKP.............HGSSS.....GPVIIIR
Manes.11G111400.1.p.mes.32355670             363  LDSEK....................GIEE..NILG.GSDNDKTNREKS.............HSSYT.....GPVIIIR
Manes.S021700.1.p.mes.32354619               360  LDSEK....................GIEE..NILG.GSDNDKINREKS.............HSSST.....GPVIIIR
Eucgr.J00624.1.p.egr.32033287                361  LDSEK....................GIEE..NILG.GPD.DKINREKS.............HGGSS.....GPVIIIR
GSVIVT01027348001.vvi.17834761               361  LDSEK....................GVED..NILC.SAEQKKTDSDKS.............HSGSM.....GPIIIIR
Kalax.0225s0069.1.p.kla.32599097             355  LDSEK....................GIEE..NILR.GADPGKPNRDKS.............HGGS......GPVIIIR
Kaladp0081s0182.1.p.kfe.35761984             355  LDSEK....................GIEE..NILR.GADTGKPNRDKS.............HGGS......GPVIIIR
Kalax.0216s0065.1.p.kla.32585583             352  LDSEK....................GIEE..NILR.GADTNKSSRDKS.............FGGS......GPVIIIR
DCAR_002130.dca.36055141                     332  LDLEK....................GVED..NILC.RTDNERTSRDKS.............HGSSS.....GPVIIIR
Aqcoe2G007600.1.p.aco.33056562               331  LDKEK....................GVED..NMLW....NMKHNNEKS.............HGGSM.....GPIVIVQ
Bradi3g03840.1.p.bdi.32813176                360  LDAEK....................GIVG..NILS.TSDHGMSDVDRS.............HVGSV.....GPIVIVQ
Brast04G302900.1.p.bst.32866594              432  LDAEK....................GIVG..NLLS.TSDPGNSDVDRS.............HVGSM.....RPIVIVR
Sevir.1G087000.1.p.svi.32667668              375  LDSEK....................DILG..NILL.TGDHDNSNVDQS.............HIRSM.....GPVVIVR
Pavir.J22990.1.p.pvi.30308347                366  LDSEK....................DIVG..NMLL.TGVHGKSNVDES.............FSKSM.....GPVVIIR
Pahal.A00287.1.pha.32525941                  358  LDSEK....................DIAG..NILL.TGVQGKSNVDKS.............FSRSM.....GPVVIVR
OAV97825.1                                   481  FEAEQF.......GPGASEDKCGSTAIEA..RISK.IKEEMGPNLDSAS.......KQPPSDAGDG.....TPTAPDA
XP_003293253.1                               434  FDQNK....................GITN..NPIE...........................................
XP_005713144.1                               355  LDADR....................KMDPSLNPLT...DEVLTNLE................................
XP_005702831.1                               426  LDYLR....................DISQ.LNPLT..............................................
XP_009857252.1                               442  FEEDF....................GSDF..NATL.KIDERVEDLRAA...............GRLQPAVSAPGIKKE
GES64941.1                                   393  LDSEM....................GGNA..DGFS.KIEERVDELRGK...............GWLQPPVTGPVSVKK
KEY79445.1                                   444  LDGEM....................GGNA..DGYS.KIEDRVEELRGK...............GWLQPPVTGPVSVKK
XP_008869116.1                               334  LDKQSF......DDPSL........GIEP...ILN.DVDATKSDRDR..................................
Q66I22                                       562  LDDREKLWSQKPREETPTLELPAQNPILK..NITDYLIEEVSAEEEELLGSAGGGDSEDGSKEGNP.....TEITVER
RXN03320.1                                   502  LDDRERLWSHKPREET....LTLENPILK..NITDYLIEEVSAEEEELLGSAGGADSEEGSKEGNP.....TEITVER
XP_015195966.1                               455  ....................LPAQNPILK..NITDYLIEEVSAEEEELLGSAGGGEPEEGVKEGNP.....AEITVER
B1H1X4                                       523  LDERAVLWEGE.......QMVLAQNPILK..NITDYLIDEVNAEEEELLFSAGRTPETELPKDGNP.....TEISVER
NP_001362205.1                               520  LDERALLWEGEQGD....QMVSAQNPILK..NITDYLIDEVNAEEEELLFSAGRTPETETPKEGNP.....TEISVER
Q99MR6                                       530  LDDRTQLWASEPGTPPVPTSLPSQNPILK..NITDYLIEEVSAEEEELLGSSGGPPPEEPPKEGNP.....AEINVER
Q9BXP5                                       531  LDDRTQLWASEPGTPPLPTSLPSQNPILK..NITDYLIEEVSAEEEELLGSSGGAPPEEPPKEGNP.....AEINVER
XP_005549330.1                               531  LDDRTQLWASEPGTPPLPTSLPSQNPILK..NITDYLIEEVSAEEEELLGSSGGAPPEEPPKEGNP.....AEINVER
H2ZUZ4                                       498  LTSTNDLLLSLLFSPPLLKTLPSQNPILK..NITDYLIEEVSAEEEELLGNSSDA.AEEGPKEGNP.....AEINVER
XP_001748332.1                               441  LSLDKDVW.SDFCAPPDLKCRLGPVGLQQQ.......TGAMMRSSKRKS.............SMKGM.....NPAFITA
XP_002286542.1                               449  LDRVR....................EISQG.NRLV.........................................DLLKLLFP
XP_649316.1                                  319  FSREGA.......IVGKGKFRLSNVEIKSTLNISL...........................DDGGR............
consensus>70                                      ld.ek....................gi....nil....d......d.................gs......gpviiir
```

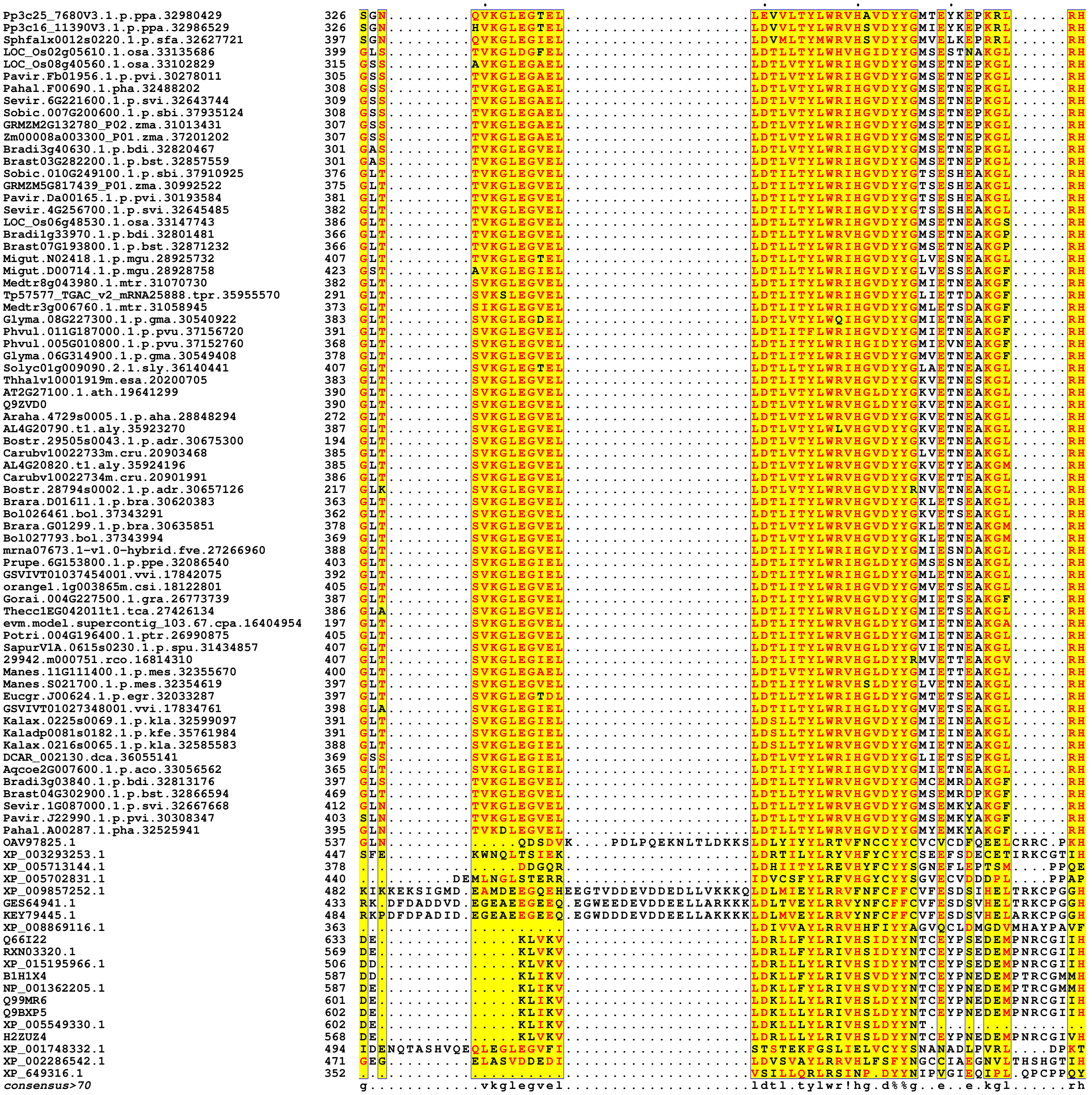
Pp3c25_7680V3.1.p.ppa.32980429 326
Pp3c16_11390V3.1.p.ppa.32986529 326
Sphfalx0012s0220.1.p.sfa.32627721 397
LOC_Os02g05610.1.osa.33135686 399
LOC_Os08g40560.1.osa.33102829 315
Pavir.Fb01956.1.p.pvi.30278011 305
Pahal.F00690.1.pha.32488202 308
Sevir.6G221600.1.p.svi.32643744 309
Sobic.007G200600.1.p.sbi.37935124 308
GRMZM2G132780_P02.zma.31013431 307
Zm00008a003300_P01.zma.37201202 307
Bradi3g40630.1.p.bdi.32820467 301
Brast03G282200.1.p.bst.32857559 301
Sobic.010G249100.1.p.sbi.37910925 376
GRMZM5G817439_P01.zma.30992522 375
Pavir.Da00165.1.p.pvi.30193584 381
Sevir.4G256700.1.p.svi.32645485 382
LOC_Os06g48530.1.osa.33147743 386
Bradi1g33970.1.p.bdi.32801481 366
Brast07G193800.1.p.bst.32871232 366
Migut.N02418.1.p.mgu.28925732 407
Migut.D00714.1.p.mgu.28928758 423
Medtr8g043980.1.mtr.31070730 382
Tp57577_TGAC_v2_mRNA25888.tpr.35955570 291
Medtr3g006760.1.mtr.31058945 373
Glyma.08G227300.1.p.gma.30540922 383
Phvul.011G187000.1.p.pvu.37156720 391
Phvul.005G010800.1.p.pvu.37152760 368
Glyma.06G314900.1.p.gma.30549408 378
Solyc01g009090.2.1.sly.36140441 407
Thhalv10001919m.esa.20200705 383
AT2G27100.1.ath.19641299 390
Q9ZVD0 390
Araha.4729s0005.1.p.aha.28848294 272
AL4G20790.t1.aly.35923270 387
Bostr.29505s0043.1.p.adr.30675300 194
Carubv10022733m.cru.20903468 385
AL4G20820.t1.aly.35924196 385
Carubv10022734m.cru.20901991 386
Bostr.28794s0002.1.p.adr.30657126 217
Brara.D01611.1.p.bra.30620383 363
Bol026461.bol.37343291 362
Brara.G01299.1.p.bra.30635851 378
Bol027793.bol.37343994 369
mrna07673.1-v1.0-hybrid.fve.27266960 388
Prupe.6G153800.1.p.ppe.32086540 403
GSVIVT01037454001.vvi.17842075 392
orange1.1g003865m.csi.18122801 405
Gorai.004G227500.1.gra.26773739 387
Thecc1EG042011t1.tca.27426134 386
evm.model.supercontig_103.67.cpa.16404954 197
Potri.004G196400.1.ptr.26990875 405
SapurV1A.0615s0230.1.p.spu.31434857 407
29942.m000751.rco.16814310 407
Manes.11G111400.1.p.mes.32355670 400
Manes.S021700.1.p.mes.32354619 397
Eucgr.J00624.1.p.egr.32033287 397
GSVIVT01027348001.vvi.17834761 398
Kalax.0225s0069.1.p.kla.32599097 391
Kaladp0081s0182.1.p.kfe.35761984 391
Kalax.0216s0065.1.p.kla.32585583 388
DCAR_002130.dca.36055141 369
Aqcoe2G007600.1.p.aco.33056562 365
Bradi3g03840.1.p.bdi.32813176 397
Brast04G302900.1.p.bst.32866594 469
Sevir.1G087000.1.p.svi.32667668 412
Pavir.J22990.1.p.pvi.30308347 403
Pahal.A00287.1.p.pha.32525941 395
OAV97825.1 537
XP_003293253.1 447
XP_005713144.1 378
XP_005702831.1 440
XP_009857252.1 482
GES64941.1 433
KEY79445.1 484
XP_008869116.1 363
Q66I22 633
RXN03320.1 569
XP_015195966.1 506
B1H1X4 587
NP_001362205.1 587
Q99MR6 601
Q9BXP5 602
XP_005549330.1 602
H2ZUZ4 568
XP_001748332.1 494
XP_002286542.1 471
XP_649316.1 352
consensus>70
g..........vkglegvel....................ldtl.tylwr!hg.d%%g..e..e.kgl......rh

```
Pp3c25_7680V3.1.p.ppa.32980429               369  IRPPEAK.............................................NSGDPKNNAEE......AGFADWEKK
Pp3c16_11390V3.1.p.ppa.32986529              369  IRAPEGK.............................................SGGDTKNGTEG......AGFADWEKK
Sphfalx0012s0220.1.p.sfa.32627721            440  IR..................................................GTVDPKNVGED......PGFGEWEKK
LOC_Os02g05610.1.osa.33135686                442  VR..................................................ADTKNAN.MDK......SSAADWEKK
LOC_Os08g40560.1.osa.33102829                358  VK..................................................ADSRTFN.GAS......SNAAEWEKK
Pavir.Fb01956.1.p.pvi.30278011               348  VK..................................................ADARTYN.GDS......SNATEWENK
Pahal.F00690.1.pha.32488202                  351  VK..................................................ADARTYN.GAS......SNATEWENK
Sevir.6G221600.1.p.svi.32643744              352  VK..................................................ADARTYN.GDS......SNATEWENK
Sobic.007G200600.1.p.sbi.37935124            351  VK..................................................ADARTYN.GAS......SNGAEWENK
GRMZM2G132780_P02.zma.31013431               350  VK..................................................ADARTYN.GAS......SNGAEWENK
Zm00008a003300_P01.zma.37201202              350  VK..................................................ADARTYN.GAS......SNGAEWENK
Bradi3g40630.1.p.bdi.32820467                344  VK..................................................SDSRTYD.GAS......SNAAEWEEN
Brast03G282200.1.p.bst.32857559              344  VK..................................................SDSRTYD.GAS......SNAAEWEEK
Sobic.010G249100.1.p.sbi.37910925            419  VR..................................................VDSKTSS.TSD......VSAADWEKK
GRMZM5G817439_P01.zma.30992522               418  VR..................................................VDSKTSS.TSD......VSSADWEKK
Pavir.Da00165.1.p.pvi.30193584               424  VR..................................................VDNKTSS.TSD......VNAADWEKK
Sevir.4G256700.1.p.svi.32645485              425  VR..................................................VDNKTSS.TSD......VNAADWEKK
LOC_Os06g48530.1.osa.33147743                429  VR..................................................ADNKTSN.TTN......INAADWEKK
Bradi1g33970.1.p.bdi.32801481                409  VR..................................................ADSKNSS.TIN......VNAVDWEKK
Brast07G193800.1.p.bst.32871232              409  VR..................................................ADSKTSS.AAN......VNAADWEKQ
Migut.N02418.1.p.mgu.28925732                450  VR..................................................VEVKNSD.AIA.......NANDWENK
Migut.D00714.1.p.mgu.28928758                466  VR..................................................VDGKNSD.STA.......NANEWENK
Medtr8g043980.1.mtr.31070730                 425  VR..................................................PERKGHE.ETG......KSGSEWEKK
Tp57577_TGAC_v2_mRNA25888.tpr.35955570       334  VR..................................................PERTGHA.ETG......NSGSEWEKK
Medtr3g006760.1.mtr.31058945                 416  VR..................................................PEGARHE.ETA......KSGSEWEKK
Glyma.08G227300.1.p.gma.30540922             426  VR..................................................PEGTAHEVVAG......KSGSEWEKK
Phvul.011G187000.1.p.pvu.37156720            434  VR..................................................PEGTGHE.ETG......KSGSDWEKK
Phvul.005G010800.1.p.pvu.37152760            411  VR..................................................PEGAGHE.ETS......KSGSEWEKK
Glyma.06G314900.1.p.gma.30549408             421  VR..................................................PEGAGHE.ETS......KSGSDWEKK
Solyc01g009090.2.1.sly.36140441              450  VR..................................................VDGKAAD.ATS.......NGAEWESK
Thhalv10001919m.esa.20200705                 426  VR..................................................AEGKGSD..........AEGDESESK
AT2G27100.1.ath.19641299                     433  VR..................................................AEGKVSD..........AKGDENESK
Q9ZVD0                                       433  VR..................................................AEGKVSD..........AKGDENESK
Araha.4729s0005.1.p.aha.28848294             315  VR..................................................AEGKVSD..........AKGDESESK
AL4G20790.t1.aly.35923270                    430  VR..................................................AEGKGSD..........AKGDESESK
Bostr.29505s0043.1.p.adr.30675300            237  VR..................................................AEGKGSD..........AKEDESESK
Carubv10022733m.cru.20903468                 428  VR..................................................AEGKGSD..........AKGDESESK
AL4G20820.t1.aly.35924196                    428  VR..................................................AEGKGSD..........AKGDESESK
Carubv10022734m.cru.20901991                 429  VR..................................................AEGKSSD..........AKGDENESK
Bostr.28794s0002.1.p.adr.30657126            260  VR..................................................AEGKGSD..........AKGDASESK
Brara.D01611.1.p.bra.30620383                406  VR..................................................AEGKGSD..........AKGDEGEDK
Bol026461.bol.37343291                       405  VR..................................................AEGKGSD..........AKGDEGEDK
Brara.G01299.1.p.bra.30635851                421  VR..................................................AEGKGSD..........AKGDESENK
Bol027793.bol.37343994                       412  VR..................................................AEGKGSD..........AKGDESENK
mrna07673.1-v1.0-hybrid.fve.27266960         431  VR..................................................VEVKGSD.IG........NGAEWEKK
Prupe.6G153800.1.p.ppe.32086540              446  VR..................................................AEGKSSD.IIS.......NGAEWEKK
GSVIVT01037454001.vvi.17842075               435  VR..................................................VEGKSSD.LTS.......NGVEWEKK
orange1.1g003865m.csi.18122801               448  VR..................................................AEGKSSD.ITN.......NGAEWEKK
Gorai.004G227500.1.gra.26773739              430  VR..................................................AEGKISD.ITS.......NGSEWEKK
Thecc1EG042011t1.tca.27426134                429  VR..................................................AEGKNSD.VTN.......NGSEWEKK
evm.model.supercontig_103.67.cpa.16404954    240  VR..................................................AEGKSSE.TT........SGAEWEKK
Potri.004G196400.1.ptr.26990875              448  VR..................................................TEGKSSD.SSN.......SGTEWEKK
SapurV1A.0615s0230.1.p.spu.31434857          450  VR..................................................AEGKSSD.SSN.......SGTEWEKK
29942.m000751.rco.16814310                   450  VR..................................................AEGKSSD.VNN.......SGMEWEKK
Manes.11G111400.1.p.mes.32355670             443  VR..................................................AEGKSAD.VTN.......NGNEWEKK
Manes.S021700.1.p.mes.32354619               440  VR..................................................AEGKNAD.ATD.......SGNEWEKK
Eucgr.J00624.1.p.egr.32033287                440  VR..................................................AEGKSAD.SGN.......NGSDWENK
GSVIVT01027348001.vvi.17834761               441  VR..................................................AEGKGHE.ENS......KAGADWEKK
Kalax.0225s0069.1.p.kla.32599097             434  VR..................................................GEAKGAD.ASN.......NAAEWEKT
Kaladp0081s0182.1.p.kfe.35761984             434  VR..................................................GEAKSAD.ASN.......NAAEWEKT
Kalax.0216s0065.1.p.kla.32585583             431  VR..................................................GEAKSDD.ATN.......NSTEWEKT
DCAR_002130.dca.36055141                     412  VR..................................................ADGKNSD.AIG.......NGDEWEKK
Aqcoe2G007600.1.p.aco.33056562               408  VR..................................................MEGKNYD.DTN......TAGADWEKK
Bradi3g03840.1.p.bdi.32813176                440  VR..................................................DDNKSTI.AFN......ISAADWEKK
Brast04G302900.1.p.bst.32866594              512  VR..................................................DDNKSIS.AFN......ISAADWERK
Sevir.1G087000.1.p.svi.32667668              455  VR..................................................AENKSGTMAEN......NNAADWEKK
Pavir.J22990.1.p.pvi.30308347                446  VR..................................................ADNKSGSMVEN......INAADWERK
Pahal.A00287.1.pha.32525941                  438  VR..................................................AENKSGSMVEN......INAADWEKK
OAV97825.1                                   596  VRKAAPR.........................QQLEDHSSTATPQSHRRN..TNAQNST.HLR......SNEQNWART
XP_003293253.1                               496  LRRSPGETTSISIEKKEEPTTTTTTTTSTTNDEQMETENNKSTEQIEKIKKNSEDENNN.LDNKPKLTTESEQLWVTS
XP_005713144.1                               415  LR..................................................PRADRGR.HMS......EADNRLLRR
XP_005702831.1                               484  LR..................................................PPKKDNN.MSE......KTGAEW..R
XP_009857252.1                               559  LRRPRSTLSSSA.KAVARASALGEPFPSK.KRKDAEDVEEGEAP........EGERKFR.TSSKTEQQLQRAYNWVKT
GES64941.1                                   508  LRRPRAGLTSQA.KDVARASALGQPFPTK.KKEPSEEGE..QSPA.......EEKRPQR.FTSKADQQLQRAFNWVRT
KEY79445.1                                   560  LRRPRAGLTSQS.KAVARASALGQPFPVK.KKEPSEEGEE.QPPA.......EEKRTHR.L.SKAEQQLQRAFNWVRT
XP_008869116.1                               399  VRPAATE.........................RDIEDDKTV....THKVF..SPTPSTE.TSG......AGWGAWSVS
Q66I22                                       676  VR..................................................GPIPPNR.ITL......REVSDWQKT
RXN03320.1                                   612  VR..................................................GPIPPNR.ITH......REVGDWQKT
XP_015195966.1                               549  VR..................................................GPIPPNR.ITH......GEVAEWQKT
B1H1X4                                       630  VR..................................................GPLPPNR.VSH......GEVAEWQKT
NP_001362205.1                               630  VR..................................................GPLPPNR.VSH......GEVAEWQKT
Q99MR6                                       644  VR..................................................GPMPPNR.ISH......GEVLEWQKT
Q9BXP5                                       645  VR..................................................GPMPPNR.ISH......GEVLEWQKT
XP_005549330.1                               628  .......................................................................LLEWQKT
H2ZUZ4                                       611  VR..................................................GPMPPNR.ITH......GEVAEWQKT
XP_001748332.1                               548  LKKA................................................PKVSKNR.AKSGAQLTVAHIVTWLKS
XP_002286542.1                               520  LRLRNADDILRK.AAEGRGIGVG........GGAMDEGEEEGGE........VETAPTT.VPNRLNSSIAKALEHVKM
XP_649316.1                                  386  IE..................................................QPVGNVP.IQS......SNGFEIPSS
consensus>70                                      vr...................................................e...................ewe.k
```

```
                                                  .           .              .             .         .        .
Pp3c25_7680V3.1.p.ppa.32980429                396 LDTTWQTRL.ESG....DIIVSMLG........REKLDTTANEAL.DPFVRKIRDEKYGWKYGCGAKGCTKLFHGPEF
Pp3c16_11390V3.1.p.ppa.32986529               396 LDTTWQTRL.QGG....DIIVSMLG........REKLDTTANEAL.DPFVRKIRDEKYGWKYGCGAKGCTKLFHGPEF
Sphfalx0012s0220.1.p.sfa.32627721             462 LESMWQARL.QSG....DLIEKMLG........REKLDSTANEAL.DPFVRKIRDEKYGWKYGCGAKGCTKLFHGPEF
LOC_Os02g05610.1.osa.33135686                 463 LDYFWQERLTNGK....DPLVALTA........KDKIDASADKVL.ESYVTKVKDDNYGWTYGCGAKGCIKVFHAPDF
LOC_Os08g40560.1.osa.33102829                 379 LDSFWQDRI.QGQ....DPMEILKA........KDKIDAAASEVL.DPHVRKIRDEKYGWKYGCGAKGCTKLFHAPEF
Pavir.Fb01956.1.p.pvi.30278011                369 LDSFWQNRI.HGQ....DPLEILKA........KDKIDAAATEVL.DPYVRKIRDEKYGWKYGCGAKGCTKLFHAAEF
Pahal.F00690.1.pha.32488202                   372 LDSFWQDRI.QGQ....DPLEILKA........KEKIDAAATEVL.DPYVRKIRDEKYGWKYGCGAKGCTKLFHAAEF
Sevir.6G221600.1.p.svi.32643744               373 LDSFWQDRI.QGQ....DPLEILKA........KEKIDAAATEVL.DPYVRKIRDEKYGWKYGCGAKGCTKLFHAAEF
Sobic.007G200600.1.p.sbi.37935124             372 LDSFWQDRI.QGQ....DPLEMLKA........KEKIDGAATEVL.DPYVRKIRDEKYGWKYGCGAKGCTKLFHAAEF
GRMZM2G132780_P02.zma.31013431                371 LDSFWQDRI.QGQ....DPLEMLKG........KEKIDGAATEVL.DPYVRKIRDEKYGWKYGCGAKGCTKLFHAAEF
Zm00008a003300_P01.zma.37201202               371 LDSFWQDRI.QGQ....DPLEMLKG........KEKIDGAATEVL.DPYVRKIRDEKYGWKYGCGAKGCTKLFHAAEF
Bradi3g40630.1.p.bdi.32820467                 365 LDSFWQDRI.QGQ....DPLEILKA........KEKIDTAASEVL.DPYVRKIRDEKYGWKYGCGAKGCTKLFHASEF
Brast03G282200.1.p.bst.32857559               365 LDSFWQDRI.QGQ....DPLEILKA........KEKIDTAASEVL.DPYVRKIRDEKYGWKYGCGAKGCTKLFHASEF
Sobic.010G249100.1.p.sbi.37910925             440 LDTFWQERL.NGQ....DPLVILTA........KDKIDAAAAEVL.EPLVRKIRDEKYGWKYGCGAKGCTKLFHAPEF
GRMZM5G817439_P01.zma.30992522                439 LDTFWQERL.NGQ....DQLVILTA........KDKIDAAAAEVL.EPLVRKIRDEKYGWKYGCGAKGCTKLFHAPEF
Pavir.Da00165.1.p.pvi.30193584                445 LDTFWQERL.NGQ....DPLVTLTA........KDKIDAAAVEVL.EPYVRKIRDEKYGWKYGCGAKGCTKLFHAPEF
Sevir.4G256700.1.p.svi.32645485               446 LDTFWQERL.NGQ....DPLVILTA........KDKIDAAAVEVL.EPYVRKIRDEKYGWKYGCGAKGCTKLFHAPEF
LOC_Os06g48530.1.osa.33147743                 450 VDTFWQERL.RGQ....DPMVILAA........KDKIDAAAVEVL.EPYVRKIRDEKYGWKYGCGAKGCTKLFHAPEF
Bradi1g33970.1.p.bdi.32801481                 430 LDTFWQERL.SGQ....DPMVILTA........KDKIDAAAAEVL.EPHVRKIRDEKYGWKYGCGAKGCTKLFHAPEF
Brast07G193800.1.p.bst.32871232               430 LDTFWQERL.SGQ....DPMVILTA........KDKIDAAAAEVL.EPHVRKIRDEKYGWKYGCGAKGCTKLFHAPEF
Migut.N02418.1.p.mgu.28925732                 470 LNSHWQERL.KGS....DPLEIMTG........KEKIDAAANEAL.NPHVRKIRDEKYGWKFGCGAKGCTKLFHAAEF
Migut.D00714.1.p.mgu.28928758                 486 LDSHWQERL.KGS....DPLEVMSA........KEKIDAVAAEAL.DPHVRKIRDEKYGWKFGCGAKGCTKLFHASEF
Medtr8g043980.1.mtr.31070730                  446 LDSFWQGRL.NGQ....DPLEVLAA........KEKMDAAAIEVL.DPYVRKIRDEKYGWKYGCGAKGCTKLFHAAEF
Tp57577_TGAC_v2_mRNA25888.tpr.35955570        355 LDSFWQGRL.NGQ....DPLEVMAA........KEKIEAAAVEVL.DPYIRKIRDEKYGWKYGCGAKGCTKLFHAAEF
Medtr3g006760.1.mtr.31058945                  437 LDSFWQRRL.DGL....DPLEVRTA........KEKIDAAAADML.DPYVRKIRDEKYGWKYGCGAKSCTKLFHAVEF
Glyma.08G227300.1.p.gma.30540922              448 LDLFWQGRL.NGQ....DPLEVMTA........KEKIDAAAMEVL.DPYVRKIRDEKYGWKYGCGAMGCTKLFHATEF
Phvul.011G187000.1.p.pvu.37156720             455 LDLFWQGRL.NGL....DPLEIMTA........KEKIDAAAMEVL.DPYVRKIRDEKYGWKYGCGAKGCTKLFHAAEF
Phvul.005G010800.1.p.pvu.37152760             432 LDQFWQGRL.NGQ....DPLELMSA........KEKIDAAAADVL.DPYVRKIRDEKYGWKYGCGAKGCTKLFHAAEF
Glyma.06G314900.1.p.gma.30549408              442 LDSFWHGRL.NGQ....DPLEVMTA........KEKIDAAATDVL.DPHVRKIRDEKYGWKYGCGAKGCTKLFHAAEF
Solyc01g009090.2.1.sly.36140441               470 VDSHWQDRL.KGK....DPLEVMAA........KEKLDAVAAEAL.DPYVRKIRDEKYGWKYGCGAKGCTKLFHASEF
Thhalv10001919m.esa.20200705                  444 FDSHWQERL.KGQ....DPLEVMAA........KEKIDAAALEVL.DPHVRKIRDEKYGWKYGCGAKGCTKLFHAAEF
AT2G27100.1.ath.19641299                      451 FDSHWQERL.KGQ....DPLEVMAA........KEKIDAAATEAL.DPHVRKIRDEKYGWKYGCGAKGCTKLFHAAEF
Q9ZVD0                                        451 FDSHWQERL.KGQ....DPLEVMAA........KEKIDAAATEAL.DPHVRKIRDEKYGWKYGCGAKGCTKLFHAAEF
Araha.4729s0005.1.p.aha.28848294              333 FDSHWQERL.KGQ....DPLEVMAA........KEKIDAAATEAL.DPHVRKIRDEKYGWKYGCGAKGCTKLFHAAEF
AL4G20790.t1.aly.35923270                     448 FDSHWQERL.KGQ....DPLEVMAA........KEKIDAAATEAL.DPHVRKIRDEKYGWKYGCGAKGCTKLFHAAEF
Bostr.29505s0043.1.p.adr.30675300             255 FDSHWQERL.KAQ....DPLEVMAA........KEKIDAAATEVL.DPHVRKIRDEKYGWKYGCGAKGCTKLFHAAEF
Carubv10022733m.cru.20903468                  446 FDSHWQERL.KGQ....DPLEVMAA........KEKIDAAATEAL.DPHVRKIRDEKYGWKYGCGAKGCTKLFHAAEF
AL4G20820.t1.aly.35924196                     446 FDSHWQERL.KGQ....DPLEVMAA........KEKIDAAATEAL.DPHVRKIRDEKYGWKYGCGAKGCTKLFHAAEF
Carubv10022734m.cru.20901991                  447 FDSHWQERL.KGQ....DPLEVMAA........KEKIDAAATEAL.DPHVRKIRDEKYGWKYGCGAKGCTKLFHAAEF
Bostr.28794s0002.1.p.adr.30657126             278 FDSHWQERL.KGQ....DPLEVMAA........KEKIDAAATEAL.DPHVRKIRDEKYGWKYGCGAKGCTKLFHAAEF
Brara.D01611.1.p.bra.30620383                 424 FDSHWQQRL.KGQ....DPLEVMAA........KEKLDAAAIEAL.DPHVRKIRDEKYGWKYGCGAKGCTKLFHAAEF
Bol026461.bol.37343291                        423 FDSHWQQRL.KGQ....DPLEVMAA........KEKLDAAAIEAL.DPHVRKIRDEKYGWKYGCGAKGCTKLFHAAEF
Brara.G01299.1.p.bra.30635851                 439 FDSHWQERL.KGP....DPLEVMAA........KEKLEAAAIEAL.DPH.............................
Bol027793.bol.37343994                        430 FDSHWQERL.KGQ....DPLEVMAA........KEKLEAAAIEAL.DPHVRKIRDEKYGWKYGCGAKGCTKLFHAAEF
mrna07673.1-v1.0-hybrid.fve.27266960          450 LDLHWQERL.HSQ....DPLEVMTA........KEKIEAAALQGL.DPFVRKIRDEKYGWKYGCGAKGCTKLFHAAEF
Prupe.6G153800.1.p.ppe.32086540               466 MDLRWQERL.QSQ....DPLEIMTA........KEKIDATASESL.DPYVRKIRDEKYGWKYGCGAKGCTKLFHAAEF
GSVIVT01037454001.vvi.17842075                455 FDSHWQERL.RSQ....DPLEIMTA........KEKIDATAIESL.DPYVRKIRDEKYGWKYGCGAKGCTKLFHAAEF
orange1.1g003865m.csi.18122801                468 LDFRWQERL.RSQ....DPLEIMTA........KDKIDAAAVDAL.DPFVRKIRDEKYGWKYGCGAKGCTKLFHAAEF
Gorai.004G227500.1.gra.26773739               450 LDSYWQDRL.RGQ....DPLEVMTA........KDKLDAAAVESL.DPFVRKIRDEKYGWKYGCGAKGCTKLFHAAEF
Thecc1EG042011t1.tca.27426134                 449 LDSRWQERL.RGQ....DPLVLMTA........KDKIDAAAVEAL.DPYVRKIRDEKYGWKYGCGAKGCTKLFHAAEF
evm.model.supercontig_103.67.cpa.16404954     259 LDSRWQERL.RGQ....DPLEVMTC........KEKIDAAAVEAL.DPYVRKIRDEKYGWKFGCGAKGCTKLFHAAEF
Potri.004G196400.1.ptr.26990875               468 LDLRWQERL.RGQ....DPLEEMTA........KEKIDAAAVEGL.DPYVRKIRDEKYGWKYGCGAKGCTKLFHASEF
SapurV1A.0615s0230.1.p.spu.31434857           470 LDFCWQERL.RGQ....DPLEKMTA........KEKIDATAVEGL.DPYVRKIRDEKYGWKYGCGAKGCTKLFHAAEF
29942.m000751.rco.16814310                    470 LDSRWQERL.RSQ....DPLEVMTA........KEKIEATAVESL.DPYVRKIRDEKYGWKYGCGAKGCTKLFHAAEF
Manes.11G111400.1.p.mes.32355670              463 LDTHWQERL.RSQ....DSLEVMTA........KEKIDAAAVESL.DPYVRKIRDEKYGWKYGCGAKGCTKLFHAAEF
Manes.S021700.1.p.mes.32354619                460 LDSHWQERL.RSQ....DPFELMTA........KEKIDAAAVESL.DPYVRKIRDEKYGWKYGCGAKGCTKLFHAAEF
Eucgr.J00624.1.p.egr.32033287                 460 LDSRWQERL.KGQ....DPLEVMTA........KEKIDAAAVEVL.DPYVRKIRDEKYGWKYGCGAKGCTKLFHAAEF
GSVIVT01027348001.vvi.17834761                462 LDSRWQDRL.RGQ....DPLETMAA........KEKIDAAAVEGL.DPYVRKIRDERYGWKYGCGAKGCTKLFHAAEF
Kalax.0225s0069.1.p.kla.32599097              454 LDSHWQDRL.LGQ....DPLEIMTA........KDKIEAAAVEAL.DPYVRKIRDEKYGWKYGCGAKGCTKLFHAAEF
Kaladp0081s0182.1.p.kfe.35761984              454 LDSHWQDRL.LGQ....DPLEIMTA........KDKIEAAAVEAL.DPYVRKIRDEKYGWKYGCGAKGCTKLFHAAEF
Kalax.0216s0065.1.p.kla.32585583              451 LDSHWQDRL.SGQ....DPLEIMTA........KDKIEAAAVEAL.DPYVRKIRDEKYGWKYGCGAKGCTKLFHAAEF
DCAR_002130.dca.36055141                      432 LDSFWKERL.NGQ....DPLEVMTA........KEKIDTAAGESL.DPYVRKIRDEKYGWKYGCGAKGCTKLFHAAEF
Aqcoe2G007600.1.p.aco.33056562                429 LDSFLQGRL.QGR....DPLEIMTA........KEKIETAAGEVL.DPYVRKIRDEKYGWKYGCGAKGCTKLFHASEF
Bradi3g03840.1.p.bdi.32813176                 461 LDSFWHERLMNGK....DPLVVLAS........QDKIDAATVETL.EPYVKKIMDDKFSWKYGCGALGCIKIFHAPEF
Brast04G302900.1.p.bst.32866594               533 LDSFWHGRLMNGK....DPLVVLAS........QDKIDAATVETL.EPYVKKIQDEKFLWRYGCGALGCIKFFHAPEF
Sevir.1G087000.1.p.svi.32667668               477 LDSFWEQRLVNGE....DPLVVLTA........KDKIDAAIVEVL.EPYVRKMRDEKYVWKYGCGAKGCEKLFHAPEY
Pavir.J22990.1.p.pvi.30308347                 468 LDSFWEERLMNGE....DPLVVLTA........KDRIGAAIAEVL.ETYVRKMRDKKYVWKYGCGANGCEKLFHAPEY
Pahal.A00287.1.pha.32525941                   460 LDSFWEERLMNGG....DPLVVLTA........KDKIGAAIVEVL.ETYVRKLQDEKYVWKYGCGANGCEKLFHAPEY
OAV97825.1                                    640 LDDRVALIL........TPSEVDPR.EF....GGERVDDEIHRLC.QP....QIIDEGAGKFRCKS..CSKLFKAMNF
XP_003293253.1                                573 LDNNTKNKIARIP....TSNEQYLC........KEAIEKAAEDYI.NNNTIKIEEE....KYKCNL..CSKLFKGAEY
XP_005713144.1                                436 VDERARWVL....E..RDYDRPRSN........SDNGEAAKEAAL.QKWFKANTKHEAEGRYRCNLPP.HKLFKGPEF
XP_005702831.1                                503 VDEPVNAIL....Q..RNYDHPKGTV.......SIDSESLREERI.AEWLEENTKYEGQGRYRCSLPP.FKLFQGKEY
XP_009857252.1                                626 FEDKIMQIL........EPDTVDIR.KL....GGKPVEDAINDEL.AKYVKQ.EDEN...KWRCKVPECTKLFKEEHF
GES64941.1                                    574 FEDKLLQIL........EPENVDIR.KL....GGKPVEEALEEEL.SKYVKQ.EDES...KYRCKVPECTKLFKADHF
KEY79445.1                                    626 FEDKLLQIL........EPDNVDIR.KL....GGKPVEEALEEEL.SKFVKQ.EDEA...KFRCKVPECTKLFKAEHF
XP_008869116.1                                439 LDEKIEAFI.KAN....APSVVEAK.RVAELAMVQDIETREEVAL.EPYTEKAGDD...GKHRCLL..CTKLFKSLEF
Q66I22                                        697 FEEKMGPLF.SVK....ENLSEDEA........AKMGRKDPEQEV.EKFVVANTQELGKDKWLCPLSG..KKFKGPEF
RXN03320.1                                    633 FEEKLGPLF.SVK....ETLSEDEA........AKMGRKDPEQEV.EKFVQANTQELGKDKWLCPLSG..KKFKGPEF
XP_015195966.1                                570 FEEKLTPLF.NVR....ESLSEEEA........VKMGRKVPEQEV.EKFVSANTQELGKDKWLCPLSG..KKFKGPEF
B1H1X4                                        651 FEEKLAPLF.AVR....ETLSEEES........IKMGKKDPEQEV.EKFVAANTQELGKEKWLCPLSG..KKFKGPEF
NP_001362205.1                                651 FEEKLSPLF.AVR....ETLSEEEA........VKMGKKDPEQEV.EKFVTANTQELGKEKWLCPLSG..KKFKGPEF
Q99MR6                                        665 FEEKLTPLL.SVR....ESLSEEEA........QKMGRKDPEQEV.EKFVTSNTQELGKDKWLCPLSG..KKFKGPEF
Q9BXP5                                        666 FEEKLTPLL.SVR....ESLSEEEA........QKMGRKDPEQEV.EKFVTSNTQELGKDKWLCPLSG..KKFKGPEF
XP_005549330.1                                635 FEEKLTPLL.SVR....ESLSEEEA........QKMGRKDPEQEV.EKFVTSNTQELGKDKWLCPLSG..KKFKGPEF
H2ZUZ4                                        632 FEDKLTPLF.SVR....ETLSEDEA........QKMGKKDPEQEV.EKFVTANTQELGKDKWLCPLSG..KKFK....
XP_001748332.1                                577 FPQHRQQIM.RAIDKLRRRFEAEAA........GIDVEAEAAQQAAQPLIPEPEEGKAIGVIGRNINGVIRLFDEEDD
XP_002286542.1                                580 LEERGPTVL........VDEETDAA........AKAIEENEQNAK.MNWIENHGLVDEDGRARCSFHFCRKLFKDRAF
XP_649316.1                                   407 LE...........V..ENPSNPSKP....................KKNFCVETPKSKYQLNYKCGY...IPLTHQQGY
consensus>70                                      .d..wq.rl........dpl..m.a........kekid.aa.e.l.dp.vrkirdekygwkygcgakgctklfha.ef
```

```
Pp3c25_7680V3.1.p.ppa.32980429              460  VHKHLKLKHSDLVADVVAKAREELYFQNYMGDP..........DAPGANQVTSSQPGQ.RDGDRG.RRGSQRP.GFSG
Pp3c16_11390V3.1.p.ppa.32986529             460  VHKHLKLKHSDLVADVVAKAREELYFQNYMSDP..........DAPGANQVTSSQPGQ.RDDDRG.RRG.LRP.GLSG
Sphfalx0012s0220.1.p.sfa.32627721           526  VHKHLKLKHSDLVSDVVAKAREELYFQNYMSDP..........EAPGTTQAVSSQPGQ.R.GDRG.RRG.RRP.GFSG
LOC_Os02g05610.1.osa.33135686               528  VLKHLNLKHPDLVSKLTSRVQEDIYFQNYMNDP..........NAPGGTPVMQ.QQSP....EQQ..........
LOC_Os08g40560.1.osa.33102829               443  VQKHLKLKHADLVRELTSKVRENIYFDNYMSDP..........KAPGGMPIMQ.QPAP...REKV..R..QRP.PIE.
Pavir.Fb01956.1.p.pvi.30278011              433  VQKHLKLKHADVVVELTSKVRDDIYFENYMNDP..........KAPGGTPIMQ.QPAP...RGKG..R..QRP.PIE.
Pahal.F00690.1.pha.32488202                 436  VQKHLKLKHPDVVVELTSKVRDDIYFENYMNDP..........KAPGGTPLMQ.QPAP...RGKG..R..QRP.PIE.
Sevir.6G221600.1.p.svi.32643744             437  VQKHLKLKHADVVVELTSKVREDIYFENYMNDP..........KAPGGTPIMQ.QPAP...RGKG..R..QRP.PIE.
Sobic.007G200600.1.p.sbi.37935124           436  VQKHLKLKHADLVVDLTSKAREDIYFENYMNDP..........KAPGGTPIMQ.QPAP...RGKG..R..QRP.PIE.
GRMZM2G132780_P02.zma.31013431              435  VQKHLKLKHADLVVDLTSKVREDIYFENYMNDP..........KAPGGTPIMQ.QPAP...RGKG..R..QRP.PIE.
Zm00008a003300_P01.zma.37201202             435  VQKHLKLKHADLVVDLTSKVREDIYFENYMNDP..........KAPGGTPIMQ.QPAP...RGKG..R..QRP.PIE.
Bradi3g40630.1.p.bdi.32820467               429  VTKHLKLKHTEFVVELTSKVRDDIYFENYMNDP..........KAPGGTPIMQ.QPAP...REKG..K..QRP.PIE.
Brast03G282200.1.p.bst.32857559             429  VTKHLKLKHTEFVVELTSKVRDDIYFENYMNDP..........KAPGGTPIMQ.QPAP...REKG..K..QRP.PIE.
Sobic.010G249100.1.p.sbi.37910925           504  VHKHLRLKHPDLVLESTSKVREDLYFQNYMNDP..........NAPGGTPVMQ.QSAP....DRG..R..RRP.SMD.
GRMZM5G817439_P01.zma.30992522              503  VHKHLKLKHPDLVLESTSKVREDLYFQNYMNDP..........NAPGGTPVMQ.QSAP....DRG..R..RRP.SMD.
Pavir.Da00165.1.p.pvi.30193584              509  VHKHLRLKHPDIVLESTSKVREDLYFQNYMDDP..........NAPGGTPVMQ.QSAP....DRG..R..RRP.GMD.
Sevir.4G256700.1.p.svi.32645485             510  VHKHLRLKHPELVLESTSKVREDLYFQNYMNDP..........NAPGGTPVMQ.QSAP....DRG..R..RKP.GMD.
LOC_Os06g48530.1.osa.33147743               514  VHKHLRLKHPELVLELTSKVREDLYFQNYMNDP..........NAPGGTPVMQ.QSAP....DKS..R..QRP.GMD.
Bradi1g33970.1.p.bdi.32801481               494  VYKHLRLKHPELVLEVTSKVREDLYFQNYMNDP..........NAPGGTPVMQ.QSAP....DKS..R..R...GMD.
Brast07G193800.1.p.bst.32871232             494  VYKHLRLKHPELVLEVTSKVREDLYFQNYMNDP..........NAPGGTPVMQ.QSAP....DKS..R..R...GMD.
Migut.N02418.1.p.mgu.28925732               534  VHKHLKLKHPELAVELTTKVREELYLQNYMKDE..........NAPGGKPVMQ.PSL....KEKL.LR..RRP.GPD.
Migut.D00714.1.p.mgu.28928758               550  VLKHLKLKHPELVMELTSKLREELYIENYMNDK..........NAPGGTPVMQ.PSFQ...KDKQ.LR..RRP.GLD.
Medtr8g043980.1.mtr.31070730                510  VYKHLKLKHPELALEQTSKLREDLYTQNYMNDP..........DAPGGKPVMQ.QPQK...DNRP.LN..QRL.GFE.
Tp57577_TGAC_v2_mRNA25888.tpr.35955570      419  VYKHLKLKHPELALEQTSKLREDLYTQNYMNDP..........DAPGGKPVMQ.QPQK...DNKP.LN..RRL.GME.
Medtr3g006760.1.mtr.31058945                501  VYKHLKLKHPELVLELTSKLREDLYFQNYMNDP..........DAPGGTPVMQ.QSQ.....DKP.LK..RRL..LE.
Glyma.08G227300.1.p.gma.30540922            512  VLKHLKLKHPELVVEQTSKVREDLYFQNYMNDK..........DAPGGMPIMQ.QPQ....KGRP.LK..RR..GLE.
Phvul.011G187000.1.p.pvu.37156720           519  VHKHLKLKHPELVMEQTTKVREDLYFQNYMNDP..........DAPGGTPIMQ.QSQ....RDRP.MK..RRL.GLE.
Phvul.005G010800.1.p.pvu.37152760           496  VYKHLKLKHPELVMELTSKLREDLYFQNYMNDP..........DAPGGTPVMQ.QPQ....KDRG.LK..RRLGGLE.
Glyma.06G314900.1.p.gma.30549408            506  VHKHLKLKHPEIVMELTSKMREDLYFQNYMNDP..........DAPGGMPVMQ.QPQ....KDRP.LK..RRLGGLE.
Solyc01g009090.2.1.sly.36140441             534  VHKHLKLKHPDLSVDLTTKVREDLYFQNYMNDE..........NSPGGTPVML.PSVP...KEKP.LR..RRP.GLD.
Thhalv10001919m.esa.20200705                508  VYKHLKLKHTELVVDLTGKVREELYFQNYTSDP..........NAPGGQPATQ.QSGP...RDRPMMR..RKP.GME.
AT2G27100.1.ath.19641299                    515  VYKHLKLKHTELVTELTTKVREELYFQNYMNDP..........NAPGGQPATQ.QSGP...RDRP.IR..RKP.SME.
Q9ZVD0                                      515  VYKHLKLKHTELVTELTTKVREELYFQNYMNDP..........NAPGGQPATQ.QSGP...RDRP.IR..RKP.SME.
Araha.4729s0005.1.p.aha.28848294            397  VYKHLKLKHTELVTELTTKVREELYFQNYMNDP..........NAPGGQPATQ.QSGP...RDRP.IR..RKP.SME.
AL4G20790.t1.aly.35923270                   512  VYKHLKLKHTELVTELTTKVREELYFQNYMNDP..........NAPGGQPATQ.QSGP...RDRP.IR..RKP.SME.
Bostr.29505s0043.1.p.adr.30675300           319  VYKHLKLKHTELVTELTTKVREELYFQNYMNDS..........NAPGGQPATQ.QSGP...RDRP.MR..RKP.GME.
Carubv10022733m.cru.20903468                510  VYKHLKLKHTELVTELTTKVREELYFQNYMNDP..........NAPGGQPATQ.QSGP...RDRP.MR..RKP.GME.
AL4G20820.t1.aly.35924196                   510  VYKHLKLKHTELVTELTTKVREELYLQNYMNDP..........NAPGGQPATQ.QSGP...RDRP.MR..RRP.GME.
Carubv10022734m.cru.20901991                511  VYKHLKLKHTELVTELTIKVREELYFQNYMNDP..........NAPGGQPAMQ.QSGP...RDRP.MR..RKP.SME.
Bostr.28794s0002.1.p.adr.30657126           342  VYKHLKLKHTELVTELTTKVQEELYFQNYMNDP..........NAPGGQPATQ.QSGP...KDRP.IR..RKP.GME.
Brara.D01611.1.p.bra.30620383               488  VHKHLKLKHAELIVDMTVKVREELYFQNYMNDP..........NAPGGQPATQ.QPGP...RDRPMMR..RKP.SME.
Bol026461.bol.37343291                      487  VHKHLKLKHAELIVDMTVKVREELYFQNYMNDP..........NAPGGQPATQ.QPGP...RDRPMMR..RKP.SME.
Brara.G01299.1.p.bra.30635851               474  ...........................................................RDRPMMR..RKP.GME.
Bol027793.bol.37343994                      494  VHKHLKLKHAELVVDQTAKVREELYFQNYMNDP..........NAPGGQPATQ.QPGP...RDRPMLR..RKP.GME.
mrna07673.1-v1.0-hybrid.fve.27266960        514  VHKHLKLKHPELLVDVTVKMREDLYFQNYMNDA..........EAPGGTPVMQ.QSIL...RDKP.QR..RRP.GME.
Prupe.6G153800.1.p.ppe.32086540             530  VHKHLKLKHPELVIEVTSKLREELYFQNYMNDA..........DAPGGTPIMQ.QPVL...REKP.QR..RRP.GVE.
GSVIVT01037454001.vvi.17842075              519  VHKHLKLKHPELVMELTSKVREDLYFLNYMSDA..........DAPGGTPVMQ.QSLL...KDKT.QK..RRL.GPD.
orange1.1g003865m.csi.18122801              532  VHKHLKLKHPELVIELTSKVREELYFQNYMNDP..........DAPGGTPVMQ.QPMP...KDRP.LR..RKL.GPD.
Gorai.004G227500.1.gra.26773739             514  VHKHLKLKHPELVIELTSKVREELYFQNYMNDP..........DAPGGTPVMQ.QPIP...KDKP.QR..RKV..ME.
Thecc1EG042011t1.tca.27426134               513  VHKHLKLKHPELVMELTSKVREELYFQNYMNDP..........DAPGGTPVMQ.QSVP...KDKP.QR..RKI..LE.
evm.model.supercontig_103.67.cpa.16404954   323  VHKHLKLKHPELVMELTVKVREELYFQNYMNDT..........DAPGGTPIMQ.QSVL...RDRP.LR..RKP.GSE.
Potri.004G196400.1.ptr.26990875             532  VQKHLKLKHPELVIDLTAKVREELYFQNYLNDP..........DAPGGTPVMQ.QSLP...KDKP.QR..RKL.GPE.
SapurV1A.0615s0230.1.p.spu.31434857         534  VQKHLKLKHPELLMELTAKVREELYFQNYLNDP..........DAPGGTPVMQ.QSLP...RDKP.QR..RKL.GPE.
29942.m000751.rco.16814310                  534  VHKHLKLKHPELVMELTSKLREDLYFQNYMNDP..........DAPGGTPVMQ.QPLP...KDKP.QR..RKL.GPE.
Manes.11G111400.1.p.mes.32355670            527  VHKHLKLKHPELVLELTSKVREELYFQNYMNDP..........VAPGGTPVMQ.QPLQ...RDKP.QR..RKL.GPE.
Manes.S021700.1.p.mes.32354619              524  VHKHLKLKHPELVMELTSKVREELYFQNYMSDP..........DAPGGTPVMQ.QPLQ...KEKP.QR..RKL.GPE.
Eucgr.J00624.1.p.egr.32033287               524  VHKHLKLKHPELVMELTAKLREELYFQNYMNDP..........DAPGGTPVMQ.QNLM...RDRP.QR..RRL.GLE.
GSVIVT01027348001.vvi.17834761              526  VHKHLKLKHPELVMDLTTKVREDLYFQNYMNDP..........DAPGGTPVMQ.QQLL...KDRL.QR..RRP.GLD.
Kalax.0225s0069.1.p.kla.32599097            518  VHKHLKLKHPELATDVTTKVRENLYFQNYMNDP..........NAPGGKPVMQ.QPSV...RDRI.QK..RKF.NLD.
Kaladp0081s0182.1.p.kfe.35761984            518  VHKHLKLKHPELATDVTTKVREELYFQNYMNDP..........NAPGGKPVMQ.QPSV...RDRI.QK..RKF.NLD.
Kalax.0216s0065.1.p.kla.32585583            515  VHKHLKLKHPELATDVTMKVREDLYFQNYMNDP..........NAPGGKPVMQ.QPSV...RDRI.QK..RKF.NLD.
DCAR_002130.dca.36055141                    496  VHKHLKLKHPELLLEVTSKVREDLFFQNYMGDE..........NAPGGIPVMQ.PTVL...KEKP.QK..RRL.GPE.
Aqcoe2G007600.1.p.aco.33056562              493  VHKHLRLKHPELVMELTLKLREDLYFQNYMNDA..........DAPGGTPIMQ.QPLP...KEKP.QR..RRP.VLE.
Bradi3g03840.1.p.bdi.32813176               526  VHRHLKLKHPELVSELTSKVQEDIYRQNYMNDP..........NAPGGIPVMQ.QSAP....NRDRIR..QIP.....
Brast04G302900.1.p.bst.32866594             598  VHRHLKLKHPDLVSELTSKVQEDIYRQNYMNDP..........NAPGGIPVMQ.QSAP...QNRDRIR..QIP.....
Sevir.1G087000.1.p.svi.32667668             542  VHKHLRLKHPLLVSILSSRIENDIYFQNYMNDP..........DAPGGKPVMQ.QTLQ....DRT..R..RRP.....
Pavir.J22990.1.p.pvi.30308347               533  VHKHLRLKHPGLVSILASRVENDIYFQNYMDDP..........DAPGGEPVMQ.KMEP....DRM..R..RRP.....
Pahal.A00287.1.pha.32525941                 525  VHKHLRLKHPDLVSTLASKVENNIYFQNYMDDP..........DAPGGEPVMQ.KMEP....DRM..R..RRP.....
OAV97825.1                                  698  IEKHIMSKHGEVINQD..SIERIKYLNNYILDPSH........TTPQAPPPPE............YG..HSR.GHNG
XP_003293253.1                              632  VKKHINLKHPEELKNESEKGTEEQFFLNYFSDP..........RRITPLPPPQ.QQPPNA........MYA.GFG.
XP_005713144.1                              498  VEKHIRTRHADKVKQVADKALMETYRANFENDA..........SKDEVVKIYH.EGKT...GGQEQEK..HMT.GKQG
XP_005702831.1                              566  VHKHLRTKHTEEMQKVIEKADKEQFIENYLNDP..........NHLDDSKVFD...........I.........
XP_009857252.1                              686  WKKHVEKRHNEWLDKLKE...EIALVNAYVIDPAHIAPSRTDANSNGHFPPSN.GQQP...TGTP..R......GFN.
GES64941.1                                  634  WRKHVEKRHTEWFENIKS...DLALVNAYVLDPARIAPSRSDANSNGHFPLAS.SQGQ...VGTP..R......GFS.
KEY79445.1                                  686  WRKHVEKRHTEWFENIKS...DLSLVNSYVLDPARIAPSRSDANSNGHFPLSS.SQNQ...TGTP..R......GFS.
XP_008869116.1                              505  VKKHIRNKHPELVVEKIVSAGESCMWDQYREDP..........DRPMDPVATT.NQPI........LR..QSS.GYD.
Q66I22                                      759  VRKHILNKHGDKIEEVKK...EVVFFNNFLMDA..........KRPCIPEMKP.PPHP....................
RXN03320.1                                  695  VRKHILNKHGDKIEEVKK...EVIFFNNFLMDA..........KRPSIPEMKP.PPPP....................
XP_015195966.1                              632  VRKHIFNKHGEKIEEVKK...EVLFFNNFLMDA..........KRPSLPEIKA.PPPP....................
B1H1X4                                      713  VRKHIFNKHAEKIEEVKK...EVVFFNNYLIDS..........KRPALLEVKP.LQPP....................
NP_001362205.1                              713  VRKHIFNKHGEKIEEVKK...EVA.FQTLPDRQ..........QRPALPEVEP.LRPP....................
Q99MR6                                      727  VRKHIFNKHAEKIEEVKK...EVAFFNNFLTDA..........KRPALPEIKP.AQPP....................
Q9BXP5                                      728  VRKHIFNKHAEKIEEVKK...EVAFFNNFLTDA..........KRPALPEIKP.AQPP....................
XP_005549330.1                              697  VRKHIFNKHAEKIEEVKK...EVAFFNNFLTDA..........KRPALPEIKP.AQPP....................
H2ZUZ4                                      690  VRRN.............S...QSLPFPKFSGGV..........TEDSATALPA.APPP....................
XP_001748332.1                              646  LEKHQAAMRARIAQAKLAAYNDDFYASREAVVP..........PPAQAPPVIE.DLDM...YDAVQSKD.AQF.GFGQ
XP_002286542.1                              641  LHKHLQKKHADHLRAECAKCHDHPMMIAWDGDE..........QRP................................
XP_649316.1                                 449  VVGMEPMKYKDFAALQPSKVQQE...SNNQNIS..........NFPGQMPFSTRQQLPPQIPSQP.IP..QEI.GYN.
consensus>70                                     v.khl.lkh.e.v.e...k..ed.yfqnymndp..........napgg.p..q.q.......d...........e.
```

```
Pp3c25_7680V3.1.p.ppa.32980429                  525  PV.GGPGNRAGADWLPSDH...PSRDNRFGPRTGERNEQGPGGREEE.RFDRGGRDEKA.........RFDRGGLELT
Pp3c16_11390V3.1.p.ppa.32986529                 524  PI.GGPGNRAGPDWPPSDH...PSRDGRFGPRNGDRYDQGPGAREEQ.RFERGTRDEKG.........RFDRGGLELT
Sphfalx0012s0220.1.p.sfa.32627721               589  PLPGGAVEPLPVDRLPPNTGVPPPRDGRFGP.......QGPGVREEEPRFDRGGREEEP.........RFDRGGPEHS
LOC_Os02g05610.1.osa.33135686                        ..............................................................................
LOC_Os08g40560.1.osa.33102829                   501  ..........................SRL...................RDERGNR.............RFDRN....D
Pavir.Fb01956.1.p.pvi.30278011                  491  ..........................SRL...................RDERGNR.............RFDTNV...D
Pahal.F00690.1.pha.32488202                     494  ..........................SRL...................RDERGNR.............RFDGNV...D
Sevir.6G221600.1.p.svi.32643744                 495  ..........................SRL...................RDERGNR.............RFDTNV...D
Sobic.007G200600.1.p.sbi.37935124               494  ..........................SRL...................RDERGNH.............RFDRNV...D
GRMZM2G132780_P02.zma.31013431                  493  ..........................SRL...................RDERGNH.............RFDRNV...D
Zm00008a003300_P01.zma.37201202                 493  ..........................SRL...................RDERGNH.............RFDRNV...D
Bradi3g40630.1.p.bdi.32820467                   487  ..........................SRL...................RDERG.R.............RFDRNA...D
Brast03G282200.1.p.bst.32857559                 487  ..........................SRL...................RDERG.R.............RFDRNA...D
Sobic.010G249100.1.p.sbi.37910925               561  ..........................NRL...................KFDRGSNREHD..KSERDGGRYGRGD...Q
GRMZM5G817439_P01.zma.30992522                  560  ..........................SRL...................KFDRGSNRDHD..KADRDGVRYGRGD...H
Pavir.Da00165.1.p.pvi.30193584                  566  ..........................SRL...................RFDRGNNRDND..KAE..GGQYGRGD...R
Sevir.4G256700.1.p.svi.32645485                 567  ..........................SRL...................RFDRGNNKDND..KAE..GGRYGRGD...R
LOC_Os06g48530.1.osa.33147743                   571  ..........................NRL...................RYDRANRREYD..RAERDGSRYGRGD...R
Bradi1g33970.1.p.bdi.32801481                   549  ..........................SRL...................RYDRTNRRDYD..RADRDGGRYGRGD...G
Brast07G193800.1.p.bst.32871232                 549  ..........................SRL...................RYDRTNRRDYD..RADRDGGRYGRGN...G
Migut.N02418.1.p.mgu.28925732                   592  ........................NNNRM...................NNERG...DRN..YRANGNEHFDRPE...N
Migut.D00714.1.p.mgu.28928758                   609  ..........................NRM...................KDDRGNQRERD..NRANGNDRFNRPE...N
Medtr8g043980.1.mtr.31070730                    569  ..........................GRL...................RDDRGNRRDHERYDRMNG....DRAN....
Tp57577_TGAC_v2_mRNA25888.tpr.35955570          478  ..........................GRL...................RDDRGNRRDHERNDRINDG...DRPD....
Medtr3g006760.1.mtr.31058945                    557  ..........................GRL...................KDDRGNRRDQDRSDRTNG....DRPD...S
Glyma.08G227300.1.p.gma.30540922                569  ..........................GRL...................RDDRGNRRDHDQSDRMNG....DRPD...N
Phvul.011G187000.1.p.pvu.37156720               577  ..........................GRL...................RDDRGNRRDHDRNDRMNG....DRPD...N
Phvul.005G010800.1.p.pvu.37152760               555  ..........................GRL...................KDDRGNR.....NEKMNG....DRDR...D
Glyma.06G314900.1.p.gma.30549408                565  ..........................GRL...................KDDRGNRRDQDRSDRMNG....DRPD...G
Solyc01g009090.2.1.sly.36140441                 593  ..........................GRL...................KDDRGNRKDRD..GRANGGERFDMSE...N
Thhalv10001919m.esa.20200705                    568  ..........................NRL...................RDDRGGRRERD..GRANGNDRVDRSE...D
AT2G27100.1.ath.19641299                        574  ..........................NRL...................RDDRGGRRERD..GRANGNDRNDRSE...D
Q9ZVD0                                          574  ..........................NRL...................RDDRGGRRERD..GRANGNDRNDRSE...D
Araha.4729s0005.1.p.aha.28848294                456  ..........................NRL...................RDDRGGHRERD..GRANGNDRIERSE...D
AL4G20790.t1.aly.35923270                       571  ..........................NRL...................RDDRGGRRERD..GRANGNDRNERSE...D
Bostr.29505s0043.1.p.adr.30675300               378  ..........................NRL...................RDDRGGRRERD..GRANGNDRNDRSE...D
Carubv10022733m.cru.20903468                    569  ..........................NRL...................RDDRGGRRERD..GRANGNDRNDRSD...D
AL4G20820.t1.aly.35924196                       569  ..........................NRL...................RDDRGGRRERD..GRANGNDRNDRSE...D
Carubv10022734m.cru.20901991                    570  ..........................NRL...................RDDRGGRRERD..GRANGDDRNDRSE...D
Bostr.28794s0002.1.p.adr.30657126               401  ..........................NRL...................RDDRGGRRERD..GRANGNDRNDRSE...D
Brara.D01611.1.p.bra.30620383                   548  ..........................SRL...................RDDRGGRRERD.........RIDRSE...D
Bol026461.bol.37343291                          547  ..........................SRL...................RDDRGGRRERD.........RIDRSE...D
Brara.G01299.1.p.bra.30635851                   487  ..........................NRL...................RDDRGGRRE.....RAYGNDRNDRSE...D
Bol027793.bol.37343994                          554  ..........................NRL...................RDDRGGRRE.....RAYGNDRNDRSE...D
mrna07673.1-v1.0-hybrid.fve.27266960            573  ..........................NRL...................KDERGNRRERE..NRANGGERYDRSE...N
Prupe.6G153800.1.p.ppe.32086540                 589  ..........................NRL...................KDERGNRRERE..NRVNGGERYDRSD...N
GSVIVT01037454001.vvi.17842075                  578  ..........................NRL...................KDN...RRERE..NRANGSERYDRSD...N
orange1.1g003865m.csi.18122801                  591  ..........................NRL...................KDERGNRRDRD..YRANGSDRYDRSD...N
Gorai.004G227500.1.gra.26773739                 572  ..........................NRL...................KDERGLRRERD..NRANGSDRFDRSE...N
Thecc1EG042011t1.tca.27426134                   571  ..........................NRL...................KDERGPRRERD..NRANGSDRYDRSE...N
evm.model.supercontig_103.67.cpa.16404954       382  ..........................NRL...................RDERG.RRERD..NRANGNDRYDRSD...N
Potri.004G196400.1.ptr.26990875                 591  ..........................NRL...................KDERGNRRERE..NRANGNERFDRSE...N
SapurV1A.0615s0230.1.p.spu.31434857             593  ..........................NRL...................KDERGNRRERE..NRVNGNERFDRSE...N
29942.m000751.rco.16814310                      593  ..........................NRL...................KDERGNRREHD..SRANGSERFDRSE...N
Manes.11G111400.1.p.mes.32355670                586  ..........................NRL...................KDERGGRRDRD..SRASGSERYDRSE...N
Manes.S021700.1.p.mes.32354619                  583  ..........................NRL...................KDERGGRRDRD..SRANGSERFDRSE...N
Eucgr.J00624.1.p.egr.32033287                   583  ..........................NRL...................RDDRGIRRERD..NRANGGERFDRSE...N
GSVIVT01027348001.vvi.17834761                  585  ..........................NRL...................KDDRGNRREHD.........RIERIN....
Kalax.0225s0069.1.p.kla.32599097                577  ..........................NRL...................KDDRGNRRGRD..SHI.GDDRFDRGE...N
Kaladp0081s0182.1.p.kfe.35761984                577  ..........................NRL...................KDDRGNRRGRD..SHI.GGDRFDRGE...N
Kalax.0216s0065.1.p.kla.32585583                574  ..........................NRL...................RDDRGSRRDRD..HHA.GSDRFVRGE...N
DCAR_002130.dca.36055141                        555  ..........................SYM...................KDERG.RRERD..NRTNGSERYDRGD...N
Aqcoe2G007600.1.p.aco.33056562                  552  ..........................NRL...................RDDRGNRREHERADRVDGDERYDRNE...N
Bradi3g03840.1.p.bdi.32813176                        ..............................................................................
Brast04G302900.1.p.bst.32866594                      ..............................................................................
Sevir.1G087000.1.p.svi.32667668                      ..............................................................................
Pavir.J22990.1.p.pvi.30308347                        ..............................................................................
Pahal.A00287.1.pha.32525941                          ..............................................................................
OAV97825.1                                      750  I...........................................SAGPSAPHGMMASMGH..................
XP_003293253.1                                       ..............................................................................
XP_005713144.1                                  559  T...........................................TINRNAAMGNGYAQT................MYN
XP_005702831.1                                       ..............................................................................
XP_009857252.1                                  748  ...........................................................................LQN
GES64941.1                                      696  ...........................................................................LSA
KEY79445.1                                      748  ...........................................................................LAN
XP_008869116.1                                  560  ..........................QRN...................RDSYGRGGGGGY..................
Q66I22                                               ..............................................................................
RXN03320.1                                           ..............................................................................
XP_015195966.1                                       ..............................................................................
B1H1X4                                               ..............................................................................
NP_001362205.1                                       ..............................................................................
Q99MR6                                               ..............................................................................
Q9BXP5                                               ..............................................................................
XP_005549330.1                                       ..............................................................................
H2ZUZ4                                               ..............................................................................
XP_001748332.1                                  708  AE..........TSLPPPALAPRGAAARF...................QEPMGGDSSTDDSNRPPPLPTSDRPGPLPA
XP_002286542.1                                       ..............................................................................
XP_649316.1                                          ..............................................................................
consensus>70                                         ...........................r......................d.g.........................
```

```
Pp3c25_7680V3.1.p.ppa.32980429                 589  PPPRDYP.LGGGGPPFE.GGPAQGG.PFEGGPMEP.PMFDHF....NGPPLRGLPGGLFVPDMPGPPQV..LMP.VP.
Pp3c16_11390V3.1.p.ppa.32986529                588  PPPRDYP.LGAGGPPFEGGGPAQGG.PFEGGPMEP.PMFDHF....NGPPVRGLPGGLFVPDMPGPPQV..LMP.VP.
Sphfalx0012s0220.1.p.sfa.32627721              651  PGPRDFPPLGGGIPPYEGGGQPQAG.HFDGDSMDA.PMFDRF....NGPPMHGLPGGLFVPDMPGPPQV..LMP.VP.
LOC_Os02g05610.1.osa.33135686                  578  .............................GPTPSE.LTPGAF....GGQGSFV........EM.PTPPV..LIP.VP.
LOC_Os08g40560.1.osa.33102829                  517  SPTHD......................GSSDNPDD..AYDSY....GDPVMH....GAFPPDI.PAPPV..LMP.VP.
Pavir.Fb01956.1.p.pvi.30278011                 508  SPPHD......................GSGENPDD.AIYDSF....GDPIMH....GAFPPDI.PAPPI..LMP.VP.
Pahal.F00690.1.p.pha.32488202                  511  SPPRD......................GSGENPED.AIYDSF....GDPIMH....GAFPPDI.PAPPI..LMP.VP.
Sevir.6G221600.1.p.svi.32643744                512  SPPHD......................GSGENPDD.AIYDSF....GDPIMH....GAFPPDI.PAPPI..LMP.VP.
Sobic.007G200600.1.p.sbi.37935124              511  SPTHD......................GSSENPDD.PIYDSF....GDPIMH....GAFPPDI.PAPPV..LMP.VP.
GRMZM2G132780_P02.zma.31013431                 510  SPKRD......................GSGENPDD.PIYDSF....GDPAMH....GAFPPDI.PAPPV..LMP.VP.
Zm00008a003300_P01.zma.37201202                510  SPTRD......................GSGENPDD.PIYDSF....GDPAMH....GAFPPDI.PAPPIS.LLP....
Bradi3g40630.1.p.bdi.32820467                  503  SPSQD......................GSGENPDD.PMYDSY....GNPTMH....GAFPPDV.PAPPM..LMP.VP.
Brast03G282200.1.p.bst.32857559                503  SPSQD......................GSGENPDD.PMYDSY....GNPTMH....GAFPPDV.PAPPM..LMP.VP.
Sobic.010G249100.1.p.sbi.37910925              589  SPSRD..........................GPDD.QMFDGF....RGRSSN....GPFVAEF.PPPPI..LMP.VP.
GRMZM5G817439_P01.zma.30992522                 588  SPSRD..........................GPDD.QMFDGF....RGRPSN....APFVAEF.PAPPI..LMP.VP.
Pavir.Da00165.1.p.pvi.30193584                 592  SPSRD..........................GPDD.QMLDAF....RGRGSN....APFVAEF.PAPPI..LMP.VP.
Sevir.4G256700.1.p.svi.32645485                593  SPSRD..........................GPDD.QMFDAF....RGRGSN....APFVAEF.PPPPI..LMP.VP.
LOC_Os06g48530.1.osa.33147743                  599  SPSLD..........................GADD.QMFDAF....RGRGPN....APFVPEL.PAPPI..LMP.IP.
Bradi1g33970.1.p.bdi.32801481                  577  SPNRD..........................GPDD.QMFDAF....RGRGSN....APFGSEF.PAPPI..LMP.VP.
Brast07G193800.1.p.bst.32871232                577  SPSHD..........................GPDD.QMFDAF....RGRGSN....APFGAEF.PAPPI..LMP.VP.
Migut.N02418.1.p.mgu.28925732                  619  SQSGDF..............ANQDG..ASGNNADD.PMYDSF....GGQGIPL....TYASDV..PPPV..LMP.VP.
Migut.D00714.1.p.mgu.28928758                  637  PQSDDFQ.............SGNDG.GAPGDNPDE.QMFDSF....AAQHMSL...GPFSGDI..PPPV..LMP.VP.
Medtr8g043980.1.mtr.31070730                   594  SPSRERQ...............SKA..LEMGNHDE.TMQDTF....AGPGV.....PPFSSDI..PPPV..LMP.VP.
Tp57577_TGAC_v2_mRNA25888.tpr.35955570         504  SPSRERQ...............SKP..LEMGNNDE.TMYDAY....AGPGV.....PPFSSDM.PPPXX..XXX.YL.
Medtr3g006760.1.mtr.31058945                   583  SPSHERQ......................LGNRDE.TMFDAY....GGPAV......SFNSDM.PPPPV..LMP.VP.
Glyma.08G227300.1.p.gma.30540922               595  SPSHEAM......................MGNRDE.TMYDSY....AGPGV.....PPFASDI.PPPPV..LMP.VP.
Phvul.011G187000.1.p.pvu.37156720              603  SPSNERQ..............S.....LEMGNRDE.TMYDSY....AGAGV.....PPFASDI.PPPPV..LMP.VP.
Phvul.005G010800.1.p.pvu.37152760              576  RDRGDRS.....................LSPSNEE.AMYEAY.......GV.....PPFTSDMPPPPPL..LMP.VP.
Glyma.06G314900.1.p.gma.30549408               591  SPSHERQ......................MGNHDE.AMYDAY....GGPGV.....PAFTSDM.PPPPV..LMP.VP.
Solyc01g009090.2.1.sly.36140441                621  PQSGDFQ.............SNNDG..ASGGNPDE.EMFDTF....GGQGIPV...ASFPSDM.APPPV..LMP.VP.
Thhalv10001919m.esa.20200705                   596  QQRGD.................GDG..PNGAN.QE.GGYEAF....GGQAGVH..VPPYSSDI.NPPPV..LMP.VP.
AT2G27100.1.ath.19641299                       602  QQRGD.................N.....DGGNPGE.VGYDAF....GGQGGVH..VPPFLSDI.NPPPM..LMP.VP.
Q9ZVD0                                         602  QQRGD.................N.....DGGNPGE.VGYDAF....GGQGGVH..VPPFLSDI.NPPPM..LMP.VP.
Araha.4729s0005.1.p.aha.28848294               484  QQRGD.................N.....DGGNPGE.LGYDAF....GGQGGVH..VPPFLSDI.NPPPM..LMP.VP.
AL4G20790.t1.aly.35923270                      599  QQRGD.................N.....DGGNPGE.LGYDAF....GGQGGVH..VPPFLSDI.NPPPM..LMP.VP.
Bostr.29505s0043.1.p.adr.30675300              406  QQRGD.................GGG..LDGANPGE.VGYDAF....GGQGGVH..VPPFLSDI.NPPPM..LMP.VP.
Carubv10022733m.cru.20903468                   597  RQRGE.................GDG..PDGGNPGE.VGYDAF....GGQGGVH..VPPFLSDI.NPPPM..LMP.VP.
AL4G20820.t1.aly.35924196                      597  QQRGD.................GDG..PDGGNPRE.VGYDAF....GGQGGVH..VPPFSSDI.NPPPM..LMP.VP.
Carubv10022734m.cru.20901991                   598  QVRGE.................GDG..PDGGNPGD.VGYDAF....GGQGGVH..VPPFSSDI.NPPPM..LMP.VP.
Bostr.28794s0002.1.p.adr.30657126              429  QLRGD.................GDG..PDGGNPGE.FGYDAF....GGQGGVH..VPPFSSDI.NPPPM..LMP.VP.
Brara.D01611.1.p.bra.30620383                  569  QQRGD.................GNG..PNGPNPEE.GGYDAF....GGQGGVH..VPSYSADM.NAPPV..LMP.VP.
Bol026461.bol.37343291                         568  QQRGD.................G.....NGPNPEE.GGYDAF....GGQGGVH..VPSYSADM.NAPPV..LMP.VP.
Brara.G01299.1.p.bra.30635851                  512  QRRGD.................GNG..PVDPNPEEGGGFEAF....GGQGGFQ..VPPYSADM.NAPPM..LMP.VP.
Bol027793.bol.37343994                         579  QRRGD.................GNG..PVDPNPEEGGGFDAFG...GGQGGFQ..VPSYSADI.NAPPM..LMP.VP.
mrna07673.1-v1.0-hybrid.fve.27266960           601  MQQGEFP.............SNNDG..QDGGNHDE.PMYDNF....GGQGMHG..GPPFPSDI.TPPPV..LMP.VP.
Prupe.6G153800.1.p.ppe.32086540                617  MQSSEFQ.............SNNDG..PEGGNLDE.PMFDNF....GGQGVRV..GPPFPSDI.PPPPV..LMP.VP.
GSVIVT01037454001.vvi.17842075                 603  HQSNDFQ.............P.NDG..PDGGNPGE.GMFDTF....GGQGMHV..APPFPSDM.PPPPV..LMP.VP.
orange1.1g003865m.csi.18122801                 619  AQASDFP.............SNDDV..PEGANRDE.SMFDTF....GGQGIRV..APPFPSDI.PPPPV..LMP.VP.
Gorai.004G227500.1.gra.26773739                600  PQSSDFP.............SNNDG..PDGGNHDD.PMFDAF....GGQGMHV..AAPFSSDI.APPPV..LMP.VP.
Thecc1EG042011t1.tca.27426134                  599  PQSSDFT.............SNNDG..PDGGNRDD.TMFDAF....GGQGMRV..AAPFSSDI.APPPV..LMP.VP.
evm.model.supercontig_103.67.cpa.16404954      409  QQSGEFP.............SNNEV..SDGGNHDE.AMYDAF....GGQGMRV...APFSSDI.APPPV..LMP.VP.
Potri.004G196400.1.ptr.26990875                619  PQSGDFQ.............S.NDG..PDGGNCDE.TMFDTF....GGQGIRV..PPPFPSDI.APPPV..LMP.VP.
SapurV1A.0615s0230.1.p.spu.31434857            621  SQSGDFQ.............S.SDG..PDGGNCDE.TMFDAF....GGQGIHV..PPPFPSDI.APPPV..LMP.VP.
29942.m000751.rco.16814310                     621  PQTGDFQ.............SNNDG..PDGGNHDD.GMYDNF....GGQGIHV...PSFPSDI.PPPPV..LMP.VP.
Manes.11G111400.1.p.mes.32355670               614  PPSGDVQ.............TNNGG..PDGGNHDD.PMYDNF....GGQAMHV...PPFPSDI..PPPV..LMP.VP.
Manes.S021700.1.p.mes.32354619                 611  QQSGDFQ.............SNNDV..PEGGNHDD.PMYDNF....GAQGMRV...PPFPSDI.PPPPV..LMP.VP.
Eucgr.J00624.1.p.egr.32033287                  611  PQSAD..................KDG..ADGGDGDD.PMYDSF....SGQGLHV...APFASDI.PPPPV..LMP.VP.
GSVIVT01027348001.vvi.17834761                 605  ............................DGGNLDE.SMYDTY....GGKDLGV..IPPFPSDM.GPPPV..LMP.VP.
Kalax.0225s0069.1.p.kla.32599097               604  MHSND..................DG..PDG.IHDE.PIFD......DGQGMHG..GPPFSSEI.APPSV..LMP.VP.
Kaladp0081s0182.1.p.kfe.35761984               604  MHSND..................DG..PDG.IHDE.PIFD......DGQGMHG..GPPFSSEI.APPSV..LMP.VP.
Kalax.0216s0065.1.p.kla.32585583               601  IHLND..................DG..PDG.NHDE.QMFD.......GQGMHG..GPPFSSEM.APPSV..LMP.VP.
DCAR_002130.dca.36055141                       582  PHSVDFQ.............ANNDA..QAGSNLDE.SMFDGF....NGQGMPV...SSFPSDM.APPPV..LMP.VP.
Aqcoe2G007600.1.p.aco.33056562                 582  SPSRDFP.............TKKDD..PEGRKPDD.PMYDNF....AGQGMRA..APPFPSDIPPPPPV..LMP.VP.
Bradi3g03840.1.p.bdi.32813176                  582  .................................DE.QISGAF....DLHGLN....APFAPES.LPPPL..LIP.VP.
Brast04G302900.1.p.bst.32866594                655  .................................DE.QISGAF....DLHDLN....GPFAPEF.PPPPL..LIP.VP.
Sevir.1G087000.1.p.svi.32667668                596  .................................DE.QMFGAS....GGRGSDA.......PLLPDPSPV..LIP.VP.
Pavir.J22990.1.p.pvi.30308347                  587  .................................DE.QKYGAS....GGRGSDT.......LLLPKPSLV..LIP.VP.
Pahal.A00287.1.pha.32525941                    579  .................................DE.QMYGAS....GGRGSDT.......LLLSKPSLV..LIP.VP.
OAV97825.1                                     767  .............HPGDF.........HNGGYHA..PMMNNSM...GGPGGMM.MGGPYGSYM.PPPPM..AGP.SPM
XP_003293253.1                                 687  .............................GG.............AGRGLPPGIAGQPRARF.NPPP..GMLPNQPM
XP_005713144.1                                 578  PYMVGMQ......FPLMM..PTAAG..FTGGYAGT.PGYANVM.AFAGRGMQT..ASMGRPGM...PP.GGIMP..P.
XP_005702831.1                                 611  ..............................................MGRNMSS.............MPV..LLP.SG.
XP_009857252.1                                 751  ....................................YAMNSML...NFPGFPI.............PS...MFPNMMN
GES64941.1                                     699  .............MPA....................AYLGGGNLPAGFQSVPG..............A...GVPGMMG
KEY79445.1                                     751  .............LPP....................YLANNVNIPGGTHGLPG.............AA...GIPGFMN
XP_008869116.1                                 575  .............HPY...........YDGG.....PRGGGYR...GGRGGGY.NTG.........PP.......TPN
Q66I22                                         803  .............................G................PGQGV...........L...SP..GGLP.FP.
RXN03320.1                                     739  .............................G................PGQGV...........L...SP..GGLP.FP.
XP_015195966.1                                 676  .............................G................PGQGV...........L...SP...GLP.YP.
B1H1X4                                         757  ............................VGA...............AGQ............AL...AA...GLL.YP.
NP_001362205.1                                 756  ............................VGT...............AGQ............AL...AA...GLL.YP.
Q99MR6                                         771  .............................G................PAQIL........PPGL...TP...GLP.YP.
Q9BXP5                                         772  .............................G................PAQIL........PPGL...TP...GLP.YP.
XP_005549330.1                                 741  .............................G................PAQIL........PPGL...TP...GLP.YP.
H2ZUZ4                                         721  .............................G................CETRSLK.IVMMSFSGM...SP...ALP.YP.
XP_001748332.1                                 757  PPSPAAAP....ATP.....PPREGRPPAGPPPV..........AQRGPALPP...SRPQAQTPPPPPM..VPP.VG.
XP_002286542.1                                 677  ..................................................................PPIQGLVP.SAV
XP_649316.1                                    509  ..............PL..........PSTGPLSSY.QMINNQQPPLSGIDQTI...........PPPP...QIP.YQV
consensus>70                                        ..................................e...yd.f.....g..........f..d....pp...lmp.vp.
```

```
Pp3c25_7680V3.1.p.ppa.32980429            655  .......................GAG.P.................LG..PF.VPAPP.....................
Pp3c16_11390V3.1.p.ppa.32986529           655  .......................GAG.P.................LG..PF.VPAPP.....................
Sphfalx0012s0220.1.p.sfa.32627721         719  .......................GAG.P.................LG..PF.VPAPP.....................
LOC_Os02g05610.1.osa.33135686             609  .......................GAG.P.................LG..PF.VPAPP.....................
LOC_Os08g40560.1.osa.33102829             558  .......................GAG.P.................LG..PF.IPAPP.....................
Pavir.Fb01956.1.p.pvi.30278011            550  .......................GAG.P.................LG..PF.IPAPP.....................
Pahal.F00690.1.pha.32488202               553  .......................GAG.P.................LG..PF.IPAPP.....................
Sevir.6G221600.1.p.svi.32643744           554  .......................GAG.P.................LG..PF.IPAPP.....................
Sobic.007G200600.1.p.sbi.37935124         553  .......................GAG.P.................LG..PF.IPAPP.....................
GRMZM2G132780_P02.zma.31013431            552  .......................GAG.P.................LG..PF.IPAPP.....................
Zm00008a003300_P01.zma.37201202           551  .........................R.P.................LG..PF.IPAPP.....................
Bradi3g40630.1.p.bdi.32820467             545  .......................GAG.P.................LG..PF.IPAPP.....................
Brast03G282200.1.p.bst.32857559           545  .......................GAG.P.................LG..PF.IPAPP.....................
Sobic.010G249100.1.p.sbi.37910925         627  .......................GAG.P.................LG..PF.VPAPP.....................
GRMZM5G817439_P01.zma.30992522            626  .......................GAG.P.................LG..PF.VPAPP.....................
Pavir.Da00165.1.p.pvi.30193584            630  .......................GAG.P.................LG..PF.VPAPP.....................
Sevir.4G256700.1.p.svi.32645485           631  .......................GAG.P.................LG..PF.VPAPP.....................
LOC_Os06g48530.1.osa.33147743             637  .......................GAG.P.................LG..PF.VPAPP.....................
Bradi1g33970.1.p.bdi.32801481             615  .......................GAG.P.................LG..PF.VPAPP.....................
Brast07G193800.1.p.bst.32871232           615  .......................GAG.P.................LG..PF.VPAPP.....................
Migut.N02418.1.p.mgu.28925732             666  .......................GAG.P.................MG..PF.VPAPP.....................
Migut.D00714.1.p.mgu.28928758             687  .......................GAG.P.................LG..PF.VPAPP.....................
Medtr8g043980.1.mtr.31070730              639  .......................GAG.P.................LG..PF.VPAPP.....................
Tp57577_TGAC_v2_mRNA25888.tpr.35955570    550  .......................VLGQP.................LG..PF.VPAPP.....................
Medtr3g006760.1.mtr.31058945              623  .......................GAG.P.................LG..PF.VPAPP.....................
Glyma.08G227300.1.p.gma.30540922          636  .......................GAG.P.................LG..PF.VPAPP.....................
Phvul.011G187000.1.p.pvu.37156720         647  .......................GAG.P.................LG..PF.VPAPP.....................
Phvul.005G010800.1.p.pvu.37152760         616  .......................GAG.P.................LG..PF.VPAPP.....................
Glyma.06G314900.1.p.gma.30549408          632  .......................GAG.P.................LG..PF.VPAPP.....................
Solyc01g009090.2.1.sly.36140441           671  .......................GAG.P.................LG..PF.VPAPP.....................
Thhalv10001919m.esa.20200705              642  .......................GAG.P.................LG..PF.VPAPP.....................
AT2G27100.1.ath.19641299                  646  .......................GAG.P.................LG..PF.VPAPP.....................
Q9ZVD0                                    646  .......................GAG.P.................LG..PF.VPAPP.....................
Araha.4729s0005.1.p.aha.28848294          528  .......................GAG.P.................LG..PF.VPAPP.....................
AL4G20790.t1.aly.35923270                 643  .......................GAG.P.................LG..PF.VPAPP.....................
Bostr.29505s0043.1.p.adr.30675300         453  .......................GAG.P.................LG..PF.VPAPP.....................
Carubv10022733m.cru.20903468              644  .......................GAG.P.................LG..PF.VPAPP.....................
AL4G20820.t1.aly.35924196                 644  .......................GAG.P.................LG..PF.VPAPP.....................
Carubv10022734m.cru.20901991              645  .......................GAG.P.................LG..PF.VPAPP.....................
Bostr.28794s0002.1.p.adr.30657126         476  .......................GAG.P.................LG..PF.VPAPP.....................
Brara.D01611.1.p.bra.30620383             616  .......................GAG.P.................LG..PF.VPAPP.....................
Bol026461.bol.37343291                    612  .......................GAG.P.................LG..PF.VPAPP.....................
Brara.G01299.1.p.bra.30635851             560  .......................GAG.P.................LG..PF.VPAPP.....................
Bol027793.bol.37343994                    628  .......................GAG.P.................LG..PF.VPAPP.....................
mrna07673.1-v1.0-hybrid.fve.27266960      652  .......................GAG.P.................LG..PF.VPAPP.....................
Prupe.6G153800.1.p.ppe.32086540           668  .......................GAG.P.................LG..PF.VPAPP.....................
GSVIVT01037454001.vvi.17842075            653  .......................GAG.P.................LG..PF.VPAPP.....................
orange1.1g003865m.csi.18122801            670  .......................GAG.P.................LG..PF.VPAPP.....................
Gorai.004G227500.1.gra.26773739           651  .......................GAG.P.................LG..PF.VPAPP.....................
Thecc1EG042011t1.tca.27426134             650  .......................GAG.P.................LG..PF.VPAPP.....................
evm.model.supercontig_103.67.cpa.16404954 459  .......................GAG.P.................LG..PF.VPAPP.....................
Potri.004G196400.1.ptr.26990875           669  .......................GAG.P.................LG..PF.VPAPP.....................
SapurV1A.0615s0230.1.p.spu.31434857       671  .......................GAG.P.................LG..PF.VPAPP.....................
29942.m000751.rco.16814310                671  .......................GAG.P.................LG..PF.VPAPP.....................
Manes.11G111400.1.p.mes.32355670          663  .......................GAG.P.................LG..PF.VPAPP.....................
Manes.S021700.1.p.mes.32354619            661  .......................GAG.P.................LG..PF.VPAPP.....................
Eucgr.J00624.1.p.egr.32033287             657  .......................GAG.P.................LG..PF.VPAPP.....................
GSVIVT01027348001.vvi.17834761            643  .......................GAG.P.................LG..PF.VPAPP.....................
Kalax.0225s0069.1.p.kla.32599097          647  .......................GAG.P.................LG..PF.VPAPP.....................
Kaladp0081s0182.1.p.kfe.35761984          647  .......................GAG.P.................LG..PF.VPAPP.....................
Kalax.0216s0065.1.p.kla.32585583          643  .......................GAG.P.................LG..PF.VPAPP.....................
DCAR_002130.dca.36055141                  632  .......................GAG.P.................LG..PF.VPAPP.....................
Aqcoe2G007600.1.p.aco.33056562            634  .......................GAG.P.................LG..PF.VPAPP.....................
Bradi3g03840.1.p.bdi.32813176             613  .......................GAG.P.................LG..PF.VPAPP.....................
Brast04G302900.1.p.bst.32866594           686  .......................GAG.P.................LG..PF.VPAPP.....................
Sevir.1G087000.1.p.svi.32667668           625  .......................GAG.P.................YG..PF.VPAPP.....................
Pavir.J22990.1.p.pvi.30308347             616  .......................GAG.P.................YG..PF.VPAPS.....................
Pahal.A00287.1.pha.32525941               608  .......................GAG.P.................YG..PF.VPAPP.....................
OAV97825.1                                813  FP........NGSMMGG.HHGS.GGG.PGYGA.......PYYMGNMG..PG.APYPSQVYSSLPAIPPPAHPIRAPNG
XP_003293253.1                            718  VQLMSPVF.A..SPNGI.....RGRG.P.................NG.......VPP.....................
XP_005713144.1                            635  .......................GRS.P.............HDSNMGV....HHRPP...........PRR.......
XP_005702831.1                            626  .......................MVA.P....................MQF.VPGSF...............VR....
XP_009857252.1                            774  AGAMAGA.ASNWHAAGG.DDRTGAGG.PIRRGGHMGGAGGRFQSRTG..PY.DRRPN.....................
GES64941.1                                727  MNAPAGSWGGNGFVPGD.SSGLHQPG.PMRRGG........RHNRSG..PY.DRRGN.............RPVRGM.N
KEY79445.1                                780  VNNQA..WNANGLVTGEHAANLHHPG.VIRRGG......GRYNNRSG..PY.DRRSN.............RPARGMSS
XP_008869116.1                            604  Y...........................P...........RRYDS........NPRPP.....................
Q66I22                                    818  .......................PQG.P.................QGLMGFGQPRPP...............LMGYG.
RXN03320.1                                754  .......................PQG.P.................QGLMGFGQPRPP...............VMGYG.
XP_015195966.1                            690  .......................HQS.P.................QGLMGYGQPRPP...............VLGYGG
B1H1X4                                    772  .......................HQG.P.................PALLPYAQPRPP...............VLGYG.
NP_001362205.1                            771  .......................HQG.P.................QALLPYAQPRPP...............VLGYA.
Q99MR6                                    788  .......................HQT.P.................QGLMPYGQPRPP...............ILGYGA
Q9BXP5                                    789  .......................HQT.P.................QGLMPYGQPRPP...............ILGYGA
XP_005549330.1                            758  .......................HQT.P.................QGLMPYGQPRPP...............ILGYGA
H2ZUZ4                                    745  .......................HQS.P.................QGLMGYGQPRPP...............VLGYGV
XP_001748332.1                            809  .......................GRG.PL..............PEVGGAPV.GPPPDHLFQFNTNLPPPDRPVVDVKS
XP_002286542.1                            688  I......................GSAEP......TAH...............DPEPE.LWK.................
XP_649316.1                               547  NQQ...............TNETYSQP.P...................QLPV.RNQPP.....................
consensus>70                                   .......................gag.p.................lg..pf.vpapp.....................
```

```
Pp3c25_7680V3.1.p.ppa.32980429                668  ..........EVAMRFLRE...GPGP..........FRPGGFDSGYQEE...GPRGG..KRGAP........GP.MM
Pp3c16_11390V3.1.p.ppa.32986529               668  ..........EIAMRFLRE...GPGP..........FRMGGFDAGYQEEGNGGPRGG..KRGAP........GP.MM
Sphfalx0012s0220.1.p.sfa.32627721             732  ..........EIAMRFLRE...GPTP..........FHPGAFVGGFDGDGNGMPRG.RKRLPVG........GP.MM
LOC_Os02g05610.1.osa.33135686                 622  ..........EVVMQMMR..............................................................
LOC_Os08g40560.1.osa.33102829                 571  ..........EVAMRMMRDQ.GGPPP..........FEPTGG............PRP.RRPGRG........GL.PM
Pavir.Fb01956.1.p.pvi.30278011                563  ..........EVAMHMFRDQ.GGPPP..........FEPAGG............PHP.RKAGRG.......GGP.PM
Pahal.F00690.1.pha.32488202                   566  ..........EVAMHMLRDQ.GGPPP..........FEPAGG............PHP.RKAGRG.......GGP.PM
Sevir.6G221600.1.p.svi.32643744               567  ..........EVAMHMMRDQ.GGPPH..........FEPAGG............PHP.RKAGRG.......GGP.PM
Sobic.007G200600.1.p.sbi.37935124             566  ..........EVAMHMLRDQ.GAPPP..........FEPTGG............PHP.RKAGRG.......VGP.QI
GRMZM2G132780_P02.zma.31013431                565  ..........EVAMHMLRDQ.GAPPP..........IEPTGG............PHP.RKAGTG.......GGP.SM
Zm00008a003300_P01.zma.37201202               562  ..........EVAMHMLRDQ.GAPPP..........IEPTGG............PHP.RKAGKG.......GGP.SM
Bradi3g40630.1.p.bdi.32820467                 558  ..........EVAMRMLREQ.GGPTP..........FEPAAG............PRP.RKAGKG.......GGP.PM
Brast03G282200.1.p.bst.32857559               558  ..........EVAMRMLREQ.GGPPP..........FEPAAG............PRP.RKAGRG.......GGP.PM
Sobic.010G249100.1.p.sbi.37910925             640  ..........EIAMHMLREQ.GPPPP..........FEPNGP............PHG..NTGML........GP.MM
GRMZM5G817439_P01.zma.30992522                639  ..........EIAMHMLREQ.GPPPP..........FEPNGA............PHG..NAGML........GP.MM
Pavir.Da00165.1.p.pvi.30193584                643  ..........EIAMHMLREQ.GPPPP..........FEPNGA............PHG..NTGML........GP.MM
Sevir.4G256700.1.p.svi.32645485               644  ..........EIAMHMMREQ.G.PPP..........FEPNGA............PHG..NTGML........GP.MM
LOC_Os06g48530.1.osa.33147743                 650  ..........EIAMHMLREQ.GPPPP..........FEPNGP............PHA..NPGVL........GP.MM
Bradi1g33970.1.p.bdi.32801481                 628  ..........EIAMHMLREQ.G.PPP..........FEPNGA............PHG..NPQVL........GQ.MM
Brast07G193800.1.p.bst.32871232               628  ..........EIAMHMLREQ.G.PPP..........FEPNGA............PHG..NSAVL........GQ.MM
Migut.N02418.1.p.mgu.28925732                 679  ..........EVAMQMMRDRGGGPSP..........FEG.G..................R.................
Migut.D00714.1.p.mgu.28928758                 700  ..........EVAMQMMRDQ.GGPSP..........FDG.G..................RNGRS........GP.QL
Medtr8g043980.1.mtr.31070730                  652  ..........EVAMQMFRDQ.GGPSS..........FDGSG..................INMLS........GPHHM
Tp57577_TGAC_v2_mRNA25888.tpr.35955570        564  ..........EVAMQMLREQ.GGPSS..........YDASG..................RNMRS........GP.HI
Medtr3g006760.1.mtr.31058945                  636  ..........EVAMQMFRDQ..GPSS..........YDPSG..................MTMRS........GP.HI
Glyma.08G227300.1.p.gma.30540922              649  ..........EVAMQMLRDQ.GG.SS..........YDASG..................RKMHS........GP.PM
Phvul.011G187000.1.p.pvu.37156720             660  ..........EVAMQMLRDQ.GGP.S..........YDASG..................RKMRG........GP.HM
Phvul.005G010800.1.p.pvu.37152760             629  ..........EVAMQMLREQGGGGAS..........YDPSG..................RKMRS........GP.HM
Glyma.06G314900.1.p.gma.30549408              645  ..........EVAMQMFREQ.GGPTS..........FDASG..................RKMRS........GP.HM
Solyc01g009090.2.1.sly.36140441               684  ..........EVAMRMMREQ.GGPTP..........FEG.G..................RNGRS........GP...
Thhalv10001919m.esa.20200705                  655  ..........EFAMQMFRDP.SGPPS..........FEGSG..................R.................
AT2G27100.1.ath.19641299                      659  ..........EVAMQMFRDP.SGPPP..........FEGSG..................R.................
Q9ZVD0                                        659  ..........EVAMQMFRDP.SGPPP..........FEGSG..................R.................
Araha.4729s0005.1.p.aha.28848294              541  ..........EVAMQMFRDP.SGPPP..........FEGSG..................R.................
AL4G20790.t1.aly.35923270                     656  ..........EVAMQMFRDP.SGPPP..........FEGSG..................R.................
Bostr.29505s0043.1.p.adr.30675300             466  ..........EVAMQMFRDP.SGPPP..........FEGNG..................R.................
Carubv10022733m.cru.20903468                  657  ..........EVAMQMFRDP.SGPPP..........FEGSG..................R.................
AL4G20820.t1.aly.35924196                     657  ..........EFAMQMF..P.SGPPP..........FEGNG..................R.................
Carubv10022734m.cru.20901991                  658  ..........EFAMQMF..P.SGPPT..........FEGSG..................R.................
Bostr.28794s0002.1.p.adr.30657126             489  ..........EFAMQMF..P.SGPPP..........FEGSG..................R.................
Brara.D01611.1.p.bra.30620383                 629  ..........EFAMQMFRDP.SGPPP..........LEGGG..................R.................
Bol026461.bol.37343291                        625  ..........EFAMQMFRDP.SGPPP..........LEGGG..................R.................
Brara.G01299.1.p.bra.30635851                 573  ..........EVAMQMFRDP.SGPPS..........FEGGG..................R.................
Bol027793.bol.37343994                        641  ..........EVAMQMFRDP.SGPPS..........FEGGG..................R.................
mrna07673.1-v1.0-hybrid.fve.27266960          665  ..........EVAMQMLRDQ.GGPPP..........FEGSG..................RNMWP........GP.QM
Prupe.6G153800.1.p.ppe.32086540               681  ..........EVAMQMLREQ.GGPPP..........FEGSG..................RNVWS........GP.QM
GSVIVT01037454001.vvi.17842075                666  ..........EVAMRMLREQ.GGPSP..........FEGGG..................RNGRS........GP.QL
orange1.1g003865m.csi.18122801                683  ..........EVAMQMIRDQ.GG.PP..........FEGGG..................RHGRP........GP.QI
Gorai.004G227500.1.gra.26773739               664  ..........ELAMQVFRER.GG.PP..........FEGNS..................RSGRP........GP.NL
Thecc1EG042011t1.tca.27426134                 663  ..........ELAMQVFRER.GGPPP..........FEGNS..................RGGRP........GP.NL
evm.model.supercontig_103.67.cpa.16404954     472  ..........EVAMRMFREQ.GG.PS..........FEGGG..................RNGRP........GP.QL
Potri.004G196400.1.ptr.26990875               682  ..........EVAMRMFRDQ.GGPPP..........FESGG..................RNARP........GP.QL
SapurV1A.0615s0230.1.p.spu.31434857           684  ..........EVAMRMFRDQ.GGPPP..........FESGG..................RKARP........GP.QL
29942.m000751.rco.16814310                    684  ..........EVAMRMFREQ.GGPPP..........FESGG..................RNGRP........GP.QL
Manes.11G111400.1.p.mes.32355670              676  ..........EVAMRMFREQ.GGPPP..........FEGGG..................RNGRP........GP.QL
Manes.S021700.1.p.mes.32354619                674  ..........EVAMRMFREQ.GGPPP..........FEGGG..................RSGRP........GP.QL
Eucgr.J00624.1.p.egr.32033287                 670  ..........EVAMQMFREQ.GAPPP..........FEGAG..................RNGRP........GS.QL
GSVIVT01027348001.vvi.17834761                656  ..........EVAMRMLREQ.GGSSS..........YEG.G..................RQMRT........GP.HI
Kalax.0225s0069.1.p.kla.32599097              660  ..........DIAMRLLREQ.GGPPP..........FEGGG..................RNGRP........GP.QG
Kaladp0081s0182.1.p.kfe.35761984              660  ..........DVAMRLLREQ.GGPPP..........FEGGG..................RNGRP........GP.QG
Kalax.0216s0065.1.p.kla.32585583              656  ..........DVAMRLLRGQ.GGPPP..........FEGGG..................RNGLP........GS.QG
DCAR_002130.dca.36055141                      645  ..........EIAMQMMREQ.GGPSP..........FEGGG..................RNGRP........GS.HL
Aqcoe2G007600.1.p.aco.33056562                647  ..........EVAMRMLREQ.GGPSP..........FEGNG..................RKGRN........GP.QV
Bradi3g03840.1.p.bdi.32813176                 626  ..........EMAIQMMEEQ.RPPRP.............NGA.................QHGKP.........P.LM
Brast04G302900.1.p.bst.32866594               699  ..........EMAIQMMEEQ.RPPRP.............NGA.................QHGKP.........P.LL
Sevir.1G087000.1.p.svi.32667668               638  ..........EMAMQMMQKGHSGP...............DAA.................RHGKP...........SV
Pavir.J22990.1.p.pvi.30308347                 629  ..........EMAMQMTQKGHSGP...............DAA.................RQWKP...........SV
Pahal.A00287.1.pha.32525941                   621  ..........EMAMQVIQKGFPGP...............DAA.................RHWKP...........SV
OAV97825.1                                    870  NNR....RLGERISRHFADEIGLPPPHYHD.............................HKRSRRDEF.....RPP...
XP_003293253.1                                742  ..........HGIVPMRRSY.GGS...........TVSGAGG...............NPRGGYS.......DQP.SS
XP_005713144.1                                654  ..........EGNRGGLRGRRGGP............RRGVGD...............YYRGGHA.............
XP_005702831.1                                640  .....................GSH...........LYRG..................MKRRGRRRVGGLRN......
XP_009857252.1                                825  .......................PPGGM..DGGMAGRGRGVGGP.....................TN....RW
GES64941.1                                    778  MY........GSGGRLPSTA.GTPPPGH..PAAAA..F.GAGGF......................AD....AM
KEY79445.1                                    833  MY........GAGARMGASG.AVPPPGH..PAAAA..LMGAGPF......................PD....AM
XP_008869116.1                                616  ............................................................................QS
Q66I22                                        839  .....................GGPPPN........QY.GGG..................RGNYDNFRG.........
RXN03320.1                                    775  .....................GGPPPN........QYGGGG..................RGNYDNFRG.........
XP_015195966.1                                712  .....................GGPPPN........QY.GGG..................RGNYDSFRG.........
B1H1X4                                        793  .....................GAPPPN........PY.GAG..................RGNYDAFRG.........
NP_001362205.1                                792  .....................GAPPPT........PY.GAG..................RGNYDAFRG.........
Q99MR6                                        810  GAVRPAVPT............GGPPHA........PY.GAG..................RGNYDAFRG.........
Q9BXP5                                        811  GAVRPAVPT............GGPPHA........PY.GAG..................RGNYDAFRG.........
XP_005549330.1                                780  GAVRPAVPT............GGPPHA........PY.GAG..................RGNYDAFRG.........
H2ZUZ4                                        767  .FVLFFLLL............GAPPPN........PY.G.G..................RGNYDSFRG.........
XP_001748332.1                                848  TQAGWYYRGPGIDQRIEEVR.GAPPPNMLEATGSGFRLQGTGENLQF.....HPTFNEEREAKSKMAGATNSRP.QA
XP_002286542.1                                705  ..........EEEARMMEEE.......................................RRRQERQE...........AA
XP_649316.1                                   568  ..........QLSPRSQQNS.QAPPPRGQQNTQVPPQLPPRGQQVP........PQL.PPRSQQQ.....PNINP.QM
consensus>70                                       ..........e.am.m.re...gp............f...g..................r.................
```

```
Pp3c25_7680V3.1.p.ppa.32980429                 708  .GGGILDIPPMMMP..SHGMRRDPRGVRSYHDLDAPEDEVSVIDYRSL
Pp3c16_11390V3.1.p.ppa.32986529                711  .EGGLLDIPPNMMP..PHGMRRDPRGVRSYHDLDAPEDEVSVIDYRSL
Sphfalx0012s0220.1.p.sfa.32627721              776  .GGGLLDVPPMMMP.PPHAMRHDPRSVRSYHDLDAPEDEVTVIDYRSL
LOC_Os02g05610.1.osa.33135686                  630  ........PVMPMY...PPRPPNPRRLRSYKDLDAPDDEVTLVDYRSL
LOC_Os08g40560.1.osa.33102829                  605  .GGP...SPILAAPL.PPPHMHDPRKIRSYQDLDAPEDEVTVMDYRSL
Pavir.Fb01956.1.p.pvi.30278011                 598  .RGP...SPMFSAP..PPPHMHDPRRIRSYQDLDAPEDEVTVMDYRSL
Pahal.F00690.1.pha.32488202                    601  .HGP...SPIFSAP..PPPHMHDPRRIRSYQDLDAPEDEVTVMDYRSL
Sevir.6G221600.1.p.svi.32643744                602  .RGP...SPIFSAP..PPTHMHDPRRIRSYQDLDAPEDEVTVMDYRSL
Sobic.007G200600.1.p.sbi.37935124              601  .RGP...SPIFTAP..PPHQMHDPRRIRSYQDLDAPEDEVTVMDYRSL
GRMZM2G132780_P02.zma.31013431                 600  .RGP...SPIFGAP..SPHHMHDPRRIRSYQDLDAPEDEVTVMDYRSL
Zm00008a003300_P01.zma.37201202                597  .RGP...SPIFGAP..SPHHMHDPRRIRSYQDLDAPEDEVTVMDYRSL
Bradi3g40630.1.p.bdi.32820467                  593  .GGP...SPMLNAPLMHDPRMHDPRKIRSYQDLDAPGDEVTVMDYRSL
Brast03G282200.1.p.bst.32857559                593  .GGP...SPMLNAPLMHDPRMHDPRKIRSYQDLDAPGDEVTVMDYRSL
Sobic.010G249100.1.p.sbi.37910925              673  .GGP...APIIAMP...PNFRHDPRRLRSYNDLDAPDEEVTVLDYRSL
GRMZM5G817439_P01.zma.30992522                 672  .GGP...APIIAMP...PNFRHDPRRLRSYNDLDAPDEEVTVLDYRSL
Pavir.Da00165.1.p.pvi.30193584                 676  .GGP...APIITMP...PNFRHDPRRLRSYNDLDAPDEEVTVLDYRSL
Sevir.4G256700.1.p.svi.32645485                676  .GGP...APIITMP...PNFRHDPRRLRSYNDLDAPDEEVTVLDYRSL
LOC_Os06g48530.1.osa.33147743                  683  .GGP...APIITMP...PSFRQDPRRLRSYNDLDAPDEEVTVLDYRSL
Bradi1g33970.1.p.bdi.32801481                  660  GGGP...APIIAMP...PSFHHDPRRLRSYNDLDAPDEEVTVLDYRSL
Brast07G193800.1.p.bst.32871232                660  GGGP...APIIAMP...PSFHHDPRRLRSYNDLDAPDEEVTVLDYRSL
Migut.N02418.1.p.mgu.28925732                  700  .NGP...SPIIALP...PRHRQDPRSLRSYNDLDAPDDEVTVIDYRSL
Migut.D00714.1.p.mgu.28928758                  728  .GGP...APIIALP...SNFRQDPRRLRSYNDLDAPDDMRTLLSFTSH
Medtr8g043980.1.mtr.31070730                   682  .GGP...APIITLP...PGFRPDPRKMRSYQDLDAPDEEVTVIDYRSL
Tp57577_TGAC_v2_mRNA25888.tpr.35955570         593  .GGP...APIIAVP...PAFRPDPRKMRSYQDLDAPDDEVTVIDYRSL
Medtr3g006760.1.mtr.31058945                   664  .GGQ...ASMIAVP...PTFRPDPRRMRSYQDLDAPEEEVTVIDYRSL
Glyma.08G227300.1.p.gma.30540922               677  .GGG...SSIIAVP...PTFRPDPRRMRSYQDLDAPEDEVTVIDYRSL
Phvul.011G187000.1.p.pvu.37156720              688  .SGA...APIIAVP...PNFRPDPRRMRSYQDLDAPEDEVTVIDYRSL
Phvul.005G010800.1.p.pvu.37152760              659  GGGP...GPIIAVP...PSFRPDPRQMRSYEDLDAPEDEVTVIDYRSL
Glyma.06G314900.1.p.gma.30549408               674  .GGP...APIIAVP...PSFRPDPRRMRSYEDLDAPEDEVTVIDYRSL
Solyc01g009090.2.1.sly.36140441                710  ......AGPIIALP...PGFRQDPRRLRSYQDLDAPEDEVTVIDYRSL
Thhalv10001919m.esa.20200705                   676  .GGA...APFL.MS...PAFRQDPRRLRSYQDLDAPEEEVTVIDYRSL
AT2G27100.1.ath.19641299                       680  .GGP...APFL.LS...PAFRQDPRRLRSYQDLDAPEEEVTVIDYRSL
Q9ZVD0                                         680  .GGP...APFL.LS...PAFRQDPRRLRSYQDLDAPEEEVTVIDYRSL
Araha.4729s0005.1.p.aha.28848294               562  .GGP...APFL.LS...PAFRQDPRRLRSYQDLDAPEEEVTVIDYRSL
AL4G20790.t1.aly.35923270                      677  .GGP...APFL.LS...PAFRQDPRRLRSYQDLDAPEEEVTVIDYRSL
Bostr.29505s0043.1.p.adr.30675300              487  .GGP...APFL.LS...PAFRQDPRRLRSYQDLDAPEEEVTVIDYRSL
Carubv10022733m.cru.20903468                   678  .GGP...APFL.LS...PAFRQDPRRLRSYQDLDAPEEEVTVIDYRSL
AL4G20820.t1.aly.35924196                      676  .GGP...APFL.LS...PAFRQDPRRLRSYQDLDAPEEEVTVIDYRSL
Carubv10022734m.cru.20901991                   677  .GGP...APFL.LS...PAFRQDPRRLRSYQDLDAPEEEVTVIDYRSL
Bostr.28794s0002.1.p.adr.30657126              508  .GGP...APFL.LS...PAFRQDPRRLRSYQDLDAPEEEVTVIDYRSL
Brara.D01611.1.p.bra.30620383                  650  .GGP...APFL.LS...PAFRQDPRRLRSYQDLDAPEEEVTVIDYRSL
Bol026461.bol.37343291                         646  .GGP...APFL.LS...PAFRQDPRRLRSYQDLDAPEEEVTVIDYRSL
Brara.G01299.1.p.bra.30635851                  594  .GGP...APFL.LS...PAFRQDPRRLRSYQDLDAPEEEVTVIDYRSL
Bol027793.bol.37343994                         662  .GGP...APFL.LS...PAFRQDPRRLRSYQDLDAPEEEVTVIDYRSL
mrna07673.1-v1.0-hybrid.fve.27266960           694  .SGP...APILALS...PAFRQDPRRLRSYQDLDAPEDEVTVIDYRSL
Prupe.6G153800.1.p.ppe.32086540                710  .SGP...APILALS...PAFRQDPRRLRSYQDLDAPEDEVTVIDYRSL
GSVIVT01037454001.vvi.17842075                 695  .SGP...APVLALS...PGLRQDPRRLRSYQDLDAPEDEVTVIDYRSL
orange1.1g003865m.csi.18122801                 711  .SGP...GPIL.LS...PAFRQDPRRIRSYQDLDAPEDEVTVMDYRSL
Gorai.004G227500.1.gra.26773739                692  .SGP...APFL.LP...PGFRQDPRRLRSYQDLDAPEDEVTVIDYRSL
Thecc1EG042011t1.tca.27426134                  692  .SGP...APFL.LP...PGFRQDPRRLRSYQDLDAPEDEVTVIDYRSL
evm.model.supercontig_103.67.cpa.16404954      500  .SGP...APFL.LS...PAFRQDPRRLRSYQDLDAPEDEVTVIDYRSL
Potri.004G196400.1.ptr.26990875                711  .GGP...APIL.LS...PAFRQDPRRIRSYQDLDVPEDEVTVIDYRSL
SapurV1A.0615s0230.1.p.spu.31434857            713  .GGP...APIL.LS...PAFRQDPRRIRSYQDLDVPEDEVTVIDYRSL
29942.m000751.rco.16814310                     713  .SGP...APIL.LS...PAFRQDPRRIRSYQDLDAPDDEVTVIDYRSL
Manes.11G111400.1.p.mes.32355670               705  .NGP...APIL.LS...PAFRQDPRRIRSYQDLDAPEDEVTVIDYRSL
Manes.S021700.1.p.mes.32354619                 703  .SGP...APIL.LS...PAFRQDPRRIRSYQDLDAPEDEVTVIDYRSL
Eucgr.J00624.1.p.egr.32033287                  699  .SGP...APIIASF...PGFRQDPRRLRSYQDLDAPEDEVTVIDYRSL
GSVIVT01027348001.vvi.17834761                 684  .GGP...APILGVS...SAFRQDPRRLRSYQDLDAPEDEVTVIDYRSL
Kalax.0225s0069.1.p.kla.32599097               689  .GGP......LLLS...PAFRQDPRRLRSYNDLDAPEDEVTVIDYRSL
Kaladp0081s0182.1.p.kfe.35761984               689  .GGP......LLLS...PAFRQDPRRLRSYNDLDAPEDEVTVIDYRSL
Kalax.0216s0065.1.p.kla.32585583               685  .GGP......LLLS...PAFRQDPRRLRSYNDLDAPEDEVTVIDYRSL
DCAR_002130.dca.36055141                       674  .GGP...APIIALP...PQFRQDPRRLRRYDDLDAPEDEVTVIDYRSL
Aqcoe2G007600.1.p.aco.33056562                 676  .SGP...GPMLGVS...PAFRQDPRRIRSYQDLDAPEDEVTVIDYRSL
Bradi3g03840.1.p.bdi.32813176                  652  ...P...RPMMPMY...PHFPRDPRPLRNYDDLDAPDEEVTVIDYRSL
Brast04G302900.1.p.bst.32866594                725  ...P...RPMMPMY...PHFPRDPRPLRNYDDLDAPDEEVTVIDYRSL
Sevir.1G087000.1.p.svi.32667668                662  .L.....GPTLPMY...PSFPLGRRTYRSYEDLDAPKEEIAAIDSRRL
Pavir.J22990.1.p.pvi.30308347                  653  .L.....GPMLPMY...PSFPLDIHTYRSYKDLDAPKEEIAALDLRRL
Pahal.A00287.1.pha.32525941                    645  .L.....GPMLPMH...PSFPLDSRTYRSYKDLDAPKEEITAVDLRRL
OAV97825.1                                     908  .SAA..FSPAVAAAANPPPMASDPRSVLAYNDLDTPAGDEVVLNY...
XP_003293253.1                                 774  .SSARYPYPNQSRH...STSSSDPRGIREYVDLDAPMDHVPEIDYRSA
XP_005713144.1                                 681  .DGR................PADPRARRAYNDLDAPSNSFDLVRYEDV
XP_005702831.1                                 661  .SGM................HSDPRAPRHYADLDAPPQGGPSVDT..L
XP_009857252.1                                 848  GDGA...AGNAAMG...PREAVQGRTIKSYEDLDATAGNGGELNY...
GES64941.1                                     812  GAGS....AQQGMG...PREAVQGRSLKSYEDLDAVGGGSGELNY...
KEY79445.1                                     868  GSGP....GQQGMG...PREATQGRSIKSYEDLDAVGGGSGELNY...
XP_008869116.1                                 618  .................RPLPVDPRQVSTYQDLDSIHTPAVKLDFQDA
Q66I22                                         859  .QGG...YPGKPRN..SRVMRGDPRNIIEYRDLDAPDD....VDF...
RXN03320.1                                     796  .QGG...YPGKPRN..SRMMRGDPRNIIEYRDLDAPDD....VDF...
XP_015195966.1                                 732  .QGG...YPGKPRN..NRMIRGDPRAIIEYRDLDAPDD....VDF...
B1H1X4                                         813  .QGM...YPGKPRN...RMMRGDPRSIIEYRDLDAPDD....VDF...
NP_001362205.1                                 812  .QGM...YPGKPRN...RMIRGDPRAIIEYRDLDAPDD....VEF...
Q99MR6                                         839  .QGG...YPGKPRN...RMVRGDPRAIVEYRDLDAPDD....VDF...
Q9BXP5                                         840  .QGG...YPGKPRN...RMVRGDPRAIVEYRDLDAPDD....VDF...
XP_005549330.1                                 809  .QGG...YPGKPRN...RMVRGDPRAIVEYRDLDAPDD....VDF...
H2ZUZ4                                         794  .QGG...YPGKPRN...RMARGDPRAIVEYRDLDAPED....VDF...
XP_001748332.1                                 918  .RAP...APAPTLPPGLPTMDWDWRSIRAFAALSMIIGSAAGVNFKKS
XP_002286542.1                                 725  EAAARAELEHQRRQ...EEMAGEKRK...FVDVDDMVEEKVELSFENV
XP_649316.1                                    620  .QQPQYYTPNQPGY...NPYQQVPRN...NNPMNQPPQQYGYAPQQGF
consensus>70                                        ..g.....p........p....dpr..rsyqdldapedevtvidyrsl
```
