# Supplementary material for: Molecular Evolution and Diversification of Proteins Involved in miRNA Maturation Pathway
Source: Plants (Basel). 2020 Mar 1;9(3):299. doi: 10.3390/plants9030299 (PMC7154892; doi:10.3390/plants9030299)
Supplement: Supplementary file 1 [file plants-09-00299-s001.zip › Supplementary Files/Supplementary File S3.pdf]

```
                                                                      1        10        20        30
Aco012726.1_Acomosus_TGH                    ...........................SFAGADYVVYGTPIER.A.GQLRWKQEPSLFLS
A0A199V6F8_Acomosus_TGH                     ................................DYVVYGTPIER.A.GQLRWKQE......
M0TMB2_Macuminata_TGH                       ................................DFVFYGTPIER.A.GQLRWKQE......
A0A2H3XE04_Pdactylifera_TGH                 ................................DYLFYGTPIER.A.GQLRWKQE......
A0A0Q3H630_Bdistachyon_TGH                  ................................DLVVYGTPIER.A.GQLRWKQE......
J3MEA3_Obrachyantha_TGH                     ................................DLVVYGTPIER.A.GQLRWKQE......
B8B2G4_Osativa_TGH                          ................................DLVVYGTPIER.A.GQLRWKQE......
K3XV17_Sitalica_TGH                         ................................DLVIYGTPIER.A.GQLRWKQE......
Q8GXN9_Athaliana_TGH                        ................................DFVFHGTPIER.SSGNLRWKQE......
Bol036258_Boleraceacapitata_TGH             ................................DFVFHGTPIER.SSGNLRWKQE......
V4MKB5_Esalsugineum_TGH                     ................................DFVFHGTPIER.SSGNLRWKQE......
A0A087GCN0_Aalpina_TGH                      ................................DFVFHGTPIER.SSGNLKWKQE......
A0A151SPT8_Ccajan_TGH                       ................................DFVFYGTPIER.SSGQLRWKQE......
I1K185_Gmax_TGH                             ................................DFVFYGTPIER.SSGQLRWKQE......
V7CD35_Pvulgaris_TGH                        ............................................................
A0A1S3UTP2_Vradiata_TGH                     ................................DFVFYGTPIER.SSGQLRWKQE......
A0A1S2YR44_Carietinum_TGH                   ................................DFVFYGTPIER.SAGQLRWKQE......
G7JT16_Mtruncatula_TGH                      ................................DFVFFGTPIER.SSGQLRWKQE......
A0A1U8FWC2_Cannuum_TGH                      ................................DFVFYGTPIER.SSGQLRWKQE......
V4TW69_Cclementina_TGH                      ................................DYVFFGTPIER.SSGHLRWKQE......
A0A1R3G1K6_Ccapsularis_TGH                  ................................DYVFFGTPIER.NSGNLRWKQE......
A0A1R3H0J1_Colitorius_TGH                   ................................DYVFFGTPIER.NSGNLRWKQE......
A0A0D2QRF0_Graimondii_TGH                   ................................DYVFFGTPIER.SSGNLRWKQE......
A0A067JJA6_Jcurcas_TGH                      ................................DFVFYGTPIER.SSGHLRWKQE......
B9RE60_Rcommunis_TGH                        ................................DFVFYGTPIER.SSGHLRWKQE......
SapurV1A.0789s0080.1_SSpurpurea_TGH         ................................DFVFYGTPIQR.SSGHLRWKQE......
A0A2I4E4A1_Jregia_TGH                       ................................DYVFYGTPIER.SSGHLRWKQE......
F6GTG8_Vvinifera_TGH                        ................................DYVFYGTPIER.SSGLLRWKQE......
W9S666_Mnotabilis_TGH                       ................................DYVFYGTPIER.SSGQLRWKQE......
A0A068TQE9_Ccanephora_TGH                   ................................DFVFYGTPIER.SSGQLRWKQE......
A0A200Q3N1_Mcordata_TGH                     ................................DFVFYGTPIQR.SAGQLRWKQE......
A0A1U8AEE6_Nnucifera_TGH                    ................................DFVFYGTPIER.STGQMRWKQE......
A0A0A0LSK9_Csativus_TGH                     ................................DFVFYGTPIER.SSGTMRWKQE......
A0A059A5E4_Egrandis_TGH                     ................................DFVFHGTPIER.SSGNLRWKQE......
A0A218XZU2_Pgranatum_TGH                    ................................DFVFYGTPVER.SSGNLRWKQE......
A0A022RKT3_Eguttata_TGH                     ................................DFVFFGKPIER.TSGQLRWKQE......
A0A0J8B830_Bvulgaris_TGH                    ................................DFVFVGTPIER.SSSQLRWKQE......
Kalax.0309s0034.1_Klaxiflora_TGH            ................................DFVFLGTPIER.SSGTLRWNQE......
A0A124SAU9_Ccardunculus_TGH                 ................................DFVFFGTPIER.SSGQLRWKQE......
W1PSA4_Atrichopoda_TGH                      ................................DYIFFGTPIER.G.GQMRWKQE......
A0A0K9NIC3_Zmarina_TGH                      ................................DFIYIGTPIGR.A.GQIRWKQE......
A0A2C9VXU1_Mesculenta_TGH                   ................................................MR..........
A0A0U9HQK1_Knitens_TGH                      ................................DYFFFGTPLPE.P.GQLKWKQE......
Sphfalx0151s0047.1_SSfallax_TGH             ................................DYVVYGTPLER.P.GQARWNQE......
D8QX10_Smoellendorffii_TGH                  ................................ALVVYGTPLEN.A.GTLRWKQD......
E1ZI32_Cvariabilis_TGH                      ................................DFHFFGTEIEDLG.SATRHKQE......
C1FFR5_Mcommoda_TGH                         ................................DFIVVGTPLEQ...AVTKHKQE......
A0A086T442_Achrysogenum_TGH                 .............................KRSPFATFGTPLPD.......WKQE......
K3V9N2_Fpseudograminearum_TGH               MATGN........................KRSPFATFGTPLPD.......WKQE......
W7LTD5_Gmoniliformis_TGH                    .............................KRSPFATFGTPLPD.......WKQE......
G9NMW3_Hatroviridis_TGH                     .............................KRTPYAIFGTPLPE.......WKQE......
A0A0G0A2Q9_Tharzianum_TGH                   .............................KRSPYAIFGTPLPE.......WKQE......
A0A1T3CLH2_Tguizhouense_TGH                 .............................KRSPYAIFGTPLPE.......WKQE......
A0A084QRJ1_Schlorohalonata_TGH              .............................KRSPYAVFGTPLPD.......WKQE......
J4KPK4_Bbassiana_TGH                        .............................KRSPYAAFGTPLPD.......WKQD......
A0A2H4SR46_Cmilitaris_TGH                   .............................KRSPYAVFGTPLPD.......WKQD......
A0A167VSL6_Cfumosorosea_TGH                 .............................KRSPYALFGTPLPD.......WKQD......
A0A179G8E9_Pchlamydosporia_TGH              .............................KRSPYALFGTPLPD.......WKQD......
A0A0L0MXB1_Tophioglossoides_TGH             .............................KRSPYAVFGTPLPD.......WKQD......
Q2GSN0_Cglobosum_TGH                        ...............YRTN..........KRSPYVAFGTPLPP.......WKQE......
G2QES8_Mthermophila_TGH                     .............................KRSPYVAFGTPLPP.......WKQE......
A0A175VUX4_Mmycetomatis_TGH                 .............................KRSPYVLFGTPLPP.......WKQE......
G2QRS6_Tterrestris_TGH                      .............................KRSPFVFYGTPLPP.......WKQE......
B2ARI9_Panserina_TGH                        .............................KRSPYVFFGTPLPP.......WKQE......
F7W019_Smacrospora_TGH                      .............................KRSPYVFFGTPLPP.......WKQE......
A0A1J7J5C9_Cligniaria_TGH                   .............................KRSPFVLYGTPLPP.......WKQE......
A0A0G2IFE8_Dampelina_TGH                    .............................KRTPYVAYGTPLPP.......WKQE......
A0A194W011_Vmali_TGH                        .............................KRSPFVIYGTPLPP.......WKQE......
R8BKG4_Tminima_TGH                          .............................KRSPFVLFGTPLPP.......WKQE......
G0SA63_Cthermophilum_TGH                    .............................KRSPYVFFGTPLPP.......WKQE......
J3NFM2_Ggraminis_TGH                        .............................KRPPFVLFGTPLPP.......WKQE......
A0A0C4DZ99_Mpoae_TGH                        .............................KRPPFVLFGTPLPP.......WKQE......
L7IH22_Moryzae_TGH                          .............................KRSPFVVFGTPLPP.......WKQE......
A0A1Y2WZC5_Daldiniasp._TGH                  .............................KRSPYVLFGTPLPP.......WKQE......
A0A1Y2V8J1_Hypoxylon                        .............................KRSPYVLFGTPLPP.......WKQE......
A0A1Y2W1E2_Hypoxylon                        .............................KRSPYVTFGTPLPP.......WKQE......
A0A1W2TFH3_Rnecatrix_TGH                    .............................KRNPFVLFGTPLPP.......WQQE......
W3WZ77_Pfici_TGH                            .............................KRSPYVLFGTPLPP.......WKQE......
A0A1Y2DPV9_Pvexata_TGH                      .............................KRTPYVVFGTPLPP.......WKQE......
A0A136JHX1_Mbolleyi_TGH                     .............................KRSPYVAYGTPLPP.......WKQE......
S3CR73_Glozoyensis_TGH                      .............................KRSPFVTYGTPLPP.......WKQE......
K1WET1_Mbrunnea_TGH                         ......SKDEITFAR......SMQADL..KRSPFVAFGTPLPP.......WKQE......
A0A218YYN9_Mcoronariae_TGH                  .............................KRSPFVVFGTPLPP.......WKQE......
A0A194XKL9_Pscopiformis_TGH                 .............................KRSPFVTFGTPLPP.......WKQE......
A0A0C3DJQ0_Omaius_TGH                       .............................KRSPFVVFGTALPP.......WKQE......
A7ECA3_Ssclerotiorum_TGH                    .............................KRSPFVVYGTPLPP.......WKQE......
W9CY85_Sborealis_TGH                        .............................KRSPFVVYGTPLPP.......WKQE......
A0A094E7L1_Pseudogymnoascus                 .............................KRTPFVAYGTPLPP.......WKQE......
A0A1B8CGM9_Pseudogymnoascus                 .............................KRTPFVAYGTPLPP.......WKQE......
A0A094H174_Pseudogymnoascus                 .............................KRTPFVAYGTPLPP.......WKQE......
Q0D178_Aterreus_TGH                         .............................KRSPFAFYGTPLPP.......WKQE......
A0A1L9NNK2_Atubingensis_TGH                 .............................KRSPYALYGTPLPP.......WKQE......
A1CQ12_Aclavatus_TGH                        .............................KRSPFAFYGTPLPP.......WKQE......
A0A1L9RVU1_Awentii_TGH                      .............................KRPPYAVYGTPLPP.......WKQE......
A0A1E3BNS4_Acristatus_TGH                   .............................KRTPYAVYGTPLPP.......WKQE......
I7ZRW9_Aoryzae_TGH                          .............................KRAPYAFYGTPLPP.......WKQE......
A0A0F8UC36_Aochraceoroseus_TGH              .............................KRTPYAFFGTPLPP.......WKQE......
Q5BE58_Enidulans_TGH                        .............................KRTPFVFYGTALPP.......WKQE......
A0A0A2KYD8_Pitalicum_TGH                    .............................KRTPYAFYGTPLPP.......WKQE......
A0A1V6QJR5_Pantarcticum_TGH                 .............................KRTPYAFYGTPLPP.......WKQE......
A0A1Q5UD44_Psubrubescens_TGH                .............................KRPPYAFYGTPLPP.......WKQE......
A0A1V6P0U0_Pdecumbens_TGH                   .............................KRPPYVYYGTPLPP.......WKQE......
A0A0F4YXS3_Remersonii_TGH                   .............................KRSPYVFYGTPLPP.......WKQE......
```

```
B8LVN1_Tstipitatus_TGH             .............................KRSPYVFYGTPLPP.......WKQE......
A0A225B272_Tatroroseus_TGH         .............................KRSPYAFYGTPLPP.......WKQE......
A0A0H1BJD0_Bsilverae_TGH           .............................KRSPYAVYGTPLPP.......WKQE......
A0A1B7NTS8_Esp._TGH                .............................KRSPYAIYGTPLPP.......WKQE......
C0RYG0_Pbrasiliensis_TGH           .............................KRSPYAVYGTPLPP.......WKQE......
C4JTX0_Ureesii_TGH                 .............................KRSPFVFYGTPLPA.......WKQE......
R7Z059_Capollinis_TGH              .............................KRNPYVFYGTPLPP.......WKQE......
A0A1S8BM68_Dseriata_TGH            .............................KRSPYAVVGTPLPP.......WKQE......
K2RXL0_Mphaseolina_TGH             .....W.............GA........KRSPYVFVGTPLPP.......WKQE......
A0A165JKY3_Xheveae_TGH             .............................KRSPYVLYGTALPP.......WKQE......
A0A0G2GV44_Pchlamydospora_TGH      .............................KRNPYAFYGTPLPP.......WKQE......
F0X9I8_Gclavigera_TGH              .............................KRSPFVSFGTPLPP.......WKQE......
S3BTH5_Opiceae_TGH                 .............................KRSPYVTFGTPLPP.......WKQE......
A0A0C2J2D4_Sbrasiliensis_TGH       .............................KRSPYVAFGSALPP.......WKQE......
A0A167VN43_Sinsectorum_TGH         .............................KRSPFVAFGTPLPP.......WKQE......
F7W020_Smacrospora_TGH             .............................DRVP..IFGHPPPQ.................
A0A151N705_Amississippiensis_TGH   .............................AGS..VSYGIALRP.......QEQT......
A0A286X9R8_Cporcellus_TGH          ..................................VSYGTGLEP.......QDQT......
A0A1S3ARH5_Eeuropaeus_TGH          ..................................VSYGKGLEP.......QDQT......
Q9BRR8_Hsapiens_TGH                ..................................VSYGTGLEP.......QDQT......
Q9DBM1_Mmusculus_TGH               ..................................ISYGTGLEP.......QDQT......
L5LZ46_Mdavidii_TGH                ..................................VSYGTGLEP.......QDQT......
S7PC93_Mbrandtii_TGH               ..................................IYHGLQICA.......LDQT......
A0A1S3FZ16_Dordii_TGH              ..................................VSYGTGLEP.......QDQT......
F6Z6Q3_Mdomestica_TGH              ..................................VTYGTGLEP.......QDQT......
G3WTW2_Sharrisii_TGH               ..................................VTYGTGLEP.......QDQT......
Q21827_Celegans_TGH                ..................................................HEEI......
Q9VUA0_Dmelanogaster_TGH           ...................................RFGTPLPA.......EDQI......
```

```
                                                 40        50        60        70        80        90
Aco012726.1_Acomosus_TGH                SPPSRCLFGPASAARRSTSSAAGRGNEIPSLKSRSAINSTLLILPHKESSLKSTTFVKQG
A0A199V6F8_Acomosus_TGH                 ............................................................
M0TMB2_Macuminata_TGH                   ............................................................
A0A2H3XE04_Pdactylifera_TGH             ............................................................
A0A0Q3H630_Bdistachyon_TGH              ............................................................
J3MEA3_Obrachyantha_TGH                 ............................................................
B8B2G4_Osativa_TGH                      ............................................................
K3XV17_Sitalica_TGH                     ............................................................
Q8GXN9_Athaliana_TGH                    ............................................................
Bo1036258_Boleraceacapitata_TGH         ............................................................
V4MKB5_Esalsugineum_TGH                 ............................................................
A0A087GCN0_Aalpina_TGH                  ............................................................
A0A151SPT8_Ccajan_TGH                   ............................................................
I1K185_Gmax_TGH                         ............................................................
V7CD35_Pvulgaris_TGH                    ............................................................
A0A1S3UTP2_Vradiata_TGH                 ............................................................
A0A1S2YR44_Carietinum_TGH               ............................................................
G7JT16_Mtruncatula_TGH                  ............................................................
A0A1U8FWC2_Cannuum_TGH                  ............................................................
V4TW69_Cclementina_TGH                  ............................................................
A0A1R3G1K6_Ccapsularis_TGH              ............................................................
A0A1R3H0J1_Colitorius_TGH               ............................................................
A0A0D2QRF0_Graimondii_TGH               ............................................................
A0A067JJA6_Jcurcas_TGH                  ............................................................
B9RE60_Rcommunis_TGH                    ............................................................
SapurV1A.0789s0080.1_SSpurpurea_TGH     ............................................................
A0A2I4E4A1_Jregia_TGH                   ............................................................
F6GTG8_Vvinifera_TGH                    ............................................................
W9S666_Mnotabilis_TGH                   ............................................................
A0A068TQE9_Ccanephora_TGH               ............................................................
A0A200Q3N1_Mcordata_TGH                 ............................................................
A0A1U8AEE6_Nnucifera_TGH                ............................................................
A0A0A0LSK9_Csativus_TGH                 ............................................................
A0A059A5E4_Egrandis_TGH                 ............................................................
A0A218XZU2_Pgranatum_TGH                ............................................................
A0A022RKT3_Eguttata_TGH                 ............................................................
A0A0J8B830_Bvulgaris_TGH                ............................................................
Kalax.0309s0034.1_Klaxiflora_TGH        ............................................................
A0A124SAU9_Ccardunculus_TGH             ............................................................
W1PSA4_Atrichopoda_TGH                  ............................................................
A0A0K9NIC3_Zmarina_TGH                  ............................................................
A0A2C9VXU1_Mesculenta_TGH               ............................................................
A0A0U9HQK1_Knitens_TGH                  ............................................................
Sphfalx0151s0047.1_SSfallax_TGH         ............................................................
D8QX10_Smoellendorffii_TGH              ............................................................
E1ZI32_Cvariabilis_TGH                  ............................................................
C1FFR5_Mcommoda_TGH                     ............................................................
A0A086T442_Achrysogenum_TGH             ............................................................
K3V9N2_Fpseudograminearum_TGH           ............................................................
W7LTD5_Gmoniliformis_TGH                ............................................................
G9NMW3_Hatroviridis_TGH                 ............................................................
A0A0G0A2Q9_Tharzianum_TGH               ............................................................
A0A1T3CLH2_Tguizhouense_TGH             ............................................................
A0A084QRJ1_Schlorohalonata_TGH          ............................................................
J4KPK4_Bbassiana_TGH                    ............................................................
A0A2H4SR46_Cmilitaris_TGH               ............................................................
A0A167VSL6_Cfumosorosea_TGH             ............................................................
A0A179G8E9_Pchlamydosporia_TGH          ............................................................
A0A0L0MXB1_Tophioglossoides_TGH         ............................................................
Q2GSN0_Cglobosum_TGH                    ............................................................
G2QES8_Mthermophila_TGH                 ............................................................
A0A175VUX4_Mmycetomatis_TGH             ............................................................
G2QRS6_Tterrestris_TGH                  ............................................................
B2ARI9_Panserina_TGH                    ............................................................
F7W019_Smacrospora_TGH                  ............................................................
A0A1J7J5C9_Cligniaria_TGH               ............................................................
A0A0G2IFE8_Dampelina_TGH                ............................................................
A0A194W011_Vmali_TGH                    ............................................................
R8BKG4_Tminima_TGH                      ............................................................
G0SA63_Cthermophilum_TGH                ............................................................
J3NFM2_Ggraminis_TGH                    ............................................................
A0A0C4DZ99_Mpoae_TGH                    ............................................................
L7IH22_Moryzae_TGH                      ............................................................
A0A1Y2WZC5_Daldiniasp._TGH              ............................................................
A0A1Y2V8J1_Hypoxylon                    ............................................................
A0A1Y2W1E2_Hypoxylon                    ............................................................
A0A1W2TFH3_Rnecatrix_TGH                ............................................................
W3WZ77_Pfici_TGH                        ............................................................
A0A1Y2DPV9_Pvexata_TGH                  ............................................................
A0A136JHX1_Mbolleyi_TGH                 ............................................................
S3CR73_Glozoyensis_TGH                  ............................................................
K1WET1_Mbrunnea_TGH                     ............................................................
A0A218YYN9_Mcoronariae_TGH              ............................................................
A0A194XKL9_Pscopiformis_TGH             ............................................................
A0A0C3DJQ0_Omaius_TGH                   ............................................................
A7ECA3_Ssclerotiorum_TGH                ............................................................
W9CY85_Sborealis_TGH                    ............................................................
A0A094E7L1_Pseudogymnoascus             ............................................................
A0A1B8CGM9_Pseudogymnoascus             ............................................................
A0A094H174_Pseudogymnoascus             ............................................................
Q0D178_Aterreus_TGH                     ............................................................
A0A1L9NNK2_Atubingensis_TGH             ............................................................
A1CQ12_Aclavatus_TGH                    ............................................................
A0A1L9RVU1_Awentii_TGH                  ............................................................
A0A1E3BNS4_Acristatus_TGH               ............................................................
I7ZRW9_Aoryzae_TGH                      ............................................................
A0A0F8UC36_Aochraceoroseus_TGH          ............................................................
Q5BE58_Enidulans_TGH                    ............................................................
A0A0A2KYD8_Pitalicum_TGH                ............................................................
A0A1V6QJR5_Pantarcticum_TGH             ............................................................
A0A1Q5UD44_Psubrubescens_TGH            ............................................................
A0A1V6P0U0_Pdecumbens_TGH               ............................................................
A0A0F4YXS3_Remersonii_TGH               ............................................................
```

```
B8LVN1_Tstipitatus_TGH                ............................................................
A0A225B272_Tatroroseus_TGH            ............................................................
A0A0H1BJD0_Bsilverae_TGH              ............................................................
A0A1B7NTS8_Esp._TGH                   ............................................................
C0RYG0_Pbrasiliensis_TGH              ............................................................
C4JTX0_Ureesii_TGH                    ............................................................
R7Z059_Capollinis_TGH                 ............................................................
A0A1S8BM68_Dseriata_TGH               ............................................................
K2RXL0_Mphaseolina_TGH                ............................................................
A0A165JKY3_Xheveae_TGH                ............................................................
A0A0G2GV44_Pchlamydospora_TGH         ............................................................
F0X9I8_Gclavigera_TGH                 ............................................................
S3BTH5_Opiceae_TGH                    ............................................................
A0A0C2J2D4_Sbrasiliensis_TGH          ............................................................
A0A167VN43_Sinsectorum_TGH            ............................................................
F7W020_Smacrospora_TGH                ............................................................
A0A151N705_Amississippiensis_TGH      ............................................................
A0A286X9R8_Cporcellus_TGH             ............................................................
A0A1S3ARH5_Eeuropaeus_TGH             ............................................................
Q9BRR8_Hsapiens_TGH                   ............................................................
Q9DBM1_Mmusculus_TGH                  ............................................................
L5LZ46_Mdavidii_TGH                   ............................................................
S7PC93_Mbrandtii_TGH                  ............................................................
A0A1S3FZ16_Dordii_TGH                 ............................................................
F6Z6Q3_Mdomestica_TGH                 ............................................................
G3WTW2_Sharrisii_TGH                  ............................................................
Q21827_Celegans_TGH                   ............................................................
Q9VUA0_Dmelanogaster_TGH              ............................................................
```

```
                                        100       110           120       130       140
Aco012726.1_Acomosus_TGH                QEDPRVRDEEGR.RRFHGAFTGGFSAG........YYNTAGSKEGWTPQTFTSSRKNR..
A0A199V6F8_Acomosus_TGH                 .....VRDEEGR.RRFHGAFTGGFSAG........YYNTAGSKEGWTPQTFTSSRKNR..
M0TMB2_Macuminata_TGH                   .....VRDEEGR.RRFHGAFTGGFSAG........YYNTVGSKEGWTPQTFSSSRKNR..
A0A2H3XE04_Pdactylifera_TGH             .....VRDEEGR.RRFHGAFTGGFSAG........YYNTVGSKEGWTPQTFTSSRKNR..
A0A0Q3H630_Bdistachyon_TGH              .....VRDEEGR.RRFHGAFTGGFSAG........FYNTAGSKEGWTPQTFTSSRKSR..
J3MEA3_Obrachyantha_TGH                 .....VRDEEGR.RRFHGAFTGGFSAG........YYNTVGTKEGWTPQTFTSSRKNR..
B8B2G4_Osativa_TGH                      .....VRDEEGR.RRFHGAFTGGFSAG........YYNTVGTKEGWTPQTFTSSRKNR..
K3XV17_Sitalica_TGH                     .....VRDEEGR.RRFHGAFTGGFSAG........FYNTVGSKEGWTPQTFTSSRKNR..
Q8GXN9_Athaliana_TGH                    .....VTDEEGR.RRFHGAFTGGYSAG........YYNTVGSKEGWAPQSFTSSRQNR..
Bol036258_Boleraceacapitata_TGH         .....VTDEEGR.RRFHGAFTGGYSAG........YYNTVGSKEGWAPQSFTSSRKNR..
V4MKB5_Esalsugineum_TGH                 .....VTDEEGR.RRFHGAFTGGYSAG........YYNTVGSKEGWAPQSFTSSRKNR..
A0A087GCN0_Aalpina_TGH                  .....VTDEEGR.RRFHGAFTGGYSAG........YYNTVGSKEGWAPQAFTSSRKNR..
A0A151SPT8_Ccajan_TGH                   .....VRDEEGR.RRFHGAFTGGYSAG........YYNTVGSKEGWAPQSFKSSRKNR..
I1K185_Gmax_TGH                         .....VRDEEGR.RRFHGAFTGGYSAG........YYNTVGSKEGWAPQSFKSSRKNR..
V7CD35_Pvulgaris_TGH                    ............................................................
A0A1S3UTP2_Vradiata_TGH                 .....VRDEEGR.RRFHGAFTGGYSAG........YYNTVGSKEGWAPQSFKSSRKNR..
A0A1S2YR44_Carietinum_TGH               .....VRDDEGR.RRFHGAFTGGFSAG........YYNTVGSKEGWAPQTFKSSRKSR..
G7JT16_Mtruncatula_TGH                  .....VRDDEGR.RRFHGAFTGGFSAG........YYNSVGSKEGWEPQTFKSSRKNR..
A0A1U8FWC2_Cannuum_TGH                  .....VTDEEGR.RRFHGAFTGGFSAG........YYNTVGSKEGWTPQTFTSSRKNR..
V4TW69_Cclementina_TGH                  .....VTDEEGR.RRFHGAFTGGFSAG........YYNTVGSKEGWTPQTFTSSRKNR..
A0A1R3G1K6_Ccapsularis_TGH              .....VRDEEGR.RRFHGAFTGGFSAG........YYNTVGSKEGWAPQSFTSSRKNR..
A0A1R3H0J1_Colitorius_TGH               .....VRDEEGR.RRFHGAFTGGFSAG........YYNTVGSKEGWAPQSFTSSRKNR..
A0A0D2QRF0_Graimondii_TGH               .....VRDEEGR.RRFHGAFTGGFSAG........YFNTVGTKEGWAPQSFTSSRKSR..
A0A067JJA6_Jcurcas_TGH                  .....VRDEEGR.RRFHGAFTGGFSAG........YYNTAGSKEGWTPQSFTSSRKNR..
B9RE60_Rcommunis_TGH                    .....VRDEEGR.RRFHGAFTGGYSAG........YYNTVGSKEGWTPQSFTSSRKNR..
SapurV1A.0789s0080.1_SSpurpurea_TGH     .....VRDEEGR.RRFHGAFTGGFSAG........YYNTAGSKEGWTPQTFTSSRKNR..
A0A2I4E4A1_Jregia_TGH                   .....VTDEEGR.RRFHGAFTGGFSAG........YYNTVGSEKGWAPQSFVSSRKNR..
F6GTG8_Vvinifera_TGH                    .....VTDEEGR.RRFHGAFTGGFSAG........FYNTVGSKEGWAPQSFTSSRKNR..
W9S666_Mnotabilis_TGH                   .....VRDEEGR.RRFHGAFTGGYSAG........YFNSVGSKEGWTPQSFVSSRKSR..
A0A068TQE9_Ccanephora_TGH               .....VRDEEGR.RRFHGAFTGGFSAG........YYNTVGSKEGWTPQSFTSSRKSR..
A0A200Q3N1_Mcordata_TGH                 .....VLDDEGR.RRFHGAFTGGFSAG........YYNTVGSKEGWTPQTFTSSRKNR..
A0A1U8AEE6_Nnucifera_TGH                .....VRDEEGR.RRFHGAFTGGFSAG........YYNTAGSKEGWTPRSFTSSRKNR..
A0A0A0LSK9_Csativus_TGH                 .....VRDEEGR.RRFHGAFTGGFSAG........HYNTVGSKEGWTPQSFTSSRKNR..
A0A059A5E4_Egrandis_TGH                 .....VRDEEGR.RRFHGAFTGGFSAG........YYNSVGSKEGWAPQSFTSSRKSR..
A0A218XZU2_Pgranatum_TGH                .....VRDEEGR.RRFHGAFTGGFSAG........YYNTAGSKEGWTPQSFTSSRKNR..
A0A022RKT3_Eguttata_TGH                 .....VTDEEGR.RRFHGAFTGGYSAG........YYNSVGSKEGWTPQTFTSSRKNR..
A0A0J8B830_Bvulgaris_TGH                .....VRDEEGR.RRFHGAFTGGFSAG........FYNTVGSQEGWTPQTFSSSRKSR..
Kalax.0309s0034.1_Klaxiflora_TGH        .....VRDEEGR.RRFHGAFTGGFSAG........YFNTAGSKEGWTPQTYVSSRQNR..
A0A124SAU9_Ccardunculus_TGH             .....VRDEEGR.RRFHGAFSGGFSAG........YFNTVGSKEGWTPQTFTSSRKKR..
W1PSA4_Atrichopoda_TGH                  .....VRDDEGR.KRFHGAFTGGFSAG........YYNTVGSKEGWTPQTFTSSRKNR..
A0A0K9NIC3_Zmarina_TGH                  .....VRDEDGR.RRFHGAFTGGFSAG........YYNTAGSKEGWTPQTFTSSRKNR..
A0A2C9VXU1_Mesculenta_TGH               ............................................................
A0A0U9HQK1_Knitens_TGH                  .....VTDEQGR.KRFHGAFTGGFSAG........FYNTVGSKEGWAPAQFVSSRNQR..
Sphfalx0151s0047.1_SSfallax_TGH         .....VTDSEGR.RRFHGAFSGGFSAG........YFNTVGSKEGWAPQSFKSSRKAR..
D8QX10_Smoellendorffii_TGH              .....ARDSEGR.RRFHGAFTGGFSAG........YFNTVGSKDGWEPKTFKSSRGSR..
E1ZI32_Cvariabilis_TGH                  .....VTDEQGR.RRFHGAFTGGFSAG........YFNTVGSKEGWQPGEFRSSRDSR..
C1FFR5_Mcommoda_TGH                     .....ATDAEGR.RRFHGAFTGGFSAG........YFNTVGSKEGWTPQQFTSSRANR..
A0A086T442_Achrysogenum_TGH             .....VRDERGR.KRLHGAFTGGWSAG........YFNTVGSKEGWTPSKFVSSRTNR..
K3V9N2_Fpseudograminearum_TGH           .....VRDERGR.KRLHGAFTGGWSAG........YFNTVGSKEGWTPSTFVSSR.NK..
W7LTD5_Gmoniliformis_TGH                .....VRDERGR.KRLHGAFTGGWSAG........YFNTVGSKEGWTPSTFVSSRNNR..
G9NMW3_Hatroviridis_TGH                 .....VRDEKGR.RRLHGAFTGGWSAG........YFNTVGSKEGWTPATFVSSRTNR..
A0A0G0A2Q9_Tharzianum_TGH               .....VRDDKGR.RRLHGAFTGGWSAG........YFNTVGSKEGWTPSTFVSSRSNR..
A0A1T3CLH2_Tguizhouense_TGH             .....VRDDKGR.RRLHGAFTGGWSAG........YFNTVGSKEGWTPSTFVSSRSNR..
A0A084QRJ1_Schlorohalonata_TGH          .....VRDERGR.KRLHGAFTGGWSAG........YFNTVGSKEGWTPSTFVSSRSNR..
J4KPK4_Bbassiana_TGH                    .....VRDERGR.KRLHGAFTGGWSAG........YFNTVGSKEGWTPSTFVSSRNSR..
A0A2H4SR46_Cmilitaris_TGH               .....VRDEQGR.KRLHGAFTGGFSAG........YFNTVGSKEGWTPSTFVSSRSKR..
A0A167VSL6_Cfumosorosea_TGH             .....VRDERGR.KRLHGAFTGGWSAG........YFNTVGSKEGWTPSTFVSSRSNR..
A0A179G8E9_Pchlamydosporia_TGH          .....VRDEKGR.RRLHGAFTGGWSAG........YFNTVGSKEGWTPSTFVSSLSNR..
A0A0L0MXB1_Tophioglossoides_TGH         .....VRDERGR.KRLHGAFTGGWSAG........YFNTVGSKEGWSPSTFVSSRTNR..
Q2GSN0_Cglobosum_TGH                    .....VRDERGR.KRLHGAFTG....G........YFNTVGSKEGWTPSTFVSSRTNR..
G2QES8_Mthermophila_TGH                 .....VRDERGR.KRLHGA................YFNTVGSKEGWTPSTFVSSRTNR..
A0A175VUX4_Mmycetomatis_TGH             .....VRDERGR.KRLHGAFTGGWSAG........YFNTVGSKEGWTPSTFVSSRTNR..
G2QRS6_Tterrestris_TGH                  .....VRDERGR.KRFHGAFTGGWSAG........YFNTVGSKEGWTPSSFVSSRTNR..
B2ARI9_Panserina_TGH                    .....VRDERGR.KRLHGAFTGGFSAG........YFNTVGSKEGWMPSTFVSSRTNR..
F7W019_Smacrospora_TGH                  .....VRDERGR.KRLHGAFTGGFSAG........YFNTVGSKEGWTPSRFVSSRTNR..
A0A1J7J5C9_Cligniaria_TGH               .....VIDERGR.KRLHGAFTGGWSAG........YFNTVGSKEGWTPSTFVSSRTNR..
A0A0G2IFE8_Dampelina_TGH                .....VRDERGR.KRLHGAFTGGWSAG........YFNTVGSKEGWTPSTFVSSRSSR..
A0A194W011_Vmali_TGH                    .....VRDERGR.KRLHGAFTGGWSAG........YFNTVGSKEGWTPSTFVSSRTNRKD
R8BKG4_Tminima_TGH                      .....VRDERGR.KRLHGAFTGGWSAG........YFNTVGSKEGWAPSTFVSSRANR..
G0SA63_Cthermophilum_TGH                .....ARDERGR.KR....................YFNTVGSKEGWTPSSFVSSRTKR..
J3NFM2_Ggraminis_TGH                    .....VRDERGR.KRLHGAFTGGFSAG........YFNTVGSKEGWTPATFVSSRTNR..
A0A0C4DZ99_Mpoae_TGH                    .....VRDERGR.KRLHGAFTGGFSAG........YFNTVGSKEGWTPSTFVSSRTNR..
L7IH22_Moryzae_TGH                      .....VRDERGR.KRLHGAFTGGFSAG........YFNTVGSKEGWTPSTFVSSRTNR..
A0A1Y2WZC5_Daldiniasp._TGH              .....VRDERGR.KRLHGAFTGGWSAG........YFNTVGSKEGWTPSTFVSSRTNR..
A0A1Y2V8J1_Hypoxylon                    .....VRDERGR.KRLHGAFTGGWSAG........YFNTVGSKEGWIPSTFVSSRSNR..
A0A1Y2W1E2_Hypoxylon                    .....VRDERGR.KRLHGAFTGGWSAG........YFNTVGSKEGWTPSTFVSSRASR..
A0A1W2TFH3_Rnecatrix_TGH                .....VRDERGR.KRLHGAFTGGWSAG........YFNTVGSKEGWTPSTFVSSRANR..
W3WZ77_Pfici_TGH                        .....VTDDRGR.KRLHGAFTGGWSAG........YFNTVGSKEGWTPSTFVSSRTKR..
A0A1Y2DPV9_Pvexata_TGH                  .....VRDERGR.KRLHGAFTGGFSAG........YFNTVGSKEGWAPSTFVSSRGNR..
A0A136JHX1_Mbolleyi_TGH                 .....VRDEQGR.KRLHGAFTGGWSAG........YFNTVGSKEGWTPSTFVSSRSNR..
S3CR73_Glozoyensis_TGH                  .....VRDERGL.KRLHGAFTGGFSAG........YFNTVGSKEGWTPSTFVSSRTNR..
K1WET1_Mbrunnea_TGH                     .....VRDERGV.KRLHGAFTGGFSAG........YFNTVGSKEGWTPSTFVSSRTNR..
A0A218YYN9_Mcoronariae_TGH              .....VRDERGL.KRLHGAFTGGFSAG........YYNTVGSKEGWTPSTFVSSRTNR..
A0A194XKL9_Pscopiformis_TGH             .....VRDERGL.KRLHGAFTGGFSAG........YYNTVGSKEGWTPSTFVSSRTNR..
A0A0C3DJQ0_Omaius_TGH                   .....VRDERGL.KRLHGAFTGGWSAG........YFNTVGSKEGWTPSTFVSSRSNR..
A7ECA3_Ssclerotiorum_TGH                .....VRDERGL.KRLHGAFTGGFSAG........YYNTVGSKEGWTPSTFVSSRSNR..
W9CY85_Sborealis_TGH                    .....VRDERGL.KRLHGAFTGGFSAG........YYNTVGSKEGWMPSTFVSSRSKR..
A0A094E7L1_Pseudogymnoascus             .....VTDERGR.KRLHGAFTGGFSAG........YFNTVGSKEGWTPSTFVSSRSSR..
A0A1B8CGM9_Pseudogymnoascus             .....VTDERGR.KRLHGAFTGGFSAG........YFNTVGSKEGWTPSTFVSSRSSR..
A0A094H174_Pseudogymnoascus             .....VTDERGR.KRLHGAFTGGFSAG........YFNTVGSKEGWTPSTFVSSRSSR..
Q0D178_Aterreus_TGH                     .....VTDDRGR.KRLHGAFTGGFSAG........YFNTVGSKEGWTPATFVSSRQNR..
A0A1L9NNK2_Atubingensis_TGH             .....VTDDRGR.KRLHGAFTGGFSAG....PIFRYFNTVGSKEGWTPATFVSSRQNR..
A1CQ12_Aclavatus_TGH                    .....VTDDRGR.KRLHGAFTGGFSAG........YFNTVGSKEGWTPATFVSSRQNR..
A0A1L9RVU1_Awentii_TGH                  .....VTDDRGR.KRLHGAFTGGFSAG........YFNSVGSKEGWTPATFVSSRQNR..
A0A1E3BNS4_Acristatus_TGH               .....VTDDRGR.KRLHGAFTGGFSAG........YFNTVGSKEGWTPATFVSSRQNR..
I7ZRW9_Aoryzae_TGH                      .....VTDDRGR.KRLHGAFTGGFSAGLKVVASFRYFNTVGSKEGWTPSTFVSSRQSR..
A0A0F8UC36_Aochraceoroseus_TGH          .....VTDDRGR.KRLHGAFTGGFSAG........YFNTVGSKEGWTPATFVSSRQNR..
Q5BE58_Enidulans_TGH                    .....VTDDRGR.KRLHGAFTGGFSAG........YFNTVGSKEGWTPATFVSSRQNR..
A0A0A2KYD8_Pitalicum_TGH                .....LRDEHGS.KRLHGAFTGGFSAG........YFNSVGSKEGWTPATFVSSRQNR..
A0A1V6QJR5_Pantarcticum_TGH             .....VTDERGR.KRLHGAFTGGFSAG........YFNSVGSKEGWTPATFVSSRQKR..
A0A1Q5UD44_Psubrubescens_TGH            .....VTDERGR.KRLHGAFTGGFSAG........YFNTVGSKEGWTPATFVSSRQNR..
A0A1V6P0U0_Pdecumbens_TGH               .....VTDERGR.KRLHGAFTGGFSAG........YFNSVGSKEGWTPATFVSSRQNR..
A0A0F4YXS3_Remersonii_TGH               .....VTDERGR.KRLHGAFTGGFSAG........YFNTVGSKEGWTPSTFVSSRQNR..
```

```
B8LVN1_Tstipitatus_TGH               .....VTDERGR.KRLHGAFTGGFSAG........YFNTVGSKEGWTPSTFVSSRHNR..
A0A225B272_Tatroroseus_TGH           .....VTDERGR.KRLHGAFTGGFSAG........YFNTVGSKEGWTPSTFVSSRQNR..
A0A0H1BJD0_Bsilverae_TGH             .....VRDERGR.KRLHGAFTGGFSAG........YFNTVGSKEGWTPSTFVSSRRNR..
A0A1B7NTS8_Esp._TGH                  .....VRDDRGR.KRLHGAFTGGFSAG........YFNTVGSKEGWTPSTFVSSRRNR..
C0RYG0_Pbrasiliensis_TGH             .....VRDDWGR.KRLHGAFTGGFSAG........YFNTVGSKEGWTPSTFVSSRRNR..
C4JTX0_Ureesii_TGH                   .....VTDERGR.KRLHGAFTGGFSAG........YFNTVGSKEGWTPSTFVSSRRDR..
R7Z059_Capollinis_TGH                .....VVDERGR.KRLHGAFTGGFSAG........YFNTVGSKDGWTPSTFVSSRASR..
A0A1S8BM68_Dseriata_TGH              .....ATDERGR.KRFHGAFTGGFSAG........YFNSVGSKEGWSPSTFVSSRSNR..
K2RXL0_Mphaseolina_TGH               .....ATDERGR.KRFHGAFTGGFSAG........YFNTVGSKEGWTPSTFVSSRSNR..
A0A165JKY3_Xheveae_TGH               .....VTDERGR.KRLHGAFTGGFSAG........YFNTVGSKEGWTPSTFVSSRTDR..
A0A0G2GV44_Pchlamydospora_TGH        .....VTDERGR.KRLHGAFTGGFSAG........YFNTVGSKEGWTPSTFVSSRQNR..
F0X9I8_Gclavigera_TGH                .....VRDENGR.RRLHGAFTGGWSAG........YYNTVGSKEGWTPSTFVSSRANR..
S3BTH5_Opiceae_TGH                   .....VRDEKGR.RRLHGAFTGGWSAG........YYNTVGSKEGWAPSTFVSSRTNR..
A0A0C2J2D4_Sbrasiliensis_TGH         .....VRDAQGR.RRLHGAFTGGWSAG........YYNTVGSKEGWTPSSFVSSRTNR..
A0A167VN43_Sinsectorum_TGH           .....VRDEKGR.RRLHGAFTGGWSAG........YYNTVGSKEGWTPAPFMSSRTNR..
F7W020_Smacrospora_TGH               ....................................HHHRLGSR........LSLHKRR..
A0A151N705_Amississippiensis_TGH     .....VRDEKGRYKRFHGAFTGGFSAG........YFNTVGSKEGWAPSTFLSSRQKR..
A0A286X9R8_Cporcellus_TGH            .....VRDEKGRYKRFHGAFSGGFSAG........YFNTVGSKEGWTPSTYVSSRQNR..
A0A1S3ARH5_Eeuropaeus_TGH            .....VRDEKGRYKRFHGAFSGGFSAG........YFNTVGSKEGWTPSAFVSSRQNR..
Q9BRR8_Hsapiens_TGH                  .....VRDEKGRYKRFHGAFSGGFSAG........YFNTVGSKEGWTPSTFVSSRQNR..
Q9DBM1_Mmusculus_TGH                 .....VRDEKGRYKRFHGAFSGGFSAG........YFNTVGSKEGWTPSTFVSSRQNR..
L5LZ46_Mdavidii_TGH                  .....VRDEKGRYKRFHGAFSGGFSAG........YFNTVGSKEGWTPSSFVSSRQNR..
S7PC93_Mbrandtii_TGH                 .....VRDEKGRYKRFHGAFSGGFSAG........YFNTVGSKEGWTPSSFVSSRQNR..
A0A1S3FZ16_Dordii_TGH                .....VRDEKGRYKRFHGAFSGGFSAG........YFNTVGSKEGWTPSTFVSSRQNR..
F6Z6Q3_Mdomestica_TGH                .....VRDEKGRYKRFHGAFSGGFSAG........YFNTVGSKEGWTPSTFISSRQNR..
G3WTW2_Sharrisii_TGH                 .....VRDEKGRYKRFHGAFSGGFSAG........YFNTVGSKEGWTPSTFISSRQNR..
Q21827_Celegans_TGH                  .....ATDEKGK.RRFHGAFTGGFSAG........YWNTVGSKQGWVPQVFSSSRNER..
Q9VUA0_Dmelanogaster_TGH             .....VKDENGK.RRFHGAFTGGFSAG........FWNTVGSLEGWTPQTFKSSRGER..
```

```
                                               150
Aco012726.1_Acomosus_TGH              .........SIYNFLDEDDIK.......................................
A0A199V6F8_Acomosus_TGH               .........SIYNFLDEDDIK.......................................
M0TMB2_Macuminata_TGH                 .........SIYNFLDEDDLK.......................................
A0A2H3XE04_Pdactylifera_TGH           .........TIYNFFDEDDIK.......................................
A0A0Q3H630_Bdistachyon_TGH            .........SIYNFLDEEDIK.......................................
J3MEA3_Obrachyantha_TGH               .........SVYSFLDEEDIK.......................................
B8B2G4_Osativa_TGH                    .........SIYSFLDEEDIK.......................................
K3XV17_Sitalica_TGH                   .........SIHSFLDEEDIK.......................................
Q8GXN9_Athaliana_TGH                  .........SISDFLDEDEKA.......................................
Bo1036258_Boleraceacapitata_TGH       .........NISDFLDEDEKA.......................................
V4MKB5_Esalsugineum_TGH               .........SVSDFLDEDERA.......................................
A0A087GCN0_Aalpina_TGH                .........SISDFLDEDEKA.......................................
A0A151SPT8_Ccajan_TGH                 .........NILNFLDEDEKA.......................................
I1K185_Gmax_TGH                       .........NILNFLDEDEKT.......................................
V7CD35_Pvulgaris_TGH                  ...........MNFLDEDEKS.......................................
A0A1S3UTP2_Vradiata_TGH               .........NILNFLDEDEKS.......................................
A0A1S2YR44_Carietinum_TGH             .........SILNFLDEDEKA.......................................
G7JT16_Mtruncatula_TGH                .........NILNFLDDDEKA.......................................
A0A1U8FWC2_Cannuum_TGH                .........SLLNFLDDDEKA.......................................
V4TW69_Cclementina_TGH                .........SILNFLDEDEKA.......................................
A0A1R3G1K6_Ccapsularis_TGH            .........SIFSFLDDDEKA.......................................
A0A1R3H0J1_Colitorius_TGH             .........SIFSFLDDDEKA.......................................
A0A0D2QRF0_Graimondii_TGH             .........SIFNFLDEDEKA.......................................
A0A067JJA6_Jcurcas_TGH                .........SILNFLDDDEKD.......................................
B9RE60_Rcommunis_TGH                  .........NILNFLDDDERA.......................................
SapurV1A.0789s0080.1_SSpurpurea_TGH   .........SMLNFLDEDEKE.......................................
A0A2I4E4A1_Jregia_TGH                 .........SILNFLDEDEKT.......................................
F6GTG8_Vvinifera_TGH                  .........SIFSFLDDDEIA.......................................
W9S666_Mnotabilis_TGH                 .........SIFNFLDEDEKAVAPAALMGVRQRLLGGRMGLEVLIRWENLPEHEASWESY
A0A068TQE9_Ccanephora_TGH             .........SVQDFLDEDEKD.......................................
A0A200Q3N1_Mcordata_TGH               .........SILNFLDDDEKA.......................................
A0A1U8AEE6_Nnucifera_TGH              .........SIFNFLDDDEKA.......................................
A0A0A0LSK9_Csativus_TGH               .........NILNFLDEDEKA.......................................
A0A059A5E4_Egrandis_TGH               .........SILNFLDEDEKV.......................................
A0A218XZU2_Pgranatum_TGH              .........NILNFLDEDEKA.......................................
A0A022RKT3_Eguttata_TGH               .........SIYNFLDEDEKA.......................................
A0A0J8B830_Bvulgaris_TGH              .........SVLDFLDEDEKE.......................................
Kalax.0309s0034.1_Klaxiflora_TGH      .........SILNFLDDDEKA.......................................
A0A124SAU9_Ccardunculus_TGH           .........DLSNFLDDDEKA.......................................
W1PSA4_Atrichopoda_TGH                .........TIYNFLDDDEKV.......................................
A0A0K9NIC3_Zmarina_TGH                .........SLLNFLDEDERE.......................................
A0A2C9VXU1_Mesculenta_TGH             .........AV......DTAS.......................................
A0A0U9HQK1_Knitens_TGH                .........AAEDYMDEDEKE.......................................
Sphfalx0151s0047.1_SSfallax_TGH       .........NAYDFMDEDEKA.......................................
D8QX10_Smoellendorffii_TGH            .........SAYDYMDDEERR.......................................
E1ZI32_Cvariabilis_TGH                .........SVEQYLDEDELE.......................................
C1FFR5_Mcommoda_TGH                   .........TAEDFMDDDERE.......................................
A0A086T442_Achrysogenum_TGH           .........RPEDYMDEEDLA.......................................
K3V9N2_Fpseudograminearum_TGH         .........RPEDYMDEEDLA.......................................
W7LTD5_Gmoniliformis_TGH              .........RPEDYMDEEDLA.......................................
G9NMW3_Hatroviridis_TGH               .........RAEDYMDDEDLA.......................................
A0A0G0A2Q9_Tharzianum_TGH             .........RAEDFMDEEDLA.......................................
A0A1T3CLH2_Tguizhouense_TGH           .........RAEDYMDEEDLA.......................................
A0A084QRJ1_Schlorohalonata_TGH        .........RPEDYMDEEDLA.......................................
J4KPK4_Bbassiana_TGH                  .........RAQDFMDDEDLA.......................................
A0A2H4SR46_Cmilitaris_TGH             .........RAQDFMDDEDLA.......................................
A0A167VSL6_Cfumosorosea_TGH           .........RAQDFMDDEDLA.......................................
A0A179G8E9_Pchlamydosporia_TGH        ....AA...RAEEYMDEEDLA.......................................
A0A0L0MXB1_Tophioglossoides_TGH       .........LADGYMDEEDLA.......................................
Q2GSN0_Cglobosum_TGH                  .........RAEDFMDEEDLA.......................................
G2QES8_Mthermophila_TGH               .........RPEDYMDEEDLA.......................................
A0A175VUX4_Mmycetomatis_TGH           .........RPEDFMDEEDLA.......................................
G2QRS6_Tterrestris_TGH                .........RPEDFMDEEDLA.......................................
B2ARI9_Panserina_TGH                  .........RPEDFMDEEDLA.......................................
F7W019_Smacrospora_TGH                .........RPEDFMDEEDLA.......................................
A0A1J7J5C9_Cligniaria_TGH             .........RPEDFMDEEDLK.......................................
A0A0G2IFE8_Dampelina_TGH              .........RPEDFMDEEDLA.......................................
A0A194W011_Vmali_TGH                  KD.......RLEDFMDEEDLA.......................................
R8BKG4_Tminima_TGH                    .........RPEDFMDEEDLA.......................................
G0SA63_Cthermophilum_TGH              .........RPEDFMDEEDLA.......................................
J3NFM2_Ggraminis_TGH                  .........RPEDFMDDEDFA.......................................
A0A0C4DZ99_Mpoae_TGH                  .........RPEDFMDDEDLA.......................................
L7IH22_Moryzae_TGH                    .........RPEDFMDEEDLA.......................................
A0A1Y2WZC5_Daldiniasp._TGH            .........RPEDFMDEEDLA.......................................
A0A1Y2V8J1_Hypoxylon                  .........RPEDFMDEEDLA.......................................
A0A1Y2W1E2_Hypoxylon                  .........RPEDFMDDEDLA.......................................
A0A1W2TFH3_Rnecatrix_TGH              .........RIEDFMDEEDLA.......................................
W3WZ77_Pfici_TGH                      .........RPEDFMDEEDLA.......................................
A0A1Y2DPV9_Pvexata_TGH                ..NG.....RVEDFMDEEDLA.......................................
A0A136JHX1_Mbolleyi_TGH               .........RPEDFMDDEDLA.......................................
S3CR73_Glozoyensis_TGH                .........RPEDFMDDEDRA.......................................
K1WET1_Mbrunnea_TGH                   .........RPEDFMDEEDIA.......................................
A0A218YYN9_Mcoronariae_TGH            .........RPEDFMDEEDIA.......................................
A0A194XKL9_Pscopiformis_TGH           .........RPEDFMDEEDIA.......................................
A0A0C3DJQ0_Omaius_TGH                 .........RPEDFMDVEDIA.......................................
A7ECA3_Ssclerotiorum_TGH              .........RPEDFMDDEDIA.......................................
W9CY85_Sborealis_TGH                  .........RPEDFMDDEDIA.......................................
A0A094E7L1_Pseudogymnoascus           .........RPEDFMDEEDLA.......................................
A0A1B8CGM9_Pseudogymnoascus           .........RPEDFMDEEDLA.......................................
A0A094H174_Pseudogymnoascus           .........RPEDFMDEEDLA.......................................
Q0D178_Aterreus_TGH                   .........RPEDFMDEEDIR.......................................
A0A1L9NNK2_Atubingensis_TGH           .........RPEDFMDEEDVR.......................................
A1CQ12_Aclavatus_TGH                  .........RPEDFMDEEDIR.......................................
A0A1L9RVU1_Awentii_TGH                .........RPEDFMDEDDIR.......................................
A0A1E3BNS4_Acristatus_TGH             .........RPEDFMDEEDLR.......................................
I7ZRW9_Aoryzae_TGH                    .........RVEDFMDEEDIR.......................................
A0A0F8UC36_Aochraceoroseus_TGH        .........RPEDFMDEEDIR.......................................
Q5BE58_Enidulans_TGH                  .........RAEDFMDEEDIR.......................................
A0A0A2KYD8_Pitalicum_TGH              .........RPEDFMDAEDLR.......................................
A0A1V6QJR5_Pantarcticum_TGH           .........RPEDYMDEDDLR.......................................
A0A1Q5UD44_Psubrubescens_TGH          .........RPEDFMDEEDLR.......................................
A0A1V6P0U0_Pdecumbens_TGH             .........RPEDFMDEEDLK.......................................
A0A0F4YXS3_Remersonii_TGH             .........RPEDFMDEEDLR.......................................
```

```
B8LVN1_Tstipitatus_TGH              .........KIEDFMDEEDIR........................................
A0A225B272_Tatroroseus_TGH          .........RIEDFMDEEDIR........................................
A0A0H1BJD0_Bsilverae_TGH            ......Q..RPEDFMDEEDLR........................................
A0A1B7NTS8_Esp._TGH                 ......QL.RPEDFMDEEDLR........................................
C0RYG0_Pbrasiliensis_TGH            .........RPEDFMDEEDLR........................................
C4JTX0_Ureesii_TGH                  .........RPEDFMDEEDLR........................................
R7Z059_Capollinis_TGH               .........RPEDFMDEEDLA........................................
A0A1S8BM68_Dseriata_TGH             .........RAEDFMDEEDLA........................................
K2RXL0_Mphaseolina_TGH              .........RPEDFMDEEDLA........................................
A0A165JKY3_Xheveae_TGH              .........RREDFMDDEDLA........................................
A0A0G2GV44_Pchlamydospora_TGH       .........RPEDFMDEDDIR........................................
F0X9I8_Gclavigera_TGH               .........RPEDYMDEEDLV........................................
S3BTH5_Opiceae_TGH                  .........RPEDYMDDEDLA........................................
A0A0C2J2D4_Sbrasiliensis_TGH        ........FRPEDYMDEEDLA........................................
A0A167VN43_Sinsectorum_TGH          .........RPEDYMDEEDLA........................................
F7W020_Smacrospora_TGH              ................EED..........................................
A0A151N705_Amississippiensis_TGH    .........GPEDFMDEEDLS........................................
A0A286X9R8_Cporcellus_TGH           .........SPEDFMDEEDLS........................................
A0A1S3ARH5_Eeuropaeus_TGH           .........GPEDFMDEEDLS........................................
Q9BRR8_Hsapiens_TGH                 .........GPEDFMDEEDLS........................................
Q9DBM1_Mmusculus_TGH                .........GPEDFMDEEDLS........................................
L5LZ46_Mdavidii_TGH                 .........GPEDFMDEEDLS........................................
S7PC93_Mbrandtii_TGH                .........GPEDFMDEEDLS........................................
A0A1S3FZ16_Dordii_TGH               .........GPEDFMDEEDLS........................................
F6Z6Q3_Mdomestica_TGH               .........RPEDFMDEEDLS........................................
G3WTW2_Sharrisii_TGH                .........GPEDFMDEEDLS........................................
Q21827_Celegans_TGH                 .........RAEDFMDAEDLG........................................
Q9VUA0_Dmelanogaster_TGH            .........KPEDFMDQEDLG........................................
```

```
Aco012726.1_Acomosus_TGH ............................................................
A0A199V6F8_Acomosus_TGH ............................................................
M0TMB2_Macuminata_TGH ............................................................
A0A2H3XE04_Pdactylifera_TGH ............................................................
A0A0Q3H630_Bdistachyon_TGH ............................................................
J3MEA3_Obrachyantha_TGH ............................................................
B8B2G4_Osativa_TGH ............................................................
K3XV17_Sitalica_TGH ............................................................
Q8GXN9_Athaliana_TGH ............................................................
Bol036258_Boleraceacapitata_TGH ............................................................
V4MKB5_Esalsugineum_TGH ............................................................
A0A087GCN0_Aalpina_TGH ............................................................
A0A151SPT8_Ccajan_TGH ............................................................
I1K185_Gmax_TGH ............................................................
V7CD35_Pvulgaris_TGH ............................................................
A0A1S3UTP2_Vradiata_TGH ............................................................
A0A1S2YR44_Carietinum_TGH ............................................................
G7JT16_Mtruncatula_TGH ............................................................
A0A1U8FWC2_Cannuum_TGH ............................................................
V4TW69_Cclementina_TGH ............................................................
A0A1R3G1K6_Ccapsularis_TGH ............................................................
A0A1R3H0J1_Colitorius_TGH ............................................................
A0A0D2QRF0_Graimondii_TGH ............................................................
A0A067JJA6_Jcurcas_TGH ............................................................
B9RE60_Rcommunis_TGH ............................................................
SapurV1A.0789s0080.1_SSpurpurea_TGH ............................................................
A0A2I4E4A1_Jregia_TGH ............................................................
F6GTG8_Vvinifera_TGH ............................................................
W9S666_Mnotabilis_TGH DVVNAQFPHLEDKVRFWGGGNDKPPVRFTYARRRRRGGFSEGAEQEVESARQPLDERQRQ
A0A068TQE9_Ccanephora_TGH ............................................................
A0A200Q3N1_Mcordata_TGH ............................................................
A0A1U8AEE6_Nnucifera_TGH ............................................................
A0A0A0LSK9_Csativus_TGH ............................................................
A0A059A5E4_Egrandis_TGH ............................................................
A0A218XZU2_Pgranatum_TGH ............................................................
A0A022RKT3_Eguttata_TGH ............................................................
A0A0J8B830_Bvulgaris_TGH ............................................................
Kalax.0309s0034.1_Klaxiflora_TGH ............................................................
A0A124SAU9_Ccardunculus_TGH ............................................................
W1PSA4_Atrichopoda_TGH ............................................................
A0A0K9NIC3_Zmarina_TGH ............................................................
A0A2C9VXU1_Mesculenta_TGH ............................................................
A0A0U9HQK1_Knitens_TGH ............................................................
Sphfalx0151s0047.1_SSfallax_TGH ............................................................
D8QX10_Smoellendorffii_TGH ............................................................
E1ZI32_Cvariabilis_TGH ............................................................
C1FFR5_Mcommoda_TGH ............................................................
A0A086T442_Achrysogenum_TGH ............................................................
K3V9N2_Fpseudograminearum_TGH ............................................................
W7LTD5_Gmoniliformis_TGH ............................................................
G9NMW3_Hatroviridis_TGH ............................................................
A0A0G0A2Q9_Tharzianum_TGH ............................................................
A0A1T3CLH2_Tguizhouense_TGH ............................................................
A0A084QRJ1_Schlorohalonata_TGH ............................................................
J4KPK4_Bbassiana_TGH ............................................................
A0A2H4SR46_Cmilitaris_TGH ............................................................
A0A167VSL6_Cfumosorosea_TGH ............................................................
A0A179G8E9_Pchlamydosporia_TGH ............................................................
A0A0L0MXB1_Tophioglossoides_TGH ............................................................
Q2GSN0_Cglobosum_TGH ............................................................
G2QES8_Mthermophila_TGH ............................................................
A0A175VUX4_Mmycetomatis_TGH ............................................................
G2QRS6_Tterrestris_TGH ............................................................
B2ARI9_Panserina_TGH ............................................................
F7W019_Smacrospora_TGH ............................................................
A0A1J7J5C9_Cligniaria_TGH ............................................................
A0A0G2IFE8_Dampelina_TGH ............................................................
A0A194W011_Vmali_TGH ............................................................
R8BKG4_Tminima_TGH ............................................................
G0SA63_Cthermophilum_TGH ............................................................
J3NFM2_Ggraminis_TGH ............................................................
A0A0C4DZ99_Mpoae_TGH ............................................................
L7IH22_Moryzae_TGH ............................................................
A0A1Y2WZC5_Daldiniasp._TGH ............................................................
A0A1Y2V8J1_Hypoxylon ............................................................
A0A1Y2W1E2_Hypoxylon ............................................................
A0A1W2TFH3_Rnecatrix_TGH ............................................................
W3WZ77_Pfici_TGH ............................................................
A0A1Y2DPV9_Pvexata_TGH ............................................................
A0A136JHX1_Mbolleyi_TGH ............................................................
S3CR73_Glozoyensis_TGH ............................................................
K1WET1_Mbrunnea_TGH ............................................................
A0A218YYN9_Mcoronariae_TGH ............................................................
A0A194XKL9_Pscopiformis_TGH ............................................................
A0A0C3DJQ0_Omaius_TGH ............................................................
A7ECA3_Ssclerotiorum_TGH ............................................................
W9CY85_Sborealis_TGH ............................................................
A0A094E7L1_Pseudogymnoascus ............................................................
A0A1B8CGM9_Pseudogymnoascus ............................................................
A0A094H174_Pseudogymnoascus ............................................................
Q0D178_Aterreus_TGH ............................................................
A0A1L9NNK2_Atubingensis_TGH ............................................................
A1CQ12_Aclavatus_TGH ............................................................
A0A1L9RVU1_Awentii_TGH ............................................................
A0A1E3BNS4_Acristatus_TGH ............................................................
I7ZRW9_Aoryzae_TGH ............................................................
A0A0F8UC36_Aochraceoroseus_TGH ............................................................
Q5BE58_Enidulans_TGH ............................................................
A0A0A2KYD8_Pitalicum_TGH ............................................................
A0A1V6QJR5_Pantarcticum_TGH ............................................................
A0A1Q5UD44_Psubrubescens_TGH ............................................................
A0A1V6P0U0_Pdecumbens_TGH ............................................................
A0A0F4YXS3_Remersonii_TGH ............................................................
```

```
B8LVN1_Tstipitatus_TGH               ............................................................
A0A225B272_Tatroroseus_TGH           ............................................................
A0A0H1BJD0_Bsilverae_TGH             ............................................................
A0A1B7NTS8_Esp._TGH                  ............................................................
C0RYG0_Pbrasiliensis_TGH             ............................................................
C4JTX0_Ureesii_TGH                   ............................................................
R7Z059_Capollinis_TGH                ............................................................
A0A1S8BM68_Dseriata_TGH              ............................................................
K2RXL0_Mphaseolina_TGH               ............................................................
A0A165JKY3_Xheveae_TGH               ............................................................
A0A0G2GV44_Pchlamydospora_TGH        ............................................................
F0X9I8_Gclavigera_TGH                ............................................................
S3BTH5_Opiceae_TGH                   ............................................................
A0A0C2J2D4_Sbrasiliensis_TGH         ............................................................
A0A167VN43_Sinsectorum_TGH           ............................................................
F7W020_Smacrospora_TGH               ............................................................
A0A151N705_Amississippiensis_TGH     ............................................................
A0A286X9R8_Cporcellus_TGH            ............................................................
A0A1S3ARH5_Eeuropaeus_TGH            ............................................................
Q9BRR8_Hsapiens_TGH                  ............................................................
Q9DBM1_Mmusculus_TGH                 ............................................................
L5LZ46_Mdavidii_TGH                  ............................................................
S7PC93_Mbrandtii_TGH                 ............................................................
A0A1S3FZ16_Dordii_TGH                ............................................................
F6Z6Q3_Mdomestica_TGH                ............................................................
G3WTW2_Sharrisii_TGH                 ............................................................
Q21827_Celegans_TGH                  ............................................................
Q9VUA0_Dmelanogaster_TGH             ............................................................
```

```
                                                                                      160
                                                                                        .
Aco012726.1_Acomosus_TGH                 .......................................ALETSMQFDTFG.........
A0A199V6F8_Acomosus_TGH                  .......................................ALETSMQFDTFG.........
M0TMB2_Macuminata_TGH                    .......................................ALETSMQFDTFG.........
A0A2H3XE04_Pdactylifera_TGH              .......................................ALETSLQFDTFG.........
A0A0Q3H630_Bdistachyon_TGH               .......................................ALETSQQYDTFG.........
J3MEA3_Obrachyantha_TGH                  .......................................ALETSQQYDTFG.........
B8B2G4_Osativa_TGH                       .......................................ALETSQQYDTFG.........
K3XV17_Sitalica_TGH                      .......................................ALETSQQYDTFG.........
Q8GXN9_Athaliana_TGH                     .......................................SLSASSQFDTFG.........
Bol036258_Boleraceacapitata_TGH          .......................................SLSASSQFDTFG.........
V4MKB5_Esalsugineum_TGH                  .......................................SLSASSQFDTFG.........
A0A087GCN0_Aalpina_TGH                   .......................................SLSASAQFDTFG.........
A0A151SPT8_Ccajan_TGH                    .......................................LLGTTSQFDTFG.........
I1K185_Gmax_TGH                          .......................................FLGTTSQFDTFG.........
V7CD35_Pvulgaris_TGH                     .......................................FLGTTSQFDTFG.........
A0A1S3UTP2_Vradiata_TGH                  .......................................FLGTTSQFDTFG.........
A0A1S2YR44_Carietinum_TGH                .......................................FLGTSSQFDTFG.........
G7JT16_Mtruncatula_TGH                   .......................................FLGTSSQFDTFG.........
A0A1U8FWC2_Cannuum_TGH                   ........................................LGTSMQYDTFG.........
V4TW69_Cclementina_TGH                   .......................................SFGTSLQFDTFG.........
A0A1R3G1K6_Ccapsularis_TGH               .......................................HLGTSSQFDTFG.....MEVC
A0A1R3H0J1_Colitorius_TGH                .......................................HLGTSSQFDTFG.........
A0A0D2QRF0_Graimondii_TGH                ........................................LGTSSQFDTFG.........
A0A067JJA6_Jcurcas_TGH                   .......................................SLGTSSEFDTFG.........
B9RE60_Rcommunis_TGH                     .......................................SLGTSSQFDTFG.........
SapurV1A.0789s0080.1_SSpurpurea_TGH      .......................................YLGTASQFDTFG.........
A0A2I4E4A1_Jregia_TGH                    .......................................SLGTSMQFDTFG.........
F6GTG8_Vvinifera_TGH                     .......................................SLGTSLQFDTFG.........
W9S666_Mnotabilis_TGH                    EAESARQLLEEGHASKLVKGRE.................SLGTSSQFDTFG.........
A0A068TQE9_Ccanephora_TGH                .......................................SLGTSMQFDTFG.........
A0A200Q3N1_Mcordata_TGH                  .......................................SLGTSLQFDTFG.........
A0A1U8AEE6_Nnucifera_TGH                 .......................................SLGTSLQFDTFG.........
A0A0A0LSK9_Csativus_TGH                  .......................................GLGTSAQFDTFG.........
A0A059A5E4_Egrandis_TGH                  .......................................SLATSSQFDTFG.........
A0A218XZU2_Pgranatum_TGH                 .......................................SLAASSQFDTFG.........
A0A022RKT3_Eguttata_TGH                  .......................................SVGTSMQFDTFG.........
A0A0J8B830_Bvulgaris_TGH                 .......................................LLGTSSQFDTFG.........
Kalax.0309s0034.1_Klaxiflora_TGH         .......................................SLAASMKFDTFG.........
A0A124SAU9_Ccardunculus_TGH              .......................................SLGTSMQFDTFG.........
W1PSA4_Atrichopoda_TGH                   .......................................SLGMTSQFDTFG.........
A0A0K9NIC3_Zmarina_TGH                   .......................................ALETSSKFDTFG.........
A0A2C9VXU1_Mesculenta_TGH                .............................................................
A0A0U9HQK1_Knitens_TGH                   .......................................ALAASADFDTFG.........
Sphfalx0151s0047.1_SSfallax_TGH          .......................................ELGTSSEFDTFG.........
D8QX10_Smoellendorffii_TGH               .......................................QIGTAQEFDTFG.ASFL....
E1Z132_Cvariabilis_TGH                   ......................NLQRGRTACWGCWGTQMSMQTTAEYDTFG.........
C1FFR5_Mcommoda_TGH                      .......................................ALEARDDYDTFG.........
A0A086T442_Achrysogenum_TGH              .......................................KLQTSQAFAGLG.........
K3V9N2_Fpseudograminearum_TGH            .......................................KVQTSQAFAGLG.........
W7LTD5_Gmoniliformis_TGH                 .......................................KVQTSQAFAGLG.........
G9NMW3_Hatroviridis_TGH                  .......................................TLKTTQAFAGLG.........
A0A0G0A2Q9_Tharzianum_TGH                .......................................TLKTTQAFAGLG.........
A0A1T3CLH2_Tguizhouense_TGH              .......................................TLKTTQAFAGLG.........
A0A084QRJ1_Schlorohalonata_TGH           .......................................KIETSQAFAGLG.........
J4KPK4_Bbassiana_TGH                     .......................................QLKTSQAFAGFG.........
A0A2H4SR46_Cmilitaris_TGH                .......................................QLKTTQAFAGFG.........
A0A167VSL6_Cfumosorosea_TGH              .......................................QLKTSQAFAGFG.........
A0A179G8E9_Pchlamydosporia_TGH           .......................................QLQTSQAFAGLG.........
A0A0L0MXB1_Tophioglossoides_TGH          .......................................KLQTSQTFAGLG.........
Q2GSN0_Cglobosum_TGH                     .......................................KIQTQAAFSSLG.........
G2QES8_Mthermophila_TGH                  .......................................KIQTQAAFSGLG.........
A0A175VUX4_Mmycetomatis_TGH              .......................................RIQTVSAFAGLG.........
G2QRS6_Tterrestris_TGH                   .......................................RIQTQASFSGLG.........
B2ARI9_Panserina_TGH                     .......................................RIQTKAAFAGLG.........
F7W019_Smacrospora_TGH                   .......................................KIQTNAAFAGLG.........
A0A1J7J5C9_Cligniaria_TGH                .......................................TIETNAAFAGLG.........
A0A0G2IFE8_Dampelina_TGH                 .......................................KLQTSHAFAGLG.........
A0A194W011_Vmali_TGH                     .......................................RIQTSQAFAGLG.........
R8BKG4_Tminima_TGH                       .......................................KIQTSQAFAGLG.........
G0SA63_Cthermophilum_TGH                 .......................................KLQTREAFSGLG.........
J3NFM2_Ggraminis_TGH                     .......................................KLQTAPDFAAFG.........
A0A0C4DZ99_Mpoae_TGH                     .......................................KLQTAQDFAAFG.........
L7IH22_Moryzae_TGH                       .......................................KLQTNQAFAALG.........
A0A1Y2WZC5_Daldiniasp._TGH               .......................................KLQTAQGFAGLG.........
A0A1Y2V8J1_Hypoxylon                     .......................................KLQTAQGFAGLG.........
A0A1Y2W1E2_Hypoxylon                     .......................................KLQTAQGFAGLG.........
A0A1W2TFH3_Rnecatrix_TGH                 .......................................KLQTAEGFAGLG.........
W3WZ77_Pfici_TGH                         .......................................KLQTADGFAGLG.........
A0A1Y2DPV9_Pvexata_TGH                   .......................................KVRMAGGFAGLG.........
A0A136JHX1_Mbolleyi_TGH                  .......................................KIQTTDSFAGLG.........
S3CR73_Glozoyensis_TGH                   .......................................RVQTAEGFSGLG.........
K1WET1_Mbrunnea_TGH                      .......................................RVQTAEGFAGLG.........
A0A218YYN9_Mcoronariae_TGH               .......................................RVQTAEGFAGLG.........
A0A194XKL9_Pscopiformis_TGH              .......................................RVETTQGFAGLG.........
A0A0C3DJQ0_Omaius_TGH                    .......................................RVQTAAGFAGLG.........
A7ECA3_Ssclerotiorum_TGH                 .......................................RVQTAEGFAGLG.........
W9CY85_Sborealis_TGH                     .......................................RVQTAEGFAGLG.........
A0A094E7L1_Pseudogymnoascus              .......................................RVLTAGDFTALG.........
A0A1B8CGM9_Pseudogymnoascus              .......................................RVLTAGDFTALG.........
A0A094H174_Pseudogymnoascus              .......................................RVLTAGDFTALG.........
Q0D178_Aterreus_TGH                      .......................................NLQTAEDFSGFG.........
A0A1L9NNK2_Atubingensis_TGH              .......................................RLQTADEYSGFG.........
A1CQ12_Aclavatus_TGH                     .......................................NLQTSDEFAGFG.........
A0A1L9RVU1_Awentii_TGH                   .......................................DIHTTDGFSGFG.........
A0A1E3BNS4_Acristatus_TGH                .......................................ELQTSSDFAGFG.........
I7ZRW9_Aoryzae_TGH                       .......................................NLHITDEFSGFG.........
A0A0F8UC36_Aochraceoroseus_TGH           .......................................NLRTTDGFSGFG.........
Q5BE58_Enidulans_TGH                     .......................................NLQTNNEFSGFG.........
A0A0A2KYD8_Pitalicum_TGH                 .......................................QLQTANEFAGFG.........
A0A1V6QJR5_Pantarcticum_TGH              .......................................KLQTADDFTGFG.........
A0A1Q5UD44_Psubrubescens_TGH             .......................................QLQTTEGFAGFG.........
A0A1V6P0U0_Pdecumbens_TGH                .......................................TLQTADGFAGFG.........
A0A0F4YXS3_Remersonii_TGH                .......................................KLQTAEDFAGFG.........
```

```
B8LVN1_Tstipitatus_TGH             ...........................................QLQTSEDFAGLG..........
A0A225B272_Tatroroseus_TGH         ...........................................ELQTSENFSGFG..........
A0A0H1BJD0_Bsilverae_TGH           ...........................................KLQTTGEYAGFG..........
A0A1B7NTS8_Esp._TGH                ...........................................KLQTAGEYSGFG..........
C0RYG0_Pbrasiliensis_TGH           ...........................................KLQTAGDYAGFG..........
C4JTX0_Ureesii_TGH                 ...........................................KLHTTDEFTGIG..........
R7Z059_Capollinis_TGH              ...........................................KLQTASAFAGLG..........
A0A1S8BM68_Dseriata_TGH            ...........................................KLQTSGAFSALG..........
K2RXL0_Mphaseolina_TGH             ...........................................KLQTSNAFSALG..........
A0A165JKY3_Xheveae_TGH             ...........................................RLQTSTSFAGLG..........
A0A0G2GV44_Pchlamydospora_TGH      ...........................................KLSTNEAFSGFG..........
F0X9I8_Gclavigera_TGH              ...........................................KIHTSNAFAGLG..........
S3BTH5_Opiceae_TGH                 ...........................................KLQTTQSFAGLG..........
A0A0C2J2D4_Sbrasiliensis_TGH       ...........................................TIHTSGGFAGLG..........
A0A167VN43_Sinsectorum_TGH         ...........................................RIQTSGAFAGLG..........
F7W020_Smacrospora_TGH             ......................................................................
A0A151N705_Amississippiensis_TGH   ...........................................EITTTDDFA...I.........
A0A286X9R8_Cporcellus_TGH          ...........................................AIVTTDDFA...I.........
A0A1S3ARH5_Eeuropaeus_TGH          ...........................................AIVTTDDFA...I.........
Q9BRR8_Hsapiens_TGH                ...........................................AIVTTDDFA...I.........
Q9DBM1_Mmusculus_TGH               ...........................................AIVTTDDFA...I.........
L5LZ46_Mdavidii_TGH                ...........................................AIVTTDDFA...I.........
S7PC93_Mbrandtii_TGH               ...........................................AIVTTDDFA...I.........
A0A1S3FZ16_Dordii_TGH              ...........................................AIVTTDDFA...I.........
F6Z6Q3_Mdomestica_TGH              ...........................................AIVTTDEFA...I.........
G3WTW2_Sharrisii_TGH               ...........................................AIVTTDDFA...I.........
Q21827_Celegans_TGH                ...........................................SIKQTAAFG...R.........
Q9VUA0_Dmelanogaster_TGH           ...........................................GIRTRDEFANED..........
```

```
                                                    170       180       190
Aco012726.1_Acomosus_TGH                 .............AIVPAANSIGIKLLMKMGWRQGRSIK.....................
A0A199V6F8_Acomosus_TGH                  .......................IKLLMKMGWRQGRSIK.....................
M0TMB2_Macuminata_TGH                    .............AIVPASNSIGVKLLLKMGWRHGHSIK.....................
A0A2H3XE04_Pdactylifera_TGH              .............AIVPAANSIGIKLLLKMGWRHGHSIK.....................
A0A0Q3H630_Bdistachyon_TGH               .............AIVPGTTSIGVKLLMKMGWRQGRTIK.....................
J3MEA3_Obrachyantha_TGH                  .............AIVPATTSIGVKLLMKMGWRQGRSIR.....................
B8B2G4_Osativa_TGH                       .............AIVPATTSIGVKLLMKMGWRQGRSIR.....................
K3XV17_Sitalica_TGH                      .............AIVPTTNSIGVTLLLKMGWRQGRSIR.....................
Q8GXN9_Athaliana_TGH                     .............AIVPVSESIGVKLLLKMGWRRGHSIK.....................
Bol036258_Boleraceacapitata_TGH          .............AIIPVSESVGVKLLLKMGWRRGHSIK.....................
V4MKB5_Esalsugineum_TGH                  .............AIVPVSESIGVKLLLKMGWRRGHSIM.....................
A0A087GCN0_Aalpina_TGH                   .............AIIPVSESIGVKLLLKMGWRRGHSIK.....................
A0A151SPT8_Ccajan_TGH                    .............IIVPATESVGVKLLLKMGWSRSRSIK.....................
I1K185_Gmax_TGH                          .............IIVPATESVGVKLLLKMGWSHGRSIK.....................
V7CD35_Pvulgaris_TGH                     .............IIVPATESVGVKLLLKMGWSRGRTIK.....................
A0A1S3UTP2_Vradiata_TGH                  .............VIVPANESIGVKLLLKMGWSRGRTIK.....................
A0A1S2YR44_Carietinum_TGH                .............VIVPATESIGVKLLLKMGWSRGRSIK.....................
G7JT16_Mtruncatula_TGH                   .............IIVPATESVGVKLLLKMGWSRGRSIK.....................
A0A1U8FWC2_Cannuum_TGH                   .............AIVPVTESIGLTLLQKMGWRRGRSIN.....................
V4TW69_Cclementina_TGH                   .............AIVPATESIGVKLLLKMGWRRGRSIK.....................
A0A1R3G1K6_Ccapsularis_TGH               WL...........AIVPAAESIGVKLLLKMGWRHGRTIK.....................
A0A1R3H0J1_Colitorius_TGH                .............AIVPAAESIGVKLLLKMGWRHGRTIK.....................
A0A0D2QRF0_Graimondii_TGH                .............AIVPASESIGVKLLLKMGWRHGRAIK.....................
A0A067JJA6_Jcurcas_TGH                   .............AIVPAAESIGIKLLLKMGWRHGHSIK.....................
B9RE60_Rcommunis_TGH                     .............AIVPATESIGVKLLLKMGWRHGHSIR.....................
SapurV1A.0789s0080.1_SSpurpurea_TGH      .............AVVPATESIGVKLLLKMGWRHGHSIN.....................
A0A2I4E4A1_Jregia_TGH                    .............AIIPVTESIGVKLLLKMGC...........................
F6GTG8_Vvinifera_TGH                     .............AIVLATESIGVKLLLKMGWRRGNSIK.....................
W9S666_Mnotabilis_TGH                    .............AIIPATVSIGVKLLLKMGWRRGRSIK.....................
A0A068TQE9_Ccanephora_TGH                .............AILPASESIGIKLLLKMGWRRGRSIK.....................
A0A200Q3N1_Mcordata_TGH                  .............AIIPVANSIGVKLLRKMGWRHGQSIK.....................
A0A1U8AEE6_Nnucifera_TGH                 .............AIVPAANSIGVKLLLKMGWRHGHSIR.....................
A0A0A0LSK9_Csativus_TGH                  .............AIIPAAESIGVKLLLKMGWRHGRAIK.....................
A0A059A5E4_Egrandis_TGH                  .............AIIPVTESIGVRLLKKMGWRHGHSIK.....................
A0A218XZU2_Pgranatum_TGH                 .............AIVPATDSVGMNLLKKMGWRYGHSLK.....................
A0A022RKT3_Eguttata_TGH                  .............TIIPVTESIGVKLLLKMGWRQGRSIK.....................
A0A0J8B830_Bvulgaris_TGH                 .............AIIPAAESIGVKLLLKMGWRHGRSIK.....................
Kalax.0309s0034.1_Klaxiflora_TGH         .............AIISATDSIGVKLLLKMGWRHGHSIK.....................
A0A124SAU9_Ccardunculus_TGH              .............AIIPATDPIGVRLMLKMGWRRGHSIK.....................
W1PSA4_Atrichopoda_TGH                   .............AIVPAPNSIGIKLLLKMGWRHGRSIG.....................
A0A0K9NIC3_Zmarina_TGH                   .............VIVPAMNSFGVKLLGKMGWRHGHSIN.....................
A0A2C9VXU1_Mesculenta_TGH                .........................LFIK...............................
A0A0U9HQK1_Knitens_TGH                   .............AIIPSAESMGVKLLKKMGWRPGKGVG.....................
Sphfalx0151s0047.1_SSfallax_TGH          .............AIVPVTESIGVRLLLKMGWRHGRVIG.....................
D8QX10_Smoellendorffii_TGH               .............VIIPPTDSIGVKLLRKMGWRHGRGIG.....................
E1Z132_Cvariabilis_TGH                   .............AIVPVADSVGIRLLQKMGWRQGKGIGVG..................
C1FFR5_Mcommoda_TGH                      ..TGDGGRRKVYGIIVPTSDPVGARLLRLMGWRRGKGIGRGGARNGNGGDGQG.......
A0A086T442_Achrysogenum_TGH              ................PQGDTVGVKLLRKMGWKDGQGIG.....................
K3V9N2_Fpseudograminearum_TGH            ................AEGDTMGLKLLRRMGWKDGQGIG.....................
W7LTD5_Gmoniliformis_TGH                 ................AEGDTMGLKLLRRMGWKDGQGIG.....................
G9NMW3_Hatroviridis_TGH                  ................ADGDTMGLKLLRRMGWKDGQGIG.....................
A0A0G0A2Q9_Tharzianum_TGH                ................ADGDTMGLKLLRRMGWKDGQGIG.....................
A0A1T3CLH2_Tguizhouense_TGH              ................ADGDTMGLKLLRRMGWKDGQGIG.....................
A0A084QRJ1_Schlorohalonata_TGH           ................ATGDSMGTKLLRRMGWKDGQGIG.....................
J4KPK4_Bbassiana_TGH                     ................TEGDTMGLKLLRRMGWKDGQGIG.....................
A0A2H4SR46_Cmilitaris_TGH                ................TDGDTMGLKLLRRMGWKDGQGIG.....................
A0A167VSL6_Cfumosorosea_TGH              ................TEGDTMGLKLLRRMGWKDGQGIG.....................
A0A179G8E9_Pchlamydosporia_TGH           ................SSGDTRGLQLLRRMGWKDGQGIG.....................
A0A0L0MXB1_Tophioglossoides_TGH          ................ADGNTMGLRLLRRMGWKDGQGIG.....................
Q2GSN0_Cglobosum_TGH                     ................ADGDTMGTRLLKKMGWKEGQGIG.....................
G2QES8_Mthermophila_TGH                  ................AEGDTMGTRLLKKMGWKEGQGIG.....................
A0A175VUX4_Mmycetomatis_TGH              ................AEGETMGTKLLKRMGWREGQGIG.....................
G2QRS6_Tterrestris_TGH                   ................VEGETIGTKLLRRMGWKDGQGIG.....................
B2ARI9_Panserina_TGH                     ................ATGETMGEKLLKKMGWKEGQGIG.....................
F7W019_Smacrospora_TGH                   ................TQGETMGVKLLKKMGWREGQGIG.....................
A0A1J7J5C9_Cligniaria_TGH                ................AQGETIGTKLLRRMGWKDGQGVG.....................
A0A0G2IFE8_Dampelina_TGH                 ................VQGETMGTKLLKKMGWKEGQGVG.....................
A0A194W011_Vmali_TGH                     ................VQGETMGTKLLKKMGWREGQGVG.....................
R8BKG4_Tminima_TGH                       ................VEGETMGTKLLKKMGWREGQGIG.....................
G0SA63_Cthermophilum_TGH                 ................VEGETMGVKLLKKMGWKEGQGIG.....................
J3NFM2_Ggraminis_TGH                     ................IEGETMGTKLLKRMGWKEGQGIG.....................
A0A0C4DZ99_Mpoae_TGH                     ................IEGETMGAKLLKRMGWREGQGIG.....................
L7IH22_Moryzae_TGH                       ................VEGETMGTKLLKKMGWKEGQGIG.....................
A0A1Y2WZC5_Daldiniasp._TGH               ................VQGDTMGVKLLRRMGWKDGQGIG.....................
A0A1Y2V8J1_Hypoxylon                     ................AQGETMGVKLLRRMGWKDGQGIG.....................
A0A1Y2W1E2_Hypoxylon                     ................AQGDTMGVKLLRRMGWKDGQGIG.....................
A0A1W2TFH3_Rnecatrix_TGH                 ................VQGETMGVKLLRRMGWKDGQGIG.....................
W3WZ77_Pfici_TGH                         ................VQGDTMGVKLLRRMGWKEGQGIG.....................
A0A1Y2DPV9_Pvexata_TGH                   ................VRGETMGVKLLRKMGWKDGQGIG.....................
A0A136JHX1_Mbolleyi_TGH                  ................TRGETMGVKLLRRMGWKEGQGIG.....................
S3CR73_Glozoyensis_TGH                   ................VEGETIGVKLLKKMGWKEGQGIG.....................
K1WET1_Mbrunnea_TGH                      ................IEGETVGVKLLKKMGWKEGQGVG.....................
A0A218YYN9_Mcoronariae_TGH               ................IEGETIGVKLLKKMGWKEGQGVG.....................
A0A194XKL9_Pscopiformis_TGH              ................VDGETVGVKLLRKMGWKEGQGIG.....................
A0A0C3DJQ0_Omaius_TGH                    ................IEGETMGAKLLKKMGWKEGQGVG.....................
A7ECA3_Ssclerotiorum_TGH                 ................VEGETMGVKLLKKMGWKEGQGVG.....................
W9CY85_Sborealis_TGH                     ................SEGETMGVKLLKKMGWKEGQGVG.....................
A0A094E7L1_Pseudogymnoascus              ................TEGETMGVKLLKKMGWREGQGVG.....................
A0A1B8CGM9_Pseudogymnoascus              ................TEGETMGVKLLKKMGWKEGQGVG.....................
A0A094H174_Pseudogymnoascus              ................TEGETMGVKLLKKMGWREGQGVG.....................
Q0D178_Aterreus_TGH                      ................MGGETMGVKLLKKMGWKEGQGIG.....................
A0A1L9NNK2_Atubingensis_TGH              ................TSGETMGVKLLKRMGWREGQGIG.....................
A1CQ12_Aclavatus_TGH                     ................AGGETMGVKLLKKMGWREGQGIG.....................
A0A1L9RVU1_Awentii_TGH                   ................TGGETMGVKLLKKMGWREGQGIG.....................
A0A1E3BNS4_Acristatus_TGH                ................TGGETMGVKLLKKMGWREGQGIG.....................
I7ZRW9_Aoryzae_TGH                       ................SGGETMGVKLLKRMGWKEGQGVG.....................
A0A0F8UC36_Aochraceoroseus_TGH           ................TTGETIGVKLMKKMGWREGQGIG.....................
Q5BE58_Enidulans_TGH                     ................TSGETVGVKLLKKMGWREGQGIG.....................
A0A0A2KYD8_Pitalicum_TGH                 ................TGGETIGVKLLKKMGWREGQGIG.....................
A0A1V6QJR5_Pantarcticum_TGH              ................TGGETKGIQLLKKMGWREGQGIG.....................
A0A1Q5UD44_Psubrubescens_TGH             ................TSGETMGVKLLKRMGWREGQGIG.....................
A0A1V6P0U0_Pdecumbens_TGH                ................AGGETMGVKLLKRMGWREGQGIG.....................
A0A0F4YXS3_Remersonii_TGH                ................IEGETMGVKLLKKMGWKEGQGIG.....................
```

```
B8LVN1_Tstipitatus_TGH                ................TEGETMGVKLLKKMGWREGQGIG.....................
A0A225B272_Tatroroseus_TGH            ................HEGETMGAKLLKKMGWREGQGIG.....................
A0A0H1BJD0_Bsilverae_TGH              ................TTGDTIGVKLLKRMGWKEGQGIG.....................
A0A1B7NTS8_Esp._TGH                   ................TTGETIGVKLLKRMGWKEGQGIG.....................
C0RYG0_Pbrasiliensis_TGH              ................TAGETMGVKLLKRMGWKEGQGIG.....................
C4JTX0_Ureesii_TGH                    ................PSGETIGVRLLKRMGWKDGQGVG.....................
R7Z059_Capollinis_TGH                 ................AGGDTVGVKLLQRMGWRQGQGVG.....................
A0A1S8BM68_Dseriata_TGH               ................TAGETIGSKLLQKMGWKPGQGVG.....................
K2RXL0_Mphaseolina_TGH                ................TTGETMGAKLLQRMGWKPGQGVG.....................
A0A165JKY3_Xheveae_TGH                ................TAGETMGVKLLKRMGWREGQGVG.....................
A0A0G2GV44_Pchlamydospora_TGH         ................PTGETKGVQLLRRMGWREGQGIG.....................
F0X9I8_Gclavigera_TGH                 ................VQGETMGTRLLKKMGWKDGQGIG.....................
S3BTH5_Opiceae_TGH                    ................ASGETMGTRLLMKMGWREGQGIG.....................
A0A0C2J2D4_Sbrasiliensis_TGH          ...............GARGETMGTKLLKKMGWKEGQGIG.....................
A0A167VN43_Sinsectorum_TGH            ................VQGETMGTKLLKKMGWREGQGIG.....................
F7W020_Smacrospora_TGH                ............................................................
A0A151N705_Amississippiensis_TGH      .............PIIPARITIGVELLRKMGWKEGQGVG....................I
A0A286X9R8_Cporcellus_TGH             .............PIIPAKLSVGFELLRKMGWKEGQGIG....................I
A0A1S3ARH5_Eeuropaeus_TGH             .............PIIPAKLSIGFELLRKMGWKEGQGIG....................I
Q9BRR8_Hsapiens_TGH                   .............PIIPAKLSVGFELLRKMGWKEGQGVG....................I
Q9DBM1_Mmusculus_TGH                  .............PIIPAKLSVGFELLRKMGWKEGQGVG....................I
L5LZ46_Mdavidii_TGH                   .............PIIPATLSVGFELLRKMGWKEGQGIG....................I
S7PC93_Mbrandtii_TGH                  .............PIIPATLSVGFELLRKMGWKEGQGIG....................I
A0A1S3FZ16_Dordii_TGH                 .............PIVPAKLSVGFELLRKMGWKEGQGVG....................I
F6Z6Q3_Mdomestica_TGH                 .............PIIPAKLSIGFELLRKMGWKEGQGVG....................I
G3WTW2_Sharrisii_TGH                  .............PIIPAKLSIGFELLRKMGWREGQGIG....................I
Q21827_Celegans_TGH                   .............TIVPVSNSIGVRMLRSMGWREGRGIG.....................
Q9VUA0_Dmelanogaster_TGH              .............AILPVRDKVAVRILKSMGWKPGQGVG..............EQYLMEH
```

```
                                                                200       210
Aco012726.1_Acomosus_TGH                 ..............................VLNPKQDLHGLGYD........PGFGIGAL
A0A199V6F8_Acomosus_TGH                  ..............................VLNPKQDLHGLGYD........PGFGIGAL
M0TMB2_Macuminata_TGH                    ............NLTENKINFLTYAYLFQEVLQPKQDLYGLGYD........PGFGIGAL
A0A2H3XE04_Pdactylifera_TGH              ..............................VLHPKLDLHGLGYD........PGFGIGAL
A0A0Q3H630_Bdistachyon_TGH               ..............................VLHPKEDLHGLGFD........PGFGIGAL
J3MEA3_Obrachyantha_TGH                  ..............................VLHPKQDLHGLGFD........PGFGIGAL
B8B2G4_Osativa_TGH                       ..............................VLHPKQDLHGLGFD........PGFGIGAL
K3XV17_Sitalica_TGH                      ..............................VLHPKQDLHGLGYD........PGFGIGAL
Q8GXN9_Athaliana_TGH                     ..............................VLNPKQDLHGLGYD........PGFGIGAL
Bol036258_Boleraceacapitata_TGH          ..............................VLNPKQDLHGLGYD........PGFGIGAL
V4MKB5_Esalsugineum_TGH                  ..............................VLNPKQDLHGLGFD........PGFGIGAL
A0A087GCN0_Aalpina_TGH                   ..............................VLNPKQDLHGLGFD........PGFGIGAL
A0A151SPT8_Ccajan_TGH                    ..............................VLNPKEDLYGLGFD........PGFGIGAL
I1K185_Gmax_TGH                          ..............................VLNPKQDLHGLGFD........PGFGIGAL
V7CD35_Pvulgaris_TGH                     ..............................VLNPKQDLHGLGFD........PGFGIGAL
A0A1S3UTP2_Vradiata_TGH                  ..............................VLNPKQDLHGLGFD........PGFGIGAL
A0A1S2YR44_Carietinum_TGH                ..............................VLNPKQDLYGLGFD........PGFGIGAL
G7JT16_Mtruncatula_TGH                   ..............................VLNPKQDLYGLGFD........PGFGIGAL
A0A1U8FWC2_Cannuum_TGH                   ..............................MLNPKQDLHGLGYD........PGFGVGAL
V4TW69_Cclementina_TGH                   ..............................VLNPKQDLHGLGYD........PGFGIGAL
A0A1R3G1K6_Ccapsularis_TGH               ..............................VLNPKEDLHGLGYD........PGFGIGAL
A0A1R3H0J1_Colitorius_TGH                ..............................VLNPKEDLHGLGYD........PGFGIGAL
A0A0D2QRF0_Graimondii_TGH                ..............................VCNPKQDLHGLGYD........PGFGIGAL
A0A067JJA6_Jcurcas_TGH                   ..............................VLNPKQDLYGLGYD........PGFGIGAL
B9RE60_Rcommunis_TGH                     ..............................VLNPKQDLYGLGYD........PGFGIGAL
SapurV1A.0789s0080.1_SSpurpurea_TGH      .......PVRIS..................ILNPKEDTHGLGYD........PGFGIGAL
A0A2I4E4A1_Jregia_TGH                    ............................................................
F6GTG8_Vvinifera_TGH                     ..............................VLNPKQDLHGLGYD........PGFGIGAL
W9S666_Mnotabilis_TGH                    ..............................TLTPKQDLHGLGFD........PGFGIGAL
A0A068TQE9_Ccanephora_TGH                ..............................VLNPKQDSHGLGYD........PGFGIGAL
A0A200Q3N1_Mcordata_TGH                  ..............................VLNPKQDMHGLGYD........PGFGIGAL
A0A1U8AEE6_Nnucifera_TGH                 ..............................VLHPKQDLHGLGYD........PGFGIGAL
A0A0A0LSK9_Csativus_TGH                  ..............................VINPKQDLHGLGFD........SGFGIGAL
A0A059A5E4_Egrandis_TGH                  ..............................VLNPKQDLHGLGYD........PGFGIGAL
A0A218XZU2_Pgranatum_TGH                 ..............................VLDPKQDLHGLGYD........PGFGIGAL
A0A022RKT3_Eguttata_TGH                  ..............................VFNPKQDLFGLGYD........PGFGIGAL
A0A0J8B830_Bvulgaris_TGH                 ..............................VRQPKQDLYGLGYD........PGFGIGAL
Kalax.0309s0034.1_Klaxiflora_TGH         ..............................VLNPKQDLHGLGFD........PGFGIGAL
A0A124SAU9_Ccardunculus_TGH              ..............................VLNPKQDMYGLGYD...P....PGFGIGAL
W1PSA4_Atrichopoda_TGH                   ..............................VRHPKRDIHGLGYD........PGIGIGAL
A0A0K9NIC3_Zmarina_TGH                   ..............................VLNPKQDLYGLGYD........PGFGIGAL
A0A2C9VXU1_Mesculenta_TGH                ..............................YLKPDLDTTGQG..................
A0A0U9HQK1_Knitens_TGH                   ..............................VLQPKKDFYGLGFD........AGFGVGAL
Sphfalx0151s0047.1_SSfallax_TGH          ....SK........................VMNPKKDLHGLGFD........SGFGIGAL
D8QX10_Smoellendorffii_TGH               ..............................VRKIKDNVHGLGFD.........GFGIGAL
E1ZI32_Cvariabilis_TGH                   ..............................LLAPKTDVHGLGFD........IAFGSGVL
C1FFR5_Mcommoda_TGH                      ..............................VLHPKTNLHGVGYD........EAFGVGVF
A0A086T442_Achrysogenum_TGH              ..............................QFNRKVDRKGLGHE........SGIGVGVL
K3V9N2_Fpseudograminearum_TGH            ..............................RFIRKSDRKGLGHD........GGIGVGIL
W7LTD5_Gmoniliformis_TGH                 ..............................QFVRKNDRKGLGHD........GGIGVGIL
G9NMW3_Hatroviridis_TGH                  ..............................RFVHKTDRKGLGHS........GGIGIGIL
A0A0G0A2Q9_Tharzianum_TGH                ..............................RFVRKTDRMGLGHN........GGIGVGIL
A0A1T3CLH2_Tguizhouense_TGH              ..............................RFVRKTDRMGLGHN........GGIGVGIL
A0A084QRJ1_Schlorohalonata_TGH           ..............................QFVRKTDRKGLGHH........GGIGVGIL
J4KPK4_Bbassiana_TGH                     ..............................RFVRKTDRMGLGYQ........GGMGVGIL
A0A2H4SR46_Cmilitaris_TGH                ..............................QFVRKIDRMGLGYQ........GGMGVGIL
A0A167VSL6_Cfumosorosea_TGH              ..............................RFVRKIDRMGLGYQ........GGMGVGIL
A0A179G8E9_Pchlamydosporia_TGH           ..............................HFVRKTDRMGLGHR........GAFGVGVL
A0A0L0MXB1_Tophioglossoides_TGH          ..............................RLIRKTDRKGLGHQ........GAFGVGVL
Q2GSN0_Cglobosum_TGH                     ..............................HFVRKTDHKGLGYA........GGIGVGIL
G2QES8_Mthermophila_TGH                  ..............................SFARKTDHRGLGYE........GGIGVGIL
A0A175VUX4_Mmycetomatis_TGH              ..............................SFVRKADHKGLGYG........GGIGVGIL
G2QRS6_Tterrestris_TGH                   ..............................SFVRKTDHKGLGYA........GGIGVGIL
B2ARI9_Panserina_TGH                     ..............................SFVRKTDHKGLGYG........GGIGIGIL
F7W019_Smacrospora_TGH                   ..............................AFVKKTDKKGLGYA........GGIGIGIL
A0A1J7J5C9_Cligniaria_TGH                ..............................SFTRKTGRKGLGFE........GGIGIGIL
A0A0G2IFE8_Dampelina_TGH                 ..............................QLIHKTDRKGLGYD........GGIGMGVL
A0A194W011_Vmali_TGH                     ..............................GFVRKTDRKGLGYQ........GGIGIGVL
R8BKG4_Tminima_TGH                       ..............................GFTRKMDRKGLGYE........GGIGIGVL
G0SA63_Cthermophilum_TGH                 ..............................SFVRKTDHKGLGYA........GSIGVGIL
J3NFM2_Ggraminis_TGH                     ..............................RFVRKTDHKGLGFA........GGIGMGVL
A0A0C4DZ99_Mpoae_TGH                     ..............................KFERKTDYKGLGFA........GGIGMGVL
L7IH22_Moryzae_TGH                       ..............................QFVKKLDHKGLGYA........GGIGIGII
A0A1Y2WZC5_Daldiniasp._TGH               ..............................SFTKKLDRKGLGYE........GGIGIGVL
A0A1Y2V8J1_Hypoxylon                     ..............................SFVKKLDRKGLGFE........GGIGIGVL
A0A1Y2W1E2_Hypoxylon                     ..............................SFTKKLDRKGLGYE........GGIGIGIL
A0A1W2TFH3_Rnecatrix_TGH                 ..............................AFIKKRDRKGLGYE........GGIGIGVL
W3WZ77_Pfici_TGH                         ..............................AIVKKLDRKGLGYA........GGIGIGIL
A0A1Y2DPV9_Pvexata_TGH                   ..............................SFVKKLDRKGLGYF........GGIGIGIL
A0A136JHX1_Mbolleyi_TGH                  ..............................AFVKKLDHQGLGYE........GGIGIGIL
S3CR73_Glozoyensis_TGH                   ..............................SFTKKNDHKGLGYE........GGIGIGIL
K1WET1_Mbrunnea_TGH                      ..............................RFVRKNDHKGLGFD........GGIGIGIL
A0A218YYN9_Mcoronariae_TGH               ..............................RFTRKNDHRGLGFD........GGIGIGIL
A0A194XKL9_Pscopiformis_TGH              ..............................SFVKKNDHKGLGFD........GGIGIGIL
A0A0C3DJQ0_Omaius_TGH                    ..............................SFAKKHDHRGLGCG........GGIGIGIL
A7ECA3_Ssclerotiorum_TGH                 ..............................RFLRKDDHKGLGFD........GGIGIGIL
W9CY85_Sborealis_TGH                     ..............................SFLRKDDHKGLGFD........GGIGIGIL
A0A094E7L1_Pseudogymnoascus              ..............................TFIRKTDHKGLGFA........GGIGMGVL
A0A1B8CGM9_Pseudogymnoascus              ..............................TFIRKIDHKGLGFA........GGIGMGVL
A0A094H174_Pseudogymnoascus              ..............................TFVRKIDHKGLGFA........GGIGMGVL
Q0D178_Aterreus_TGH                      ..............................AFIHKTDHKGLGFE........GAFGVGVL
A0A1L9NNK2_Atubingensis_TGH              ..............................AFIHKTDHKGLGFE........GAFGVGVL
A1CQ12_Aclavatus_TGH                     ..............................AFVHKTDHKGLGFE........GAFGVGVL
A0A1L9RVU1_Awentii_TGH                   ..............................AFIRKTDHKGLGFE........GGFGVGIL
A0A1E3BNS4_Acristatus_TGH                ..............................AFVQKNDHKGLGYE........GAFGVGVL
I7ZRW9_Aoryzae_TGH                       ..............................SFTHKTDHKGLGFE........GAFGVGVL
A0A0F8UC36_Aochraceoroseus_TGH           ..............................AFIHKTDYKGLGFE........GAFGVGVL
Q5BE58_Enidulans_TGH                     ..............................AFIRKNDYKGLGFE........GAFGVGVL
A0A0A2KYD8_Pitalicum_TGH                 ..............................AFIRKTDRKGLGFE........GGLGVGPL
A0A1V6QJR5_Pantarcticum_TGH              ..............................AFVRKTDRTGLGFE........GGLGVGVL
A0A1Q5UD44_Psubrubescens_TGH             ..............................SFIRKTDFKGLGFE........GGFGVGIL
A0A1V6P0U0_Pdecumbens_TGH                ..............................AFIRKTDHKGLGFE........GGIGIGIL
A0A0F4YXS3_Remersonii_TGH                ..............................AFVRKLDHKGLGFE........GGFGVGVL
```

```
B8LVN1_Tstipitatus_TGH                .............................TFVRKVDHFGLGFQ........GGFGVGIL
A0A225B272_Tatroroseus_TGH            .............................AFTRKTDHFGLGFA........GGMGVGIL
A0A0H1BJD0_Bsilverae_TGH              .............................SFAKKNDHKGLGFE........GGFGVGIL
A0A1B7NTS8_Esp._TGH                   .............................SFVKKNDYKGLGFE........GGFGVGIL
C0RYG0_Pbrasiliensis_TGH              .............................TFMKKNDYKGLGFE........GGFGVGIL
C4JTX0_Ureesii_TGH                    .............................SFNRKDDSKGLGFR........GGFGVGIL
R7Z059_Capollinis_TGH                 .............................GFARKNDRKGLGYS........GGFGVGIL
A0A1S8BM68_Dseriata_TGH               .............................TFVRKNDHKGLGYE........SGFGVGVL
K2RXL0_Mphaseolina_TGH                .............................SFVRKNDHKGLGYE........GGFGVGVL
A0A165JKY3_Xheveae_TGH                .............................SFDRKDDYQGLGYV........GGFGVGVL
A0A0G2GV44_Pchlamydospora_TGH         .............................AIVHKTDSKGLGYG........GGFGVGIL
F0X9I8_Gclavigera_TGH                 .............................RLLKKTDRKGLGYE..G.....GGIGMGVL
S3BTH5_Opiceae_TGH                    .............................SLVKKSDRKGLGFE........GGIGVGIL
A0A0C2J2D4_Sbrasiliensis_TGH          .............................RLVKKTDHKGLGFQ........GGIGLGVL
A0A167VN43_Sinsectorum_TGH            .............................RLVKKTDRKGLGFA....EGDGGGIGIGVL
F7W020_Smacrospora_TGH                ...........................................................
A0A151N705_Amississippiensis_TGH      YGC...Y......................QLTLKENVHGLGYKNL.....QAFGVGAL
A0A286X9R8_Cporcellus_TGH             YGC...Y......................DFTPKDNVHGLEYKNL.....QAFGVGAL
A0A1S3ARH5_Eeuropaeus_TGH             YGC...Y......................DFTPKDNVHGLAYKNL.....QAFGVGAL
Q9BRR8_Hsapiens_TGH                   YGC...Y......................DFTPKDNVHGLAYKNL.....QAFGVGAL
Q9DBM1_Mmusculus_TGH                  YGC...Y......................DFTPKDNVHGLAYKNL.....QAFGVGAL
L5LZ46_Mdavidii_TGH                   YGC...Y......................DFTPKDNVHGLAYKNL.....QAFGVGAL
S7PC93_Mbrandtii_TGH                  YGC...Y......................DFTPKDNVHGLAYKNL.....QAFGVGAL
A0A1S3FZ16_Dordii_TGH                 YGC...Y......................DFTPKDNVHGLAYKNL.....QAFGVGAL
F6Z6Q3_Mdomestica_TGH                 YGC...Y......................DFTPKDNVHGLAYRNF.....QAFGVGAL
G3WTW2_Sharrisii_TGH                  YGC...Y......................DFTPKDNVHGLAYKNL.....QAFGVGAL
Q21827_Celegans_TGH                   ......E......................QLTPLTGTHGIGYQ........QAFGVGAF
Q9VUA0_Dmelanogaster_TGH              YGAE.........................FYTPKENRFGMSYS........QAFGVGAF
```

220

Aco012726.1_Acomosus_TGH	ELD............................IEDE.DIYA....................
A0A199V6F8_Acomosus_TGH	ELD............................IEDE.DIYA....................
M0TMB2_Macuminata_TGH	ELD............................FEDE.DIYA....................
A0A2H3XE04_Pdactylifera_TGH	ELD............................IEDE.DIYA....................
A0A0Q3H630_Bdistachyon_TGH	ELD............................VEDE.DIYA....................
J3MEA3_Obrachyantha_TGH	ELG............................VEDE.DIYA....................
B8B2G4_Osativa_TGH	ELG............................VEDE.DIYA....................
K3XV17_Sitalica_TGH	ELD............................VEDE.DIYA....................
Q8GXN9_Athaliana_TGH	ELD............................VEDE.DVYA....................
Bo1036258_Boleraceacapitata_TGH	ELD............................VEDE.DVYA....................
V4MKB5_Esalsugineum_TGH	ELD............................VEDE.DVYA....................
A0A087GCN0_Aalpina_TGH	ELD............................VEDE.DVYA....................
A0A151SPT8_Ccajan_TGH	ELD............................AEDE.DVYA....................
I1K185_Gmax_TGH	ELD............................AEDE.DVYA....................
V7CD35_Pvulgaris_TGH	ELD............................AEDE.DVYA....................
A0A1S3UTP2_Vradiata_TGH	ELD............................AEDE.DVYA....................
A0A1S2YR44_Carietinum_TGH	ELD............................AEDE.DVYA....................
G7JT16_Mtruncatula_TGH	ELD............................AEDE.DVYA....................
A0A1U8FWC2_Cannuum_TGH	ELD............................VEDE.DVYA....................
V4TW69_Cclementina_TGH	EYD............................AEDE.DLYG....................
A0A1R3G1K6_Ccapsularis_TGH	EFD............................AEDE.DVYTFTLVNMHLRVIKVYWSLWKA
A0A1R3H0J1_Colitorius_TGH	EFD............................AEDE.DVYT....................
A0A0D2QRF0_Graimondii_TGH	EYD............................AEDE.DIYG....................
A0A067JJA6_Jcurcas_TGH	EYD............................AEDE.DVYA....................
B9RE60_Rcommunis_TGH	EYD............................AEDE.DLYG....................
SapurV1A.0789s0080.1_SSpurpurea_TGH	EYD............................AEDE.DVYA....................
A0A2I4E4A1_Jregia_TGH	....
F6GTG8_Vvinifera_TGH	ELD............................VEDE.DVYA....................
W9S666_Mnotabilis_TGH	ELD............................AEDE.DVFA....................
A0A068TQE9_Ccanephora_TGH	EYD............................AEDE.DVYA....................
A0A200Q3N1_Mcordata_TGH	ELD............................TEDE.DIYA....................
A0A1U8AEE6_Nnucifera_TGH	ELD............................VEDE.DIYA....................
A0A0A0LSK9_Csativus_TGH	ELD............................VEDE.DVYT....................
A0A059A5E4_Egrandis_TGH	ELD............................AEDE.DVYA....................
A0A218XZU2_Pgranatum_TGH	ELD............................AEDE.DVYG....................
A0A022RKT3_Eguttata_TGH	ELD............................TEDA.DVYD....................
A0A0J8B830_Bvulgaris_TGH	ELD............................VEDE.DVYS....................
Kalax.0309s0034.1_Klaxiflora_TGH	ELD............................AEDD.DVYA....................
A0A124SAU9_Ccardunculus_TGH	ELD............................AEDE.DVYA....................
W1PSA4_Atrichopoda_TGH	ELD............................VEDE.DIYA....................
A0A0K9NIC3_Zmarina_TGH	ELD............................IEDE.DIYE....................
A0A2C9VXU1_Mesculenta_TGH	....
A0A0U9HQK1_Knitens_TGH	EVG............................EEDE.DVYA....................
Sphfalx0151s0047.1_SSfallax_TGH	ELG............................EEDE.DVYS....................
D8QX10_Smoellendorffii_TGH	EMG............................DEDE.DVYA....................
E1ZI32_Cvariabilis_TGH	DEDGYGHGASLDDYVTHDDVGEYDDEVEAGGMPRS.RVPK....................
C1FFR5_Mcommoda_TGH	EDD.............................PDE.DVYR....................
A0A086T442_Achrysogenum_TGH	NNG............................SDEE.DPYE....................
K3V9N2_Fpseudograminearum_TGH	NNG............................SDEE.DPYE....................
W7LTD5_Gmoniliformis_TGH	NNG............................SDEE.DPYE....................
G9NMW3_Hatroviridis_TGH	NTG............................SDDE.DPYE....................
A0A0G0A2Q9_Tharzianum_TGH	NDG............................SDDE.DPYE....................
A0A1T3CLH2_Tguizhouense_TGH	NDG............................SDDE.DPYE....................
A0A084QRJ1_Schlorohalonata_TGH	NDG............................SDEE.DPYE....................
J4KPK4_Bbassiana_TGH	NNG............................SDDE.DPYE....................
A0A2H4SR46_Cmilitaris_TGH	NNG............................SDDE.DPYE....................
A0A167VSL6_Cfumosorosea_TGH	NNG............................SDDE.NPYE....................
A0A179G8E9_Pchlamydosporia_TGH	NTG............................SDEE.DPYD....................
A0A0L0MXB1_Tophioglossoides_TGH	NTG............................SDDE.DPYD....................
Q2GSN0_Cglobosum_TGH	NTG............................SDDE.DPYE....................
G2QES8_Mthermophila_TGH	NTG............................SDDE.DPYE....................
A0A175VUX4_Mmycetomatis_TGH	NTG............................SDDE.DPYE....................
G2QRS6_Tterrestris_TGH	NTG............................SDDE.DPYE....................
B2ARI9_Panserina_TGH	NTG............................SDDE.DPYE....................
F7W019_Smacrospora_TGH	NTG............................SDDE.DPYE....................
A0A1J7J5C9_Cligniaria_TGH	NTG............................SDDE.DPYE....................
A0A0G2IFE8_Dampelina_TGH	NTG............................SDDE.DPYE....................
A0A194W011_Vmali_TGH	NTG............................SDDE.DPYE....................
R8BKG4_Tminima_TGH	NTG............................SDDE.DPYE....................
G0SA63_Cthermophilum_TGH	NTG............................SDDE.DPYE....................
J3NFM2_Ggraminis_TGH	NTG............................SDDE.DPYE....................
A0A0C4DZ99_Mpoae_TGH	NTG............................SDDE.DPYE....................
L7IH22_Moryzae_TGH	NTG............................SDDE.DPYE....................
A0A1Y2WZC5_Daldiniasp._TGH	NTG............................SDDE.DPYE....................
A0A1Y2V8J1_Hypoxylon	NTG............................SDDE.DPYE....................
A0A1Y2W1E2_Hypoxylon	NTG............................SDDE.DPYE....................
A0A1W2TFH3_Rnecatrix_TGH	NTG............................SDDE.DPFE....................
W3WZ77_Pfici_TGH	NNG............................SDDE.DPYE....................
A0A1Y2DPV9_Pvexata_TGH	NNG............................SDDE.DPYE....................
A0A136JHX1_Mbolleyi_TGH	NTG............................SDDE.DPYE....................
S3CR73_Glozoyensis_TGH	NTG............................SDDE.DPYE....................
K1WET1_Mbrunnea_TGH	NTG............................SDDE.DPYE....................
A0A218YYN9_Mcoronariae_TGH	NTG............................SDDE.DPYE....................
A0A194XKL9_Pscopiformis_TGH	NTG............................SDDE.DPYE....................
A0A0C3DJQ0_Omaius_TGH	NTG............................SDDE.DPYE....................
A7ECA3_Ssclerotiorum_TGH	NTG............................SDDE.DPYE....................
W9CY85_Sborealis_TGH	NNG............................SDDE.DPYE....................
A0A094E7L1_Pseudogymnoascus	NTG............................SDDE.DPYE....................
A0A1B8CGM9_Pseudogymnoascus	NTG............................SDDE.DPYE....................
A0A094H174_Pseudogymnoascus	NTG............................SDDE.DPYE....................
Q0D178_Aterreus_TGH	NTG............................SDDE.DPYS....................
A0A1L9NNK2_Atubingensis_TGH	NTG............................SDDE.DPYS....................
A1CQ12_Aclavatus_TGH	NTG............................SDDE.DPYA....................
A0A1L9RVU1_Awentii_TGH	NTG............................SDDE.DPYS....................
A0A1E3BNS4_Acristatus_TGH	NTG............................SDDE.DPYS....................
I7ZRW9_Aoryzae_TGH	NTG............................SDDE.DPYS....................
A0A0F8UC36_Aochraceoroseus_TGH	NTG............................SDDE.DPYS....................
Q5BE58_Enidulans_TGH	NTG............................SDDE.DPYS....................
A0A0A2KYD8_Pitalicum_TGH	NTG............................SDDE.DPYS....................
A0A1V6QJR5_Pantarcticum_TGH	NTG............................SDDE.DPYS....................
A0A1Q5UD44_Psubrubescens_TGH	NTG............................SDDE.DPYS....................
A0A1V6P0U0_Pdecumbens_TGH	NTG............................SDDE.DPYS....................
A0A0F4YXS3_Remersonii_TGH	NTG............................SDEE.DPYE....................

```
B8LVN1_Tstipitatus_TGH               NTG............................SDDE.DPYS....................
A0A225B272_Tatroroseus_TGH           NTG............................SDDD.DPYS....................
A0A0H1BJD0_Bsilverae_TGH             NTG............................SDDE.DPYE....................
A0A1B7NTS8_Esp._TGH                  NTG............................SDDE.DPYE....................
C0RYG0_Pbrasiliensis_TGH             NTG............................SDDE.DPYE....................
C4JTX0_Ureesii_TGH                   NTG............................SDDE.DPYE....................
R7Z059_Capollinis_TGH                NTG............................SDEE.DAYE....................
A0A1S8BM68_Dseriata_TGH              NDG............................SGDE.DPYE....................
K2RXL0_Mphaseolina_TGH               NDG............................SDEE.DPYA....................
A0A165JKY3_Xheveae_TGH               NTG............................SDDE.DPFE....................
A0A0G2GV44_Pchlamydospora_TGH        NTG............................SDDE.DPYE....................
F0X9I8_Gclavigera_TGH                NTG............................SDDE.DPYD....................
S3BTH5_Opiceae_TGH                   NTG............................SDDE.DPYD....................
A0A0C2J2D4_Sbrasiliensis_TGH         NTG............................SDDE.DPYD....................
A0A167VN43_Sinsectorum_TGH           NTG............................SDDEDDPYD....................
F7W020_Smacrospora_TGH               ...............................SDDN........................
A0A151N705_Amississippiensis_TGH     E...............................DDD.DIYA....................
A0A286X9R8_Cporcellus_TGH            E...............................EDD.DIYA....................
A0A1S3ARH5_Eeuropaeus_TGH            E...............................EDD.DIYA....................
Q9BRR8_Hsapiens_TGH                  E...............................EDD.DIYA....................
Q9DBM1_Mmusculus_TGH                 E...............................EDD.DIYA....................
L5LZ46_Mdavidii_TGH                  E...............................EDD.DIYA....................
S7PC93_Mbrandtii_TGH                 E...............................EDD.DIYA....................
A0A1S3FZ16_Dordii_TGH                E...............................EDD.DIYA....................
F6Z6Q3_Mdomestica_TGH                E...............................EDD.DIYA....................
G3WTW2_Sharrisii_TGH                 E...............................EDD.DIYA....................
Q21827_Celegans_TGH                  E...............................EDE.SVYS....................
Q9VUA0_Dmelanogaster_TGH             E...............................EDE.DIYA....................
```

```
                                                                                                   230
                                                                                                     .
Aco012726.1_Acomosus_TGH                ..............................................ER.FRPPVI.PADF
A0A199V6F8_Acomosus_TGH                 ..............................................ER.FRPPVI.PADF
M0TMB2_Macuminata_TGH                   ..............................................ER.FHPPVI.PVNF
A0A2H3XE04_Pdactylifera_TGH             ..............................................VR.FLPPVI.PADF
A0A0Q3H630_Bdistachyon_TGH              ..............................................ER.FHPPEI.PADF
J3MEA3_Obrachyantha_TGH                 ..............................................ER.FDPPDI.PANF
B8B2G4_Osativa_TGH                      ..............................................ER.FDPPEI.PSDF
K3XV17_Sitalica_TGH                     ..............................................ER.FLPPEI.PDDF
Q8GXN9_Athaliana_TGH                    ..............................................ER.FNPPII.PKDF
Bol036258_Boleraceacapitata_TGH         ..............................................ER.FDPPKI.PKDF
V4MKB5_Esalsugineum_TGH                 ..............................................DR.FDPPKI.PKDF
A0A087GCN0_Aalpina_TGH                  ..............................................ER.FDPPKI.PKDF
A0A151SPT8_Ccajan_TGH                   ..............................................ER.FDSPLI.PKDF
I1K185_Gmax_TGH                         ...........................KEVEEPST...........ER.FEAPLI.PKDF
V7CD35_Pvulgaris_TGH                    ..............................................ER.FESPLI.PKDF
A0A1S3UTP2_Vradiata_TGH                 ..............................................ER.FEAPLI.PKDF
A0A1S2YR44_Carietinum_TGH               ..............................................ER.FEAPRI.PKDF
G7JT16_Mtruncatula_TGH                  ..............................................ER.FEAPLV.PKDF
A0A1U8FWC2_Cannuum_TGH                  ..............................................ER.FDPPVI.PQNF
V4TW69_Cclementina_TGH                  ..............................................ER.FDPPVV.PKDF
A0A1R3G1K6_Ccapsularis_TGH              KLIASVTFYGGVKKSYFVFPKY........................ER.FDPPII.PKDF
A0A1R3H0J1_Colitorius_TGH               ..............................................ER.FDPPII.PKDF
A0A0D2QRF0_Graimondii_TGH               ..............................................ER.FDPPII.PKDF
A0A067JJA6_Jcurcas_TGH                  ..............................................ER.FQPPAI.PEDF
B9RE60_Rcommunis_TGH                    ..............................................ER.FDPPVI.PKDF
SapurV1A.0789s0080.1_SSpurpurea_TGH     ..............................................ER.FDPPVI.PKDF
A0A2I4E4A1_Jregia_TGH                   ..............................................ER.FDAPVI.PKDF
F6GTG8_Vvinifera_TGH                    ..............................................ER.FDPPVV.PKNF
W9S666_Mnotabilis_TGH                   ..............................................ER.FDPPLV.PKDF
A0A068TQE9_Ccanephora_TGH               ..............................................ER.FGPPVI.PKDF
A0A200Q3N1_Mcordata_TGH                 ..............................................ER.CGPPVI.PDDF
A0A1U8AEE6_Nnucifera_TGH                ..............................................ER.FDPPVI.PNDF
A0A0A0LSK9_Csativus_TGH                 ..............................................ER.FDPPVI.PKDF
A0A059A5E4_Egrandis_TGH                 ..............................................QR.FDPPVI.PKDF
A0A218XZU2_Pgranatum_TGH                ..............................................ER.FDPPVI.PKDF
A0A022RKT3_Eguttata_TGH                 ..............................................ER.FDPPVI.PKDF
A0A0J8B830_Bvulgaris_TGH                ..............................................ER.FDPPIV.PKDF
Kalax.0309s0034.1_Klaxiflora_TGH        ..............................................ER.YNPPII.PKDF
A0A124SAU9_Ccardunculus_TGH             ..............................................QR.FDPPVV.PKDF
W1PSA4_Atrichopoda_TGH                  ..............................................ER.FLPPVV.PSDF
A0A0K9NIC3_Zmarina_TGH                  ..............................................ER.FPPPNI.PLGF
A0A2C9VXU1_Mesculenta_TGH               ..............................................ER.FDPPVI.PKDF
A0A0U9HQK1_Knitens_TGH                  ..........................F...................QEWFAPPVV.PKGF
Sphfalx0151s0047.1_SSfallax_TGH         ..............................................KQWFPPPVV.PLDF
D8QX10_Smoellendorffii_TGH              ..............................................EW.FHPPVV.PPDY
E1ZI32_Cvariabilis_TGH                  ..........................................QG..AY.FPPPRV.PPGY
C1FFR5_Mcommoda_TGH                     ..........................................EG..TW.YPPPVV.PKSF
A0A086T442_Achrysogenum_TGH             ......................................LRRCHD.......PPAV.PPGW
K3V9N2_Fpseudograminearum_TGH           ......................................LGRCHD.......PPPV.PEGW
W7LTD5_Gmoniliformis_TGH                ......................................LGRCHD.......PPPV.PEGW
G9NMW3_Hatroviridis_TGH                 ......................................LRRCQD.......PPPV.PPDW
A0A0G0A2Q9_Tharzianum_TGH               ......................................LRRCQD.......PPPV.PPGW
A0A1T3CLH2_Tguizhouense_TGH             ......................................LRRCQD.......PPPV.PPGW
A0A084QRJ1_Schlorohalonata_TGH          ......................................LRRCHD.......PPPV.PQGW
J4KPK4_Bbassiana_TGH                    ......................................LHRCHD.......PLGV.PEGW
A0A2H4SR46_Cmilitaris_TGH               ......................................LRRCHD.......PPEV.PEGW
A0A167VSL6_Cfumosorosea_TGH             ......................................LHRCHD.......PLAV.PEGW
A0A179G8E9_Pchlamydosporia_TGH          ......................................LRRCHD.......PPPI.PEGW
A0A0L0MXB1_Tophioglossoides_TGH         ......................................FRRCHD.......PPAI.SSGW
Q2GSN0_Cglobosum_TGH                    ...................................A..ARKCHD.......PPNI.PPDW
G2QES8_Mthermophila_TGH                 ...................................G..VRKCHD.......PPKI.PDGW
A0A175VUX4_Mmycetomatis_TGH             ...................................A..VRKCHD.......PPRV.PPGW
G2QRS6_Tterrestris_TGH                  ...................................A..VRKCHD.......PPQI.PPGW
B2ARI9_Panserina_TGH                    ...................................A..VRKCHD.......PPQV.PPGW
F7W019_Smacrospora_TGH                  ...................................S..VRKCHD.......PPQI.PPGW
A0A1J7J5C9_Cligniaria_TGH               ......................................VRRCHD.......PPEI.PPGW
A0A0G2IFE8_Dampelina_TGH                ......................................VRKCHD.......PPEI.PIGW
A0A194W011_Vmali_TGH                    ......................................IRKCHD.......PPEI.PPDW
R8BKG4_Tminima_TGH                      ......................................VRKCHD.......PPTI.PPDW
G0SA63_Cthermophilum_TGH                ...................................A...............PPRI.PPGW
J3NFM2_Ggraminis_TGH                    .......................................KRCHD.......PPQV.PPDW
A0A0C4DZ99_Mpoae_TGH                    .......................................KKCHD.......PPQV.PPDW
L7IH22_Moryzae_TGH                      ......................................AKKCHD.......PPKV.PPEW
A0A1Y2WZC5_Daldiniasp._TGH              ......................................IRKCHD.......PPKI.PPDW
A0A1Y2V8J1_Hypoxylon                    ......................................IRKCHD.......PPTI.PPDW
A0A1Y2W1E2_Hypoxylon                    ......................................LRKCHD.......PPTI.PADW
A0A1W2TFH3_Rnecatrix_TGH                ......................................LRKCHD.......PPQI.PPGW
W3WZ77_Pfici_TGH                        ......................................LKRCHD.......PPQI.PPGW
A0A1Y2DPV9_Pvexata_TGH                  ......................................LRKCHD.......PPQI.PPDW
A0A136JHX1_Mbolleyi_TGH                 ......................................LRKCHD.......PPQI.PPGW
S3CR73_Glozoyensis_TGH                  ......................................FRKCRD.......PLEI.PEGW
K1WET1_Mbrunnea_TGH                     ......................................RRKCHD........PKV.PEGW
A0A218YYN9_Mcoronariae_TGH              ......................................IRRCHD.......PPQI.PEGW
A0A194XKL9_Pscopiformis_TGH             ......................................FRKCHD.......PPKI.PEGW
A0A0C3DJQ0_Omaius_TGH                   ......................................LRRCHD.......PPKI.PENW
A7ECA3_Ssclerotiorum_TGH                ......................................LRKCHD.......PPTI.PEGW
W9CY85_Sborealis_TGH                    ......................................LRKCHD.......PPTI.PQGW
A0A094E7L1_Pseudogymnoascus             ......................................FRKCHD.......PVEI.PPDW
A0A1B8CGM9_Pseudogymnoascus             ......................................FRKCHD.......PVEV.PPDW
A0A094H174_Pseudogymnoascus             ......................................FRKCHD.......PVEV.PPDW
Q0D178_Aterreus_TGH                     ......................................FRKCHDQ......PPEI.PKDW
A0A1L9NNK2_Atubingensis_TGH             ......................................FRKCSDQ......PPEI.PKDW
A1CQ12_Aclavatus_TGH                    ......................................FRKCHDQ......PPEI.PKDW
A0A1L9RVU1_Awentii_TGH                  ......................................FRKCHDQ......PPEI.PKDW
A0A1E3BNS4_Acristatus_TGH               ......................................FRKCHDQ.......PEV.PSDW
I7ZRW9_Aoryzae_TGH                      ......................................FRKCHDQ......PPEV.PKDW
A0A0F8UC36_Aochraceoroseus_TGH          ......................................FRKCHD.......PPEI.PKDW
Q5BE58_Enidulans_TGH                    ......................................FRKCHD.......PPEI.PKDW
A0A0A2KYD8_Pitalicum_TGH                ......................................FRKCRD.......PPVI.PEDW
A0A1V6QJR5_Pantarcticum_TGH             ......................................FRKCRD.......PPVI.PEGW
A0A1Q5UD44_Psubrubescens_TGH            ......................................FRKCRD.......PPRI.PEGW
A0A1V6P0U0_Pdecumbens_TGH               ......................................FRKCRD.......PPKI.PEDW
A0A0F4YXS3_Remersonii_TGH               ......................................FRKCHD.......PPEI.PKDW
```

```
B8LVN1_Tstipitatus_TGH                ......................................FRKCHD.......PPDI.PPGW
A0A225B272_Tatroroseus_TGH            ......................................FRKCHD.......PPDI.PSGW
A0A0H1BJD0_Bsilverae_TGH              .....................................EFRKCHDQ......APEI.PKDW
A0A1B7NTS8_Esp._TGH                   .....................................EFRKCHDQ......APDI.PKDW
C0RYG0_Pbrasiliensis_TGH              .....................................EFRKCHDR......PPEI.PKNW
C4JTX0_Ureesii_TGH                    ......................................LRKCHDT......PPDV.PTDW
R7Z059_Capollinis_TGH                 ......................................LRKCHD.......PPKI.PEGW
A0A1S8BM68_Dseriata_TGH               ......................................FRKCHD.......PPTI.PEGW
K2RXL0_Mphaseolina_TGH                ......................................FRRCHD.......PPTI.PEGW
A0A165JKY3_Xheveae_TGH                ......................................FRKCHD.......PPKI.PPDW
A0A0G2GV44_Pchlamydospora_TGH         ......................................FRKCHD.......PPEV.PKEW
F0X9I8_Gclavigera_TGH                 ......................................ARLCHD.......PPTI.PAGW
S3BTH5_Opiceae_TGH                    ......................................ARLCHD.......PPTI.PPGW
A0A0C2J2D4_Sbrasiliensis_TGH          ......................................SRVCHD.......PPVI.PPGW
A0A167VN43_Sinsectorum_TGH            ......................................VRLCYN.......PPTI.PPGW
F7W020_Smacrospora_TGH                ....................................C...EVND.......PPQY.PASE
A0A151N705_Amississippiensis_TGH      ......................DEEP.........................PPDL.PRGY
A0A286X9R8_Cporcellus_TGH             ......................DEEP.........................PPEL.PRDY
A0A1S3ARH5_Eeuropaeus_TGH             ......................DEEP.........................PPQL.PKDY
Q9BRR8_Hsapiens_TGH                   ......................DEEP.........................PPEL.PRDY
Q9DBM1_Mmusculus_TGH                  ......................DEEP.........................PPQL.PRDY
L5LZ46_Mdavidii_TGH                   ......................DEEP.........................PPEL.PRDY
S7PC93_Mbrandtii_TGH                  ......................DEEP.........................PPEL.PRDY
A0A1S3FZ16_Dordii_TGH                 ......................DEEP...................A.....VP.......
F6Z6Q3_Mdomestica_TGH                 ......................DEEP.........................PPEL.PKDY
G3WTW2_Sharrisii_TGH                  ......................DEEP.........................PPEL.PKDY
Q21827_Celegans_TGH                   ...................................................PPRV.PPNF
Q9VUA0_Dmelanogaster_TGH              .................................................DLPRDFQPRNW
```

```
                                       240       250       260       270
Aco012726.1_Acomosus_TGH              ESRHPT....GKSIETNQLFLFLSGGEGHNYYERKLWEQK....................
A0A199V6F8_Acomosus_TGH               ESRHPT....GKSIETNQLFLFLSGGEGHNYYERKLWEQK....................
M0TMB2_Macuminata_TGH                 DPHHSA....GKIFESNPLFYFLSGGDGHNYYERKLWEEK....................
A0A2H3XE04_Pdactylifera_TGH           EPYHPT....GKHFESNPLFSFLNGGNGHSYYARKLWEEK....................
A0A0Q3H630_Bdistachyon_TGH            DGHHSS....GKRIEASPQFLFLDGGDGCGYYTRKLWEHQ....................
J3MEA3_Obrachyantha_TGH               DGRHLT....GKHIETNTQFNFLNEGDGCSYYSRKLWEYQ....................
B8B2G4_Osativa_TGH                    DGRHLT....GKHIETNTQFNFLNEGDGCSYYARKLWEYQ....................
K3XV17_Sitalica_TGH                   DARHPT....GKHIETNPQFMFLSGGDGCKYYMRKLWEHQ....................
Q8GXN9_Athaliana_TGH                  VARHSG....GKLYESNQLFDFLREGNGHDYYARRLWEEQ....................
Bol036258_Boleraceacapitata_TGH       VARHLG....GKLYESNQLFDFLRGGSGHDYYVRRLWEEQ....................
V4MKB5_Esalsugineum_TGH               VARHPG....GKLYESNQLFDFLRGGNGHDYYVRRLWEEQ....................
A0A087GCN0_Aalpina_TGH                VARHSG....GKLYESNQMFDFLRGGNGHDYYVRRLWEEQ....................
A0A151SPT8_Ccajan_TGH                 VPHHSG....GKLYETNPLFSFLSGGTGHDYYARKLWESQ....................
I1K185_Gmax_TGH                       VPHHSG....GKLYESNPLFNFLSGGTGHEYYARKLWEAQ....................
V7CD35_Pvulgaris_TGH                  LPHHSG....GKLYESNPLFSFLSGGTGHEYYARKLWEAQ....................
A0A1S3UTP2_Vradiata_TGH               VPHHSG....GKLYESNPLFSFLSGGTGHEYYARKLWEAQ....................
A0A1S2YR44_Carietinum_TGH             VPRHSG....GKLYESNPLFNFLSGGTGHDYYARKLWEAQ....................
G7JT16_Mtruncatula_TGH                VPHHSG....GKLYESNPLFNFLSGGTGHDYYARKLWEAQ....................
A0A1U8FWC2_Cannuum_TGH                VPLHAA....GKLLEFNPLFGFLNGGEGHEYYARKLWEER....................
V4TW69_Cclementina_TGH                VPHHPG....GKLFESNPLFSFLTGGNGHDYYARKLWEAR....................
A0A1R3G1K6_Ccapsularis_TGH            VPHHPG....GKLFESNPLFSFLSGGNGHDYYARKLWEEH....................
A0A1R3H0J1_Colitorius_TGH             VPHHPG....GKLFESNPLFSFLSGGNGHDYYARKLWEEH....................
A0A0D2QRF0_Graimondii_TGH             VPHHPG....GKLFESNPLFSFLSGGDGHDYYERRLWEEH....................
A0A067JJA6_Jcurcas_TGH                VPHHPS....GQLFESNPLFSFLNGGNGQDYYARKLWEER....................
B9RE60_Rcommunis_TGH                  VPHHPG....GKLFESNPLFSFLNGGNGHEYYARKLWEEC....................
SapurV1A.0789s0080.1_SSpurpurea_TGH   LPHHPG....GKLFESNPLFSFLTGGNGHDYYSRKLWEEQ....................
A0A2I4E4A1_Jregia_TGH                 VPHHPG....GKLFESNPLFNFLTGGNGHDYYSRKLWEER....................
F6GTG8_Vvinifera_TGH                  VPHHHA....GKLFESNPLFSFLTGGNGCDYYARKLWEER....................
W9S666_Mnotabilis_TGH                 EPHHPG....GKLFESNPLFSFLRGGNGHDYYTRKLWEAR....................
A0A068TQE9_Ccanephora_TGH             VPHHTA....GKLFESNPLFAFLFGGNGQDYYTRKLWEER....................
A0A200Q3N1_Mcordata_TGH               EPYHSA....GKLFEKNPLFSFLDGGKGHDYYARKLWEEQ....................
A0A1U8AEE6_Nnucifera_TGH              KPHHAS....GKLFESNPLFCFLTGGKGHDYYARKLWEEQ....................
A0A0A0LSK9_Csativus_TGH               IPHHAG....GKLFESNPLFSFLNGGTGQEYYSRKLWEEQ....................
A0A059A5E4_Egrandis_TGH               EPHHSG....GKLFESNPLFSFLYGGDFCGYYERRVWEEK....................
A0A218XZU2_Pgranatum_TGH              EPRHPG....GKLFESNQLYSFLIGGNFHDYYVRKLWEER....................
A0A022RKT3_Eguttata_TGH               IPHHPA....GKLFESNPLFDFLRGGNGSDFYIRKLWEER....................
A0A0J8B830_Bvulgaris_TGH              VPQHPA....GKLIESNPLFNFLNGGSGHEYYARKLWEAK....................
Kalax.0309s0034.1_Klaxiflora_TGH      ILVHSG....GKLFESNPLFSFLRGGEGHDYYLKKLWEER....................
A0A124SAU9_Ccardunculus_TGH           VPHHPA....GPLFESNALFDFLNGGNGHDYYTRKLWEAR....................
W1PSA4_Atrichopoda_TGH                EPHHPV....GRAFEGNPLFSFLCGGDSEEYYRRRLWEEQ....................
A0A0K9NIC3_Zmarina_TGH                KSCHST....GKLFEGNPLFCFLYGGNGCDYYTRRLWEEQ....................
A0A2C9VXU1_Mesculenta_TGH             VPHHPG....GKLFESNPLFSFLNGGNGHDYYARKLWEER....................
A0A0U9HQK1_Knitens_TGH                VPRHVS....GEKLEGSPEFGFLFGGPGKEYYA...WKKS............VEEPGGGF
Sphfalx0151s0047.1_SSfallax_TGH       IPFHAS....GQRFEGNPAFAFLFGGEGHDYYVRRLWEEE....................
D8QX10_Smoellendorffii_TGH            VPVHVA....GTQLEENPLFQFLFGGMGGDYYTRRLWEER....................
E1ZI32_Cvariabilis_TGH                VPLH......GIIFEKEGQHAFLSGGEGAAYYRWKVHSLR........PHQQ........
C1FFR5_Mcommoda_TGH                   RAFHPPGTEPGPAFEGDPKFSFLFGGPDEGYYRYKLGECRRELGAGTG............
A0A086T442_Achrysogenum_TGH           ASAK........................................................
K3V9N2_Fpseudograminearum_TGH         VSSK........................................................
W7LTD5_Gmoniliformis_TGH              MSSK........................................................
G9NMW3_Hatroviridis_TGH               KSSK........................................................
A0A0G0A2Q9_Tharzianum_TGH             MSPK........................................................
A0A1T3CLH2_Tguizhouense_TGH           ISSK........................................................
A0A084QRJ1_Schlorohalonata_TGH        VPSR........................................................
J4KPK4_Bbassiana_TGH                  TSTK........................................................
A0A2H4SR46_Cmilitaris_TGH             TWTK........................................................
A0A167VSL6_Cfumosorosea_TGH           ISAN........................................................
A0A179G8E9_Pchlamydosporia_TGH        KSSK........................................................
A0A0L0MXB1_Tophioglossoides_TGH       KSAK........................................................
Q2GSN0_Cglobosum_TGH                  ISAK........................................................
G2QES8_Mthermophila_TGH               VSAK........................................................
A0A175VUX4_Mmycetomatis_TGH           VSSK........................................................
G2QRS6_Tterrestris_TGH                VSAK........................................................
B2ARI9_Panserina_TGH                  VSSK........................................................
F7W019_Smacrospora_TGH                VSSK........................................................
A0A1J7J5C9_Cligniaria_TGH             KSSK........................................................
A0A0G2IFE8_Dampelina_TGH              KSSE........................................................
A0A194W011_Vmali_TGH                  KSSK........................................................
R8BKG4_Tminima_TGH                    KSNK........................................................
G0SA63_Cthermophilum_TGH              VSSK........................................................
J3NFM2_Ggraminis_TGH                  RPSR........................................................
A0A0C4DZ99_Mpoae_TGH                  KSSR........................................................
L7IH22_Moryzae_TGH                    KSTR........................................................
A0A1Y2WZC5_Daldiniasp._TGH            KSNK........................................................
A0A1Y2V8J1_Hypoxylon                  KPSK........................................................
A0A1Y2W1E2_Hypoxylon                  KSSK........................................................
A0A1W2TFH3_Rnecatrix_TGH              KPSK........................................................
W3WZ77_Pfici_TGH                      KTSK........................................................
A0A1Y2DPV9_Pvexata_TGH                KSSR........................................................
A0A136JHX1_Mbolleyi_TGH               VSSK........................................................
S3CR73_Glozoyensis_TGH                KSSK........................................................
K1WET1_Mbrunnea_TGH                   VSSK........................................................
A0A218YYN9_Mcoronariae_TGH            VSSK........................................................
A0A194XKL9_Pscopiformis_TGH           KSSK........................................................
A0A0C3DJQ0_Omaius_TGH                 TSSK........................................................
A7ECA3_Ssclerotiorum_TGH              KSAK........................................................
W9CY85_Sborealis_TGH                  KSAK........................................................
A0A094E7L1_Pseudogymnoascus           TSAK........................................................
A0A1B8CGM9_Pseudogymnoascus           TSAK........................................................
A0A094H174_Pseudogymnoascus           TSIK........................................................
Q0D178_Aterreus_TGH                   KSRK........................................................
A0A1L9NNK2_Atubingensis_TGH           KSSK........................................................
A1CQ12_Aclavatus_TGH                  KSCK........................................................
A0A1L9RVU1_Awentii_TGH                KSSK........................................................
A0A1E3BNS4_Acristatus_TGH             KSSK........................................................
I7ZRW9_Aoryzae_TGH                    KCSK........................................................
A0A0F8UC36_Aochraceoroseus_TGH        KSKK........................................................
Q5BE58_Enidulans_TGH                  KSKK........................................................
A0A0A2KYD8_Pitalicum_TGH              KPAK........................................................
A0A1V6QJR5_Pantarcticum_TGH           KSAK........................................................
A0A1Q5UD44_Psubrubescens_TGH          KSTK........................................................
A0A1V6P0U0_Pdecumbens_TGH             KPAK........................................................
A0A0F4YXS3_Remersonii_TGH             KSSK........................................................
```

```
B8LVN1_Tstipitatus_TGH                KSSK........................................................
A0A225B272_Tatroroseus_TGH            KSSK........................................................
A0A0H1BJD0_Bsilverae_TGH              KSTK........................................................
A0A1B7NTS8_Esp._TGH                   KSAK........................................................
C0RYG0_Pbrasiliensis_TGH              KSTK........................................................
C4JTX0_Ureesii_TGH                    ESSK........................................................
R7Z059_Capollinis_TGH                 KSAK........................................................
A0A1S8BM68_Dseriata_TGH               SSSK........................................................
K2RXL0_Mphaseolina_TGH                KSSK........................................................
A0A165JKY3_Xheveae_TGH                KSAK........................................................
A0A0G2GV44_Pchlamydospora_TGH         KSSK........................................................
F0X9I8_Gclavigera_TGH                 TSSK........................................................
S3BTH5_Opiceae_TGH                    RSSK........................................................
A0A0C2J2D4_Sbrasiliensis_TGH          KSSK........................................................
A0A167VN43_Sinsectorum_TGH            TSAK........................................................
F7W020_Smacrospora_TGH                SSSA........................................................
A0A151N705_Amississippiensis_TGH      RPVH........................................................
A0A286X9R8_Cporcellus_TGH             RPVH........................................................
A0A1S3ARH5_Eeuropaeus_TGH             RPVH........................................................
Q9BRR8_Hsapiens_TGH                   RPVH........................................................
Q9DBM1_Mmusculus_TGH                  RPVH........................................................
L5LZ46_Mdavidii_TGH                   RPVH........................................................
S7PC93_Mbrandtii_TGH                  RPVH........................................................
A0A1S3FZ16_Dordii_TGH                 ............................................................
F6Z6Q3_Mdomestica_TGH                 RPVH........................................................
G3WTW2_Sharrisii_TGH                  RPIH........................................................
Q21827_Celegans_TGH                   RGDH........................................................
Q9VUA0_Dmelanogaster_TGH              LQRRF..........GR...........................................
```

```
                                                                                                 280
                                                                                                   .
Aco012726.1_Acomosus_TGH                 ............................................................PFKYDPAKQER
A0A199V6F8_Acomosus_TGH                  ............................................................PFKYDPAKQER
M0TMB2_Macuminata_TGH                    ............................................................PFKDDPAKQER
A0A2H3XE04_Pdactylifera_TGH              ............................................................PFKNDPAKQER
A0A0Q3H630_Bdistachyon_TGH               ............................................................PFRNEPAKQAR
J3MEA3_Obrachyantha_TGH                  ............................................................PFRNDPAKQAR
B8B2G4_Osativa_TGH                       ............................................................PFRNDPAKQAR
K3XV17_Sitalica_TGH                      ............................................................PFSNDPAKQAR
Q8GXN9_Athaliana_TGH                     ............................................................PFKDDPAKQER
Bol036258_Boleraceacapitata_TGH          ............................................................PFKDDPAKQER
V4MKB5_Esalsugineum_TGH                  ............................................................PFKDDMAKQER
A0A087GCN0_Aalpina_TGH                   ............................................................PFKDEPAKQER
A0A151SPT8_Ccajan_TGH                    ............................................................PFKDDLAKQER
I1K185_Gmax_TGH                          ............................................................PFKDDPAKQER
V7CD35_Pvulgaris_TGH                     ............................................................PFKDDPAKEER
A0A1S3UTP2_Vradiata_TGH                  ............................................................PFKDDPAKQER
A0A1S2YR44_Carietinum_TGH                ............................................................PFKADPAKQER
G7JT16_Mtruncatula_TGH                   ............................................................PFKDDPAKQGR
A0A1U8FWC2_Cannuum_TGH                   ............................................................PFQDDPAKQER
V4TW69_Cclementina_TGH                   ............................................................PFQDDPVKQER
A0A1R3G1K6_Ccapsularis_TGH               ............................................................PFKDDPAKQAR
A0A1R3H0J1_Colitorius_TGH                ............................................................PFKDDPAKQER
A0A0D2QRF0_Graimondii_TGH                ............................................................PFKDDPAKQER
A0A067JJA6_Jcurcas_TGH                   ............................................................PFKDDPAKQER
B9RE60_Rcommunis_TGH                     ............................................................PFKDDPAKQER
SapurV1A.0789s0080.1_SSpurpurea_TGH      ............................................................PFQDDPGKQAR
A0A2I4E4A1_Jregia_TGH                    ............................................................PFNDDPVKQER
F6GTG8_Vvinifera_TGH                     ............................................................PFKEDPAKQER
W9S666_Mnotabilis_TGH                    ............................................................PFNDDPAKQER
A0A068TQE9_Ccanephora_TGH                ............................................................PFQDDPAKQAR
A0A200Q3N1_Mcordata_TGH                  ............................................................PFKADPEKQER
A0A1U8AEE6_Nnucifera_TGH                 ............................................................PFIDDPAKQER
A0A0A0LSK9_Csativus_TGH                  ............................................................PFKDDSAKQER
A0A059A5E4_Egrandis_TGH                  ............................................................PFGDDPAKQDR
A0A218XZU2_Pgranatum_TGH                 ............................................................PFKDNPAKQER
A0A022RKT3_Eguttata_TGH                  ............................................................PFHDDPEKQKR
A0A0J8B830_Bvulgaris_TGH                 ............................................................PYKDDPAKQER
Kalax.0309s0034.1_Klaxiflora_TGH         ............................................................PFQNDVAKQER
A0A124SAU9_Ccardunculus_TGH              ............................................................PFQHDPAKQER
W1PSA4_Atrichopoda_TGH                   ............................................................PFKNDPAKQER
A0A0K9NIC3_Zmarina_TGH                   ............................................................PFEKEPAKQKR
A0A2C9VXU1_Mesculenta_TGH                ............................................................PFKDDPAKQER
A0A0U9HQK1_Knitens_TGH                   RAEFA......DAKSSGTDQKAGSGP..................................PFAADPAKQAR
Sphfalx0151s0047.1_SSfallax_TGH          ............................................................FFKNDPAKQAR
D8QX10_Smoellendorffii_TGH               .................................................FF.........PFEDDPAKQAR
E1ZI32_Cvariabilis_TGH                   ...........................................................................
C1FFR5_Mcommoda_TGH                      ................................PPGTKVPEKNVKG...............P..........
A0A086T442_Achrysogenum_TGH              ............................................................PYADDEAKRQR
K3V9N2_Fpseudograminearum_TGH            ............................................................PYADNEEKKAR
W7LTD5_Gmoniliformis_TGH                 ............................................................PYADNEEKKAR
G9NMW3_Hatroviridis_TGH                  ............................................................PYADNEAKRAR
A0A0G0A2Q9_Tharzianum_TGH                ............................................................PYADDETKRAR
A0A1T3CLH2_Tguizhouense_TGH              ............................................................PYADDETKRAR
A0A084QRJ1_Schlorohalonata_TGH           ............................................................PYADDGAKRAR
J4KPK4_Bbassiana_TGH                     ............................................................PYADDERKRNR
A0A2H4SR46_Cmilitaris_TGH                ............................................................PYADDERKRER
A0A167VSL6_Cfumosorosea_TGH              ............................................................PYADDERKRNR
A0A179G8E9_Pchlamydosporia_TGH           ................................................P...........PYADDEAKRGR
A0A0L0MXB1_Tophioglossoides_TGH          ............................................................PYADDESKRTR
Q2GSN0_Cglobosum_TGH                     ............................................................PYRDDEAKRSR
G2QES8_Mthermophila_TGH                  ............................................................PYRDDEAKRAR
A0A175VUX4_Mmycetomatis_TGH              ............................................................PYADDEAKRSR
G2QRS6_Tterrestris_TGH                   ............................................................PYADDEAKRSR
B2ARI9_Panserina_TGH                     ............................................................PYADDEKKRAR
F7W019_Smacrospora_TGH                   ............................................................PYSEDEEKRAR
A0A1J7J5C9_Cligniaria_TGH                ............................................................PYSEDDDKRSR
A0A0G2IFE8_Dampelina_TGH                 ............................................................PYGDNEDKRSR
A0A194W011_Vmali_TGH                     ............................................................PYGDNDDKRSR
R8BKG4_Tminima_TGH                       ............................................................PYGDDEAKRSR
G0SA63_Cthermophilum_TGH                 ............................................................PYADDEAKRSR
J3NFM2_Ggraminis_TGH                     ............................................................PYADNEAKRSR
A0A0C4DZ99_Mpoae_TGH                     ............................................................PYADNEAKRSR
L7IH22_Moryzae_TGH                       ............................................................PYADNEDKRKR
A0A1Y2WZC5_Daldiniasp._TGH               ............................................................PYADDEAKRAR
A0A1Y2V8J1_Hypoxylon                     .........................................................GGGPYADDEAKRAR
A0A1Y2W1E2_Hypoxylon                     ............................................................PYADDEAKRAR
A0A1W2TFH3_Rnecatrix_TGH                 ............................................................PYADDEAKRGR
W3WZ77_Pfici_TGH                         ............................................................PYADDEAKRTR
A0A1Y2DPV9_Pvexata_TGH                   ............................................................PYADNESKRSR
A0A136JHX1_Mbolleyi_TGH                  ............................................................PYADNEAKRTR
S3CR73_Glozoyensis_TGH                   ............................................................PYGEDEAKRSR
K1WET1_Mbrunnea_TGH                      ............................................................PYGEDEAKRSR
A0A218YYN9_Mcoronariae_TGH               ............................................................PYGEDQTKRGR
A0A194XKL9_Pscopiformis_TGH              ............................................................PYGEDEAKRNR
A0A0C3DJQ0_Omaius_TGH                    ............................................................PYGDDLPKRSR
A7ECA3_Ssclerotiorum_TGH                 ............................................................VYGEDEAKRIR
W9CY85_Sborealis_TGH                     ............................................................VYTEDEAKRIR
A0A094E7L1_Pseudogymnoascus              ............................................................PYSDNEEKRLR
A0A1B8CGM9_Pseudogymnoascus              ............................................................PYSDNEEKRSR
A0A094H174_Pseudogymnoascus              ............................................................PYSDNDEKRSR
Q0D178_Aterreus_TGH                      ............................................................PYSEDPDKRTR
A0A1L9NNK2_Atubingensis_TGH              ............................................................PYAEDLSKRDR
A1CQ12_Aclavatus_TGH                     ............................................................PYSEDQGKRAR
A0A1L9RVU1_Awentii_TGH                   ............................................................PYAEDEGKRAR
A0A1E3BNS4_Acristatus_TGH                ............................................................PYSEDPDKRVR
I7ZRW9_Aoryzae_TGH                       ............................................................PYSEDPDKRSR
A0A0F8UC36_Aochraceoroseus_TGH           ............................................................PYSDDETKRAR
Q5BE58_Enidulans_TGH                     ............................................................PYSEDESKRTR
A0A0A2KYD8_Pitalicum_TGH                 ............................................................PYQEDEKKRAR
A0A1V6QJR5_Pantarcticum_TGH              ............................................................PYSEDEKKRAR
A0A1Q5UD44_Psubrubescens_TGH             ............................................................PYAEDEKKRAR
A0A1V6P0U0_Pdecumbens_TGH                ............................................................PYAEDEHKRAR
A0A0F4YXS3_Remersonii_TGH                ............................................................PYSEDEGKRAR
```

```
B8LVN1_Tstipitatus_TGH                ................................................PYSEDENKRAR
A0A225B272_Tatroroseus_TGH            ................................................PYSDDENKRAR
A0A0H1BJD0_Bsilverae_TGH              ................................................PYAEDEAKRSR
A0A1B7NTS8_Esp._TGH                   ................................................PYAEDEAKRSR
C0RYG0_Pbrasiliensis_TGH              ................................................PYAEDEAKRSR
C4JTX0_Ureesii_TGH                    ................................................PYSEDESKRSR
R7Z059_Capollinis_TGH                 ................................................PYAEDERKRAR
A0A1S8BM68_Dseriata_TGH               ................................................PYSEDEKKRAR
K2RXL0_Mphaseolina_TGH                ................................................PYADDEKKRAR
A0A165JKY3_Xheveae_TGH                ................................................PYAEDESKRSR
A0A0G2GV44_Pchlamydospora_TGH         ................................................PYAEDESKRAR
F0X9I8_Gclavigera_TGH                 ................................................PYQDNEAKRSR
S3BTH5_Opiceae_TGH                    ................................................PYQDNEGKRSR
A0A0C2J2D4_Sbrasiliensis_TGH          ................................................PYQDNEAKRSR
A0A167VN43_Sinsectorum_TGH            ................................................PYQDDEAKRAR
F7W020_Smacrospora_TGH                ...........................................................
A0A151N705_Amississippiensis_TGH      ................................................PFAKNPEKQKR
A0A286X9R8_Cporcellus_TGH             ................................................PFAKDPEKQKR
A0A1S3ARH5_Eeuropaeus_TGH             ................................................PFAKDPEKQKR
Q9BRR8_Hsapiens_TGH                   ................................................PFAKDPEKQKR
Q9DBM1_Mmusculus_TGH                  ................................................PFAKDPEKQRR
L5LZ46_Mdavidii_TGH                   ................................................PFAKDPEKQKR
S7PC93_Mbrandtii_TGH                  ................................................PFAKDPEKQKR
A0A1S3FZ16_Dordii_TGH                 ................................................PFAKDPEKQKR
F6Z6Q3_Mdomestica_TGH                 ................................................PFTKDPEKQKR
G3WTW2_Sharrisii_TGH                  ................................................PFAKDPEKQKR
Q21827_Celegans_TGH                   ................................................EFPDEPMRQAR
Q9VUA0_Dmelanogaster_TGH              .....AEEQPK..............................GKDL...PFIADEAKQLR
```

```
                                            290        300
Aco012726.1_Acomosus_TGH                 FEQFLREK...MSETDRARERL..DF...............................RFV
A0A199V6F8_Acomosus_TGH                  FEQFLREK...MSETDRARERL..DF...............................RFV
M0TMB2_Macuminata_TGH                    FEQFLKDK...MSENDRARERL..DF...............................QFI
A0A2H3XE04_Pdactylifera_TGH              FEYFLKDK...MSEADRARERL..DF...............................FFI
A0A0Q3H630_Bdistachyon_TGH               FEQFLKEK...MSEADRARERL..DF...............................RFV
J3MEA3_Obrachyantha_TGH                  FEQFLKDK...MSDADRARERL..DF...............................RFV
B8B2G4_Osativa_TGH                       FEQFLKDK...MSDADRARERL..DF...............................RFV
K3XV17_Sitalica_TGH                      FEQFLKDK...MSEADRARERL..DF...............................RFV
Q8GXN9_Athaliana_TGH                     FEQFLKEK...MSESARAQERL..DF...............................QF.
Bol036258_Boleraceacapitata_TGH          FEQFLKEK...MSEAARAQERL..DF...............................QF.
V4MKB5_Esalsugineum_TGH                  FEQFLKEK...MSESARAQERL..DF...............................QF.
A0A087GCN0_Aalpina_TGH                   FEQFLKEK...MSESSRAQERL..DF...............................QF.
A0A151SPT8_Ccajan_TGH                    FEQFLKEK...MSEAARAQERL..SF...............................QF.
I1K185_Gmax_TGH                          FEQFLKEK...MSEVARAQERL..SF...............................QF.
V7CD35_Pvulgaris_TGH                     FEQFLKEK...MSEAARAQERL..SF...............................QF.
A0A1S3UTP2_Vradiata_TGH                  FEQFLKEK...MSEAARAQERL..SF...............................QF.
A0A1S2YR44_Carietinum_TGH                FEQFIKEK...MSEDARAQERL..SF...............................EF.
G7JT16_Mtruncatula_TGH                   FEQFIKEK...MSEAARAQERL..SF...............................EF.
A0A1U8FWC2_Cannuum_TGH                   FEQFLKEK...MSEAARARERL..EF...............................QF.
V4TW69_Cclementina_TGH                   FERFLKEK...MSEAARARERL..DF...............................LF.
A0A1R3G1K6_Ccapsularis_TGH               FERFLKEK...MSEGARARERL..DF...............................QF.
A0A1R3H0J1_Colitorius_TGH                FERFLKEK...MSEEARARERL..DF...............................QF.
A0A0D2QRF0_Graimondii_TGH                FEQFLKEK...MSEAARAREKL..DF...............................QF.
A0A067JJA6_Jcurcas_TGH                   FERFLKEK...MSEAARARERL..DF...............................QF.
B9RE60_Rcommunis_TGH                     FEQFLKEK...MSEAARARERL..DF...............................QF.
SapurV1A.0789s0080.1_SSpurpurea_TGH      FEQFLKEK...MSEAARARERL..DF...............................QL.
A0A2I4E4A1_Jregia_TGH                    FEQFLKAK...MSEAARAHERL..DF...............................QF.
F6GTG8_Vvinifera_TGH                     FELFLKEK...MSEAARAREKL..DF...............................EF.
W9S666_Mnotabilis_TGH                    FELFLKEK...MSEAARACERL..DF...............................QF.
A0A068TQE9_Ccanephora_TGH                FEQFLKEK...MSEAARARERL..EF...............................QF.
A0A200Q3N1_Mcordata_TGH                  FERFLKEK...MSEADRAREKL..DF................G..............QF.
A0A1U8AEE6_Nnucifera_TGH                 FELFLKEK...MSEAERARERL..DF...............................QF.
A0A0A0LSK9_Csativus_TGH                  FEQFLKEK...MSEAARARERL..DF...............................QF.
A0A059A5E4_Egrandis_TGH                  FEQFLKEK...MSEAARARERL..DF.......................GHG.....QF.
A0A218XZU2_Pgranatum_TGH                 FEQFLKEK...MSESARARERL..DF...............................QF.
A0A022RKT3_Eguttata_TGH                  FEQFMKEK...MAESARARERL..EF...............................QF.
A0A0J8B830_Bvulgaris_TGH                 FEQFLKDK...MTEGARARERL..DF...............................QF.
Kalax.0309s0034.1_Klaxiflora_TGH         FEQFLKQK...MTEDARARERL..DF...............................QF.
A0A124SAU9_Ccardunculus_TGH              FEQYLKEK...MSEAARARERL..EF...............................QF.
W1PSA4_Atrichopoda_TGH                   FEQFLKDK...MSELHRAQERL..DF...............................RF.
A0A0K9NIC3_Zmarina_TGH                   FEQFIKDK...WSENDRARERL..DF...............................QF.
A0A2C9VXU1_Mesculenta_TGH                FERFLKEK...MSESARARERL..DF...............................LF.
A0A0U9HQK1_Knitens_TGH                   YETFLQER...LTEEERLREKE..EF.................GLKPAP........RF.
Sphfalx0151s0047.1_SSfallax_TGH          FEQFMKDK...LSEIQAAHEMM..EF...............................RF.
D8QX10_Smoellendorffii_TGH               YEQFLKDK...LTEAQRAQEIL..EF...............................RF.
E1ZI32_Cvariabilis_TGH                   ............................................................
C1FFR5_Mcommoda_TGH                      ........F..LEDPKEARERE..ER..................................
A0A086T442_Achrysogenum_TGH              YREYLE.....MTDEDFLREMN..EF...............................RF.
K3V9N2_Fpseudograminearum_TGH            YRTYLE.....MAYDDFARELS..EF...............................RF.
W7LTD5_Gmoniliformis_TGH                 YRTYLE.....MTFDDFARELS..EF...............................RF.
G9NMW3_Hatroviridis_TGH                  YKRYLE.....TSDDEFLQEMT..EF...............................RF.
A0A0G0A2Q9_Tharzianum_TGH                YKRYLE.....MSDDEFLREMT..EF...............................RF.
A0A1T3CLH2_Tguizhouense_TGH              YKRYLE.....MSDDEFLREMT..EF...............................RF.
A0A084QRJ1_Schlorohalonata_TGH           FRTYLE.....VRDEDFLREMT..EF...............................RF.
J4KPK4_Bbassiana_TGH                     YRAYLE.....LSDDDYLREMT..EF...............................RF.
A0A2H4SR46_Cmilitaris_TGH                YRAYLE.....LSDDDYLREMT..EF...............................RF.
A0A167VSL6_Cfumosorosea_TGH              YRAYLE.....YADDDYLREMT..EF...............................RF.
A0A179G8E9_Pchlamydosporia_TGH           YRKYLM.....TFDDDYIREMN..EF...............................RF.
A0A0L0MXB1_Tophioglossoides_TGH          YRRYLE.....MTDEGFLREVN..EF...............................RF.
Q2GSN0_Cglobosum_TGH                     YISFLE.....TNSEEWLRELH..EF...............................RF.
G2QES8_Mthermophila_TGH                  YIAFLE.....MNNEEWLREMH..EF...............................RF.
A0A175VUX4_Mmycetomatis_TGH              YIAFLE.....VNSEDWLREMH..EF...............................RF.
G2QRS6_Tterrestris_TGH                   YIAFLE.....MNSEEWLRELQ..EF...............................RF.
B2ARI9_Panserina_TGH                     YIAFLE.....MNSEDWLRELT..EF...............................RF.
F7W019_Smacrospora_TGH                   YRGYLE.....LTDEEWLREFH..EF...............................RF.
A0A1J7J5C9_Cligniaria_TGH                YRAYLE.....MSLDDWLKEFY..EF...............................RF.
A0A0G2IFE8_Dampelina_TGH                 YRGYLE.....MTNDEWIQEFH..EF...............................RF.
A0A194W011_Vmali_TGH                     YRAYLE.....MTTDEWIQEMH..EF...............................RF.
R8BKG4_Tminima_TGH                       YRAYLE.....MNNDDWLREFQ..EF...............................RF.
G0SA63_Cthermophilum_TGH                 YLSFLE.....MKDDDWLRELH..EF...............................RF.
J3NFM2_Ggraminis_TGH                     YRAYLE.....MSNDDWLREFH..EF...............................RF.
A0A0C4DZ99_Mpoae_TGH                     YRSYLE.....MSNDDWLREFH..EF...............................RF.
L7IH22_Moryzae_TGH                       YRAYLE.....VKDDDWLREFY..EF...............................RF.
A0A1Y2WZC5_Daldiniasp._TGH               YRKYLE.....VSVEDWLRELH..EF...............................RF.
A0A1Y2V8J1_Hypoxylon                     YRAYLE.....VGVEDWLRELH..EF...............................RF.
A0A1Y2W1E2_Hypoxylon                     YRGYLE.....ESSEEYLREMH..EF...............................RF.
A0A1W2TFH3_Rnecatrix_TGH                 YRAYLE.....TGAQAWARELH..EF...............................RF.
W3WZ77_Pfici_TGH                         YRTYLE.....QSSQDWLRELR..EF...............................RF.
A0A1Y2DPV9_Pvexata_TGH                   YRAYLE.....MSSDDWLRELR..EF...............................RF.
A0A136JHX1_Mbolleyi_TGH                  YRQFLE.....FKDNDWTRELN..EF...............................RF.
S3CR73_Glozoyensis_TGH                   YRNYLE.....FSKNDWLKELQ..EF...............................RF.
K1WET1_Mbrunnea_TGH                      YRAYLE.....VSKDDWLKELR..EF...............................RF.
A0A218YYN9_Mcoronariae_TGH               YRAYLE.....VSRDDWLKELR..EF...............................RF.
A0A194XKL9_Pscopiformis_TGH              YRAYLE.....VSRIDWLKELK..EF...............................RF.
A0A0C3DJQ0_Omaius_TGH                    YRAYLE.....MSKDDWLKELR..EF...............................RF.
A7ECA3_Ssclerotiorum_TGH                 YRAYLE.....TNKEDWLKELK..EF...............................RF.
W9CY85_Sborealis_TGH                     YRAYLE.....ASKEDWLKELG..EF...............................RF.
A0A094E7L1_Pseudogymnoascus              YKIYLE.....VAKEDWIRELQ..EF...............................RF.
A0A1B8CGM9_Pseudogymnoascus              YKIYLE.....VAKEDWIRELQ..EF...............................RF.
A0A094H174_Pseudogymnoascus              YKIYLE.....VAKEDWIRELQ..EF...............................RF.
Q0D178_Aterreus_TGH                      YRTFLE.....STTDEWIAELN..EF...............................RF.
A0A1L9NNK2_Atubingensis_TGH              YRTFLQ.....STTDEWAAELH..EF...............................RF.
A1CQ12_Aclavatus_TGH                     YRTFLE.....STTDEWVTELH..EF...............................RF.
A0A1L9RVU1_Awentii_TGH                   YRTFLE.....SSTDEWVNELN..EF...............................RF.
A0A1E3BNS4_Acristatus_TGH                YRSFLE.....STTDEWVSELH..EF...............................RF.
I7ZRW9_Aoryzae_TGH                       YRTFLQ.....SSTDEWVAELH..EF...............................RF.
A0A0F8UC36_Aochraceoroseus_TGH           YRTFLE.....TSTDEWITELH..EF...............................RF.
Q5BE58_Enidulans_TGH                     YRTFLE.....FTTNEWVTELE..EF...............................RF.
A0A0A2KYD8_Pitalicum_TGH                 YQIFLE.....SSTDEWVIEMQ..EF...............................RF.
A0A1V6QJR5_Pantarcticum_TGH              YRVFLE.....SNTDEWVTEMQ..EF...............................RF.
A0A1Q5UD44_Psubrubescens_TGH             YRTFLE.....SSTDEWVNEMQ..EF...............................RF.
A0A1V6P0U0_Pdecumbens_TGH                YRTFLE.....SSTDDWVAEMH..EF...............................RF.
A0A0F4YXS3_Remersonii_TGH                YRTFLE.....STTDEWVAEMQ..EF...............................RF.
```

```
B8LVN1_Tstipitatus_TGH                 YRTFLE.....STTDDWVREMQ..EF...............................RF.
A0A225B272_Tatroroseus_TGH             YRTFLE.....STTDEWVREMQ..EF...............................RF.
A0A0H1BJD0_Bsilverae_TGH               YRSFLE.....TTTDDWLIELQ..EF...............................RF.
A0A1B7NTS8_Esp._TGH                    YRSFLE.....ATTDDWIMELQ..EF...............................RF.
C0RYG0_Pbrasiliensis_TGH               YRSFLE.....ATTDNWIAELN..EF...............................RF.
C4JTX0_Ureesii_TGH                     YRSFLE.....ASTNEWVVELN..EF...............................RF.
R7Z059_Capollinis_TGH                  YRAFIE.....MSTDEWVKELQ..EF...............................RF.
A0A1S8BM68_Dseriata_TGH                YRGFLE.....VSTDDWARELH..EF...............................RF.
K2RXL0_Mphaseolina_TGH                 YRGFLE.....MSTDDWVRELQ..EF...............................RF.
A0A165JKY3_Xheveae_TGH                 YRTFLE.....MSTEDWVKELH..EF...............................RF.
A0A0G2GV44_Pchlamydospora_TGH          YRTFLE.....MPKDNWITEMY..EF...............................RF.
F0X9I8_Gclavigera_TGH                  YRAYLE.....LSNDDWLREFH..EF...............................RF.
S3BTH5_Opiceae_TGH                     YRAYLE.....VSDDDWLREFY..EF...............................RF.
A0A0C2J2D4_Sbrasiliensis_TGH           YRAYLE.....MKDDDWLREAH..EF...............................RF.
A0A167VN43_Sinsectorum_TGH             YRAYLE.....VSDDDWLRECH..EF...............................RF.
F7W020_Smacrospora_TGH                 ........................EL...............................VS.
A0A151N705_Amississippiensis_TGH       YENYVES....MTEWEQGREQE..EF...............................RF.
A0A286X9R8_Cporcellus_TGH              YEKFLLH....MTEWERGRERE..EF...............................RF.
A0A1S3ARH5_Eeuropaeus_TGH              YEEFLVN....MTEWERGRERD..EF...............................RF.
Q9BRR8_Hsapiens_TGH                    YDEFLVH....MTEWERGRERD..EF...............................RF.
Q9DBM1_Mmusculus_TGH                   YEEFLVH....MTEWERSRERE..EF...............................RF.
L5LZ46_Mdavidii_TGH                    YEKFLAN....VSQ...........................................RF.
S7PC93_Mbrandtii_TGH                   YEKFLAN....MTEWERGRERE..EF...............................RF.
A0A1S3FZ16_Dordii_TGH                  YEEYLVH....MTEWERGRERE..EF...............................RF.
F6Z6Q3_Mdomestica_TGH                  YEKYLGN....MTEWERARERD..EL...............................RF.
G3WTW2_Sharrisii_TGH                   YEKYLGN....MTEWERGRERD..EF...............................RF.
Q21827_Celegans_TGH                    FKEFLHY....LTVWEWEWEKK..EFESKLTSDERGMLPEVQ..........IAAPIKF.
Q9VUA0_Dmelanogaster_TGH               YEKFVS...FLVTLSLWDREMEKKEF...............................RF.
```

```
Aco012726.1_Acomosus_TGH                ............................................................
A0A199V6F8_Acomosus_TGH                 ............................................................
M0TMB2_Macuminata_TGH                   ............................................................
A0A2H3XE04_Pdactylifera_TGH             ............................................................
A0A0Q3H630_Bdistachyon_TGH              ............................................................
J3MEA3_Obrachyantha_TGH                 ............................................................
B8B2G4_Osativa_TGH                      ............................................................
K3XV17_Sitalica_TGH                     ............................................................
Q8GXN9_Athaliana_TGH                    ............................................................
Bol036258_Boleraceacapitata_TGH         ............................................................
V4MKB5_Esalsugineum_TGH                 ............................................................
A0A087GCN0_Aalpina_TGH                  ............................................................
A0A151SPT8_Ccajan_TGH                   ............................................................
I1K185_Gmax_TGH                         ............................................................
V7CD35_Pvulgaris_TGH                    ............................................................
A0A1S3UTP2_Vradiata_TGH                 ............................................................
A0A1S2YR44_Carietinum_TGH               ............................................................
G7JT16_Mtruncatula_TGH                  ............................................................
A0A1U8FWC2_Cannuum_TGH                  ............................................................
V4TW69_Cclementina_TGH                  ............................................................
A0A1R3G1K6_Ccapsularis_TGH              ............................................................
A0A1R3H0J1_Colitorius_TGH               ............................................................
A0A0D2QRF0_Graimondii_TGH               ............................................................
A0A067JJA6_Jcurcas_TGH                  ............................................................
B9RE60_Rcommunis_TGH                    ............................................................
SapurV1A.0789s0080.1_SSpurpurea_TGH     ............................................................
A0A2I4E4A1_Jregia_TGH                   ............................................................
F6GTG8_Vvinifera_TGH                    ............................................................
W9S666_Mnotabilis_TGH                   ............................................................
A0A068TQE9_Ccanephora_TGH               ............................................................
A0A200Q3N1_Mcordata_TGH                 ............................................................
A0A1U8AEE6_Nnucifera_TGH                ............................................................
A0A0A0LSK9_Csativus_TGH                 ............................................................
A0A059A5E4_Egrandis_TGH                 ............................................................
A0A218XZU2_Pgranatum_TGH                ............................................................
A0A022RKT3_Eguttata_TGH                 ............................................................
A0A0J8B830_Bvulgaris_TGH                ............................................................
Kalax.0309s0034.1_Klaxiflora_TGH        ............................................................
A0A124SAU9_Ccardunculus_TGH             ............................................................
W1PSA4_Atrichopoda_TGH                  ............................................................
A0A0K9NIC3_Zmarina_TGH                  ............................................................
A0A2C9VXU1_Mesculenta_TGH               ............SCEIISNLDEDQEFDNIDFHNSDCFNLENENLDSDDNDGLAKDEESSM
A0A0U9HQK1_Knitens_TGH                  ............................................................
Sphfalx0151s0047.1_SSfallax_TGH         ............................................................
D8QX10_Smoellendorffii_TGH              ............................................................
E1ZI32_Cvariabilis_TGH                  ............................................................
C1FFR5_Mcommoda_TGH                     ............................................................
A0A086T442_Achrysogenum_TGH             ............................................................
K3V9N2_Fpseudograminearum_TGH           ............................................................
W7LTD5_Gmoniliformis_TGH                ............................................................
G9NMW3_Hatroviridis_TGH                 ............................................................
A0A0G0A2Q9_Tharzianum_TGH               ............................................................
A0A1T3CLH2_Tguizhouense_TGH             ............................................................
A0A084QRJ1_Schlorohalonata_TGH          ............................................................
J4KPK4_Bbassiana_TGH                    ............................................................
A0A2H4SR46_Cmilitaris_TGH               ............................................................
A0A167VSL6_Cfumosorosea_TGH             ............................................................
A0A179G8E9_Pchlamydosporia_TGH          ............................................................
A0A0L0MXB1_Tophioglossoides_TGH         ............................................................
Q2GSN0_Cglobosum_TGH                    ............................................................
G2QES8_Mthermophila_TGH                 ............................................................
A0A175VUX4_Mmycetomatis_TGH             ............................................................
G2QRS6_Tterrestris_TGH                  ............................................................
B2ARI9_Panserina_TGH                    ............................................................
F7W019_Smacrospora_TGH                  ............................................................
A0A1J7J5C9_Cligniaria_TGH               ............................................................
A0A0G2IFE8_Dampelina_TGH                ............................................................
A0A194W011_Vmali_TGH                    ............................................................
R8BKG4_Tminima_TGH                      ............................................................
G0SA63_Cthermophilum_TGH                ............................................................
J3NFM2_Ggraminis_TGH                    ............................................................
A0A0C4DZ99_Mpoae_TGH                    ............................................................
L7IH22_Moryzae_TGH                      ............................................................
A0A1Y2WZC5_Daldiniasp._TGH              ............................................................
A0A1Y2V8J1_Hypoxylon                    ............................................................
A0A1Y2W1E2_Hypoxylon                    ............................................................
A0A1W2TFH3_Rnecatrix_TGH                ............................................................
W3WZ77_Pfici_TGH                        ............................................................
A0A1Y2DPV9_Pvexata_TGH                  ............................................................
A0A136JHX1_Mbolleyi_TGH                 ............................................................
S3CR73_Glozoyensis_TGH                  ............................................................
K1WET1_Mbrunnea_TGH                     ............................................................
A0A218YYN9_Mcoronariae_TGH              ............................................................
A0A194XKL9_Pscopiformis_TGH             ............................................................
A0A0C3DJQ0_Omaius_TGH                   ............................................................
A7ECA3_Ssclerotiorum_TGH                ............................................................
W9CY85_Sborealis_TGH                    ............................................................
A0A094E7L1_Pseudogymnoascus             ............................................................
A0A1B8CGM9_Pseudogymnoascus             ............................................................
A0A094H174_Pseudogymnoascus             ............................................................
Q0D178_Aterreus_TGH                     ............................................................
A0A1L9NNK2_Atubingensis_TGH             ............................................................
A1CQ12_Aclavatus_TGH                    ............................................................
A0A1L9RVU1_Awentii_TGH                  ............................................................
A0A1E3BNS4_Acristatus_TGH               ............................................................
I7ZRW9_Aoryzae_TGH                      ............................................................
A0A0F8UC36_Aochraceoroseus_TGH          ............................................................
Q5BE58_Enidulans_TGH                    ............................................................
A0A0A2KYD8_Pitalicum_TGH                ............................................................
A0A1V6QJR5_Pantarcticum_TGH             ............................................................
A0A1Q5UD44_Psubrubescens_TGH            ............................................................
A0A1V6P0U0_Pdecumbens_TGH               ............................................................
A0A0F4YXS3_Remersonii_TGH               ............................................................
```

**B8LVN1_Tstipitatus_TGH**	....
**A0A225B272_Tatroroseus_TGH**	....
**A0A0H1BJD0_Bsilverae_TGH**	....
**A0A1B7NTS8_Esp._TGH**	....
**C0RYG0_Pbrasiliensis_TGH**	....
**C4JTX0_Ureesii_TGH**	....
**R7Z059_Capollinis_TGH**	....
**A0A1S8BM68_Dseriata_TGH**	....
**K2RXL0_Mphaseolina_TGH**	....
**A0A165JKY3_Xheveae_TGH**	....
**A0A0G2GV44_Pchlamydospora_TGH**	....
**F0X9I8_Gclavigera_TGH**	....
**S3BTH5_Opiceae_TGH**	....
**A0A0C2J2D4_Sbrasiliensis_TGH**	....
**A0A167VN43_Sinsectorum_TGH**	....
**F7W020_Smacrospora_TGH**	FDNNDVVVDDQN..
**A0A151N705_Amississippiensis_TGH**	....
**A0A286X9R8_Cporcellus_TGH**	....
**A0A1S3ARH5_Eeuropaeus_TGH**	....
**Q9BRR8_Hsapiens_TGH**	....
**Q9DBM1_Mmusculus_TGH**	....
**L5LZ46_Mdavidii_TGH**	....
**S7PC93_Mbrandtii_TGH**	....
**A0A1S3FZ16_Dordii_TGH**	....
**F6Z6Q3_Mdomestica_TGH**	....
**G3WTW2_Sharrisii_TGH**	....
**Q21827_Celegans_TGH**	....
**Q9VUA0_Dmelanogaster_TGH**	....

```
Aco012726.1_Acomosus_TGH                 ............................................................
A0A199V6F8_Acomosus_TGH                  ............................................................
M0TMB2_Macuminata_TGH                    ............................................................
A0A2H3XE04_Pdactylifera_TGH              ............................................................
A0A0Q3H630_Bdistachyon_TGH               ............................................................
J3MEA3_Obrachyantha_TGH                  ............................................................
B8B2G4_Osativa_TGH                       ............................................................
K3XV17_Sitalica_TGH                      ............................................................
Q8GXN9_Athaliana_TGH                     ............................................................
Bol036258_Boleraceacapitata_TGH          ............................................................
V4MKB5_Esalsugineum_TGH                  ............................................................
A0A087GCN0_Aalpina_TGH                   ............................................................
A0A151SPT8_Ccajan_TGH                    ............................................................
I1K185_Gmax_TGH                          ............................................................
V7CD35_Pvulgaris_TGH                     ............................................................
A0A1S3UTP2_Vradiata_TGH                  ............................................................
A0A1S2YR44_Carietinum_TGH                ............................................................
G7JT16_Mtruncatula_TGH                   ............................................................
A0A1U8FWC2_Cannuum_TGH                   ............................................................
V4TW69_Cclementina_TGH                   ............................................................
A0A1R3G1K6_Ccapsularis_TGH               ............................................................
A0A1R3H0J1_Colitorius_TGH                ............................................................
A0A0D2QRF0_Graimondii_TGH                ............................................................
A0A067JJA6_Jcurcas_TGH                   ............................................................
B9RE60_Rcommunis_TGH                     ............................................................
SapurV1A.0789s0080.1_SSpurpurea_TGH      ............................................................
A0A2I4E4A1_Jregia_TGH                    ............................................................
F6GTG8_Vvinifera_TGH                     ............................................................
W9S666_Mnotabilis_TGH                    ............................................................
A0A068TQE9_Ccanephora_TGH                ............................................................
A0A200Q3N1_Mcordata_TGH                  ............................................................
A0A1U8AEE6_Nnucifera_TGH                 ............................................................
A0A0A0LSK9_Csativus_TGH                  ............................................................
A0A059A5E4_Egrandis_TGH                  ............................................................
A0A218XZU2_Pgranatum_TGH                 ............................................................
A0A022RKT3_Eguttata_TGH                  ............................................................
A0A0J8B830_Bvulgaris_TGH                 ............................................................
Kalax.0309s0034.1_Klaxiflora_TGH         ............................................................
A0A124SAU9_Ccardunculus_TGH              ............................................................
W1PSA4_Atrichopoda_TGH                   ............................................................
A0A0K9NIC3_Zmarina_TGH                   ............................................................
A0A2C9VXU1_Mesculenta_TGH                DFGCPNLQEHDSDGKTNQVSKIEEESCEIVYNLDEDQEFDNIDFHNSDCSNLEKENLDND
A0A0U9HQK1_Knitens_TGH                   ............................................................
Sphfalx0151s0047.1_SSfallax_TGH          ............................................................
D8QX10_Smoellendorffii_TGH               ............................................................
E1ZI32_Cvariabilis_TGH                   ............................................................
C1FFR5_Mcommoda_TGH                      ............................................................
A0A086T442_Achrysogenum_TGH              ............................................................
K3V9N2_Fpseudograminearum_TGH            ............................................................
W7LTD5_Gmoniliformis_TGH                 ............................................................
G9NMW3_Hatroviridis_TGH                  ............................................................
A0A0G0A2Q9_Tharzianum_TGH                ............................................................
A0A1T3CLH2_Tguizhouense_TGH              ............................................................
A0A084QRJ1_Schlorohalonata_TGH           ............................................................
J4KPK4_Bbassiana_TGH                     ............................................................
A0A2H4SR46_Cmilitaris_TGH                ............................................................
A0A167VSL6_Cfumosorosea_TGH              ............................................................
A0A179G8E9_Pchlamydosporia_TGH           ............................................................
A0A0L0MXB1_Tophioglossoides_TGH          ............................................................
Q2GSN0_Cglobosum_TGH                     ............................................................
G2QES8_Mthermophila_TGH                  ............................................................
A0A175VUX4_Mmycetomatis_TGH              ............................................................
G2QRS6_Tterrestris_TGH                   ............................................................
B2ARI9_Panserina_TGH                     ............................................................
F7W019_Smacrospora_TGH                   ............................................................
A0A1J7J5C9_Cligniaria_TGH                ............................................................
A0A0G2IFE8_Dampelina_TGH                 ............................................................
A0A194W011_Vmali_TGH                     ............................................................
R8BKG4_Tminima_TGH                       ............................................................
G0SA63_Cthermophilum_TGH                 ............................................................
J3NFM2_Ggraminis_TGH                     ............................................................
A0A0C4DZ99_Mpoae_TGH                     ............................................................
L7IH22_Moryzae_TGH                       ............................................................
A0A1Y2WZC5_Daldiniasp._TGH               ............................................................
A0A1Y2V8J1_Hypoxylon                     ............................................................
A0A1Y2W1E2_Hypoxylon                     ............................................................
A0A1W2TFH3_Rnecatrix_TGH                 ............................................................
W3WZ77_Pfici_TGH                         ............................................................
A0A1Y2DPV9_Pvexata_TGH                   ............................................................
A0A136JHX1_Mbolleyi_TGH                  ............................................................
S3CR73_Glozoyensis_TGH                   ............................................................
K1WET1_Mbrunnea_TGH                      ............................................................
A0A218YYN9_Mcoronariae_TGH               ............................................................
A0A194XKL9_Pscopiformis_TGH              ............................................................
A0A0C3DJQ0_Omaius_TGH                    ............................................................
A7ECA3_Ssclerotiorum_TGH                 ............................................................
W9CY85_Sborealis_TGH                     ............................................................
A0A094E7L1_Pseudogymnoascus              ............................................................
A0A1B8CGM9_Pseudogymnoascus              ............................................................
A0A094H174_Pseudogymnoascus              ............................................................
Q0D178_Aterreus_TGH                      ............................................................
A0A1L9NNK2_Atubingensis_TGH              ............................................................
A1CQ12_Aclavatus_TGH                     ............................................................
A0A1L9RVU1_Awentii_TGH                   ............................................................
A0A1E3BNS4_Acristatus_TGH                ............................................................
I7ZRW9_Aoryzae_TGH                       ............................................................
A0A0F8UC36_Aochraceoroseus_TGH           ............................................................
Q5BE58_Enidulans_TGH                     ............................................................
A0A0A2KYD8_Pitalicum_TGH                 ............................................................
A0A1V6QJR5_Pantarcticum_TGH              ............................................................
A0A1Q5UD44_Psubrubescens_TGH             ............................................................
A0A1V6P0U0_Pdecumbens_TGH                ............................................................
A0A0F4YXS3_Remersonii_TGH                ............................................................
```

```
B8LVN1_Tstipitatus_TGH               ............................................................
A0A225B272_Tatroroseus_TGH           ............................................................
A0A0H1BJD0_Bsilverae_TGH             ............................................................
A0A1B7NTS8_Esp._TGH                  ............................................................
C0RYG0_Pbrasiliensis_TGH             ............................................................
C4JTX0_Ureesii_TGH                   ............................................................
R7Z059_Capollinis_TGH                ............................................................
A0A1S8BM68_Dseriata_TGH              ............................................................
K2RXL0_Mphaseolina_TGH               ............................................................
A0A165JKY3_Xheveae_TGH               ............................................................
A0A0G2GV44_Pchlamydospora_TGH        ............................................................
F0X9I8_Gclavigera_TGH                ............................................................
S3BTH5_Opiceae_TGH                   ............................................................
A0A0C2J2D4_Sbrasiliensis_TGH         ............................................................
A0A167VN43_Sinsectorum_TGH           ............................................................
F7W020_Smacrospora_TGH               ............................................................
A0A151N705_Amississippiensis_TGH     ............................................................
A0A286X9R8_Cporcellus_TGH            ............................................................
A0A1S3ARH5_Eeuropaeus_TGH            ............................................................
Q9BRR8_Hsapiens_TGH                  ............................................................
Q9DBM1_Mmusculus_TGH                 ............................................................
L5LZ46_Mdavidii_TGH                  ............................................................
S7PC93_Mbrandtii_TGH                 ............................................................
A0A1S3FZ16_Dordii_TGH                ............................................................
F6Z6Q3_Mdomestica_TGH                ............................................................
G3WTW2_Sharrisii_TGH                 ............................................................
Q21827_Celegans_TGH                  ............................................................
Q9VUA0_Dmelanogaster_TGH             ............................................................
```

	310
Aco012726.1_Acomosus_TGH	....REEFEWR
A0A199V6F8_Acomosus_TGH	....REEFEWR
M0TMB2_Macuminata_TGH	....REEFQWR
A0A2H3XE04_Pdactylifera_TGH	....REEFQWR
A0A0Q3H630_Bdistachyon_TGH	....REEFEWR
J3MEA3_Obrachyantha_TGH	....REEFEWR
B8B2G4_Osativa_TGH	....REEFEWR
K3XV17_Sitalica_TGH	....REQFEWR
Q8GXN9_Athaliana_TGH	....REEFQWR
Bol036258_Boleraceacapitata_TGH	....REEFQWR
V4MKB5_Esalsugineum_TGH	....REEFQWR
A0A087GCN0_Aalpina_TGH	....REEFQWR
A0A151SPT8_Ccajan_TGH	....REEFQWR
I1K185_Gmax_TGH	....REEFQWR
V7CD35_Pvulgaris_TGH	....REEFQWR
A0A1S3UTP2_Vradiata_TGH	....REEFQWR
A0A1S2YR44_Carietinum_TGH	....REEFQWR
G7JT16_Mtruncatula_TGH	....REEFQWR
A0A1U8FWC2_Cannuum_TGH	....REEFQWR
V4TW69_Cclementina_TGH	....REEFQWR
A0A1R3G1K6_Ccapsularis_TGH	....REEFQWR
A0A1R3H0J1_Colitorius_TGH	....REEFQWR
A0A0D2QRF0_Graimondii_TGH	....REEFQWR
A0A067JJA6_Jcurcas_TGH	....REEFQWR
B9RE60_Rcommunis_TGH	....REEFQWR
SapurV1A.0789s0080.1_SSpurpurea_TGH	....REEFQWR
A0A2I4E4A1_Jregia_TGH	....REECQWR
F6GTG8_Vvinifera_TGH	....REEFQWR
W9S666_Mnotabilis_TGH	....REEYQWR
A0A068TQE9_Ccanephora_TGH	....REEFQWR
A0A200Q3N1_Mcordata_TGH	....REEYQWR
A0A1U8AEE6_Nnucifera_TGH	....REEFQWR
A0A0A0LSK9_Csativus_TGH	....REEYQWR
A0A059A5E4_Egrandis_TGH	....REEFQWR
A0A218XZU2_Pgranatum_TGH	....REEFQWR
A0A022RKT3_Eguttata_TGH	....REEFQWR
A0A0J8B830_Bvulgaris_TGH	....REEFQWR
Kalax.0309s0034.1_Klaxiflora_TGH	....REEFHWR
A0A124SAU9_Ccardunculus_TGH	....REEFQWR
W1PSA4_Atrichopoda_TGH	....REEYQWR
A0A0K9NIC3_Zmarina_TGH	....REEFQWR
A0A2C9VXU1_Mesculenta_TGH	GLAKDEESSMDFGCPNLQ...................................REEFQWR
A0A0U9HQK1_Knitens_TGH	....REVEEWR
Sphfalx0151s0047.1_SSfallax_TGH	....RIENPWR
D8QX10_Smoellendorffii_TGH	....RTEEPWR
E1ZI32_Cvariabilis_TGH	....WA
C1FFR5_Mcommoda_TGH	....RVSRDWA
A0A086T442_Achrysogenum_TGH	....................................ADPAEEAAKMGMFGTM.RTVEDFY
K3V9N2_Fpseudograminearum_TGH	....................................TDPAEDAAKVGMYGKM.RTVDNFY
W7LTD5_Gmoniliformis_TGH	....................................ADPAEEAAKIGMYGQM.RTVEDFY
G9NMW3_Hatroviridis_TGH	....................................LDPAEEAAKMGMFGNL.RSVEDFY
A0A0G0A2Q9_Tharzianum_TGH	....................................SDPAEEAAKMGMFGKM.RSVEDFY
A0A1T3CLH2_Tguizhouense_TGH	....................................SDPAEEAAKMGMFGKM.RSVEDFY
A0A084QRJ1_Schlorohalonata_TGH	....................................VDPAEEAAKMGMFGPM.RKVEDFL
J4KPK4_Bbassiana_TGH	....................................TDAAEDAAKVGMYGKL.RSVRDFY
A0A2H4SR46_Cmilitaris_TGH	....................................TDPAEDAAKVGMYGKL.RSVRDFY
A0A167VSL6_Cfumosorosea_TGH	....................................TDPAEEAANLGMYGKL.RSVRDFY
A0A179G8E9_Pchlamydosporia_TGH	....................................ANPAEEAAKMGMFGHM.RSVEDFY
A0A0L0MXB1_Tophioglossoides_TGH	....................................ADEAEVAEKMGMFGEK.RSVQDFF
Q2GSN0_Cglobosum_TGH	....................................QDPAEEAAKLGMFGPM.RSVANFY
G2QES8_Mthermophila_TGH	....................................QDPSEEAAKLGMFGPM.RSVTEFY
A0A175VUX4_Mmycetomatis_TGH	....................................QDPAEEAAKLGMFGPM.RSVTDFY
G2QRS6_Tterrestris_TGH	....................................QDPAEEAAKLGMFGPM.RSVADFY
B2ARI9_Panserina_TGH	....................................QDPSEEAAKLGMFGPM.RSVTDFY
F7W019_Smacrospora_TGH	....................................EDPAEEAARLGMFGTM.RSTTDFY
A0A1J7J5C9_Cligniaria_TGH	....................................KDPAEEAAKLGMFGHM.RSVADFY
A0A0G2IFE8_Dampelina_TGH	....................................EDPAEAAAKMGMYGHM.RSTRDFY
A0A194W011_Vmali_TGH	....................................EDPAEQAAKMGMFGHM.RSSKDFY
R8BKG4_Tminima_TGH	....................................SDPAEEAAKMGMYGEM.RSVKDFY
G0SA63_Cthermophilum_TGH	....................................QDPTEEAAKLGMFGPM.RTVTEFY
J3NFM2_Ggraminis_TGH	....................................LDPAEEAAKMGMFGPL.RSSHDFY
A0A0C4DZ99_Mpoae_TGH	....................................SDPAEEAARMGMFGPM.RSSHDFY
L7IH22_Moryzae_TGH	....................................TDPAEEAAKLGMFGPM.RSVKDFY
A0A1Y2WZC5_Daldiniasp._TGH	....................................VDPAEQAAKLGMYGAM.RSVGDFY
A0A1Y2V8J1_Hypoxylon	....................................LDPAEEAAKMGMYGSM.RTVTDFY
A0A1Y2W1E2_Hypoxylon	....................................VDPAEEAARLGMYGAMTRSVTDFY
A0A1W2TFH3_Rnecatrix_TGH	....................................KDPAEEAASLGMYGAM.RSVTDFY
W3WZ77_Pfici_TGH	....................................TDPAEEAAKMGMYGTM.RSTTDFY
A0A1Y2DPV9_Pvexata_TGH	....................................ADPSEEAARLGMYGAM.RSVTDFY
A0A136JHX1_Mbolleyi_TGH	....................................ADPAEEAAKMGMYGHL.RTSADFY
S3CR73_Glozoyensis_TGH	....................................EDPAEQAAKLGMYGPM.RSSKDWY
K1WET1_Mbrunnea_TGH	....................................VDPAEQAAKLGMYGPM.RSTADWY
A0A218YYN9_Mcoronariae_TGH	....................................ANPAEQAAKLGMYGPL.RSSADWY
A0A194XKL9_Pscopiformis_TGH	....................................EDPAEQAAKLGMYGPM.RSTKDWY
A0A0C3DJQ0_Omaius_TGH	....................................EDPAEQAAKVSMYGPL.RSTSSWY
A7ECA3_Ssclerotiorum_TGH	....................................EDPAEQAAKMGMYGPM.REKKDWF
W9CY85_Sborealis_TGH	....................................EDPAEQAAKLGMYGPM.RERKDWF
A0A094E7L1_Pseudogymnoascus	....................................EDPAEAAAKAGMFGRL.RSEEDFY
A0A1B8CGM9_Pseudogymnoascus	....................................EDPAEAAAKAGMFGRL.RSEENFY
A0A094H174_Pseudogymnoascus	....................................EDPAEAAAKAGMFGRL.RSEEDFY
Q0D178_Aterreus_TGH	....................................ADPAEEAAKIGMFGPM.RSHHTFY
A0A1L9NNK2_Atubingensis_TGH	....................................EDPAVEAAKIGMFGPM.RSSISFY
A1CQ12_Aclavatus_TGH	....................................EDPAMAAAKIGMFGPM.RSVSSFY
A0A1L9RVU1_Awentii_TGH	....................................EDPAVAAAKIGMFGPM.RSTISFY
A0A1E3BNS4_Acristatus_TGH	....................................EDPAVVAAKIGMFGPM.RSTISFY
I7ZRW9_Aoryzae_TGH	....................................EDPAVAAAKIGMFGPM.RSNISFY
A0A0F8UC36_Aochraceoroseus_TGH	....................................EDPAVAAAKIGMFGPM.RTTISFY
Q5BE58_Enidulans_TGH	....................................QDPVIEAAKIGMFGPM.RTVISFY
A0A0A2KYD8_Pitalicum_TGH	....................................DDPAVAAAKIGMFGPM.RSTISFY
A0A1V6QJR5_Pantarcticum_TGH	....................................DTPAVAAAKIGMFGQM.RSTISFY
A0A1Q5UD44_Psubrubescens_TGH	....................................DSPAVAAAKIGMFGQM.RSSISFY
A0A1V6P0U0_Pdecumbens_TGH	....................................DPPAVAAAKIGMFGQM.RSVIPFY
A0A0F4YXS3_Remersonii_TGH	....................................QDPAEEAAKIGMFGPM.RSTLSFY

```
B8LVN1_Tstipitatus_TGH                ....................................EDPAEEAAKIGMFGPL.RSTVSFY
A0A225B272_Tatroroseus_TGH            ....................................ADPAEEAANIGMFGPM.RSTLTFY
A0A0H1BJD0_Bsilverae_TGH              ...........................QQGS.....QDPAEAAAKIGMYGPL.RSSIPFS
A0A1B7NTS8_Esp._TGH                   ..............................T.....QDPAEAAAKIGMYGPL.RSSVPFS
C0RYG0_Pbrasiliensis_TGH              ....................................KDPAEEAAKMGMYGPL.RSSLPFA
C4JTX0_Ureesii_TGH                    ....................................EDPAEAAAKFGMYGPM.RLTLRFS
R7Z059_Capollinis_TGH                 ....................................TDPAEEAAKIGMYGPM.RSVLQFF
A0A1S8BM68_Dseriata_TGH               ....................................ADPAEEAAKLGMYGPM.RQILQFY
K2RXL0_Mphaseolina_TGH                ....................................ADPAEEAAKLGMYGPM.RQVLPFY
A0A165JKY3_Xheveae_TGH                ....................................EDPAESAAKMGMFGPL.RSVQQFF
A0A0G2GV44_Pchlamydospora_TGH         ....................................EDPADQAAKLGMFGPM.RTITNFY
F0X9I8_Gclavigera_TGH                 ....................................QDPAEEAASMGMYGPL.RSVQEFF
S3BTH5_Opiceae_TGH                    ....................................RDPAEEAAKMGMYGPL.RTVTDFF
A0A0C2J2D4_Sbrasiliensis_TGH          ....................................QDPAEEAAKMGMYGPM.RTVGEFF
A0A167VN43_Sinsectorum_TGH            ..................TAGTSNNDG.........QDPAEEAAKMGMYGPL.RSVQDFF
F7W020_Smacrospora_TGH                ....................................SDP.....................
A0A151N705_Amississippiensis_TGH      ....................................IDDKESAVKMKMFGKL.RDKFEWH
A0A286X9R8_Cporcellus_TGH             ....................................VTDKQSAVKMKMFGKL.RDVFEWH
A0A1S3ARH5_Eeuropaeus_TGH             ....................................VNDKQSAVKMKMFGKL.RDTFEWH
Q9BRR8_Hsapiens_TGH                   ....................................VGDKQSAVKMKMFGKL.RDTFEWH
Q9DBM1_Mmusculus_TGH                  ....................................VSDKQSAVKMKMFGKL.RDTFEWH
L5LZ46_Mdavidii_TGH                   ...............................RVLRSVNDKQSAVKMKMFGKL.RDTFEWH
S7PC93_Mbrandtii_TGH                  ....................................VNDKQSAVKMKMFGKL.RDTFEWH
A0A1S3FZ16_Dordii_TGH                 ....................................VNDKQSAVKMKMFGKL.RDTFEWH
F6Z6Q3_Mdomestica_TGH                 ....................................VDDKLSAVKMKMFGKL.RDKFEWH
G3WTW2_Sharrisii_TGH                  ....................................VDDKLSAVKMKMFGKL.RDKFEWH
Q21827_Celegans_TGH                   ......................................DKLAAVKMEMFGEK.RQSFDWY
Q9VUA0_Dmelanogaster_TGH              ......................................PEERKIVM........RTKTMWK
```

```
                                          320
                                            .
Aco012726.1_Acomosus_TGH                  PSPILCKRFDLIDPFM............................................
A0A199V6F8_Acomosus_TGH                   PSPILCKRFDLIDPFM............................................
M0TMB2_Macuminata_TGH                     PLPILCKRFDIIDPFM............................................
A0A2H3XE04_Pdactylifera_TGH               PTPILCKRFDIVDPFM............................................
A0A0Q3H630_Bdistachyon_TGH                PSPILCKRFDIVDPFM............................................
J3MEA3_Obrachyantha_TGH                   PSPILCKRFDIVDPFM............................................
B8B2G4_Osativa_TGH                        PSPILCKRFDIVDPFM............................................
K3XV17_Sitalica_TGH                       PLPILCKRFDIVDPFM............................................
Q8GXN9_Athaliana_TGH                      PSPLLCKRFDLPDPFM............................................
Bol036258_Boleraceacapitata_TGH           PAPLLCKRFDLPDPFM............................................
V4MKB5_Esalsugineum_TGH                   PAPLLCKRFDLPDPFM............................................
A0A087GCN0_Aalpina_TGH                    PASLLCKRFDLPDPFM............................................
A0A151SPT8_Ccajan_TGH                     PSPLLCKRFDLIDPYM............................................
I1K185_Gmax_TGH                           PSPLLCKRFDLIDPYM............................................
V7CD35_Pvulgaris_TGH                      PSPLLCKRFDLIDPYM............................................
A0A1S3UTP2_Vradiata_TGH                   PSPLLCKRFDLIDPYM............................................
A0A1S2YR44_Carietinum_TGH                 PSPLLCKRFDLIDPYM............................................
G7JT16_Mtruncatula_TGH                    PSPLLCKRFDLVDPYM............................................
A0A1U8FWC2_Cannuum_TGH                    PSPILCKRFDLIDPYM............................................
V4TW69_Cclementina_TGH                    PSPILCKRFDLIDPYI............................................
A0A1R3G1K6_Ccapsularis_TGH                PMPILCKRFDLIDPYM............................................
A0A1R3H0J1_Colitorius_TGH                 PMPILCKRFDLIDPYM............................................
A0A0D2QRF0_Graimondii_TGH                 PLPVLCKRFDLIDPFM............................................
A0A067JJA6_Jcurcas_TGH                    PLPVLCKRFDLIDPYM............................................
B9RE60_Rcommunis_TGH                      PLPILCKRFDLIDPYM............................................
SapurV1A.0789s0080.1_SSpurpurea_TGH       PSGILCKRFDLIDPYM............................................
A0A2I4E4A1_Jregia_TGH                     PSPVLCKRFDLIDPYM............................................
F6GTG8_Vvinifera_TGH                      PSPILCKRFDIIDPFM............................................
W9S666_Mnotabilis_TGH                     PSPILCKRFDLIDPYM............................................
A0A068TQE9_Ccanephora_TGH                 PSPILCKRFDLIDPYM............................................
A0A200Q3N1_Mcordata_TGH                   PSSLLCKRFDIIDPYM............................................
A0A1U8AEE6_Nnucifera_TGH                  PSPILCKRFDIIDPYM............................................
A0A0A0LSK9_Csativus_TGH                   PAPILCKRFDLIDPYM............................................
A0A059A5E4_Egrandis_TGH                   PSPILCKRFDLIDPYM............................................
A0A218XZU2_Pgranatum_TGH                  PSPILCKRFDLIDPYM............................................
A0A022RKT3_Eguttata_TGH                   PAPILCKRFDLTDPYM............................................
A0A0J8B830_Bvulgaris_TGH                  PAPLLCKRFDIVDPYM............................................
Kalax.0309s0034.1_Klaxiflora_TGH          PSPILCKRFDLTDPYI............................................
A0A124SAU9_Ccardunculus_TGH               PASILCKRFDLIDPYM............................................
W1PSA4_Atrichopoda_TGH                    PAPILCKRFDLLDPFA............................................
A0A0K9NIC3_Zmarina_TGH                    PAPILCKRFDITDPFM............................................
A0A2C9VXU1_Mesculenta_TGH                 PLPVLCKRFDLIDPYM............................................
A0A0U9HQK1_Knitens_TGH                    PAPLLCRRFNILDPYE............................................
Sphfalx0151s0047.1_SSfallax_TGH           PSSMLCKRFNLLDPYA............................................
D8QX10_Smoellendorffii_TGH                PLPLLCKRFDVLDPYT............................................
E1ZI32_Cvariabilis_TGH                    ................DAA.........................................
C1FFR5_Mcommoda_TGH                       PESLLCKRFDVRDPFE...SIV.....VLE......................PPPQTPV.
A0A086T442_Achrysogenum_TGH               PTRLLCKRFNVK................................................
K3V9N2_Fpseudograminearum_TGH             PTRLLCKRFNVR................................................
W7LTD5_Gmoniliformis_TGH                  PTRLLCKRFNVR................................................
G9NMW3_Hatroviridis_TGH                   PTRLLCKRFNVK................................................
A0A0G0A2Q9_Tharzianum_TGH                 PTRLLCKRFNVK................................................
A0A1T3CLH2_Tguizhouense_TGH               PTRLLCKRFNVK................................................
A0A084QRJ1_Schlorohalonata_TGH            PTRLLCKRFNVK................................................
J4KPK4_Bbassiana_TGH                      PSRLLCKRFNVR................................................
A0A2H4SR46_Cmilitaris_TGH                 PSRLLCKRFNVR................................................
A0A167VSL6_Cfumosorosea_TGH               PSRLLCKRFNVR................................................
A0A179G8E9_Pchlamydosporia_TGH            PTRLLCKRFNVR................................................
A0A0L0MXB1_Tophioglossoides_TGH           PTRLLCKRFNVK................................................
Q2GSN0_Cglobosum_TGH                      PTRLLCKRFNVR................................................
G2QES8_Mthermophila_TGH                   PARLLCKRFNVK................................................
A0A175VUX4_Mmycetomatis_TGH               PTRLLCKRFNVK................................................
G2QRS6_Tterrestris_TGH                    PTRLLCKRFNVK................................................
B2ARI9_Panserina_TGH                      PTRLLCKRFNVR................................................
F7W019_Smacrospora_TGH                    PTRLLCKRFGVK................................................
A0A1J7J5C9_Cligniaria_TGH                 PSRLLCKRFGVK................................................
A0A0G2IFE8_Dampelina_TGH                  PARLLCKRFNVK................................................
A0A194W011_Vmali_TGH                      PSRLLCKRFNVK................................................
R8BKG4_Tminima_TGH                        PTRLLCKRFNVK................................................
G0SA63_Cthermophilum_TGH                  PSRLLCKRFGVN................................................
J3NFM2_Ggraminis_TGH                      PARLLCKRFNVK................................................
A0A0C4DZ99_Mpoae_TGH                      PARLLCKRFNVK................................................
L7IH22_Moryzae_TGH                        PNRLLCKRFNVK................................................
A0A1Y2WZC5_Daldiniasp._TGH                PTRLLCKRFNVK................................................
A0A1Y2V8J1_Hypoxylon                      PTRLLCKRFNVR................................................
A0A1Y2W1E2_Hypoxylon                      PTRLLCKRFNVR................................................
A0A1W2TFH3_Rnecatrix_TGH                  PTRLLCKRFNVP................................................
W3WZ77_Pfici_TGH                          PTRLLCKRFNVP................................................
A0A1Y2DPV9_Pvexata_TGH                    PTRLLCKRFNVR.....................................DM.........
A0A136JHX1_Mbolleyi_TGH                   PTRLLCKRFNVK........................KEPDWTSLASLPS...........
S3CR73_Glozoyensis_TGH                    PTRLLCKRFNVQ................................................
K1WET1_Mbrunnea_TGH                       PSRLLCKRFNVK................................................
A0A218YYN9_Mcoronariae_TGH                PSRLLCKRFNIQ................................................
A0A194XKL9_Pscopiformis_TGH               PTRLLCKRFNVK................................................
A0A0C3DJQ0_Omaius_TGH                     PTRLLCKRFNIK................................................
A7ECA3_Ssclerotiorum_TGH                  PTRLLCKRFNVQ................................................
W9CY85_Sborealis_TGH                      PSRLLCKRFNVQ................................................
A0A094E7L1_Pseudogymnoascus               PTRLLCKRFGVR................................................
A0A1B8CGM9_Pseudogymnoascus               PTRLLCKRFGVR................................................
A0A094H174_Pseudogymnoascus               PTRLLCKRFGVR................................................
Q0D178_Aterreus_TGH                       PTRLLCKRFNVK................................................
A0A1L9NNK2_Atubingensis_TGH               PTRLLCKRFNVR................................................
A1CQ12_Aclavatus_TGH                      PARLLCKRLNVK................................................
A0A1L9RVU1_Awentii_TGH                    PARLLCKRLNVK................................................
A0A1E3BNS4_Acristatus_TGH                 PGRLLCKRFNVK................................................
I7ZRW9_Aoryzae_TGH                        PTRLLCKRLNVK................................................
A0A0F8UC36_Aochraceoroseus_TGH            PTRLLCKRFNIK................................................
Q5BE58_Enidulans_TGH                      PTRLLCKRFNVK................................................
A0A0A2KYD8_Pitalicum_TGH                  PSRLLCKRFNVK................................................
A0A1V6QJR5_Pantarcticum_TGH               PSRLLCKRFNIK................................................
A0A1Q5UD44_Psubrubescens_TGH              PSRLLCKRFNVK................................................
A0A1V6P0U0_Pdecumbens_TGH                 PARLLCKRFNIK................................................
A0A0F4YXS3_Remersonii_TGH                 PSRLLCKRFNVK................................................
```

```
B8LVN1_Tstipitatus_TGH                PTRLLCKRFNIK................................................
A0A225B272_Tatroroseus_TGH            PTRLLCKRFNIR................................................
A0A0H1BJD0_Bsilverae_TGH              PSRLLCKRFNVR................................................
A0A1B7NTS8_Esp._TGH                   PFRLLCKRFNVR................................................
C0RYG0_Pbrasiliensis_TGH              PFRLLCKRFNVR...............................................T
C4JTX0_Ureesii_TGH                    PTRLLYKRFNLRF...............................................
R7Z059_Capollinis_TGH                 PTRLVCKRFNVK................................................
A0A1S8BM68_Dseriata_TGH               PTRLVCKRFNVK................................................
K2RXL0_Mphaseolina_TGH                PSRLVCKRFNVK................................................
A0A165JKY3_Xheveae_TGH                PTRLLCKRFNIR................................................
A0A0G2GV44_Pchlamydospora_TGH         PTRLLCKRFNVK................................................
F0X9I8_Gclavigera_TGH                 PTRLLCKRLGVK................................................
S3BTH5_Opiceae_TGH                    PTRLLCKRMGVQ................................................
A0A0C2J2D4_Sbrasiliensis_TGH          PTRLLCKRMGVK.......................................T........
A0A167VN43_Sinsectorum_TGH            PTRLLCKRLGVP................................................
F7W020_Smacrospora_TGH                ........FVVH................................................
A0A151N705_Amississippiensis_TGH      PEKLLCKRFNVPDPYS...............IFNFL........................
A0A286X9R8_Cporcellus_TGH             PDKLLCKRFNVPDPYP...............VFNFL........................
A0A1S3ARH5_Eeuropaeus_TGH             PDKLLCKRFNVPDPYP...............VFNFL........................
Q9BRR8_Hsapiens_TGH                   PDKLLCKRFNVPDPYP...............VFNFL........................
Q9DBM1_Mmusculus_TGH                  PDKLLCKRFNVPDPYP...............VFNFL........................
L5LZ46_Mdavidii_TGH                   PDKLLCKRFNVPDPYP...............VFNFL........................
S7PC93_Mbrandtii_TGH                  PDKLLCKRFNVPDPYP...............VFNFL........................
A0A1S3FZ16_Dordii_TGH                 PDKLLCKRFNVPDPYP...............VFNFL........................
F6Z6Q3_Mdomestica_TGH                 PEKLLCKRFNIPDPYP...............VFSFL........................
G3WTW2_Sharrisii_TGH                  PEKLLCKRFNIPDPYP...............VFNFL........................
Q21827_Celegans_TGH                   PDNLLAKRFNVPHPYP......TDWKRK...VSATL........................
Q9VUA0_Dmelanogaster_TGH              PTALLCKRYNIAEPF.............................................
```

```
330
Aco012726.1_Acomosus_TGH             .....................LYKAIF.................................
A0A199V6F8_Acomosus_TGH              .....................LYKAIF.................................
M0TMB2_Macuminata_TGH                .....................LYKAIF.................................
A0A2H3XE04_Pdactylifera_TGH          .....................LYKAIF.................................
A0A0Q3H630_Bdistachyon_TGH           .....................LYKAIF.................................
J3MEA3_Obrachyantha_TGH              .....................LYKAIF.................................
B8B2G4_Osativa_TGH                   .....................LYKAIF.................................
K3XV17_Sitalica_TGH                  .....................LYKAIF.................................
Q8GXN9_Athaliana_TGH                 .....................LYKAIF.................................
Bol036258_Boleraceacapitata_TGH      .....................LYKAIF.................................
V4MKB5_Esalsugineum_TGH              .....................LYKAIF............................D....
A0A087GCN0_Aalpina_TGH               .....................LYKAIF.................................
A0A151SPT8_Ccajan_TGH                .....................LYKAIF.................................
I1K185_Gmax_TGH                      .....................LYKAIF.................................
V7CD35_Pvulgaris_TGH                 .....................LYKAIF.................................
A0A1S3UTP2_Vradiata_TGH              .....................LYKAIF.................................
A0A1S2YR44_Carietinum_TGH            .....................LYKAIF.................................
G7JT16_Mtruncatula_TGH               .....................LYKAIF.................................
A0A1U8FWC2_Cannuum_TGH               .....................LYKAIF.................................
V4TW69_Cclementina_TGH               .....................LYKAIF.................................
A0A1R3G1K6_Ccapsularis_TGH           .....................LYKAIF.................................
A0A1R3H0J1_Colitorius_TGH            .....................LYKAIF.................................
A0A0D2QRF0_Graimondii_TGH            .....................LYKAIF.................................
A0A067JJA6_Jcurcas_TGH               .....................LYKAIF.................................
B9RE60_Rcommunis_TGH                 .....................LYKAIF.................................
SapurV1A.0789s0080.1_SSpurpurea_TGH  .....................LYKAIF.................................
A0A2I4E4A1_Jregia_TGH                .....................LYKAIF.................................
F6GTG8_Vvinifera_TGH                 .....................LYKAIF.................................
W9S666_Mnotabilis_TGH                .....................LYKAIF.................................
A0A068TQE9_Ccanephora_TGH            .....................LYKAIF.................................
A0A200Q3N1_Mcordata_TGH              .....................LYKAIF.................................
A0A1U8AEE6_Nnucifera_TGH             .....................LYKAIF.................................
A0A0A0LSK9_Csativus_TGH              .....................LYKAIF.................................
A0A059A5E4_Egrandis_TGH              .....................LYKAIF.................................
A0A218XZU2_Pgranatum_TGH             .....................LYKAIF.................................
A0A022RKT3_Eguttata_TGH              .....................LYKAIF.................................
A0A0J8B830_Bvulgaris_TGH             .....................LYKAIF.................................
Kalax.0309s0034.1_Klaxiflora_TGH     .....................LYKAIF.................................
A0A124SAU9_Ccardunculus_TGH          ........................AIF.................................
W1PSA4_Atrichopoda_TGH               .....................LYKAIF.................................
A0A0K9NIC3_Zmarina_TGH               .....................LYKAIF.................................
A0A2C9VXU1_Mesculenta_TGH            .....................LYKAIF.................................
A0A0U9HQK1_Knitens_TGH               .....................LFKAIF.................................
Sphfalx0151s0047.1_SSfallax_TGH      .....................LYKAIF.................................
D8QX10_Smoellendorffii_TGH           .....................LYKAIF.................................
E1ZI32_Cvariabilis_TGH               .....................LFKAIF.................................
C1FFR5_Mcommoda_TGH                  ................GSFLDFFKAIF.................................
A0A086T442_Achrysogenum_TGH          .....................VLRAIF.................................
K3V9N2_Fpseudograminearum_TGH        .....................VLRAIF.................................
W7LTD5_Gmoniliformis_TGH             .....................VLRAIF.................................
G9NMW3_Hatroviridis_TGH              .....................VLRAIF.................................
A0A0G0A2Q9_Tharzianum_TGH            ..........................H.................................
A0A1T3CLH2_Tguizhouense_TGH          .....................VLRAIF.................................
A0A084QRJ1_Schlorohalonata_TGH       .....................VLRAIF.................................
J4KPK4_Bbassiana_TGH                 .....................LLKAVF.................................
A0A2H4SR46_Cmilitaris_TGH            .....................LLKAES.................................
A0A167VSL6_Cfumosorosea_TGH          .....................LLKAVF.................................
A0A179G8E9_Pchlamydosporia_TGH       .....................VLKAIF.................................
A0A0L0MXB1_Tophioglossoides_TGH      .....................ALRAVF.................................
Q2GSN0_Cglobosum_TGH                 .....................VFKAIF.................................
G2QES8_Mthermophila_TGH              .....................VFRAIF.................................
A0A175VUX4_Mmycetomatis_TGH          .....................VFKAIF.................................
G2QRS6_Tterrestris_TGH               .....................VFKAIF.................................
B2ARI9_Panserina_TGH                 .....................VFKAIF.................................
F7W019_Smacrospora_TGH               .....................VFKAIF.................................
A0A1J7J5C9_Cligniaria_TGH            .....................VFKAIF.................................
A0A0G2IFE8_Dampelina_TGH             .....................VYKAIF.................................
A0A194W011_Vmali_TGH                 .....A...............VFKAIF.................................
R8BKG4_Tminima_TGH                   .....................VFKAIF.................................
G0SA63_Cthermophilum_TGH             .....................VFKAVF.................................
J3NFM2_Ggraminis_TGH                 .....................VFKAVF.................................
A0A0C4DZ99_Mpoae_TGH                 .....................VFKAVF.................................
L7IH22_Moryzae_TGH                   .....................VFKAIFAALPIKAFDKPVPLPLDARHSSANSSIV.....
A0A1Y2WZC5_Daldiniasp._TGH           .....................VLRAIF.................................
A0A1Y2V8J1_Hypoxylon                 .....................VLKAIF.................................
A0A1Y2W1E2_Hypoxylon                 .....................VLKAIF.................................
A0A1W2TFH3_Rnecatrix_TGH             .....................VLKAIF.................................
W3WZ77_Pfici_TGH                     .....................VLKAIF.................................
A0A1Y2DPV9_Pvexata_TGH               .....................VLKAIF.................................
A0A136JHX1_Mbolleyi_TGH              .....................VLRAIF.................................
S3CR73_Glozoyensis_TGH               .....................VFKAIF.................................
K1WET1_Mbrunnea_TGH                  .....................VFQAIF.................................
A0A218YYN9_Mcoronariae_TGH           .....................VFKAIF.................................
A0A194XKL9_Pscopiformis_TGH          .....................VFKAIF.................................
A0A0C3DJQ0_Omaius_TGH                .....................VFKAIF.................................
A7ECA3_Ssclerotiorum_TGH             ......DDNFDL...D.....VFRAVF.................................
W9CY85_Sborealis_TGH                 ......DIDVDLNEN......VFKAIF.................................
A0A094E7L1_Pseudogymnoascus          .....................VFKSIF.................................
A0A1B8CGM9_Pseudogymnoascus          .....................VFKSIF.................................
A0A094H174_Pseudogymnoascus          .....................VFKSIF.............................GVVQ
Q0D178_Aterreus_TGH                  .....................VFKAIF.................................
A0A1L9NNK2_Atubingensis_TGH          .....................VFKAIF.................................
A1CQ12_Aclavatus_TGH                 .....................VFKAIF.................................
A0A1L9RVU1_Awentii_TGH               .....................VFKAIF.................................
A0A1E3BNS4_Acristatus_TGH            .....................VFKAIF.................................
I7ZRW9_Aoryzae_TGH                   .....................IFKAIF.................................
A0A0F8UC36_Aochraceoroseus_TGH       .....................VFKAIF.................................
Q5BE58_Enidulans_TGH                 .....................VFKAIF.................................
A0A0A2KYD8_Pitalicum_TGH             .....................VFKAIF.................................
A0A1V6QJR5_Pantarcticum_TGH          .....................VFKAIF.................................
A0A1Q5UD44_Psubrubescens_TGH         .....................VFKAIF.................................
A0A1V6P0U0_Pdecumbens_TGH            .....................VFKAIF.................................
A0A0F4YXS3_Remersonii_TGH            .....................VFKAIF.................................
```

```
B8LVN1_Tstipitatus_TGH               .....................VFKAIF......................................
A0A225B272_Tatroroseus_TGH           .....................VFKAIF......................................
A0A0H1BJD0_Bsilverae_TGH             ...GQ................VFKAIF......................................
A0A1B7NTS8_Esp._TGH                  .....................VFKAIF......................................
C0RYG0_Pbrasiliensis_TGH             GL...................VFKAIF......................................
C4JTX0_Ureesii_TGH                   .....................VFKAIF......................................
R7Z059_Capollinis_TGH                .....................VFRAIF......................................
A0A1S8BM68_Dseriata_TGH              .....................VFKAIF......................................
K2RXL0_Mphaseolina_TGH               .....................VFKAIF......................................
A0A165JKY3_Xheveae_TGH               .....................MFKAIF......................................
A0A0G2GV44_Pchlamydospora_TGH        .....................VFKAIF......................................
F0X9I8_Gclavigera_TGH                .....................VFRAV.......................................
S3BTH5_Opiceae_TGH                   .....................VFRAV.......................................
A0A0C2J2D4_Sbrasiliensis_TGH         .....................VFRAK.......................................
A0A167VN43_Sinsectorum_TGH           .....................VFRAVF......................................
F7W020_Smacrospora_TGH               .................................................................
A0A151N705_Amississippiensis_TGH     .....................LFKAVF......................................
A0A286X9R8_Cporcellus_TGH            ..G..................LFKAIF......................................
A0A1S3ARH5_Eeuropaeus_TGH            .....................LFKAIF......................................
Q9BRR8_Hsapiens_TGH                  .....................LFRAIF......................................
Q9DBM1_Mmusculus_TGH                 .....................LFKAIF......................................
L5LZ46_Mdavidii_TGH                  .....................LFKAIF......................................
S7PC93_Mbrandtii_TGH                 .....................LFKAIF......................................
A0A1S3FZ16_Dordii_TGH                .....................LFRAIF......................................
F6Z6Q3_Mdomestica_TGH                .....................LFKAIF......................................
G3WTW2_Sharrisii_TGH                 .....................LFKAIF......................................
Q21827_Celegans_TGH                  .....................FFDFIF......................................
Q9VUA0_Dmelanogaster_TGH             .....................LFKSIF......................................
```

```
Aco012726.1_Acomosus_TGH                ............................................................
A0A199V6F8_Acomosus_TGH                 ............................................................
M0TMB2_Macuminata_TGH                   ............................................................
A0A2H3XE04_Pdactylifera_TGH             ............................................................
A0A0Q3H630_Bdistachyon_TGH              ............................................................
J3MEA3_Obrachyantha_TGH                 ............................................................
B8B2G4_Osativa_TGH                      ............................................................
K3XV17_Sitalica_TGH                     ............................................................
Q8GXN9_Athaliana_TGH                    ............................................................
Bol036258_Boleraceacapitata_TGH         ..................................................PEG.......
V4MKB5_Esalsugineum_TGH                 ..................................................PEG.......
A0A087GCN0_Aalpina_TGH                  ..................................................PEV.......
A0A151SPT8_Ccajan_TGH                   ............................................................
I1K185_Gmax_TGH                         ............................................................
V7CD35_Pvulgaris_TGH                    ............................................................
A0A1S3UTP2_Vradiata_TGH                 ............................................................
A0A1S2YR44_Carietinum_TGH               ............................................................
G7JT16_Mtruncatula_TGH                  ............................................................
A0A1U8FWC2_Cannuum_TGH                  ............................................................
V4TW69_Cclementina_TGH                  ............................................................
A0A1R3G1K6_Ccapsularis_TGH              ............................................................
A0A1R3H0J1_Colitorius_TGH               ............................................................
A0A0D2QRF0_Graimondii_TGH               ............................................................
A0A067JJA6_Jcurcas_TGH                  ............................................................
B9RE60_Rcommunis_TGH                    ............................................................
SapurV1A.0789s0080.1_SSpurpurea_TGH     ............................................................
A0A2I4E4A1_Jregia_TGH                   ............................................................
F6GTG8_Vvinifera_TGH                    ............................................................
W9S666_Mnotabilis_TGH                   ............................................................
A0A068TQE9_Ccanephora_TGH               ............................................................
A0A200Q3N1_Mcordata_TGH                 ............................................................
A0A1U8AEE6_Nnucifera_TGH                ............................................................
A0A0A0LSK9_Csativus_TGH                 ............................................................
A0A059A5E4_Egrandis_TGH                 ............................................................
A0A218XZU2_Pgranatum_TGH                ............................................................
A0A022RKT3_Eguttata_TGH                 ............................................................
A0A0J8B830_Bvulgaris_TGH                ............................................................
Kalax.0309s0034.1_Klaxiflora_TGH        ............................................................
A0A124SAU9_Ccardunculus_TGH             ............................................................
W1PSA4_Atrichopoda_TGH                  ............................................................
A0A0K9NIC3_Zmarina_TGH                  ............................................................
A0A2C9VXU1_Mesculenta_TGH               ............................................................
A0A0U9HQK1_Knitens_TGH                  ............................................................
Sphfalx0151s0047.1_SSfallax_TGH         ............................................................
D8QX10_Smoellendorffii_TGH              ............................................................
E1ZI32_Cvariabilis_TGH                  ............................................................
C1FFR5_Mcommoda_TGH                     ................................................GR..........
A0A086T442_Achrysogenum_TGH             ............................................................
K3V9N2_Fpseudograminearum_TGH           ............................................................
W7LTD5_Gmoniliformis_TGH                ............................................................
G9NMW3_Hatroviridis_TGH                 ............................................................
A0A0G0A2Q9_Tharzianum_TGH               ............................................................
A0A1T3CLH2_Tguizhouense_TGH             ............................................................
A0A084QRJ1_Schlorohalonata_TGH          ............................................................
J4KPK4_Bbassiana_TGH                    ............................................................
A0A2H4SR46_Cmilitaris_TGH               ............................................................
A0A167VSL6_Cfumosorosea_TGH             ............................................................
A0A179G8E9_Pchlamydosporia_TGH          ............................................................
A0A0L0MXB1_Tophioglossoides_TGH         ............................................................
Q2GSN0_Cglobosum_TGH                    ............................................................
G2QES8_Mthermophila_TGH                 ............................................................
A0A175VUX4_Mmycetomatis_TGH             ............................................................
G2QRS6_Tterrestris_TGH                  ............................................................
B2ARI9_Panserina_TGH                    ............................................................
F7W019_Smacrospora_TGH                  ............................................................
A0A1J7J5C9_Cligniaria_TGH               ............................................................
A0A0G2IFE8_Dampelina_TGH                ............................................................
A0A194W011_Vmali_TGH                    ............................................................
R8BKG4_Tminima_TGH                      ............................................................
G0SA63_Cthermophilum_TGH                ............................................................
J3NFM2_Ggraminis_TGH                    ............................................................
A0A0C4DZ99_Mpoae_TGH                    ............................................................
L7IH22_Moryzae_TGH                      ............................................................
A0A1Y2WZC5_Daldiniasp._TGH              ............................................................
A0A1Y2V8J1_Hypoxylon                    ............................................................
A0A1Y2W1E2_Hypoxylon                    ............................................................
A0A1W2TFH3_Rnecatrix_TGH                ............................................................
W3WZ77_Pfici_TGH                        ............................................................
A0A1Y2DPV9_Pvexata_TGH                  ............................................................
A0A136JHX1_Mbolleyi_TGH                 ............................................................
S3CR73_Glozoyensis_TGH                  ............................................................
K1WET1_Mbrunnea_TGH                     ............................................................
A0A218YYN9_Mcoronariae_TGH              ............................................................
A0A194XKL9_Pscopiformis_TGH             ............................................................
A0A0C3DJQ0_Omaius_TGH                   ............................................................
A7ECA3_Ssclerotiorum_TGH                ............................................................
W9CY85_Sborealis_TGH                    ............................................................
A0A094E7L1_Pseudogymnoascus             ............................................................
A0A1B8CGM9_Pseudogymnoascus             ............................................................
A0A094H174_Pseudogymnoascus             ............................................................
Q0D178_Aterreus_TGH                     ............................................................
A0A1L9NNK2_Atubingensis_TGH             QCDTAGVIGWQYQLGS...................G...........L............
A1CQ12_Aclavatus_TGH                    ............................................................
A0A1L9RVU1_Awentii_TGH                  ............................................................
A0A1E3BNS4_Acristatus_TGH               ............................................................
I7ZRW9_Aoryzae_TGH                      ................SSHVFLFF......Q.............................
A0A0F8UC36_Aochraceoroseus_TGH          ............................................................
Q5BE58_Enidulans_TGH                    ............................................................
A0A0A2KYD8_Pitalicum_TGH                ............................................................
A0A1V6QJR5_Pantarcticum_TGH             ............................................................
A0A1Q5UD44_Psubrubescens_TGH            ............................................................
A0A1V6P0U0_Pdecumbens_TGH               ............................................................
A0A0F4YXS3_Remersonii_TGH               ....................................FVQTQAGRWGA.............
```

```
B8LVN1_Tstipitatus_TGH                ............................................................
A0A225B272_Tatroroseus_TGH            ............................................................
A0A0H1BJD0_Bsilverae_TGH              ............................................................
A0A1B7NTS8_Esp._TGH                   ............................................................
C0RYG0_Pbrasiliensis_TGH              ............................................................
C4JTX0_Ureesii_TGH                    ............................................................
R7Z059_Capollinis_TGH                 ............................................................
A0A1S8BM68_Dseriata_TGH               ............................................................
K2RXL0_Mphaseolina_TGH                ............................................................
A0A165JKY3_Xheveae_TGH                ............................................................
A0A0G2GV44_Pchlamydospora_TGH         ............................................................
F0X9I8_Gclavigera_TGH                 ............................................................
S3BTH5_Opiceae_TGH                    ............................................................
A0A0C2J2D4_Sbrasiliensis_TGH          ............................................................
A0A167VN43_Sinsectorum_TGH            ........................EQTRGG.APGR..................EHADAQT
F7W020_Smacrospora_TGH                ............................................................
A0A151N705_Amississippiensis_TGH      ............................................................
A0A286X9R8_Cporcellus_TGH             ............................................................
A0A1S3ARH5_Eeuropaeus_TGH             ............................................................
Q9BRR8_Hsapiens_TGH                   ............................................................
Q9DBM1_Mmusculus_TGH                  ............................................................
L5LZ46_Mdavidii_TGH                   ............................................................
S7PC93_Mbrandtii_TGH                  ............................................................
A0A1S3FZ16_Dordii_TGH                 ............................................................
F6Z6Q3_Mdomestica_TGH                 ............................................................
G3WTW2_Sharrisii_TGH                  ............................................................
Q21827_Celegans_TGH                   ............................................................
Q9VUA0_Dmelanogaster_TGH              ............................................................
```

```
Aco012726.1_Acomosus_TGH                ............................................................
A0A199V6F8_Acomosus_TGH                 ............................................................
M0TMB2_Macuminata_TGH                   ...........................LSSFARDGDD.......................
A0A2H3XE04_Pdactylifera_TGH             ...........................VPPFNGDGHG.......................
A0A0Q3H630_Bdistachyon_TGH              ............................................................
J3MEA3_Obrachyantha_TGH                 ............................................................
B8B2G4_Osativa_TGH                      ............................................................
K3XV17_Sitalica_TGH                     ..........................L.................................
Q8GXN9_Athaliana_TGH                    ............................................................
Bol036258_Boleraceacapitata_TGH         ............................................................
V4MKB5_Esalsugineum_TGH                 ............................................................
A0A087GCN0_Aalpina_TGH                  ............................................................
A0A151SPT8_Ccajan_TGH                   ............................................................
I1K185_Gmax_TGH                         ............................................................
V7CD35_Pvulgaris_TGH                    ............................................................
A0A1S3UTP2_Vradiata_TGH                 ............................................................
A0A1S2YR44_Carietinum_TGH               ............................................................
G7JT16_Mtruncatula_TGH                  ............................................................
A0A1U8FWC2_Cannuum_TGH                  ............................................................
V4TW69_Cclementina_TGH                  ............................................................
A0A1R3G1K6_Ccapsularis_TGH              ............................................................
A0A1R3H0J1_Colitorius_TGH               ............................................................
A0A0D2QRF0_Graimondii_TGH               ............................................................
A0A067JJA6_Jcurcas_TGH                  ............................................................
B9RE60_Rcommunis_TGH                    ............................................................
SapurV1A.0789s0080.1_SSpurpurea_TGH     ............................................................
A0A2I4E4A1_Jregia_TGH                   ............................................................
F6GTG8_Vvinifera_TGH                    ............................................................
W9S666_Mnotabilis_TGH                   ............................................................
A0A068TQE9_Ccanephora_TGH               ............................................................
A0A200Q3N1_Mcordata_TGH                 ............................................................
A0A1U8AEE6_Nnucifera_TGH                ............................................................
A0A0A0LSK9_Csativus_TGH                 ............................................................
A0A059A5E4_Egrandis_TGH                 ............................................................
A0A218XZU2_Pgranatum_TGH                ............................................................
A0A022RKT3_Eguttata_TGH                 ............................................................
A0A0J8B830_Bvulgaris_TGH                ............................................................
Kalax.0309s0034.1_Klaxiflora_TGH        ............................................................
A0A124SAU9_Ccardunculus_TGH             ............................................................
W1PSA4_Atrichopoda_TGH                  ............................................ICEL............
A0A0K9NIC3_Zmarina_TGH                  ............................................................
A0A2C9VXU1_Mesculenta_TGH               ............................................................
A0A0U9HQK1_Knitens_TGH                  ................................................GVAERNGTVQSG
Sphfalx0151s0047.1_SSfallax_TGH         .....................................FPTPREV................
D8QX10_Smoellendorffii_TGH              ............................................................
E1ZI32_Cvariabilis_TGH                  ............................................................
C1FFR5_Mcommoda_TGH                     ............................................................
A0A086T442_Achrysogenum_TGH             ............................................................
K3V9N2_Fpseudograminearum_TGH           ............................................................
W7LTD5_Gmoniliformis_TGH                ............................................................
G9NMW3_Hatroviridis_TGH                 ............................................................
A0A0G0A2Q9_Tharzianum_TGH               ............................................................
A0A1T3CLH2_Tguizhouense_TGH             ............................................................
A0A084QRJ1_Schlorohalonata_TGH          ............................................................
J4KPK4_Bbassiana_TGH                    ............................................................
A0A2H4SR46_Cmilitaris_TGH               ............................................................
A0A167VSL6_Cfumosorosea_TGH             ............................................................
A0A179G8E9_Pchlamydosporia_TGH          ............................................................
A0A0L0MXB1_Tophioglossoides_TGH         ............................................................
Q2GSN0_Cglobosum_TGH                    ............................................................
G2QES8_Mthermophila_TGH                 ............................................................
A0A175VUX4_Mmycetomatis_TGH             ............................................................
G2QRS6_Tterrestris_TGH                  ............................................................
B2ARI9_Panserina_TGH                    ............................................................
F7W019_Smacrospora_TGH                  ............................................................
A0A1J7J5C9_Cligniaria_TGH               ............................................................
A0A0G2IFE8_Dampelina_TGH                ............................................................
A0A194W011_Vmali_TGH                    ............................................................
R8BKG4_Tminima_TGH                      ............................................................
G0SA63_Cthermophilum_TGH                ............................................................
J3NFM2_Ggraminis_TGH                    ............................................................
A0A0C4DZ99_Mpoae_TGH                    ............................................................
L7IH22_Moryzae_TGH                      ............................................................
A0A1Y2WZC5_Daldiniasp._TGH              ............................................................
A0A1Y2V8J1_Hypoxylon                    ............................................................
A0A1Y2W1E2_Hypoxylon                    ............................................................
A0A1W2TFH3_Rnecatrix_TGH                ............................................................
W3WZ77_Pfici_TGH                        ............................................................
A0A1Y2DPV9_Pvexata_TGH                  ............................................................
A0A136JHX1_Mbolleyi_TGH                 ............................................................
S3CR73_Glozoyensis_TGH                  ............................................................
K1WET1_Mbrunnea_TGH                     ............................................................
A0A218YYN9_Mcoronariae_TGH              ............................................................
A0A194XKL9_Pscopiformis_TGH             ............................................................
A0A0C3DJQ0_Omaius_TGH                   ............................................................
A7ECA3_Ssclerotiorum_TGH                ............................................................
W9CY85_Sborealis_TGH                    ............................................................
A0A094E7L1_Pseudogymnoascus             ............................................................
A0A1B8CGM9_Pseudogymnoascus             ............................................................
A0A094H174_Pseudogymnoascus             ............................................................
Q0D178_Aterreus_TGH                     ............................................................
A0A1L9NNK2_Atubingensis_TGH             ................HVWYSKRRLW..................................
A1CQ12_Aclavatus_TGH                    ............................................................
A0A1L9RVU1_Awentii_TGH                  ............................................................
A0A1E3BNS4_Acristatus_TGH               ............................................................
I7ZRW9_Aoryzae_TGH                      ................HVWYSKRRLW..................................
A0A0F8UC36_Aochraceoroseus_TGH          ............................................................
Q5BE58_Enidulans_TGH                    ............................................................
A0A0A2KYD8_Pitalicum_TGH                ............................................................
A0A1V6QJR5_Pantarcticum_TGH             ............................................................
A0A1Q5UD44_Psubrubescens_TGH            ............................................................
A0A1V6P0U0_Pdecumbens_TGH               ............................................................
A0A0F4YXS3_Remersonii_TGH               ................HVWRSKRRTW..................................
```

```
B8LVN1_Tstipitatus_TGH               ............................................................
A0A225B272_Tatroroseus_TGH           ............................................................
A0A0H1BJD0_Bsilverae_TGH             ............................................................
A0A1B7NTS8_Esp._TGH                  ............................................................
C0RYG0_Pbrasiliensis_TGH             ............................................................
C4JTX0_Ureesii_TGH                   ............................................................
R7Z059_Capollinis_TGH                ............................................................
A0A1S8BM68_Dseriata_TGH              ............................................................
K2RXL0_Mphaseolina_TGH               ............................................................
A0A165JKY3_Xheveae_TGH               ............................................................
A0A0G2GV44_Pchlamydospora_TGH        ............................................................
F0X9I8_Gclavigera_TGH                ............................................................
S3BTH5_Opiceae_TGH                   ............................................................
A0A0C2J2D4_Sbrasiliensis_TGH         ..LP........................................................
A0A167VN43_Sinsectorum_TGH           ERAPQQQQQQEGQPQP............................................
F7W020_Smacrospora_TGH               ............................................................
A0A151N705_Amississippiensis_TGH     ............................................................
A0A286X9R8_Cporcellus_TGH            ............................................................
A0A1S3ARH5_Eeuropaeus_TGH            ............................................................
Q9BRR8_Hsapiens_TGH                  ............................................................
Q9DBM1_Mmusculus_TGH                 ............................................................
L5LZ46_Mdavidii_TGH                  ............................................................
S7PC93_Mbrandtii_TGH                 ............................................................
A0A1S3FZ16_Dordii_TGH                ............................................................
F6Z6Q3_Mdomestica_TGH                ............................................................
G3WTW2_Sharrisii_TGH                 ............................................................
Q21827_Celegans_TGH                  ............................................................
Q9VUA0_Dmelanogaster_TGH             ............................................................
```

Aco012726.1_Acomosus_TGH	....
A0A199V6F8_Acomosus_TGH	....
M0TMB2_Macuminata_TGH	....
A0A2H3XE04_Pdactylifera_TGH	....
A0A0Q3H630_Bdistachyon_TGH	....
J3MEA3_Obrachyantha_TGH	....
B8B2G4_Osativa_TGH	....
K3XV17_Sitalica_TGH	....
Q8GXN9_Athaliana_TGH	....
Bol036258_Boleraceacapitata_TGH	.................................S..........................
V4MKB5_Esalsugineum_TGH	....
A0A087GCN0_Aalpina_TGH	....
A0A151SPT8_Ccajan_TGH	....
I1K185_Gmax_TGH	....
V7CD35_Pvulgaris_TGH	....
A0A1S3UTP2_Vradiata_TGH	....
A0A1S2YR44_Carietinum_TGH	....
G7JT16_Mtruncatula_TGH	....
A0A1U8FWC2_Cannuum_TGH	....
V4TW69_Cclementina_TGH	....
A0A1R3G1K6_Ccapsularis_TGH	....
A0A1R3H0J1_Colitorius_TGH	....
A0A0D2QRF0_Graimondii_TGH	....
A0A067JJA6_Jcurcas_TGH	....
B9RE60_Rcommunis_TGH	....
SapurV1A.0789s0080.1_SSpurpurea_TGH	....
A0A2I4E4A1_Jregia_TGH	....
F6GTG8_Vvinifera_TGH	....
W9S666_Mnotabilis_TGH	....
A0A068TQE9_Ccanephora_TGH	....
A0A200Q3N1_Mcordata_TGH	....
A0A1U8AEE6_Nnucifera_TGH	....
A0A0A0LSK9_Csativus_TGH	....
A0A059A5E4_Egrandis_TGH	....
A0A218XZU2_Pgranatum_TGH	....
A0A022RKT3_Eguttata_TGH	....
A0A0J8B830_Bvulgaris_TGH	....
Kalax.0309s0034.1_Klaxiflora_TGH	....
A0A124SAU9_Ccardunculus_TGH	....
W1PSA4_Atrichopoda_TGH	....
A0A0K9NIC3_Zmarina_TGH	....
A0A2C9VXU1_Mesculenta_TGH	....
A0A0U9HQK1_Knitens_TGH	NGTR..AVMGPLPPDRRREGERKDGLETGGSGR...........................
Sphfalx0151s0047.1_SSfallax_TGH	....
D8QX10_Smoellendorffii_TGH	....
E1ZI32_Cvariabilis_TGH	....
C1FFR5_Mcommoda_TGH	....
A0A086T442_Achrysogenum_TGH	....
K3V9N2_Fpseudograminearum_TGH	....
W7LTD5_Gmoniliformis_TGH	....
G9NMW3_Hatroviridis_TGH	....
A0A0G0A2Q9_Tharzianum_TGH	....
A0A1T3CLH2_Tguizhouense_TGH	....
A0A084QRJ1_Schlorohalonata_TGH	....
J4KPK4_Bbassiana_TGH	....
A0A2H4SR46_Cmilitaris_TGH	....
A0A167VSL6_Cfumosorosea_TGH	....
A0A179G8E9_Pchlamydosporia_TGH	....
A0A0L0MXB1_Tophioglossoides_TGH	....
Q2GSN0_Cglobosum_TGH	....
G2QES8_Mthermophila_TGH	....
A0A175VUX4_Mmycetomatis_TGH	....
G2QRS6_Tterrestris_TGH	....
B2ARI9_Panserina_TGH	....
F7W019_Smacrospora_TGH	....
A0A1J7J5C9_Cligniaria_TGH	....
A0A0G2IFE8_Dampelina_TGH	....
A0A194W011_Vmali_TGH	....
R8BKG4_Tminima_TGH	....
G0SA63_Cthermophilum_TGH	..................................GYYDSRGAGHSSR.............
J3NFM2_Ggraminis_TGH	....
A0A0C4DZ99_Mpoae_TGH	....
L7IH22_Moryzae_TGH	....
A0A1Y2WZC5_Daldiniasp._TGH	....
A0A1Y2V8J1_Hypoxylon	....
A0A1Y2W1E2_Hypoxylon	....
A0A1W2TFH3_Rnecatrix_TGH	....
W3WZ77_Pfici_TGH	....
A0A1Y2DPV9_Pvexata_TGH	....
A0A136JHX1_Mbolleyi_TGH	....
S3CR73_Glozoyensis_TGH	....
K1WET1_Mbrunnea_TGH	....
A0A218YYN9_Mcoronariae_TGH	....
A0A194XKL9_Pscopiformis_TGH	....
A0A0C3DJQ0_Omaius_TGH	....
A7ECA3_Ssclerotiorum_TGH	....
W9CY85_Sborealis_TGH	....
A0A094E7L1_Pseudogymnoascus	....ER.
A0A1B8CGM9_Pseudogymnoascus	....DR.
A0A094H174_Pseudogymnoascus	....E.
Q0D178_Aterreus_TGH	....
A0A1L9NNK2_Atubingensis_TGH	....
A1CQ12_Aclavatus_TGH	....
A0A1L9RVU1_Awentii_TGH	....
A0A1E3BNS4_Acristatus_TGH	....
I7ZRW9_Aoryzae_TGH	....
A0A0F8UC36_Aochraceoroseus_TGH	....
Q5BE58_Enidulans_TGH	....
A0A0A2KYD8_Pitalicum_TGH	....
A0A1V6QJR5_Pantarcticum_TGH	....
A0A1Q5UD44_Psubrubescens_TGH	....
A0A1V6P0U0_Pdecumbens_TGH	....
A0A0F4YXS3_Remersonii_TGH	....

```
B8LVN1_Tstipitatus_TGH                ............................................................
A0A225B272_Tatroroseus_TGH            ............................................................
A0A0H1BJD0_Bsilverae_TGH              ............................................................
A0A1B7NTS8_Esp._TGH                   ............................................................
C0RYG0_Pbrasiliensis_TGH              ............................................................
C4JTX0_Ureesii_TGH                    ............................................................
R7Z059_Capollinis_TGH                 ............................................................
A0A1S8BM68_Dseriata_TGH               ............................................................
K2RXL0_Mphaseolina_TGH                ............................................................
A0A165JKY3_Xheveae_TGH                ............................................................
A0A0G2GV44_Pchlamydospora_TGH         ............................................................
F0X9I8_Gclavigera_TGH                 ............................................................
S3BTH5_Opiceae_TGH                    ............................................................
A0A0C2J2D4_Sbrasiliensis_TGH          ............................................................
A0A167VN43_Sinsectorum_TGH            ............................................................
F7W020_Smacrospora_TGH                ............................................................
A0A151N705_Amississippiensis_TGH      .........................................................F..
A0A286X9R8_Cporcellus_TGH             ............................................................
A0A1S3ARH5_Eeuropaeus_TGH             ............................................................
Q9BRR8_Hsapiens_TGH                   ............................................................
Q9DBM1_Mmusculus_TGH                  ............................................................
L5LZ46_Mdavidii_TGH                   ...............................................PKYSDKGESS.VW
S7PC93_Mbrandtii_TGH                  ............................................................
A0A1S3FZ16_Dordii_TGH                 ............................................................
F6Z6Q3_Mdomestica_TGH                 ............................................................
G3WTW2_Sharrisii_TGH                  ............................................................
Q21827_Celegans_TGH                   ............................................................
Q9VUA0_Dmelanogaster_TGH              ............................................................
```

```
Aco012726.1_Acomosus_TGH                ............................................................
A0A199V6F8_Acomosus_TGH                 ............................................................
M0TMB2_Macuminata_TGH                   ............................................................
A0A2H3XE04_Pdactylifera_TGH             ............................................................
A0A0Q3H630_Bdistachyon_TGH              ............................................................
J3MEA3_Obrachyantha_TGH                 ............................................................
B8B2G4_Osativa_TGH                      ............................................................
K3XV17_Sitalica_TGH                     ............................................................
Q8GXN9_Athaliana_TGH                    ............................................................
Bol036258_Boleraceacapitata_TGH         ............................................................
V4MKB5_Esalsugineum_TGH                 ............................................................
A0A087GCN0_Aalpina_TGH                  ............................................................
A0A151SPT8_Ccajan_TGH                   ............................................................
I1K185_Gmax_TGH                         ............................................................
V7CD35_Pvulgaris_TGH                    ............................................................
A0A1S3UTP2_Vradiata_TGH                 ............................................................
A0A1S2YR44_Carietinum_TGH               ............................................................
G7JT16_Mtruncatula_TGH                  ............................................................
A0A1U8FWC2_Cannuum_TGH                  ............................................................
V4TW69_Cclementina_TGH                  ............................................................
A0A1R3G1K6_Ccapsularis_TGH              ............................................................
A0A1R3H0J1_Colitorius_TGH               ............................................................
A0A0D2QRF0_Graimondii_TGH               ............................................................
A0A067JJA6_Jcurcas_TGH                  ............................................................
B9RE60_Rcommunis_TGH                    ............................................................
SapurV1A.0789s0080.1_SSpurpurea_TGH     ............................................................
A0A2I4E4A1_Jregia_TGH                   ............................................................
F6GTG8_Vvinifera_TGH                    ............................................................
W9S666_Mnotabilis_TGH                   ............................................................
A0A068TQE9_Ccanephora_TGH               ............................................................
A0A200Q3N1_Mcordata_TGH                 ............................................................
A0A1U8AEE6_Nnucifera_TGH                ............................................................
A0A0A0LSK9_Csativus_TGH                 ............................................................
A0A059A5E4_Egrandis_TGH                 ............................................................
A0A218XZU2_Pgranatum_TGH                ............................................................
A0A022RKT3_Eguttata_TGH                 ............................................................
A0A0J8B830_Bvulgaris_TGH                ............................................................
Kalax.0309s0034.1_Klaxiflora_TGH        ............................................................
A0A124SAU9_Ccardunculus_TGH             ............................................................
W1PSA4_Atrichopoda_TGH                  ............................................................
A0A0K9NIC3_Zmarina_TGH                  ............................................................
A0A2C9VXU1_Mesculenta_TGH               ............................................................
A0A0U9HQK1_Knitens_TGH                  ............................................................
Sphfalx0151s0047.1_SSfallax_TGH         ............................................................
D8QX10_Smoellendorffii_TGH              ............................................................
E1ZI32_Cvariabilis_TGH                  ............................................................
C1FFR5_Mcommoda_TGH                     ............................................................
A0A086T442_Achrysogenum_TGH             ............................................................
K3V9N2_Fpseudograminearum_TGH           ............................................................
W7LTD5_Gmoniliformis_TGH                ............................................................
G9NMW3_Hatroviridis_TGH                 ............................................................
A0A0G0A2Q9_Tharzianum_TGH               ............................................................
A0A1T3CLH2_Tguizhouense_TGH             ............................................................
A0A084QRJ1_Schlorohalonata_TGH          ............................................................
J4KPK4_Bbassiana_TGH                    ............................................................
A0A2H4SR46_Cmilitaris_TGH               ............................................................
A0A167VSL6_Cfumosorosea_TGH             ............................................................
A0A179G8E9_Pchlamydosporia_TGH          ............................................................
A0A0L0MXB1_Tophioglossoides_TGH         ............................................................
Q2GSN0_Cglobosum_TGH                    ............................................................
G2QES8_Mthermophila_TGH                 ............................................................
A0A175VUX4_Mmycetomatis_TGH             ............................................................
G2QRS6_Tterrestris_TGH                  ............................................................
B2ARI9_Panserina_TGH                    ............................................................
F7W019_Smacrospora_TGH                  ............................................................
A0A1J7J5C9_Cligniaria_TGH               ............................................................
A0A0G2IFE8_Dampelina_TGH                ............................................................
A0A194W011_Vmali_TGH                    ............................................................
R8BKG4_Tminima_TGH                      ............................................................
G0SA63_Cthermophilum_TGH                ............................................................
J3NFM2_Ggraminis_TGH                    ............................................................
A0A0C4DZ99_Mpoae_TGH                    ............................................................
L7IH22_Moryzae_TGH                      ............................................................
A0A1Y2WZC5_Daldiniasp._TGH              ............................................................
A0A1Y2V8J1_Hypoxylon                    ............................................................
A0A1Y2W1E2_Hypoxylon                    ............................................................
A0A1W2TFH3_Rnecatrix_TGH                ............................................................
W3WZ77_Pfici_TGH                        ............................................................
A0A1Y2DPV9_Pvexata_TGH                  ............................................................
A0A136JHX1_Mbolleyi_TGH                 ............................................................
S3CR73_Glozoyensis_TGH                  ............................................................
K1WET1_Mbrunnea_TGH                     ............................................................
A0A218YYN9_Mcoronariae_TGH              ............................................................
A0A194XKL9_Pscopiformis_TGH             ............................................................
A0A0C3DJQ0_Omaius_TGH                   ............................................................
A7ECA3_Ssclerotiorum_TGH                ............................................................
W9CY85_Sborealis_TGH                    ............................................................
A0A094E7L1_Pseudogymnoascus             ............................................................
A0A1B8CGM9_Pseudogymnoascus             ............................................................
A0A094H174_Pseudogymnoascus             ............................................................
Q0D178_Aterreus_TGH                     ............................................................
A0A1L9NNK2_Atubingensis_TGH             ............................................................
A1CQ12_Aclavatus_TGH                    ............................................................
A0A1L9RVU1_Awentii_TGH                  ............................................................
A0A1E3BNS4_Acristatus_TGH               ............................................................
I7ZRW9_Aoryzae_TGH                      ............................................................
A0A0F8UC36_Aochraceoroseus_TGH          ............................................................
Q5BE58_Enidulans_TGH                    ............................................................
A0A0A2KYD8_Pitalicum_TGH                ............................................................
A0A1V6QJR5_Pantarcticum_TGH             ............................................................
A0A1Q5UD44_Psubrubescens_TGH            ............................................................
A0A1V6P0U0_Pdecumbens_TGH               ............................................................
A0A0F4YXS3_Remersonii_TGH               ............................................................
```

```
B8LVN1_Tstipitatus_TGH                  ............................................................
A0A225B272_Tatroroseus_TGH              ............................................................
A0A0H1BJD0_Bsilverae_TGH                ............................................................
A0A1B7NTS8_Esp._TGH                     ............................................................
C0RYG0_Pbrasiliensis_TGH                ............................................................
C4JTX0_Ureesii_TGH                      ............................................................
R7Z059_Capollinis_TGH                   ............................................................
A0A1S8BM68_Dseriata_TGH                 ............................................................
K2RXL0_Mphaseolina_TGH                  ............................................................
A0A165JKY3_Xheveae_TGH                  ............................................................
A0A0G2GV44_Pchlamydospora_TGH           ............................................................
F0X9I8_Gclavigera_TGH                   ............................................................
S3BTH5_Opiceae_TGH                      ............................................................
A0A0C2J2D4_Sbrasiliensis_TGH            ............................................................
A0A167VN43_Sinsectorum_TGH              ............................................................
F7W020_Smacrospora_TGH                  ............................................................
A0A151N705_Amississippiensis_TGH        ............................................................
A0A286X9R8_Cporcellus_TGH               ............................................................
A0A1S3ARH5_Eeuropaeus_TGH               ............................................................
Q9BRR8_Hsapiens_TGH                     ............................................................
Q9DBM1_Mmusculus_TGH                    ............................................................
L5LZ46_Mdavidii_TGH                     VTLIRAVFQRRRSQTLSSFNPLAMELDVPQQHPAPEKTFWPLSKDAMDGSIIRDFDEGPK
S7PC93_Mbrandtii_TGH                    ............................................................
A0A1S3FZ16_Dordii_TGH                   ............................................................
F6Z6Q3_Mdomestica_TGH                   ............................................................
G3WTW2_Sharrisii_TGH                    ............................................................
Q21827_Celegans_TGH                     ............................................................
Q9VUA0_Dmelanogaster_TGH                ............................................................
```

**Aco012726.1_Acomosus_TGH**	........................E...................................
**A0A199V6F8_Acomosus_TGH**	........................E...................................
**M0TMB2_Macuminata_TGH**	........................H...................................
**A0A2H3XE04_Pdactylifera_TGH**	........................Q...................................
**A0A0Q3H630_Bdistachyon_TGH**	........................K...................................
**J3MEA3_Obrachyantha_TGH**	........................K...................................
**B8B2G4_Osativa_TGH**	........................K...................................
**K3XV17_Sitalica_TGH**	........................K...................................
**Q8GXN9_Athaliana_TGH**	....
**Bol036258_Boleraceacapitata_TGH**	....
**V4MKB5_Esalsugineum_TGH**	....
**A0A087GCN0_Aalpina_TGH**	....
**A0A151SPT8_Ccajan_TGH**	....
**I1K185_Gmax_TGH**	....
**V7CD35_Pvulgaris_TGH**	....
**A0A1S3UTP2_Vradiata_TGH**	....
**A0A1S2YR44_Carietinum_TGH**	....
**G7JT16_Mtruncatula_TGH**	....
**A0A1U8FWC2_Cannuum_TGH**	....
**V4TW69_Cclementina_TGH**	....
**A0A1R3G1K6_Ccapsularis_TGH**	..........VPF...
**A0A1R3H0J1_Colitorius_TGH**	....
**A0A0D2QRF0_Graimondii_TGH**	....
**A0A067JJA6_Jcurcas_TGH**	....
**B9RE60_Rcommunis_TGH**	....
**SapurV1A.0789s0080.1_SSpurpurea_TGH**	....
**A0A2I4E4A1_Jregia_TGH**	....
**F6GTG8_Vvinifera_TGH**	....
**W9S666_Mnotabilis_TGH**	....
**A0A068TQE9_Ccanephora_TGH**	....
**A0A200Q3N1_Mcordata_TGH**	....
**A0A1U8AEE6_Nnucifera_TGH**	....
**A0A0A0LSK9_Csativus_TGH**	....
**A0A059A5E4_Egrandis_TGH**	....
**A0A218XZU2_Pgranatum_TGH**	....
**A0A022RKT3_Eguttata_TGH**	....
**A0A0J8B830_Bvulgaris_TGH**	....
**Kalax.0309s0034.1_Klaxiflora_TGH**	....
**A0A124SAU9_Ccardunculus_TGH**	....
**W1PSA4_Atrichopoda_TGH**	....SITKFCTKELSFARLS
**A0A0K9NIC3_Zmarina_TGH**	....
**A0A2C9VXU1_Mesculenta_TGH**	....
**A0A0U9HQK1_Knitens_TGH**	....
**Sphfalx0151s0047.1_SSfallax_TGH**	....
**D8QX10_Smoellendorffii_TGH**	....
**E1ZI32_Cvariabilis_TGH**	....
**C1FFR5_Mcommoda_TGH**	....
**A0A086T442_Achrysogenum_TGH**	.....R.......PPTEAHLIEML....................................
**K3V9N2_Fpseudograminearum_TGH**	.....R.......PPTEAHLIEML....................................
**W7LTD5_Gmoniliformis_TGH**	.....R.......PPTEAHLIEML....................................
**G9NMW3_Hatroviridis_TGH**	.....R.......PPTEAHLIEML....................................
**A0A0G0A2Q9_Tharzianum_TGH**	.....R.......PPTEAHLIEML....................................
**A0A1T3CLH2_Tguizhouense_TGH**	.....R.......PPTEAHLIEML....................................
**A0A084QRJ1_Schlorohalonata_TGH**	.....R.......PPTEAHLIEML....................................
**J4KPK4_Bbassiana_TGH**	.....R.......PPTEAHLIEML....................................
**A0A2H4SR46_Cmilitaris_TGH**	.....R.......PPTEAHLIEML....................................
**A0A167VSL6_Cfumosorosea_TGH**	....
**A0A179G8E9_Pchlamydosporia_TGH**	....
**A0A0L0MXB1_Tophioglossoides_TGH**	....
**Q2GSN0_Cglobosum_TGH**	....
**G2QES8_Mthermophila_TGH**	....
**A0A175VUX4_Mmycetomatis_TGH**	....
**G2QRS6_Tterrestris_TGH**	....
**B2ARI9_Panserina_TGH**	....
**F7W019_Smacrospora_TGH**	....
**A0A1J7J5C9_Cligniaria_TGH**	....
**A0A0G2IFE8_Dampelina_TGH**	....
**A0A194W011_Vmali_TGH**	....
**R8BKG4_Tminima_TGH**	....
**G0SA63_Cthermophilum_TGH**	.....R.......PPTEAHLIEML....................................
**J3NFM2_Ggraminis_TGH**	....
**A0A0C4DZ99_Mpoae_TGH**	....
**L7IH22_Moryzae_TGH**	.....R.......PPTEAHLIEML....................................
**A0A1Y2WZC5_Daldiniasp._TGH**	....
**A0A1Y2V8J1_Hypoxylon**	....
**A0A1Y2W1E2_Hypoxylon**	....
**A0A1W2TFH3_Rnecatrix_TGH**	....
**W3WZ77_Pfici_TGH**	....
**A0A1Y2DPV9_Pvexata_TGH**	....
**A0A136JHX1_Mbolleyi_TGH**	....
**S3CR73_Glozoyensis_TGH**	....
**K1WET1_Mbrunnea_TGH**	....
**A0A218YYN9_Mcoronariae_TGH**	....
**A0A194XKL9_Pscopiformis_TGH**	....
**A0A0C3DJQ0_Omaius_TGH**	....
**A7ECA3_Ssclerotiorum_TGH**	....
**W9CY85_Sborealis_TGH**	....
**A0A094E7L1_Pseudogymnoascus**	.....R..V....PPTEQHLIEML....................................
**A0A1B8CGM9_Pseudogymnoascus**	.....R..T....PPTEQHLIEML....................................
**A0A094H174_Pseudogymnoascus**	.....R.......PPTEQHLIEML....................................
**Q0D178_Aterreus_TGH**	....
**A0A1L9NNK2_Atubingensis_TGH**	....
**A1CQ12_Aclavatus_TGH**	....
**A0A1L9RVU1_Awentii_TGH**	....
**A0A1E3BNS4_Acristatus_TGH**	....
**I7ZRW9_Aoryzae_TGH**	....
**A0A0F8UC36_Aochraceoroseus_TGH**	....
**Q5BE58_Enidulans_TGH**	....
**A0A0A2KYD8_Pitalicum_TGH**	....
**A0A1V6QJR5_Pantarcticum_TGH**	....
**A0A1Q5UD44_Psubrubescens_TGH**	....
**A0A1V6P0U0_Pdecumbens_TGH**	....
**A0A0F4YXS3_Remersonii_TGH**	....

**B8LVN1_Tstipitatus_TGH**	....
**A0A225B272_Tatroroseus_TGH**	....
**A0A0H1BJD0_Bsilverae_TGH**	....
**A0A1B7NTS8_Esp._TGH**	....
**C0RYG0_Pbrasiliensis_TGH**	....
**C4JTX0_Ureesii_TGH**	....
**R7Z059_Capollinis_TGH**	....
**A0A1S8BM68_Dseriata_TGH**	....
**K2RXL0_Mphaseolina_TGH**	....
**A0A165JKY3_Xheveae_TGH**	....
**A0A0G2GV44_Pchlamydospora_TGH**	....
**F0X9I8_Gclavigera_TGH**	....
**S3BTH5_Opiceae_TGH**	....
**A0A0C2J2D4_Sbrasiliensis_TGH**	.....R.......PPTEAHLIEML....................................
**A0A167VN43_Sinsectorum_TGH**	.....RTN.....PPTEAHLIEML....................................
**F7W020_Smacrospora_TGH**	.....R...G...PPTEAHLIEML....................................
**A0A151N705_Amississippiensis_TGH**	.........................HHGPIVL........LQCN................
**A0A286X9R8_Cporcellus_TGH**	....
**A0A1S3ARH5_Eeuropaeus_TGH**	....
**Q9BRR8_Hsapiens_TGH**	....
**Q9DBM1_Mmusculus_TGH**	....
**L5LZ46_Mdavidii_TGH**	APVLE...........................FSTYDSDV....................
**S7PC93_Mbrandtii_TGH**	....
**A0A1S3FZ16_Dordii_TGH**	....
**F6Z6Q3_Mdomestica_TGH**	....
**G3WTW2_Sharrisii_TGH**	....
**Q21827_Celegans_TGH**	....
**Q9VUA0_Dmelanogaster_TGH**	....

```
Aco012726.1_Acomosus_TGH               ........................
A0A199V6F8_Acomosus_TGH                ........................
M0TMB2_Macuminata_TGH                  ........................
A0A2H3XE04_Pdactylifera_TGH            ........................
A0A0Q3H630_Bdistachyon_TGH             ........................
J3MEA3_Obrachyantha_TGH                ........................
B8B2G4_Osativa_TGH                     ........................
K3XV17_Sitalica_TGH                    ........................
Q8GXN9_Athaliana_TGH                   ........................
Bol036258_Boleraceacapitata_TGH        ........................
V4MKB5_Esalsugineum_TGH                ........................
A0A087GCN0_Aalpina_TGH                 ........................
A0A151SPT8_Ccajan_TGH                  ........................
I1K185_Gmax_TGH                        ........................
V7CD35_Pvulgaris_TGH                   ........................
A0A1S3UTP2_Vradiata_TGH                ........................
A0A1S2YR44_Carietinum_TGH              ........................
G7JT16_Mtruncatula_TGH                 ........................
A0A1U8FWC2_Cannuum_TGH                 ........................
V4TW69_Cclementina_TGH                 ........................
A0A1R3G1K6_Ccapsularis_TGH             ........................
A0A1R3H0J1_Colitorius_TGH              ........................
A0A0D2QRF0_Graimondii_TGH              ........................
A0A067JJA6_Jcurcas_TGH                 ........................
B9RE60_Rcommunis_TGH                   ........................
SapurV1A.0789s0080.1_SSpurpurea_TGH    ........................
A0A2I4E4A1_Jregia_TGH                  ........................
F6GTG8_Vvinifera_TGH                   ........................
W9S666_Mnotabilis_TGH                  ........................
A0A068TQE9_Ccanephora_TGH              ........................
A0A200Q3N1_Mcordata_TGH                ........................
A0A1U8AEE6_Nnucifera_TGH               ........................
A0A0A0LSK9_Csativus_TGH                ........................
A0A059A5E4_Egrandis_TGH                ........................
A0A218XZU2_Pgranatum_TGH               ........................
A0A022RKT3_Eguttata_TGH                ........................
A0A0J8B830_Bvulgaris_TGH               ........................
Kalax.0309s0034.1_Klaxiflora_TGH       ........................
A0A124SAU9_Ccardunculus_TGH            ........................
W1PSA4_Atrichopoda_TGH                 ASVLASLQLNPIRRG.DN......
A0A0K9NIC3_Zmarina_TGH                 ........................
A0A2C9VXU1_Mesculenta_TGH              ........................
A0A0U9HQK1_Knitens_TGH                 ........................
Sphfalx0151s0047.1_SSfallax_TGH        ........................
D8QX10_Smoellendorffii_TGH             ........................
E1ZI32_Cvariabilis_TGH                 ........................
C1FFR5_Mcommoda_TGH                    ........................
A0A086T442_Achrysogenum_TGH            ........................
K3V9N2_Fpseudograminearum_TGH          ........................
W7LTD5_Gmoniliformis_TGH               ........................
G9NMW3_Hatroviridis_TGH                ........................
A0A0G0A2Q9_Tharzianum_TGH              ........................
A0A1T3CLH2_Tguizhouense_TGH            ........................
A0A084QRJ1_Schlorohalonata_TGH         ........................
J4KPK4_Bbassiana_TGH                   ........................
A0A2H4SR46_Cmilitaris_TGH              ........................
A0A167VSL6_Cfumosorosea_TGH            ........................
A0A179G8E9_Pchlamydosporia_TGH         ........................
A0A0L0MXB1_Tophioglossoides_TGH        ........................
Q2GSN0_Cglobosum_TGH                   ........................
G2QES8_Mthermophila_TGH                ........................
A0A175VUX4_Mmycetomatis_TGH            ........................
G2QRS6_Tterrestris_TGH                 ........................
B2ARI9_Panserina_TGH                   ........................
F7W019_Smacrospora_TGH                 ........................
A0A1J7J5C9_Cligniaria_TGH              ........................
A0A0G2IFE8_Dampelina_TGH               ........................
A0A194W011_Vmali_TGH                   ........................
R8BKG4_Tminima_TGH                     ........................
G0SA63_Cthermophilum_TGH               ........................
J3NFM2_Ggraminis_TGH                   ........................
A0A0C4DZ99_Mpoae_TGH                   ........................
L7IH22_Moryzae_TGH                     ........................
A0A1Y2WZC5_Daldiniasp._TGH             ........................
A0A1Y2V8J1_Hypoxylon                   ........................
A0A1Y2W1E2_Hypoxylon                   ........................
A0A1W2TFH3_Rnecatrix_TGH               ........................
W3WZ77_Pfici_TGH                       ........................
A0A1Y2DPV9_Pvexata_TGH                 ........................
A0A136JHX1_Mbolleyi_TGH                ........................
S3CR73_Glozoyensis_TGH                 ........................
K1WET1_Mbrunnea_TGH                    ........................
A0A218YYN9_Mcoronariae_TGH             ........................
A0A194XKL9_Pscopiformis_TGH            ........................
A0A0C3DJQ0_Omaius_TGH                  ........................
A7ECA3_Ssclerotiorum_TGH               ........................
W9CY85_Sborealis_TGH                   ........................
A0A094E7L1_Pseudogymnoascus            ........................
A0A1B8CGM9_Pseudogymnoascus            ........................
A0A094H174_Pseudogymnoascus            ........................
Q0D178_Aterreus_TGH                    ........................
A0A1L9NNK2_Atubingensis_TGH            ..................HVISS.
A1CQ12_Aclavatus_TGH                   ........................
A0A1L9RVU1_Awentii_TGH                 ........................
A0A1E3BNS4_Acristatus_TGH              ........................
I7ZRW9_Aoryzae_TGH                     ..................NVINSC
A0A0F8UC36_Aochraceoroseus_TGH         ........................
Q5BE58_Enidulans_TGH                   ........................
A0A0A2KYD8_Pitalicum_TGH               ........................
A0A1V6QJR5_Pantarcticum_TGH            ........................
A0A1Q5UD44_Psubrubescens_TGH           ........................
A0A1V6P0U0_Pdecumbens_TGH              ........................
A0A0F4YXS3_Remersonii_TGH              ........................
```

```
B8LVN1_Tstipitatus_TGH                 ........................
A0A225B272_Tatroroseus_TGH             ........................
A0A0H1BJD0_Bsilverae_TGH               ........................
A0A1B7NTS8_Esp._TGH                    ........................
C0RYG0_Pbrasiliensis_TGH               ........................
C4JTX0_Ureesii_TGH                     ........................
R7Z059_Capollinis_TGH                  ........................
A0A1S8BM68_Dseriata_TGH                ........................
K2RXL0_Mphaseolina_TGH                 ........................
A0A165JKY3_Xheveae_TGH                 ........................
A0A0G2GV44_Pchlamydospora_TGH          ........................
F0X9I8_Gclavigera_TGH                  ........................
S3BTH5_Opiceae_TGH                     ........................
A0A0C2J2D4_Sbrasiliensis_TGH           ........................
A0A167VN43_Sinsectorum_TGH             ........................
F7W020_Smacrospora_TGH                 ...............V........
A0A151N705_Amississippiensis_TGH       ........................
A0A286X9R8_Cporcellus_TGH              ........................
A0A1S3ARH5_Eeuropaeus_TGH              ........................
Q9BRR8_Hsapiens_TGH                    ........................
Q9DBM1_Mmusculus_TGH                   ........................
L5LZ46_Mdavidii_TGH                    ........................
S7PC93_Mbrandtii_TGH                   ........................
A0A1S3FZ16_Dordii_TGH                  ........................
F6Z6Q3_Mdomestica_TGH                  ........................
G3WTW2_Sharrisii_TGH                   ........................
Q21827_Celegans_TGH                    ........................
Q9VUA0_Dmelanogaster_TGH               ........................
```
