# Supplementary material for: Molecular Evolution and Diversification of Proteins Involved in miRNA Maturation Pathway
Source: Plants (Basel). 2020 Mar 1;9(3):299. doi: 10.3390/plants9030299 (PMC7154892; doi:10.3390/plants9030299)
Supplement: Supplementary file 1 [file Supplementary File S1.pdf › Supplementary Files/Supplementary File S1.pdf]

```
Cre09.g390430.t1.1.cre.30781206
                                    β1      β2   β3     β4      β5          β6             β7         β8 β9   β10   β11   β12
                                  --->TT-> -> ---> -> TT ------> TT ------.--> -->TT-> TT -> TT -->TT --->
                                  1        10        20        30        40        50        60        70        80        90        100       110

Cre09.g390430.t1.1.cre.30781206   WRLYIFKNDQLQDEPYHIHRMDHYLFGRDLTVADIVTAHPSCSKQHAVLQFRLTEKADEYGLAVGPARPY.LLDLGSINGTFLNGEKVEPLRYYELLEKDVVRFGQSSREYVLLHDRSGG
Vocar.0056s0018.1.p.vca.32896672  WRLYIFKNDQLQDEPYRIHRMNHYLFGRDLQVADIITAHPSCSKQHAVLQFRLTEKNDELG...RPVRPY.LLDLGSVNGTFLNGERLEPLRYYELLEKDVVRLGQSSREYVLLHDKSG.
14945.mpu.27347251                WRLYVFKNGELTGEPLHIHRQSYLFGRERKVVDVPTDHPSCSKQHAVIQYRERTKDDDEGADVKVAVPY.IMDLNSTNGTHLNGDRIEPQRYYELLEKDTIKFGMSTREYVLLNEDSK.
DCAR_019897.dca.36084630          WRLYVFKGGDVLNEPLYVHRQTSYLFGRERRVADIPTDHPSCSKQHAVLQYRQVETEQPDGTLSKQVRPY.IMDLGSTNGTFINEERIDPQRYYELREKDTIKFGNSAREFSGIMDDSEG
Manes.03G127500.1.p.mes.32363355  WRLYVFKNGEVLNEPLYIHRQTCYLFGRERRVADIPTDHPSCSKQHAVIQFRQVEKDQPDGTISKQVRII.RLNLNVIKEILLNLVTVAENMYYRIQQADGCRLAS..............
Sevir.3G283500.1.p.svi.32676284   WRLYVFKGGEPLNEPLYVHRLTCYLFGRERKVADIPTDHPSCSKQHAVLQYRLVEKEQPDGMMAKKVRPY.LMDLDSTNGTFINENRIEPRRYYELFEKDTIKFGNSSREYVLLHENSTG
Seita.3G276000.1.p.sit.32712291   WRLYVFKGGEPLNEPLYVHRLTCYLFGRERKVADIPTDHPSCSKQHAVLQYRLVEKEQPDGMMAKKVRPY.LMDLDSTNGTFINENRIEPRRYYELFEKDTIKFGNSSREYVLLHENSTG
Sobic.009G105800.1.p.sbi.37919000 WRLYVFKDGEPLNEPLYVHRMTCYLFGRERKVADVPTDHPSCSKQHAVLQYRLVEKEQPDGMMAKKIRPY.LMDLDSTNGTFINGNRIEPRRYYELFEKDTIKFGNSSREYVLLHENSTE
GRMZM2G113156_P03.zma.31024620    WRLYVFKDGEPLNEPLYVHRMTCYLFGRERKVADVPTDHPSCSKQHAVLQYRLVEKEQLDGMMTKKIRPY.LMDLDSTNGTFINGNRIEPRRYYELFEKDTLKFGNSSREYVLLHENSTE
Zm00008a025628_P01.zma.37219100   WRLYVFKDGEPLNEPLYVHRMTCYLFGRERKVADVPTDHPSCSKQHAVLQYRLVEKEQLDGMMTKKIRPY.LMDLDSTNGTFINGNRIEPRRYYELFEKDTIKFGNSRYDLNHLKQQNED
Pahal.F02843.1.pha.32514749       WSPYVFKGGEPLNGSCSLDLFT..............PCMNTIFV.........................SCHGFC............................................
Pavir.Cb01329.1.p.pvi.30267644    WRLYVFKGGEPLNGSCSLNLFT..............PCMNTIFA.........................SCHGFY............................................
LOC_Os05g46780.1.osa.33156821     WRLYVFKGGEPLEEPLYVHRMSSYLFGRERKVADIPTDHPSCSKQHAVLQYRLVEKEQPDGMMSKQVRPY.LMDLGSTNGTFINENRIEPSRYYELFEKDTIKFGNSSREYVLLHENSKD
Bradi4g26200.4.p.bdi.32787504     WRLYVFKGGEALNEPLYVHRMSHYLFGRERRVADIPTDHPSCSKQHAVLQYRLVDKEQPDGMMAKKVRPY.LMDLGSTNGTFINENRIESHRYYELFERDNIKFGNSSREYVLLHENSTG
Brast05G008800.1.p.bst.32867190   WRLYVFKGGEALNEPLYVHRMSHYLFGRERRVADIPTDHPSCSKQHAVLQYRLVDKEQPDGMMAKKVRPY.LMDLGSTNGTFINENRIESHRYYELFERDNIKFGNSSREYVLLHENSTG
GSVIVT01030604001.vvi.17837076    WRLYVFKAGEVLNEPLYIHRQSCYLFGRERRVADVPTDHPSCSKQHAVVQFRQIEKEQPDGMLSKQVRPY.LMDLGSTNGTFINDSRIEPQRYYELFEKDTIKFGNSSREYVILHENSTD
Eucgr.F00968.1.p.egr.32052537     WRLYVFKGGEVLNEPLYIHRQSCYLFGRERRVADIPTDHPSCSKQHAVIQFRQVEKEQPDGTLSKKTRPY.LMDLGSTNKTLLNENPIEPQRYYELFEKDTIKFGNSSREYVLLHENSAG
Prupe.4G208000.1.p.ppe.32092913   WRLYVFKGGEVLNEPLYIHRQSCYLFGRERRVADVPTDHPSCSKQHAVIQFRQVEMEQPDGTLSKKVRPY.LMDLGSTNKTFLNDTAIEPQRYYELMEKDTIRFGNSSREYVLLHENSMN
orange1.1g010574m.csi.18098403    WRLYVFKAGEMLKEPLYIHRQSCYLFGRERRVADIPTDHPSCSKQHAVIQFRQVEKEQPDGMLSKEVRPY.IMDLGSTNKTYLNDNPIEPQRYYELFEKDTIKFGNSSREYVLLHENSND
Ciclev10008061m.ccl.20793131      WRLYVFKAGEMLKEPLYIHRQSCYLFGRERRVADIPTDHPSCSKQHAVIQFRQVEKEQPDGMLSKEVRPY.IMDLGSTNKTYLNDNPIEPQRYYELFEKDTIKFGNSSREYVLLHENSND
Migut.H01845.1.p.mgu.28952892     WRLYVFKGGEVLNEPLYVHRQSCYLFGRERRVADIPTDHPSCSKQHAVLQYRQVEEENPDGTMSKRVKPY.VMDLGSTNGTFINENQIEPQRYYELMEKDTVKFGNSSREYVILHENSAE
Solyc10g006390.2.1.sly.36154699   WRLYVFKGGEVLNDPLYVHRQSCYLFGRERRVADVPTDHPSCSKQHAVLQYRQVEKDKPDGTSSKQVRPY.VMDLGSTNGTFINENRIEPERYYELFEKDTLKFGNSSREYVLLHENSA.
PGSC0003DMP400025420.stu.37478734 WRLYVFKGGEVLNEPLYVHRQSCYLFGRERRVADIPTDHPSCSKQHAVLQYRQVEKDKPDGTSSKQVRPY.VMDLGSTNGTFINENRIEPERYYELLEKDTLKFGNSSREYVLLHENSA.
Brara.C03794.1.p.bra.30617963     WRLYVFKDSEPLDEPLQLHRQSCYLFGRERRIADIPTDHPSCSKQHAVIQYREVTVEKPDGMVEKQVKPY.LMDLGSTNKTYINKDPIEPQRYYELREKDTIKFGNSSREYVLLHEGSAE
Bol026651.bol.37345714            WRLYVFKDSEPLDEPLQLHRQSCYLFGRERRIADIPTDHPSCSKQHAVIQYREVTVEKPDGMVEKQVKPY.LMDLGSTNKTYINKDPIEPQRYYELHEKDTIKFGNSSREYVLLHEGSAE
Bostr.19424s0598.1.p.adr.30661583 WRLYVFKDGEPLNEPLYLHRQSCYLFGRERRIADIPTDHPSCSKQHAVIQYREVEKEKPDGMMGKQVRPY.IMDLGSTNKTYINV.....................NSHRATKIL......
Carubv10014011m.cru.20898832      WRLYVFKGGEPLNEPLYLHRQSCYLFGRERRIADIPTDHPSCSKQHAVIQYREMEKEKPNGMMGKQVKPY.IMDLGSTNKTYINEKPIEPQRYYELFEKDTIKFGNSSREYVLLHENSAE
Cagra.0316s0011.1.p.cgr.28915685  WRLYVFKGGEPLNEPLYLHRQSCYLFGRERRIADIPTDHPSCSKQHAVIQYREMEKEKPNGMVGKQVKPY.IMDLGSTNKTYINEKPIEPQRYYELFEKDTIKFGNSSREYVLLHENSAE
Araha.13920s0006.1.p.aha.28852390 WRLYVFKDGEPLNEPLCLHRQSCYLFGRERRIADIPTDHPSCSKQHAVIQYREMEKEKPDGMMGKQVKPY.IMDLGSTNKTYINESPIEPQRYYELFEKDTIKFGNSSREYVLLHENSAE
AL3G34380.t1.aly.35940399         WRLYVFKDGEPLNEPLCLHRQSCYLFGRERRIADIPTDHPSCSKQHAVIQYREMEKEKPDGMMGKQVKPY.IMDLGSTNKTYINESPIEPQRYYELFEKDTIKFGNSSREYVLLHENSAE
AT3G20550.1.ath.19659615          WRLYVFKDGEPLNEPLCLHRQSCYLFGRERRIADIPTDHPSCSKQHAVIQYREMEKEKPDGMMGKQVKPY.IMDLGSTNKTYINESPIEPQRYYELFEKDTIKFGNSSREYVLLHENSAE
Potri.001G420400.1.ptr.27042006   WRLYVFKGGEALNEPLYIHRQSCYLFGRERRVADIPTDHPSCSKQHAVIQFRQVEKEQPDGMLKKQVRPY.VMDLGSTNKTFINDNPIEPQRYYELFEKDTIKFGNSSREYVLLHENSSE
SapurV1A.0784s0090.1.p.spu.31437518 WRLYVFKGGEALNEPLYIHRQSCYLFGRERRVADIPTDHPSCSKQHAVIQFRQVEKEQRDGMLKKQPR.....ICTAARELSRMMFIKRLLQYGFLTSAFWRIKNILFIHQILF.....
Medtr3g049440.1.mtr.31060687      WRLYVFKTGEMLNEPLYIHRQSCYLFGRERRVADVPTDHPSCSKQHAVIQFRQVEKEQPDGMIVKQTRPY.IMDLGSTNKTFVNDSPIEPQRYYELREQDTIKFGNSSREYVLLHENSAS
Phvul.006G037500.1.p.pvu.37173844 WRLYVFKGGEVLNEPLYIHRQSCYLFGRERRVADIPTDHPSCSKQHAVIQFRQVEKEQADGTLLKQIRPY.IMDLGSTNKTLINDSPIEPQRYYELREKDTIKFGNSSREYVLLHENSMG
Glyma.09G101200.1.p.gma.30483085  WRLYVFKAGEVLNEPLYIHRQSCYLFGRERRVADIPTDHPSCSKQHAVIQFRQVEKEQPDGTLLKQVRPY.VMDLGSTNKTFINDSPIEPQRYYELKEKDTIKFGNSSREYVLLHENSIG
Glyma.18G102900.1.p.gma.30556844  WRLYVFKAGEVLNEPLYIHRQSCYLFGRERRVADIPTDHPSCSKQHAVIQFRQVEKEQPDGTLLKQVRPY.IMDLGSTNKTFINDGPIEPQRYYELREKDTIKFGNSSREYVLLHENSIG
Kalax.1450s0004.1.p.kla.32537357  WRLYVFKAGEMLNEPLYIHRQSCYLFGRERRIADIPTDHPSCSKQHAVIQYRQIEKEQPDGMLKKITRPY.LMDLGSTNGTFINDNRLEPQRYYELIEQDTIKFGNSSREYVILHENSAK
Kaladp0037s0494.1.p.kfe.35775181  WRLYVFKAGEMLNEPLYIHRQSCYLFGRERRIADIPTDHPSCSKQHAVIQYRQIEKEQPDGMLKKITRPY.LMDLGSTNGTFINDNRLEPQRYYELIEQDTIKFGNSSREYVILHENSAK
Aqcoe1G346800.1.p.aco.33079128    WRLYVFKAGEVLNDPLYIHRQTCYLFGRERRVADVPTDHPSCSKQHAVIQFRQIEKEQPDGMLSKQVRPY.LMDLGSTNGTFLNENRIEPQRYYELFEKDTIKFGNSSREYVVLHENSVS
mrna03205.1-v1.0-hybrid.fve.27274674 WRLYVFKGGEVLDG.......................................KSLES..........................................................
Aqcoe5G248600.1.p.aco.33090844    WRLYVFKAGEMGNEPLYIHRQSCYLFGRERRVADIPTDHPSCSKQHAVIQFRQIEKEDADGIFLKKVRPY.LIDLGSTNGTFVNGNRIDAERYYELFEKDTIKFGNSSREFVILHEKSA.
Sphfalx0354s0008.1.p.sfa.32606484 WRLYVFKDGEALNDPLYIHRQTCYLFGRERRVADIPTDHPSCSKQHAVIQYRLTEKEEPDGMIASKVRPY.IMDLGSTNGTFLNGKHIDPQRYYELLETDTIKFGNSSREYVLLHEHSTG
Pp3c19_14080V3.1.p.ppa.32939346   WRLYVFKDGEPMNEPLYIHRQTCYLFGRERKKVADVPTDHPSCSKQHAVIQYRLTEIEGPDGMMESKVRPY.IMDLGSTNGTHLNNTRIESRRYYELREKDTLKFGNSSREYVLLHENSAA
XP_008875863.1                    WRLYVFKNDENV.ATLHLHRQSAYLFGREKKVADVYLEHGSISKQHAVVQFRMRHKEQPE..LVSEVLPY.LMDLKSTNHTFLNGKQIEHSRYIQLKEKDVITFGESTREYVLLKDTKGS
XP_002289349.1                    WRLYVFRDD..LIDTYHISRQSAYLFGRERKVADIPVDHPSLSKQHAVLQYRALPSNEPD...KLQCRPY.LMDLESTNGTFINGVRLDSARYYELRRGDVITLGASSREYVLLTEQSA.
tr|W5MFC8|W5MFC8_LEPOC            WRLYPFKNDEPL.PVMYIHRQSAYLLGRQRKIADIPIDHPSCSKQHAVFQYRLVEFTRSDGTTGRRVKPY.IIDLGSGNGTYLNNQRIEPQRYYELKEKDVLKFGFSSREYVLLHESSDT
tr|A0A498LC39|A0A498LC39_LABRO    WRLYPFKNDEPL.PVMYIHRQSAYLLGRQRKIADIPIDHPSCSKQHAVFQYRLVEFTRADGTMGRRVKPY.IIDLGSGNGTYLNNQRIEPQRYYELKEKDVLKFGFSSREYVLLHEFSDT
tr|Q4V8W8|Q4V8W8_DANRE            WRLYPFKNDEPL.PVMYIHRQSAYLLGRLRKIADIPIDHPSCSKQHAVFQYRLVEFTRVDGTAGRRVKPY.IIDLGSGNGTYLNNQRIEPQRYYELKEKDVLKFGFSSREYVLLHEFSDT
tr|H3BC77|H3BC77_LATCH            WRLYPFKNDEPL.PVMYIHRQSAYLLGRQRRIADIPIDHPSCSKQHAVLQYRLVEFTRSDGTVGRRVKPY.IIDLGSGNGTFLNNQLIESHRYYELKEKDVLKFGFSSREYVLLHESSDT
tr|G7NUA1|G7NUA1_MACFA            WRLYPFKNDEVL.PVMYIHRQSAYLLGRHRRIADIPIDHPSCSKQHAVFQYRLVEYTRADGTVGRRVKPY.IIDLGSGNGTFLNNKRIEPQRYYELKEKDVLKFGFSSREYVLLHESSDT
NP_078976.2                       WRLYPFKNDEVL.PVMYIHRQSAYLLGRHRRIADIPIDHPSCSKQHAVFQYRLVEYTRADGTVGRRVKPY.IIDLGSGNGTFLNNKRIEPQRYYELKEKDVLKFGFSSREYVLLHESSDT
sp|Q5M9G6|SNIP1_RAT               WRLYPFKNDEVL.PVMYIHRQSAYLLGRHRRIADIPIDHPSCSKQHAVFQYRLVEYTRADGTVGRRVKPY.IIDLGSGNGTFLNNKRIEPQRYYELKEKDVLKFGFSSREYVLLHESSDT
sp|Q8BIZ6|SNIP1_MOUSE             WRLYPFKNDEVL.PVMYIHRQSAYLLGRHRRIADIPIDHPSCSKQHAVFQYRLVEYTRADGTVGRRVKPY.IIDLGSGNGTFLNNKRIEPQRYYELKEKDVLKFGFSSREYVLLHESSDT
tr|A0A1U7SMF0|A0A1U7SMF0_ALLSI    WRLYPFKNDEVL.PVMYIHRQSAYLLGRHRRIADIPIDHPSCSKQHAVFQYRLVEYTRADGTVGRRVKPY.IIDLGSGNGTFLNNQRIEPKRYYELKEKDVLKFGFSSREYVLLHESSDK
tr|A0A1L8H7R2|A0A1L8H7R2_XENLA    WRLYPFKNDEAL.PVMYVHRQSAYLLGRQRRIADIPVDHPSCSKQHAVLQYRMVEFTRANGTSGRRVRPY.IIDLGSGNGTYLNNQRIEPQRYYELKEKDVLKFGFSSREYVVLHETSDT
tr|Q6DDL6|Q6DDL6_XENLA            WRLYPFKNDEAL.PVMYVHRQSAYLMGRQRRIADIPIDHPSCSKQHAVLQYRMVEFTRANGTSGRRVRPY.IIDLGSGNGTYLNNQRIEPQRYYELKEKDVLKFGFSSREYVVLHETSDT
tr|Q6PBD3|Q6PBD3_XENTR            WRLYPFKNDEAL.PVMYIHRQSAYLLGRQRRIADIPIDHPSCSKQHAVLQYRMVQFTRANGTSGRRVKPY.IIDLGSGNGTYLNNQRIEPQRYYELKEKDVLKFGFSSREYVVLHESSDT
tr|A0A0C4EL35|A0A0C4EL35_PUCT1    WRLYVFKGKEQL.DLFHIHRQSAYLFGRDRVVVDIPIDHPSSSKQHAVIQFRQICSTNEFGDSKTLVKPF.IIDLESANGTFVNGTKIPDSRYYELQSGDVIKFGCSTREFVLIPET...
tr|Q0CNP8|Q0CNP8_ASPTN            WRLYVFKGQDLL.EVVEIYERSCWLIGRERLVVDFPLDHPSCSKQHAALQFRYVEKRNEFGDRIGRVKPY.LIDLESANGSSVNGDKIPGGRYVELRDKDVLQFGLSSREYVLMLPKPE.
tr|A0A0J5PWZ6|A0A0J5PWZ6_ASPFM    WRLYVFKGEDLL.EVVELAERSCWLIGRERLVADFPLDHPSCSKQHAAIQFRYVEKRNEFGDRIGKVKPY.IIDLESANGSNVNGDTIPAGRYVELRDKDVLKFGLSSREYVLMLPQPD.
tr|F8MSL9|F8MSL9_NEUT8            WRLYVFKGDEVI.DTIELHTRSCWLVGRDLAIADLPAEHPSIKSQHAVIQFRYTEKRNEYGDKIGCVKPY.LIDLESANGTKLNGDKVPDSRYLELRDKDMIQFGSSTREYVLMLPPKD.
XP_005705899.1                    WRMYVFKNGKLLEGEFYIHQKSNYLFGRDRDVVDIPIDHPSAKSKQHAVLQFRQVMPKNGN...KLVIKPY.IMDLESTNGTFLNNERIESLRYYELLEKDLLRFGHSSREFILLTGHK..
XP_652652.1                       WRFYCFKGDEEIEHPFVMNNRSFYIFGTDKENVDIVLRHPTNEPQHAVVQFRYHNNE........ILPY.IIDLNSKEGVYLNKNRIKENVYIELRNGDVLMFGHSTREYVLLKEKPFH
XP_013763103.1                    WVLFVFDDDDGATAPLYVCSQSHFLVGSDPRVAHVVVGHQTVAPQHAVLQHRRVRKAAPD.VIRTTVKPY.IIDLNSAGGTFVNGDRVPPAKYYELRAKDVITLGTAPTEYVLVNSKAAK
EGA61315.1                        YELVIYRKNDKDKGPYDLNGRSCYLVGRELGVADIGIPEETSSKQHCVIQFRNVR.......DILKCY.VMDLDSSNGTCLNNVVIPGARYIELRSGDVLTLSEFDTDYELIF.....
RHW73524.1                        YRLRVLKEGETVATFNDISRRAVTLMGKDRGINDIPLDHPSCSAQHAALEVNFVHRNSPDGRLCAHAMELQVVDLGSTNGTNLNGELLRPLERATVIEGDVLTFGYSTRKYLIVR.....
XP_001704039.1                    ...............YIALHKPIIVVGRSAD.CDISLDSKSISGHHAKILLEGPR.............NSAVIDLNSRNGTYINDIKIKN.DSVPVYPGDTLRFGTDLPPFRLEFSVDKA
WP_103895587.1                    ...............YLIHK.VMTIGRNPD.NDIVIKDPYVSGNHARFYVKNDK..............LFVEDLNSTNGTFVNGKRISK..VEKLQEGDIIEIGRVSFKVLE.......
WP_073344202.1                    ...............NYTLYN.VTTIGRASD.CDVVLKNEFVSSKHAQIYKKGGK..............FWLDDLKSTNGTFVNGRRVRK..PVRLKSGDIISFGTEEFKFIEN......
WP_014063273.1                    ...............TFNLFE.VTTIGRSEE.CDIVIESPYVSARHAIIRKRGKR..............FYIQDLNSTNGTFVNGKRIKG..IAKIKNGDVITLGDVELKFIL.......
WP_102043286.1                    ...............RFVLKRGKTTIGRRTD.NDIILPDDSVSAQHAWILHENGH..............YRVINMLSTNGTFINDAKAH...DRPLQDGDRVRFGRAEFVFRAG...AAP
WP_017926476.1                    ...............RLTLKPGKQSVGRQGD.NDIVLQEGSVSSQHAWLVNDGGV..............CRVVNLLSTNGTWVNERKVHEA...TLNDGDHVRFGRAEFIFRRR...DDG
RMH47081.1                        ...............VFNFMNTSVTIGRNSD.QDIVIDDPSVSAMHAKITLDNGS..............WQIANLLSSNGTFVNGKKIS...VIRLNNGDRVRFGKVEFVFKAGQLTGAN
WP_015651203.1                    ...............RFLLDQPTTTAGRHPE.SDIFLDDVTVSRRHAEFRINDGE..............FEVVDVGSLNGTYVNREPRN...AQVLQTGDEIQIGKFRLVFLAG...PAE
KFD42068.1                        ...............TFYLKTSPMIIGRHDS.CDIILNDHSVSRHQAKIEYQQGK..............YILTDLESTNGTFVNGKKIT...THPLKAGDTIKAGTSLLLFNFLP.....
consensus>70                      wrlyvfk..e.l....y.hr...yl.grer.vadip.dhpscskqhav.qyr..e....dg.....v.py...dl.s.ngtf.n...ie..ryyel.e.d...fg.s.reyv.l.e....
```

```
                                         α1
Cre09.g390430.t1.1.cre.30781206       ℓℓℓℓ...........................
                                    120
Cre09.g390430.t1.1.cre.30781206       DDDD...........................
Vocar.0056s0018.1.p.vca.32896672      ..DE...........................
14945.mpu.27347251                    ...............................
DCAR_019897.dca.36084630              NCVK....S................QLSHM
Manes.03G127500.1.p.mes.32363355      ...............................
Sevir.3G283500.1.p.svi.32676284       ...............................
Seita.3G276000.1.p.sit.32712291       ...............................
Sobic.009G105800.1.p.sbi.37919000     ...............................
GRMZM2G113156_P03.zma.31024620        ...............................
Zm00008a025628_P01.zma.37219100       ...............................
Pahal.F02843.1.pha.32514749           ...............................
Pavir.Cb01329.1.p.pvi.30267644        ...............................
LOC_Os05g46780.1.osa.33156821         ...............................
Bradi4g26200.4.p.bdi.32787504         ...............................
Brast05G008800.1.p.bst.32867190       ...............................
GSVIVT01030604001.vvi.17837076        ...............................
Eucgr.F00968.1.p.egr.32052537         ...............................
Prupe.4G208000.1.p.ppe.32092913       ...............................
orange1.1g010574m.csi.18098403        ...............................
Ciclev10008061m.ccl.20793131          ...............................
Migut.H01845.1.p.mgu.28952892         ...............................
Solyc10g006390.2.1.sly.36154699       ...............................
PGSC0003DMP400025420.stu.37478734     ...............................
Brara.C03794.1.p.bra.30617963         ...............................
Bol026651.bol.37345714                ...............................
Bostr.19424s0598.1.p.adr.30661583     ...............................
Carubv10014011m.cru.20898832          ...............................
Cagra.0316s0011.1.p.cgr.28915685      ...............................
Araha.13920s0006.1.p.aha.28852390     ...............................
AL3G34380.t1.aly.35940399             ...............................
AT3G20550.1.ath.19659615              ...............................
Potri.001G420400.1.ptr.27042006       ...............................
SapurV1A.0784s0090.1.p.spu.31437518   ...............................
Medtr3g049440.1.mtr.31060687          AS.............................
Phvul.006G037500.1.p.pvu.37173844     ...............................
Glyma.09G101200.1.p.gma.30483085      Q..............................
Glyma.18G102900.1.p.gma.30556844      Q..............................
Kalax.1450s0004.1.p.kla.32537357      GE.............................
Kaladp0037s0494.1.p.kfe.35775181      GE.............................
Aqcoe1G346800.1.p.aco.33079128        V..............................
mrna03205.1-v1.0-hybrid.fve.27274674  ...............................
Aqcoe5G248600.1.p.aco.33090844        ...............................
Sphfalx0354s0008.1.p.sfa.32606484     ...............................
Pp3c19_14080V3.1.p.ppa.32939346       AS.............................
XP_008875863.1                        KSKH.S.........................
XP_002289349.1                        ...............................
tr|W5MFC8|W5MFC8_LEPOC                TEVE.EKQ...DDDDE.....GL.DE.....
tr|A0A498LC39|A0A498LC39_LABRO        AEVD.EKK....EGEEEDDE.GL.DE.....
tr|Q4V8W8|Q4V8W8_DANRE                TEVD.AKK...KEEEEEEDE.GL.DE.....
tr|H3BC77|H3BC77_LATCH                TEVD.GKQ......EDEEDE.GL.ES.....
tr|G7NUA1|G7NUA1_MACFA                SEID.RKD...DEDEEEEEE.VS.DS.....
NP_078976.2                           SEID.RKD...DEDEEEEEE.VS.DS.....
sp|Q5M9G6|SNIP1_RAT                   SELD.RKE...DEDEEEEEEMVS.DS.....
sp|Q8BIZ6|SNIP1_MOUSE                 SELD.RKE...DEDDEEEEM.VS.DS.....
tr|A0A1U7SMF0|A0A1U7SMF0_ALLSI        SEAD.TKE...EEVEEEEKS...DNS.....
tr|A0A1L8H7R2|A0A1L8H7R2_XENLA        SEVD.KKQ...DEDEDEEDE.GT.DS.....
tr|Q6DDL6|Q6DDL6_XENLA                SEVD.KKQ...DDDEDEEDE.GT.DS.....
tr|Q6PBD3|Q6PBD3_XENTR                SEVD.KKQD.DDDDEDEEDE.GT.DS.....
tr|A0A0C4EL35|A0A0C4EL35_PUCT1        ...............................
tr|Q0CNP8|Q0CNP8_ASPTN                ...............................
tr|A0A0J5PWZ6|A0A0J5PWZ6_ASPFM        ...............................
tr|F8MSL9|F8MSL9_NEUT8                ...............................
XP_005705899.1                        ...............................
XP_652652.1                          HTHLHSK.........................
XP_013763103.1                       G...............................
EGA61315.1                           ................................
RHW73524.1                           ................................
XP_001704039.1                       IEPL...PT.DQQDEDEEDHLSLTETALAEK
WP_103895587.1                       ................................
WP_073344202.1                       ..............................FN
WP_014063273.1                       ................................
WP_102043286.1                        SR.L...PS................LLVLG
WP_017926476.1                        SEVV...PT................LLEST
RMH47081.1                            GSVT...PST...............LYFLL
WP_015651203.1                        ...............................
KFD42068.1                            ...............................
consensus>70                          ...............................
```
